# Data on genome sequencing, analysis and annotation of a pathogenic *Bacillus cereus* 062011msu

**DOI:** 10.1016/j.dib.2017.12.054

**Published:** 2018-01-03

**Authors:** Rashmi Rathy, Sayan Paul, Vasanthakumar Ponesakki, Paulkumar Kanniah, Suriya Prabha Muthu, Arun Arumugaperumal, Emmanuel Joshua Jebasingh Sathiya Balasingh Thangapandi, Subburathinam Balakrishnan, Rajendhran Jeyaprakash, Sudhakar Sivasubramaniam

**Affiliations:** a Department of Biotechnology, Manonmaniam Sundaranar University, Tirunelveli, Tamil Nadu 627012, India; b Department of Genetics, School of Biological Sciences, Madurai Kamaraj University, Madurai, Tamil Nadu 625021, India

**Keywords:** *Bacillus cereus*, Genome sequencing, Abscessation, Virulence factors

## Abstract

*Bacillus* species 062011 msu is a harmful pathogenic strain responsible for causing abscessation in sheep and goat population studied by Mariappan et al. (2012) [1]. The organism specifically targets the female sheep and goat population and results in the reduction of milk and meat production. In the present study, we have performed the whole genome sequencing of the pathogenic isolate using the Ion Torrent sequencing platform and generated 458,944 raw reads with an average length of 198.2 bp. The genome sequence was assembled, annotated and analysed for the genetic islands, metabolic pathways, orthologous groups, virulence factors and antibiotic resistance genes associated with the pathogen. Simultaneously the 16S rRNA sequencing study and genome sequence comparison data confirmed that the strain belongs to the species *Bacillus cereus* and exhibits 99% sequence homo;logy with the genomes of *B. cereus* ATCC 10987 and *B. cereus* FRI-35. Hence, we have renamed the organism as *Bacillus cereus* 062011msu. The Whole Genome Shotgun (WGS) project has been deposited at DDBJ/ENA/GenBank under the accession NTMF00000000 (https://www.ncbi.nlm.nih.gov/bioproject/PRJNA404036(SAMN07629099)).

**Specifications Table**

**Table:** 

Subject area	Biology
More specific subject area	Bioinformatics (Genomics)
Type of data	Table, figure
How data was acquired	Genome sequencing: Ion Torrent personal genome machine (PGM) (Life Technologies, Carlsbad, CA),
	Denovo sequence assembly: CLC genomics workbench version 9.0.1,
	Bioinformatics approaches: NCBI Prokaryotic Genomes Automatic Annotation Pipeline (PGAAP), RAST genome annotation server (http://rast.nmpdr.org/), EggNog database integrated in BLAST2GO (eggnog.embl.de; https://www.blast2go.com/), MEGA7 (Multiple sequence alignment and phylogenetic analysis), DNAPlotter (http://www.sanger.ac.uk/science/tools/dnaplotter).
Data format	Analyzed
Experimental factors	Genome sequencing, genome annotation, KEGG pathway analysis, orthologous group analysis, 16S rRNA and 23S rRNA based phylogeny.
Experimental features	The whole genome sequencing of *Bacillus cereus* 062011msu was performed by using Ion Torrent Personal Genome Machine (PGM) platform. Quality analysis, filtering and de novo assembly of the raw reads were performed by CLC genomics workbench 9.0.1. Genome annotation was done by using the PGAAP pipeline and RAST genome annotation server. Multiple sequence alignment and phylogenetic analysis based on 16S rRNA and 23S rRNA sequences were performed by the MEGA7 tool. The circular genome map of our species was generated by DNAPlotter.
Data source location	Maruthamputhur village, Alangulam, Tirunelveli District, Tamil Nadu, India. (latitude: 8.8646N and longitude: 77.4960 E).
Data accessibility	Genome analysis and annotation data are given within this article and the raw data along with NCBI PGAAP annotation were deposited at NCBI repository:
	https://www.ncbi.nlm.nih.gov/bioproject/PRJNA404036,
	Bioproject ID: 404036, BioSample: SAMN07629099
	The Whole Genome Shotgun (WGS) project has been deposited at DDBJ/ENA/GenBank under the accession NTMF00000000 (https://www.ncbi.nlm.nih.gov/nuccore/NTMF00000000)
	The genome annotation data obtained from the RAST server are given in this article.
Related research article	“Bacillus sp. causing abscessation in sheep and goat population” by Mariappan et al. (2012) [1].

**Value of the data**

• The *Bacillus cereus* 062011msu is a deadly pathogenic bacterium known for causing abscess mainly in the female sheep and goat population. Hence, the genome sequence resource and their annotation details can be effectively utilized to understand the pathogenicity of the bacterium for the benefit of the farmers who rear the sheep and goat.
• The genome annotation data of *Bacillus cereus* 062011msu provided a broad overview regarding the subsystem features, metabolic pathways, orthologous groups, virulence factors and antibiotic resistant genes associated with the genome of the species. Most of the unique genes of the species were found to be clustered in ten genetic islands. In this study we provided a detailed analysis of the genes clustered on the genetic islands.
• The data obtained from 16S rRNA analysis and genome sequence comparison with other *Bacillus* species provided significant information regarding the identification and taxonomic classification of this new bacterial strain. Although according to the previous study using the partial 16S RNA sequences the pathogen was reported to be genetically similar to *Bacillus anthracis* [1], but the whole genome data confirmed that the strain is in fact belongs to the species *Bacillus cereus* and phylogenetically related with *B. cereus* ATCC 10987 and *B. cereus* FRI-35.
• The entire genome dataset can be utilized further for determining the genes and biochemical pathways related to the pathogenicity (abscess) of the strain and developing new antimicrobial drugs for the pathogen.

## 1 Data

**Fig. 1:**
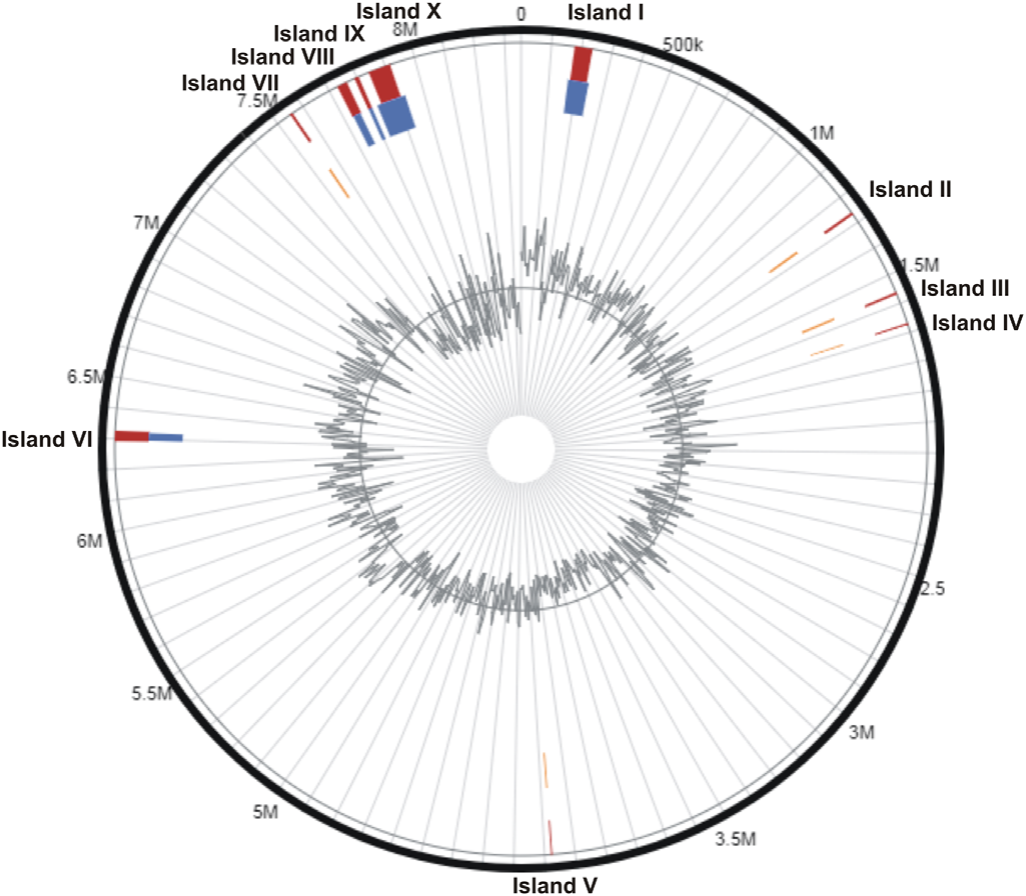
Genetic islands in the *Bacillus cereus* 062011msu. Total 10 genetic islands were predicted by using the Island Viewer 4.

**Fig. 2:**
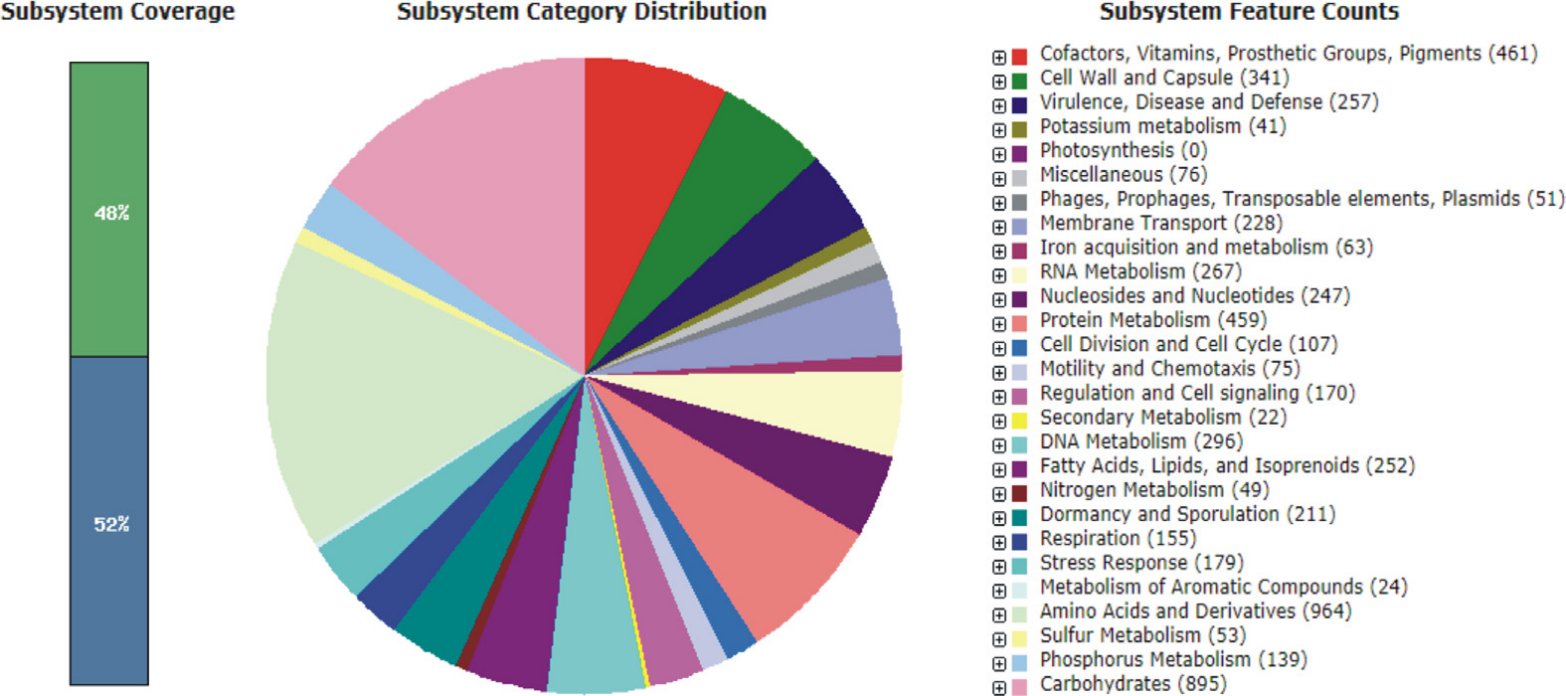
Subsystem distribution of *Bacillus cereus* 062011msu genome based on RAST annotation server.

**Fig. 3:**
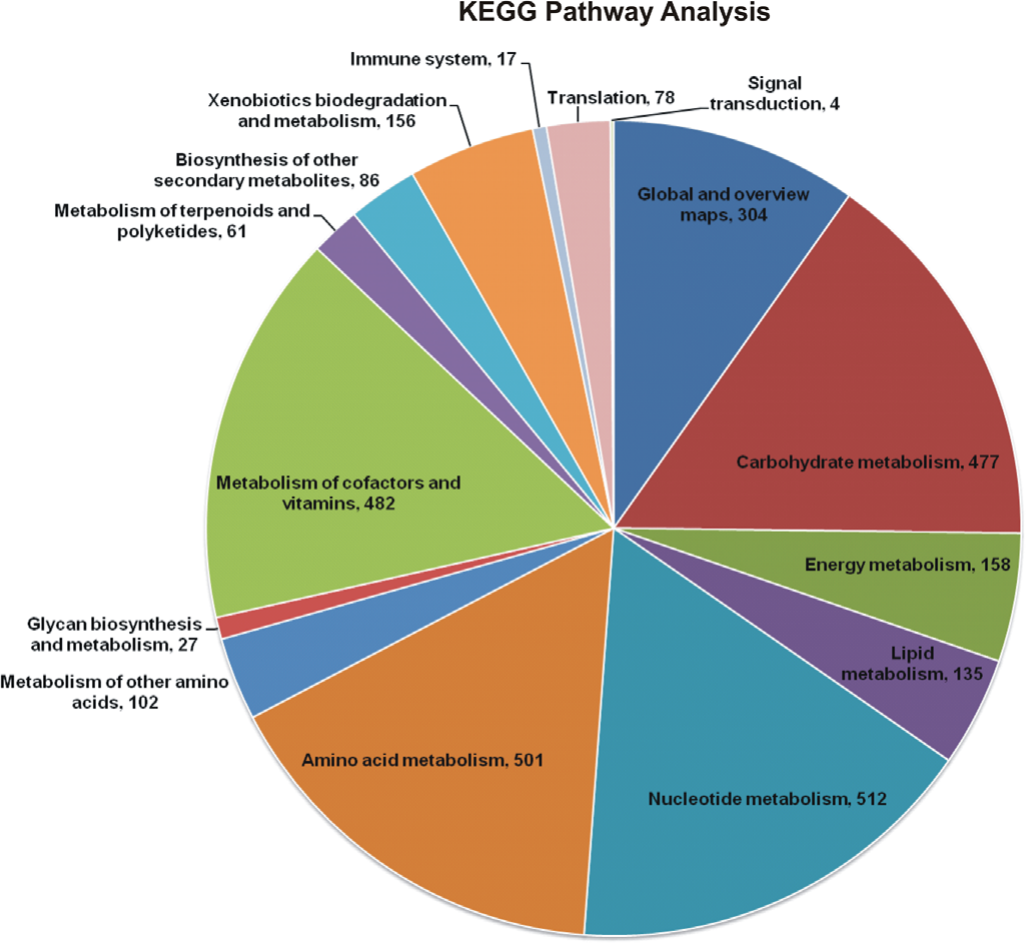
Pie chart representing the distribution of KEGG pathways associated with the genome of *Bacillus cereus* 062011msu. The pathways were obtained by annotating the protein coding sequences against the KEGG database.

**Fig. 4:**
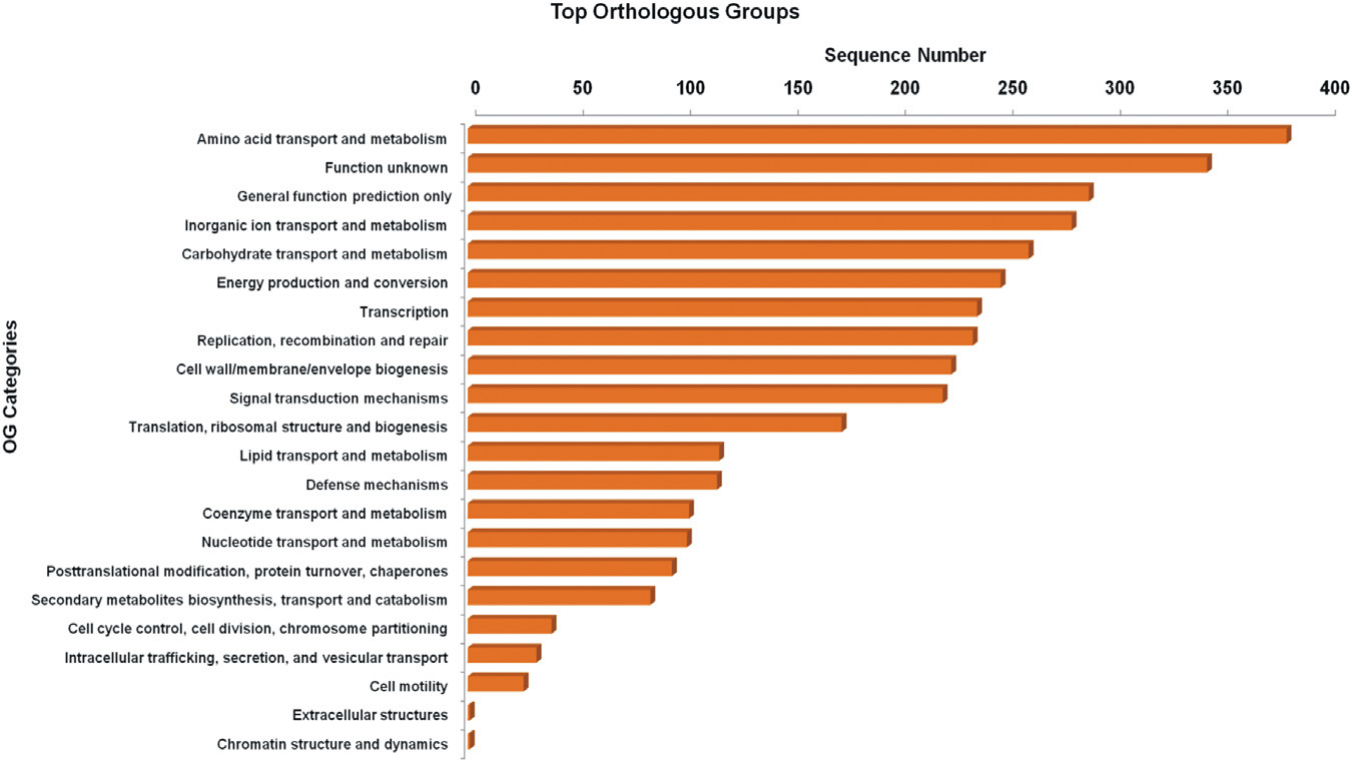
Clusters of Orthologous Groups (COG) distribution of the protein coding genes in *Bacillus cereus* 062011msu obtained from EggNog database.

**Fig. 5:**
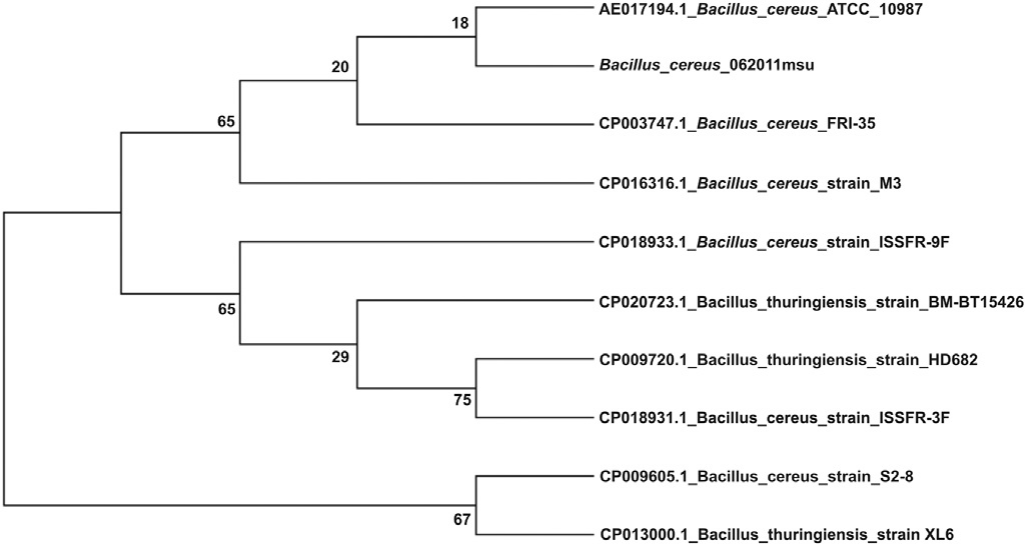
Phylogenetic tree based on 16S rRNA comparison of *Bacillus cereus* 062011msu with its closely related homologs using the MEGA7 software.

**Fig. 6:**
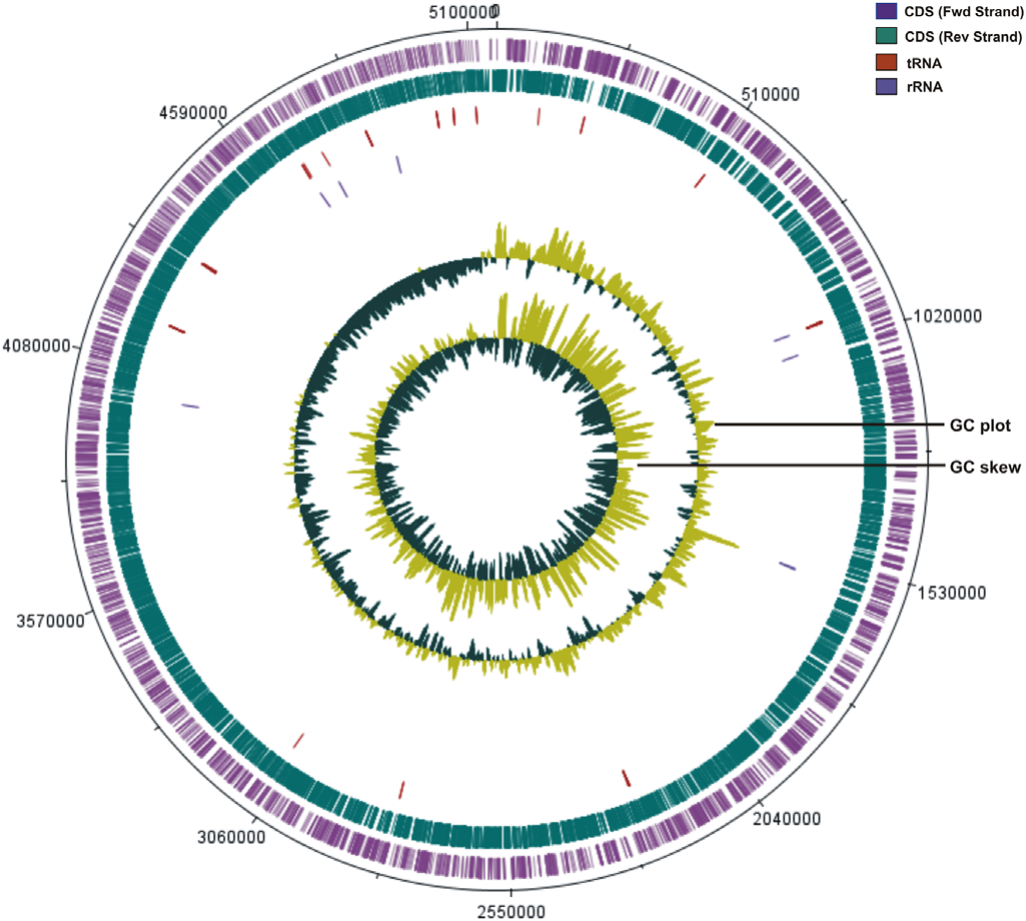
Circular genome map of *Bacillus cereus* 062011msu generated by DNAPlotter tool.

**Fig. S1:**
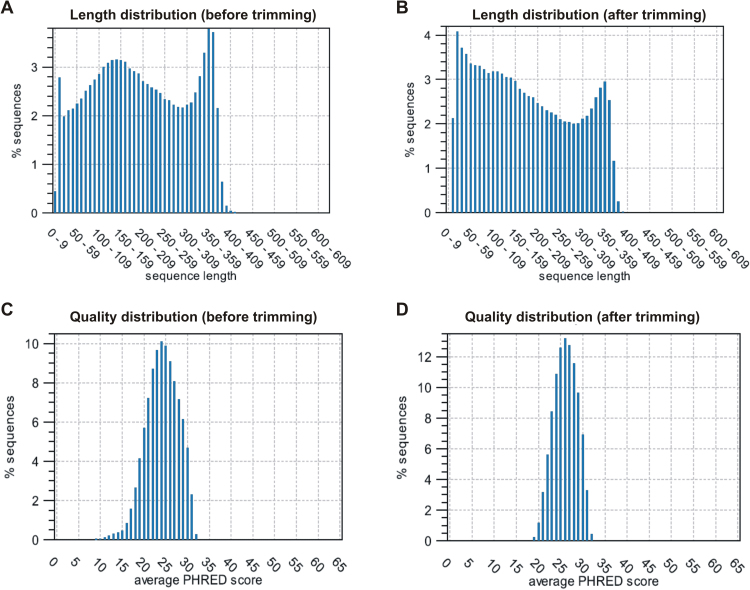
Histogram representing the **(A)** length distribution of the raw reads (before trimming). **(B)** Length distribution of the filtered reads (after trimming). **(C)** Phred quality score distribution of the raw reads (before trimming). **(D)** Phred quality score distribution of the filtered reads (after trimming).

**Fig. S2:**
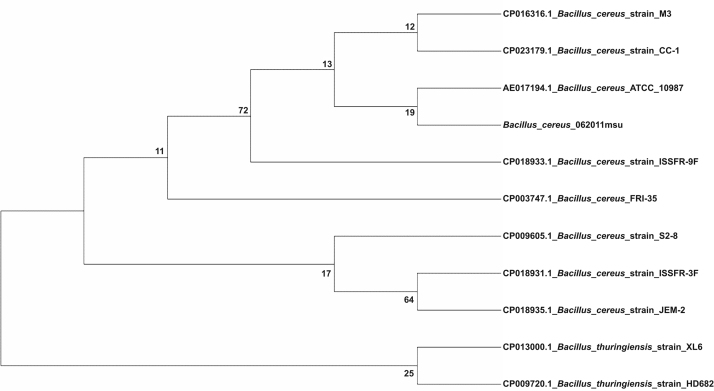
Phylogenetic tree obtained from 23S rRNA comparison of *Bacillus cereus* 062011msu with its closely related homologs using the MEGA7 software.

The overall data represents the genome sequencing, assembly, annotation and comparative analysis of pathogenic bacteria *Bacillus cereus* 062011msu. Table 1 denotes the summary statistics of the draft genome assembly of the *B. cereus* 062011msu. The data describing the length and Phred quality score distribution of the raw and filtered reads are illustrated in Supplementary Fig. S1. Data on Fig. 1 represent 10 genetic islands predicted in the genome of the isolate. The details of the genes clustered on the genetic islands are shown in Supplementary Table S1. Fig. 2 shows the subsystem distribution of *the B. cereus* 062011msu genome based on RAST genome annotation. The complete list of the RAST annotated genes is given in Supplementary Table S2. Fig. 3 gives a complete overview of the KEGG (Kyoto Encyclopedia of Genes and Genomes) pathways associated with the annotated genome sequence. The data illustrated in Fig. 4 show the Clusters of Orthologous Groups (COG) distribution of the protein coding genes obtained from RAST annotation. Table 2 denotes the list of virulence factors (with complete homology) identified in the annotated genome dataset. The data regarding the antibiotic resistance genes identified in the pathogen are given in Supplementary Table S3. The top 20 closest neighboring strains of *Bacillus cereus* 062011msu based on RAST annotation are listed in Supplementary Table S4. Supplementary Table S5 portrays the top 10 species showing maximum sequence homology with the genome of *Bacillus cereus* 062011msu resulted from BLAST genome alignment. Fig. 5 represents the phylogenetic tree constructed based on 16S rRNA comparison of the strain with its closely related homologs. Simultaneously the phylogenetic analysis data obtained from 23S rRNA comparison study are depicted in Fig. S2. Data on Fig. 6 represents the visualization of the annotated circular genome map of *B. cereus* 062011msu obtained from DNAPlotter.

**Table 1:** Summary statistics of *Bacillus cereus* 062011msu genome assembly.

**No. of raw reads**	**458,944**
average quality PHRED-score Q20 (before trimming)	26,143 (abs)
average quality PHRED-score Q25 (before trimming)	45,447 (abs)
average quality PHRED-score Q30 (before trimming)	21,587 (abs)
No. of clean reads	432,619
Average length of clean reads	178.08
average quality PHRED-score Q20 (after trimming)	5096 (abs)
average quality PHRED-score Q25 (after trimming)	54,520 (abs)
average quality PHRED-score Q30 (after trimming)	29,972 (abs)
Percentage of clean reads	94.27%
Total no. of assembled contigs	3200
Mean contig length	1611
N50 length	2922
N25 length	5365
Sequencing depth	15X
GC%	35.3%
Size of genome	5,154,790

Abs: number of sequences observed at that quality score.

**Table 2:** List of virulence factors identified in the annotated genome dataset of *Bacillus cereus* 062011msu.

SeqName	Start	Stop	Strand	Description	Length	e-Value	Sim mean
contig_1017	240	1	−	Flagellum-specific ATP synthase	240	6.88E−53	100
contig_1017	1466	1305	−	Flagellar assembly protein H	162	3.05E−15	100
contig_1017	2984	1980	−	Flagellar motor switch protein G	1005	0	100
contig_1017	3453	2998	−	Flagellar MS-ring protein	456	1.33E−40	100
contig_1026	114	1106	+	UDP-glucose 4-epimerase	993	0	100
contig_1050	1366	557	−	UTP-glucose-1-phosphate uridylyltransferase	810	0	100
contig_1052	162	16	−	Flagellar biosynthesis protein FliR	147	1.96E−12	100
contig_1052	305	192	−	Flagellar biosynthesis protein FliQ	114	4.26E−20	100
contig_1052	1144	503	−	Flagellar biosynthesis protein FliP	642	1.91E−130	100
contig_1052	1792	1601	−	Flagellar motor switch protein FliN	192	7.43E−39	100
contig_1108	28	837	+	Flagellar motor protein MotB	810	6.97E−171	100
contig_1140	301	1116	+	Flagellar biosynthesis regulator FlhF	816	0	100
contig_1140	1159	1497	+	Flagellar basal body rod protein FlgG	339	6.74E−76	100
contig_1208	569	387	−	Flagellar hook protein FlgE	183	5.15E−35	100
contig_1208	1752	1633	−	Flagellar basal body rod modification protein	120	1.19E−10	100
contig_1235	13	726	+	Immune inhibitor A metalloprotease	714	9.71E−152	100
contig_1277	720	535	−	Glycosyl transferase, group 2 family protein	186	8.07E−19	100
contig_1321	1747	1574	−	Chemotaxis protein CheV	237	1.09E−50	100
contig_1339	805	488	−	Nonhemolytic enterotoxin NHE	144	2.77E−25	100
contig_1358	522	379	−	Thiol-activated cytolysin	837	0	100
contig_1613	547	1092	+	Caspsular polysaccharide biosynthesis protein	264	4.12E−52	100
contig_1634	912	76	−	Hypothetical protein NEAT-type hemophore-mediated heme uptake system	585	4.82E−92	100
contig_1673	392	93	−	Flagellar hook-basal body protein FliE	300	1.15E−69	100
contig_1673	834	538	−	Flagellar basal body rod protein FlgC	297	1.71E−68	100
contig_1912	261	37	−	Non-hemolytic enterotoxin A	225	2.03E−42	100
contig_1926	852	496	−	Flagellar protein FliS, putative	357	2.32E−74	100
contig_1926	1621	977	−	Flagellar capping protein	645	4.44E−148	100
contig_2028	167	21	−	Flagellar motor protein MotS	147	1.05E−29	100
contig_2028	1158	886	−	Flagellar motor protein MotP	273	1.14E−33	100
contig_2037	1066	857	−	Chemotaxis protein methyltransferase CheR	210	3.60E−45	100
contig_2173	701	471	−	Flagellar biosynthesis protein FliR	231	7.44E−40	100
contig_2221	38	262	+	Hemolysin III	225	8.80E−48	100
contig_2339	364	29	−	Glycosyl transferase, group 1 family protein	336	5.30E−77	100
contig_2555	475	242	−	Flagellar hook-associated protein FlgL	234	2.66E−48	100
contig_2723	583	191	−	O-antigen polymerase wzy	393	5.21E−71	100
contig_35	1687	995	−	UDP-galactose phosphate transferase	693	1.77E−168	100
contig_35	2379	1687	−	Aminotransferase family protein	693	6.51E−174	100
contig_439	4672	5061	+	Transcriptional regulator PlcR, putative	390	3.32E−90	100
contig_572	306	88	−	NAD dependent epimerase/dehydratase family protein	219	2.36E−44	100
contig_572	1657	284	−	UDP-glucose 6-dehydrogenase	1374	0	100
contig_572	2422	1751	−	Polysaccharide transport protein, putative	672	9.86E−147	100
contig_582	743	630	−	Flagellar hook-associated protein FlgK	114	2.91E−19	100
contig_681	545	45	−	Internalin, putative	501	1.98E−116	100
contig_694	5767	5450	−	Channel protein, hemolysin III family	318	5.60E−57	100
contig_716	152	9	−	Cytotoxin K	144	4.42E−24	100
contig_734	1083	232	−	Phospholipase C	852	0	100
contig_875	1156	1413	+	Iron compound ABC transporter iron compound-binding protein	258	3.82E−40	100
contig_875	2446	2847	+	Iron compound ABC transporter permease protein	402	8.95E−50	100
contig_876	733	329	−	Capsular exopolysaccharide family protein	405	1.42E−75	100
contig_882	3022	2846	−	Flagellar motor switch protein	177	1.30E−33	100
contig_882	4973	4479	−	Chemotaxis histidine kinase	495	5.15E−94	100
contig_899	782	63	−	Capsular polysaccharide biosynthesis protein	720	6.46E−178	100
contig_899	1383	1264	−	Tyrosine-protein kinase	120	9.58E−21	100
contig_903	2407	1010	−	Flagellin	1398	0	100
contig_965	15	983	+	Non-hemolytic enterotoxin C	969	0	100
contig_986	1110	1253	+	Membrane-bound transcriptional regulator LytR	144	2.26E−10	100

## 2 Experimental design, materials and methods

### 2.1 Genome sequencing, quality assessment and de novo assembly

The *Bacillus cereus* 062011msu was isolated from the abscess tissue of the affected female sheep and goats in Maruthamputhur village near Alangulam Region, Tirunelveli District, Tamil Nadu, India [1]. The whole genome sequencing of the species using Ion Torrent personal genome machine (Life Technologies, Carlsbad, CA) [2] produced 458,944 raw reads having average length of 198.2 bp and total size of 90,974,357 bp (90.974 MB). The FastQC (version.0.11.5) plug-in software (https://www.bioinformatics.babraham.ac.uk/projects/fastqc/) [3] and CLC genomics workbench version 9.0.1 [4] were used for analyzing the read quality and trimming of ambiguous low quality reads. After quality assessment and trimming total 432,619 cleaned reads were obtained with an average length of 178.08 bp. The trimmed reads were assembled into 3,200 contigs with an average length of 1,611 bp and GC content of 35.3% using the denovo assembly algorithm of CLC Genomics Workbench version 9.0.1.

### 2.2 Genome sequence annotation and genomic data analysis

The draft genome contigs of *Bacillus cereus* 062011 msu were annotated by using the NCBI Prokaryotic Genomes Automatic Annotation Pipeline (PGAAP) [5] and Rapid Annotation of microbial genome using Subsystem Technology (RAST) version 2.0 (http://rast.nmpdr.org/) [6]. The PGAAP annotation of the isolate's genome showed total 7,061 CDS (2,301 protein coding genes and 4,760 pseudo genes) and 81 RNA genes including 66 tRNAs, 10 rRNAs and 5 ncRNAs. The annotation details were given in whole genome shotgun (WGS) project with the project accession NTMF00000000. The genomic islands are set of genes with probable horizontal origin which facilitate in the diversification, adaptation and evolution of pathogenic microbes [7], [8]. The Genomic islands in our study were predicted by submitting the PGAAP generated GenBank file to the Island Viewer 4 (http://www.pathogenomics.sfu.ca/islandviewer/) [8]. Total 219 genes were clustered on 10 genetic islands.

Simultaneously the data obtained from the RAST annotation server revealed that the draft genome contains 8721 coding sequences and 472 subsystems with “Amino Acids and Derivatives” and “Carbohydrates” were the most represented subsystem features. In addition the annotated subsystem features denoted 257 genes associated with “Virulence, Disease and Defense” including 154 genes associated with antibiotics and toxicity resistance, 52 genes associated with the synthesis of antibacterial peptides, Bacteriocins, 50 genes associated with invasion and intracellular resistance and one gene associated with adhesion. The KEGG (Kyoto Encyclopedia of Genes and Genomes) biological pathways associated with the genome of *Bacillus cereus* 062011msu were identified by annotating the protein coding sequences against the KEGG pathway database using the BLAST2GO program [9]. A total of 3104 sequences were mapped to 116 different KEGG pathways. Among them the pathways associated with Nucleotide metabolism, Amino acid metabolism, Metabolism of cofactors and vitamins and Carbohydrate metabolism were the most dominant KEGG pathways observed in the genome dataset. The prediction and classification of the orthologous groups associated with the Protein coding genes of *Bacillus cereus* 062011msu were performed by using the EggNog database (Evolutionary genealogy of genes) embedded within the BLAST2GO software [10]. The COG (Clusters of Orthologous Groups) data denoted that the cluster for “Amino acid transport and metabolism” (381 sequences) forms the largest functional group. Among the other functional groups the clusters for “Function unknown” (344 sequences), “General Function Prediction Only” (289 sequences), “Inorganic ion transport and metabolism” (281 sequences) and “Carbohydrate transport and metabolism” (261 sequences) were the highly represented categories.

Emphasizing the pathogenic nature of the strain, the virulence factors and toxic genes residing in the genome of *Bacillus cereus* 062011msu were further screened by annotating the coding sequences against the Virulence Factor Database (VFDB) [11] using the local BLASTX with E-value cutoff of 1E-5. A total of 1108 sequences homologous to 743 virulence factors and toxic genes were identified from the BLAST search. Among them 56 genes showed complete sequence homology (100%) with the annotated genome dataset of *B. cereus* 062011msu, indicating that the flagellar proteins might play regulatory role in the pathogenicity of the bacterium. The previous in vitro experiments by Mariappan et al., 2012 reported that the pathogen was sensitive to tetracycline (TET) and ciprofloxacin (CPFX) [1]. The antibiotic resistance genes present in *Bacillus cereus* 062011msu were screened by using the curated database, Antibiotic Resistance Genes Database (ARDB) (http://ardb.cbcb.umd.edu/) [12]. The data illustrated that the pathogen consists of total 14 crucial antibiotics resistance genes exhibiting resistance to the antibiotics like bacitracin, penicillin, fosfomycin, streptogramin_a, chloramphenicol, doxorubicin, fluoroquinolone, puromycin, streptomycin, beta_lactam, lincomycin and fosmidomycin, thus confirming the susceptibility of the strain to TET and CPFX.

### 2.3 Genome sequence comparison, 16S and 23S rRNA analysis, and genome map visualization

The closest neighboring strains for *Bacillus cereus* 062011msu based on the genome sequence comparison using RAST server were identified as *Bacillus cereus* AND1407 (score 544), *Bacillus cereus* MSX-D12 (score 406) and *Bacillus cereus* BAG3O-2 (score 387). Based on the local similarity of the aligned nucleotide sequences using the rapid sequence comparison tool BLAST [13] the genome of *Bacillus cereus* 062011msu exhibited 99% sequence homology with the genomes of *Bacillus cereus* ATCC 10987, *Bacillus cereus* strain M3, *Bacillus cereus* FRI-35, *Bacillus thuringiensis* serovar finitimus YBT-020, *Bacillus cereus* strain CC-1, *Bacillus cereus* NC7401, *Bacillus cereus* AH187 respectively. In microbial genomics research the comparison of 16S rRNA gene sequence has emerged as a reliable technique to identify new bacterial strains associated with pathogenicity and infections [14]. The deduced 16S rRNA sequence for *Bacillus cereus* 062011msu genome was aligned to its nearby homologs using the Clustal W multiple sequence alignment and the phylogenetic analysis was performed through the maximum likelihood method with 100 bootstrap replicates using the MEGA7 software (www.megasoftware.net/) [15]. The phylogenetic tree based on 16S rRNA sequence comparison confirmed that the pathogenic strain belongs to the species *Bacillus cereus* and exhibits close evolutionary relationship with *B. cereus* ATCC 10987 and *B. cereus* FRI-35 as they were clustered together as a monophyletic clade. Simultaneously we have also performed the phylogenetic analysis based on 23S rRNA gene sequence comparison using the MEGA7 software. The 16S rRNA gene derived phylogenetic tree was found to be concordant with the 23S rRNA gene tree as it also identified *B. cereus* ATCC 10987 as the closest evolutionary homolog of the pathogen. The complete genomic map of *Bacillus cereus* 062011msu representing the GC content, GC skew graphs, coordinates and coding sequence features on both forward and reverse strands obtained from RAST annotation was generated by DNAPlotter (http://www.sanger.ac.uk/science/tools/dnaplotter) [16].
