# Supplementary material for: Data on genome sequencing, analysis and annotation of a pathogenic Bacillus cereus 062011msu
Source: Data Brief. 2018 Jan 3;17:15–23. doi: 10.1016/j.dib.2017.12.054 (PMC5988026; doi:10.1016/j.dib.2017.12.054)
Supplement: Supplementary file 1 — Transparency document [file mmc1.zip › Conflict of Interests.pdf]

Conflict of Interests:

The authors declare no potential conflicts of interest.
