# Supplementary material for: Data on genome sequencing, analysis and annotation of a pathogenic Bacillus cereus 062011msu
Source: Data Brief. 2018 Jan 3;17:15–23. doi: 10.1016/j.dib.2017.12.054 (PMC5988026; doi:10.1016/j.dib.2017.12.054)
Supplement: Supplementary file 2 — Supplementary material [file mmc4.docx]

Table S1: List of genes clustered in the genetic islands of *Bacillus cereus* 062011msu

| **Island start** | **Island end** | **Length** | **Gene start** | **Gene end** | **Strand** | **Description** |
| --- | --- | --- | --- | --- | --- | --- |
| 176521 | 234722 | 58201 | 176521 | 176766 | 1 | hypothetical protein |
| 176521 | 234722 | 58201 | 178116 | 178337 | 1 | hypothetical protein |
| 176521 | 234722 | 58201 | 179584 | 179846 | 1 | hypothetical protein |
| 176521 | 234722 | 58201 | 181267 | 182013 | 1 | hypothetical protein |
| 176521 | 234722 | 58201 | 183501 | 183746 | 1 | hypothetical protein |
| 176521 | 234722 | 58201 | 183787 | 184032 | 1 | hypothetical protein |
| 176521 | 234722 | 58201 | 184013 | 184564 | 1 | hypothetical protein |
| 176521 | 234722 | 58201 | 185968 | 186204 | -1 | XRE family transcriptional regulator |
| 176521 | 234722 | 58201 | 186454 | 187062 | 1 | IS4 family transposase |
| 176521 | 234722 | 58201 | 187175 | 187369 | 1 | transcriptional regulator |
| 176521 | 234722 | 58201 | 187489 | 187872 | -1 | aspartate--tRNA(Asn) ligase |
| 176521 | 234722 | 58201 | 189014 | 189208 | 1 | hypothetical protein |
| 176521 | 234722 | 58201 | 189429 | 189635 | -1 | hypothetical protein |
| 176521 | 234722 | 58201 | 190650 | 190896 | 1 | hemolysin D |
| 176521 | 234722 | 58201 | 191897 | 192583 | 1 | hemolysin D |
| 176521 | 234722 | 58201 | 193611 | 194194 | 1 | autotransporter |
| 176521 | 234722 | 58201 | 194215 | 194486 | 1 | hypothetical protein |
| 176521 | 234722 | 58201 | 194664 | 195129 | -1 | hypothetical protein |
| 176521 | 234722 | 58201 | 195372 | 195911 | 1 | hypothetical protein |
| 176521 | 234722 | 58201 | 196997 | 197801 | 1 | heteromeric transposase endonuclease subunit TnsA |
| 176521 | 234722 | 58201 | 198803 | 198955 | 1 | heteromeric transposase endonuclease subunit TnsA |
| 176521 | 234722 | 58201 | 199006 | 199206 | 1 | hypothetical protein |
| 176521 | 234722 | 58201 | 199175 | 199369 | 1 | hypothetical protein |
| 176521 | 234722 | 58201 | 200370 | 200659 | 1 | DNA-binding protein |
| 176521 | 234722 | 58201 | 201660 | 203210 | 1 | DNA-binding protein |
| 176521 | 234722 | 58201 | 204245 | 205436 | 1 | transposase |
| 176521 | 234722 | 58201 | 206510 | 207213 | 1 | hypothetical protein |
| 176521 | 234722 | 58201 | 207191 | 207469 | 1 | hypothetical protein |
| 176521 | 234722 | 58201 | 207411 | 208310 | 1 | hypothetical protein |
| 176521 | 234722 | 58201 | 208267 | 209058 | 1 | hypothetical protein |
| 176521 | 234722 | 58201 | 209087 | 209992 | 1 | hypothetical protein |
| 176521 | 234722 | 58201 | 209962 | 210402 | 1 | hypothetical protein |
| 176521 | 234722 | 58201 | 210386 | 210916 | 1 | hypothetical protein |
| 176521 | 234722 | 58201 | 211234 | 212555 | 1 | hypothetical protein |
| 176521 | 234722 | 58201 | 212602 | 213111 | 1 | hypothetical protein |
| 176521 | 234722 | 58201 | 214219 | 215010 | 1 | hypothetical protein |
| 176521 | 234722 | 58201 | 216011 | 216458 | 1 | hypothetical protein |
| 176521 | 234722 | 58201 | 217893 | 218727 | 1 | heteromeric transposase endonuclease subunit TnsA |
| 176521 | 234722 | 58201 | 218724 | 219213 | 1 | transposase |
| 176521 | 234722 | 58201 | 220214 | 220767 | 1 | transposase |
| 176521 | 234722 | 58201 | 220748 | 221251 | 1 | hypothetical protein |
| 176521 | 234722 | 58201 | 221196 | 221411 | 1 | hypothetical protein |
| 176521 | 234722 | 58201 | 221448 | 221627 | 1 | hypothetical protein |
| 176521 | 234722 | 58201 | 221661 | 223293 | 1 | transposase |
| 176521 | 234722 | 58201 | 223297 | 224265 | 1 | hypothetical protein |
| 176521 | 234722 | 58201 | 224253 | 224525 | 1 | hypothetical protein |
| 176521 | 234722 | 58201 | 224539 | 225045 | 1 | hypothetical protein |
| 176521 | 234722 | 58201 | 225192 | 225911 | 1 | hypothetical protein |
| 176521 | 234722 | 58201 | 225880 | 226077 | 1 | hypothetical protein |
| 176521 | 234722 | 58201 | 226089 | 226316 | 1 | hypothetical protein |
| 176521 | 234722 | 58201 | 226372 | 226683 | 1 | hypothetical protein |
| 176521 | 234722 | 58201 | 227085 | 227516 | 1 | hypothetical protein |
| 176521 | 234722 | 58201 | 227513 | 228067 | 1 | hypothetical protein |
| 176521 | 234722 | 58201 | 228192 | 228446 | 1 | hypothetical protein |
| 176521 | 234722 | 58201 | 230751 | 232712 | 1 | S9 family peptidase |
| 176521 | 234722 | 58201 | 232816 | 233384 | 1 | DUF4256 domain-containing protein |
| 176521 | 234722 | 58201 | 234499 | 234722 | -1 | hypothetical protein |
| 1261763 | 1270274 | 8511 | 1261763 | 1262014 | -1 | chromosome partitioning protein ParB |
| 1261763 | 1270274 | 8511 | 1263555 | 1263932 | 1 | hypothetical protein |
| 1261763 | 1270274 | 8511 | 1264933 | 1265534 | 1 | replication protein |
| 1261763 | 1270274 | 8511 | 1265522 | 1265913 | 1 | replicative DNA helicase |
| 1261763 | 1270274 | 8511 | 1266914 | 1267397 | 1 | replicative DNA helicase |
| 1261763 | 1270274 | 8511 | 1268499 | 1268817 | -1 | TrmB family transcriptional regulator |
| 1261763 | 1270274 | 8511 | 1269818 | 1270274 | -1 | TrmB family transcriptional regulator |
| 1561155 | 1568745 | 7590 | 1561155 | 1561461 | 1 | hypothetical protein |
| 1561155 | 1568745 | 7590 | 1562530 | 1563374 | 1 | HAD family hydrolase |
| 1561155 | 1568745 | 7590 | 1564375 | 1564665 | 1 | hypothetical protein |
| 1561155 | 1568745 | 7590 | 1565666 | 1568745 | 1 | HAD family hydrolase |
| 1668812 | 1674267 | 5455 | 1668812 | 1669047 | -1 | spore coat protein |
| 1668812 | 1674267 | 5455 | 1670048 | 1670299 | -1 | spore coat protein |
| 1668812 | 1674267 | 5455 | 1670442 | 1670707 | -1 | ATP-dependent DNA helicase |
| 1668812 | 1674267 | 5455 | 1671708 | 1673743 | -1 | ATP-dependent DNA helicase |
| 1668812 | 1674267 | 5455 | 1673843 | 1674267 | -1 | GNAT family N-acetyltransferase |
| 4073903 | 4078989 | 5086 | 4073903 | 4074839 | 1 | aminotransferase |
| 4073903 | 4078989 | 5086 | 4074879 | 4076310 | 1 | autoinducer-2 kinase |
| 4073903 | 4078989 | 5086 | 4077311 | 4077519 | 1 | autoinducer-2 kinase |
| 4073903 | 4078989 | 5086 | 4078627 | 4078989 | 1 | cupin domain-containing protein |
| 6292141 | 6326885 | 34744 | 6292141 | 6292241 | 1 | phosphohydrolase |
| 6292141 | 6326885 | 34744 | 6293489 | 6294046 | 1 | phosphohydrolase |
| 6292141 | 6326885 | 34744 | 6294186 | 6294879 | 1 | CPBP family intramembrane metalloprotease |
| 6292141 | 6326885 | 34744 | 6296053 | 6296810 | 1 | CPBP family intramembrane metalloprotease |
| 6292141 | 6326885 | 34744 | 6296785 | 6297105 | -1 | RNA polymerase subunit sigma-70 |
| 6292141 | 6326885 | 34744 | 6298250 | 6298904 | -1 | HlyD family secretion protein |
| 6292141 | 6326885 | 34744 | 6299202 | 6299820 | 1 | TetR/AcrR family transcriptional regulator |
| 6292141 | 6326885 | 34744 | 6299861 | 6300948 | -1 | peptidase M28 |
| 6292141 | 6326885 | 34744 | 6301018 | 6301509 | -1 | dUTPase |
| 6292141 | 6326885 | 34744 | 6302819 | 6303172 | -1 | 50S ribosomal protein L20 |
| 6292141 | 6326885 | 34744 | 6303210 | 6303410 | -1 | 50S ribosomal protein L35 |
| 6292141 | 6326885 | 34744 | 6303432 | 6303992 | -1 | translation initiation factor IF-3 |
| 6292141 | 6326885 | 34744 | 6304329 | 6305127 | -1 | threonine--tRNA ligase |
| 6292141 | 6326885 | 34744 | 6306128 | 6307062 | -1 | threonine--tRNA ligase |
| 6292141 | 6326885 | 34744 | 6307382 | 6308256 | -1 | putative sporulation protein YtxC |
| 6292141 | 6326885 | 34744 | 6308535 | 6309473 | -1 | primosomal protein DnaI |
| 6292141 | 6326885 | 34744 | 6309507 | 6310552 | -1 | Replication initiation and membrane attachment protein |
| 6292141 | 6326885 | 34744 | 6311591 | 6312013 | -1 | Replication initiation and membrane attachment protein |
| 6292141 | 6326885 | 34744 | 6312141 | 6312602 | -1 | transcriptional regulator NrdR |
| 6292141 | 6326885 | 34744 | 6313650 | 6314045 | -1 | S-adenosylmethionine decarboxylase proenzyme |
| 6292141 | 6326885 | 34744 | 6314200 | 6314333 | -1 | hypothetical protein |
| 6292141 | 6326885 | 34744 | 6315416 | 6316444 | -1 | type I glyceraldehyde-3-phosphate dehydrogenase |
| 6292141 | 6326885 | 34744 | 6316554 | 6317159 | -1 | dephospho-CoA kinase |
| 6292141 | 6326885 | 34744 | 6317211 | 6317688 | -1 | sporulation membrane protein YtaF |
| 6292141 | 6326885 | 34744 | 6318689 | 6318987 | -1 | sporulation membrane protein YtaF |
| 6292141 | 6326885 | 34744 | 6319060 | 6319403 | -1 | DNA-formamidopyrimidine glycosylase |
| 6292141 | 6326885 | 34744 | 6320404 | 6320910 | -1 | DNA-formamidopyrimidine glycosylase |
| 6292141 | 6326885 | 34744 | 6320923 | 6323058 | -1 | DNA polymerase I |
| 6292141 | 6326885 | 34744 | 6324059 | 6324296 | -1 | hypothetical protein |
| 6292141 | 6326885 | 34744 | 6324539 | 6326305 | -1 | PAS domain-containing sensor histidine kinase |
| 6292141 | 6326885 | 34744 | 6326298 | 6326885 | -1 | DNA-binding response regulator |
| 7550953 | 7560100 | 9147 | 7550953 | 7551281 | -1 | type IV secretion protein Rhs |
| 7550953 | 7560100 | 9147 | 7552285 | 7553088 | 1 | hypothetical protein |
| 7550953 | 7560100 | 9147 | 7553115 | 7553374 | 1 | hypothetical protein |
| 7550953 | 7560100 | 9147 | 7553407 | 7553748 | 1 | hypothetical protein |
| 7550953 | 7560100 | 9147 | 7554290 | 7554503 | -1 | hypothetical protein |
| 7550953 | 7560100 | 9147 | 7557000 | 7557670 | -1 | hypothetical protein |
| 7550953 | 7560100 | 9147 | 7558688 | 7558864 | -1 | RsfA family transcriptional regulator |
| 7550953 | 7560100 | 9147 | 7559948 | 7560100 | 1 | UDP-N-acetylmuramoylalanyl-D-glutamate--2, 6-diaminopimelate ligase |
| 7729666 | 7764130 | 34464 | 7729496 | 7729675 | -1 | hypothetical protein |
| 7729666 | 7764130 | 34464 | 7729666 | 7729883 | 1 | multimodular transpeptidase-transglycosylase / penicillin-binding protein 1A/1B |
| 7729666 | 7764130 | 34464 | 7731013 | 7731214 | -1 | histidine kinase |
| 7729666 | 7764130 | 34464 | 7732230 | 7732361 | -1 | hypothetical protein |
| 7729666 | 7764130 | 34464 | 7732357 | 7732638 | -1 | hypothetical protein |
| 7729666 | 7764130 | 34464 | 7733739 | 7733954 | -1 | 23S rRNA pseudouridine synthase F |
| 7729666 | 7764130 | 34464 | 7734955 | 7735134 | -1 | D-tyrosyl-tRNA(Tyr) deacylase |
| 7729666 | 7764130 | 34464 | 7735152 | 7735331 | -1 | hypothetical protein |
| 7729666 | 7764130 | 34464 | 7736453 | 7736651 | 1 | transposase |
| 7729666 | 7764130 | 34464 | 7738911 | 7739075 | -1 | hypothetical protein |
| 7729666 | 7764130 | 34464 | 7741446 | 7741633 | 1 | hypothetical protein |
| 7729666 | 7764130 | 34464 | 7741696 | 7742283 | 1 | mob protein |
| 7729666 | 7764130 | 34464 | 7743284 | 7743651 | -1 | cell surface protein |
| 7729666 | 7764130 | 34464 | 7745855 | 7746239 | -1 | alpha/beta hydrolase |
| 7729666 | 7764130 | 34464 | 7747403 | 7747774 | 1 | conjugal transfer protein TraG |
| 7729666 | 7764130 | 34464 | 7748775 | 7748906 | -1 | ABC transporter ATP-binding protein |
| 7729666 | 7764130 | 34464 | 7748961 | 7749017 | -1 | stage II sporulation protein E |
| 7729666 | 7764130 | 34464 | 7750034 | 7750422 | -1 | chromosome partitioning protein ParB |
| 7729666 | 7764130 | 34464 | 7750423 | 7750633 | -1 | hypothetical protein |
| 7729666 | 7764130 | 34464 | 7751670 | 7752073 | 1 | serine dehydratase |
| 7729666 | 7764130 | 34464 | 7757163 | 7757453 | 1 | Wall associated protein |
| 7729666 | 7764130 | 34464 | 7760061 | 7760223 | 1 | spore protein |
| 7729666 | 7764130 | 34464 | 7761286 | 7761369 | -1 | SAM-dependent methyltransferase |
| 7729666 | 7764130 | 34464 | 7762477 | 7762704 | -1 | hypothetical protein |
| 7729666 | 7764130 | 34464 | 7763728 | 7764130 | 1 | IS4 family transposase |
| 7792172 | 7806391 | 14219 | 7792172 | 7792408 | -1 | hypothetical protein |
| 7792172 | 7806391 | 14219 | 7792413 | 7792592 | -1 | hypothetical protein |
| 7792172 | 7806391 | 14219 | 7792592 | 7792891 | -1 | hypothetical protein |
| 7792172 | 7806391 | 14219 | 7794101 | 7794322 | -1 | hypothetical protein |
| 7792172 | 7806391 | 14219 | 7794350 | 7794735 | -1 | ArpU family transcriptional regulator |
| 7792172 | 7806391 | 14219 | 7794992 | 7795378 | -1 | DUF1064 domain-containing protein |
| 7792172 | 7806391 | 14219 | 7795416 | 7795613 | -1 | hypothetical protein |
| 7792172 | 7806391 | 14219 | 7795624 | 7795801 | 1 | sodium:alanine symporter family protein |
| 7792172 | 7806391 | 14219 | 7796817 | 7797012 | -1 | hypothetical protein |
| 7792172 | 7806391 | 14219 | 7798027 | 7798690 | -1 | DNA topoisomerase I |
| 7792172 | 7806391 | 14219 | 7799811 | 7800188 | 1 | resolvase |
| 7792172 | 7806391 | 14219 | 7800309 | 7800548 | -1 | hypothetical protein |
| 7792172 | 7806391 | 14219 | 7800818 | 7800946 | 1 | SAM-dependent methyltransferase |
| 7792172 | 7806391 | 14219 | 7803467 | 7803659 | -1 | phosphoribosylformylglycinamidine synthase subunit PurS |
| 7792172 | 7806391 | 14219 | 7804734 | 7804901 | -1 | 23S rRNA pseudouridine synthase F |
| 7792172 | 7806391 | 14219 | 7806052 | 7806391 | 1 | hypothetical protein |
| 7842796 | 7916909 | 74113 | 7842796 | 7843063 | -1 | disulfide bond formation protein DsbA |
| 7842796 | 7916909 | 74113 | 7844064 | 7844252 | 1 | hypothetical protein |
| 7842796 | 7916909 | 74113 | 7845268 | 7845494 | -1 | hypothetical protein |
| 7842796 | 7916909 | 74113 | 7846522 | 7846742 | -1 | butanediol dehydrogenase |
| 7842796 | 7916909 | 74113 | 7847994 | 7848212 | 1 | hypothetical protein |
| 7842796 | 7916909 | 74113 | 7849329 | 7850257 | -1 | hypothetical protein |
| 7842796 | 7916909 | 74113 | 7850267 | 7851251 | -1 | RNA-binding protein |
| 7842796 | 7916909 | 74113 | 7851225 | 7851419 | 1 | hypothetical protein |
| 7842796 | 7916909 | 74113 | 7852894 | 7853156 | -1 | TrmB family transcriptional regulator |
| 7842796 | 7916909 | 74113 | 7854161 | 7854325 | 1 | hypothetical protein |
| 7842796 | 7916909 | 74113 | 7855586 | 7855747 | -1 | cobalt transporter |
| 7842796 | 7916909 | 74113 | 7856762 | 7856978 | -1 | 4-hydroxy-tetrahydrodipicolinate synthase |
| 7842796 | 7916909 | 74113 | 7856990 | 7857245 | -1 | aspartate kinase |
| 7842796 | 7916909 | 74113 | 7858280 | 7858503 | 1 | hypothetical protein |
| 7842796 | 7916909 | 74113 | 7858509 | 7858703 | -1 | hypothetical protein |
| 7842796 | 7916909 | 74113 | 7859976 | 7860318 | 1 | DUF445 domain-containing protein |
| 7842796 | 7916909 | 74113 | 7861319 | 7861556 | -1 | two-component sensor histidine kinase |
| 7842796 | 7916909 | 74113 | 7862558 | 7862801 | -1 | glycosyl transferase |
| 7842796 | 7916909 | 74113 | 7863894 | 7864065 | -1 | plasmid recombination enzyme |
| 7842796 | 7916909 | 74113 | 7864469 | 7864682 | -1 | hypothetical protein |
| 7842796 | 7916909 | 74113 | 7865699 | 7865979 | -1 | methyl-accepting chemotaxis protein |
| 7842796 | 7916909 | 74113 | 7869539 | 7869710 | -1 | transposase |
| 7842796 | 7916909 | 74113 | 7870711 | 7871769 | -1 | transposase |
| 7842796 | 7916909 | 74113 | 7871859 | 7871972 | -1 | aspartate phosphatase |
| 7842796 | 7916909 | 74113 | 7872132 | 7872527 | -1 | DUF4320 domain-containing protein |
| 7842796 | 7916909 | 74113 | 7873548 | 7873954 | -1 | hypothetical protein |
| 7842796 | 7916909 | 74113 | 7874080 | 7874409 | -1 | hypothetical protein |
| 7842796 | 7916909 | 74113 | 7875570 | 7875666 | 1 | AAA family ATPase |
| 7842796 | 7916909 | 74113 | 7876918 | 7877122 | -1 | zinc ribbon domain-containing protein |
| 7842796 | 7916909 | 74113 | 7879384 | 7880066 | -1 | DNA-binding protein |
| 7842796 | 7916909 | 74113 | 7880503 | 7880742 | -1 | hypothetical protein |
| 7842796 | 7916909 | 74113 | 7880745 | 7881293 | -1 | hypothetical protein |
| 7842796 | 7916909 | 74113 | 7881271 | 7881552 | -1 | hypothetical protein |
| 7842796 | 7916909 | 74113 | 7881545 | 7881751 | -1 | hypothetical protein |
| 7842796 | 7916909 | 74113 | 7881846 | 7882100 | -1 | hypothetical protein |
| 7842796 | 7916909 | 74113 | 7883268 | 7884135 | -1 | hypothetical protein |
| 7842796 | 7916909 | 74113 | 7884137 | 7885070 | -1 | hypothetical protein |
| 7842796 | 7916909 | 74113 | 7885063 | 7886492 | -1 | Type II secretion system protein |
| 7842796 | 7916909 | 74113 | 7886508 | 7886908 | -1 | chromosome partitioning protein ParA |
| 7842796 | 7916909 | 74113 | 7891275 | 7891826 | -1 | resolvase |
| 7842796 | 7916909 | 74113 | 7891991 | 7892209 | -1 | hypothetical protein |
| 7842796 | 7916909 | 74113 | 7892202 | 7893286 | -1 | spore gernimation protein |
| 7842796 | 7916909 | 74113 | 7893342 | 7893756 | -1 | spore gernimation protein |
| 7842796 | 7916909 | 74113 | 7896001 | 7896243 | -1 | gamma carbonic anhydrase family protein |
| 7842796 | 7916909 | 74113 | 7897273 | 7897461 | -1 | hypothetical protein |
| 7842796 | 7916909 | 74113 | 7898529 | 7898831 | -1 | AraC family transcriptional regulator |
| 7842796 | 7916909 | 74113 | 7899950 | 7900252 | -1 | hypothetical protein |
| 7842796 | 7916909 | 74113 | 7901551 | 7901657 | -1 | phosphohydrolase |
| 7842796 | 7916909 | 74113 | 7902788 | 7903091 | -1 | hypothetical protein |
| 7842796 | 7916909 | 74113 | 7904092 | 7904675 | -1 | IS4 family transposase |
| 7842796 | 7916909 | 74113 | 7905728 | 7906010 | -1 | hypothetical protein |
| 7842796 | 7916909 | 74113 | 7907025 | 7907131 | -1 | type I methionyl aminopeptidase |
| 7842796 | 7916909 | 74113 | 7907131 | 7907383 | -1 | adenylate kinase |
| 7842796 | 7916909 | 74113 | 7908417 | 7908869 | -1 | SMI1/KNR4 family protein |
| 7842796 | 7916909 | 74113 | 7908882 | 7909289 | -1 | type VII secretion protein |
| 7842796 | 7916909 | 74113 | 7910302 | 7911017 | -1 | chromosome partitioning protein ParA |
| 7842796 | 7916909 | 74113 | 7911034 | 7911895 | -1 | Flp pilus assembly protein CpaB |
| 7842796 | 7916909 | 74113 | 7912977 | 7913183 | -1 | glycoside hydrolase |
| 7842796 | 7916909 | 74113 | 7914201 | 7914403 | 1 | ATP-dependent 6-phosphofructokinase |
| 7842796 | 7916909 | 74113 | 7915421 | 7915685 | -1 | GTPase HflX |
| 7842796 | 7916909 | 74113 | 7916686 | 7916909 | -1 | adhesin |
