# Supplementary material for: Data on genome sequencing, analysis and annotation of a pathogenic Bacillus cereus 062011msu
Source: Data Brief. 2018 Jan 3;17:15–23. doi: 10.1016/j.dib.2017.12.054 (PMC5988026; doi:10.1016/j.dib.2017.12.054)
Supplement: Supplementary file 3 — Supplementary material [file mmc5.docx]

Table S2: RAST annotation summary of *Bacillus cereus* 062011msu

| **Contig_ID** | **Start** | **Stop** | **Strand** | **Gene Description** |
| --- | --- | --- | --- | --- |
| contig_1 | 195 | 728 | + | Glutamate synthase [NADPH] large chain (EC 1.4.1.13) |
| contig_1 | 799 | 3981 | + | Glutamate synthase [NADPH] large chain (EC 1.4.1.13) |
| contig_1 | 4200 | 4319 | + | Putative lipoprotein SAV1865 |
| contig_1 | 4279 | 4896 | + | Putative lipoprotein SAV1865 |
| contig_1 | 4916 | 5338 | + | Putative lipoprotein SAV1865 |
| contig_1 | 5504 | 5352 | - | Lipid A export ATP-binding/permease protein MsbA |
| contig_1 | 6171 | 5533 | - | Lipid A export ATP-binding/permease protein MsbA |
| contig_1 | 6328 | 6164 | - | Lipid A export ATP-binding/permease protein MsbA |
| contig_1 | 6551 | 6306 | - | Lipid A export ATP-binding/permease protein MsbA |
| contig_1 | 7117 | 6548 | - | Lipid A export ATP-binding/permease protein MsbA |
| contig_1 | 7702 | 7172 | - | Cysteinyl-tRNA synthetase related protein |
| contig_10 | 1275 | 397 | - | SSU rRNA (adenine(1518)-N(6)/adenine(1519)-N(6))-dimethyltransferase (EC 2.1.1.182) |
| contig_10 | 1830 | 1300 | - | Ribonuclease M5 (EC 3.1.26.8) |
| contig_100 | 192 | 10 | - | Amidohydrolase AmhX |
| contig_100 | 593 | 1939 | + | Na+/H+ antiporter NhaC |
| contig_100 | 2393 | 1974 | - | Transcriptional regulator domain protein |
| contig_100 | 2544 | 2368 | - | Transcriptional regulator domain protein |
| contig_100 | 3247 | 2522 | - | Transcriptional regulator domain protein |
| contig_100 | 3340 | 3579 | + | N-succinyl arginine/lysine racemase |
| contig_100 | 3576 | 4448 | + | N-succinyl arginine/lysine racemase |
| contig_100 | 4463 | 5158 | + | Protein clustered with N-succinyl arginine/lysine racemase |
| contig_1000 | 128 | 265 | + | hypothetical protein |
| contig_1000 | 289 | 651 | + | transcriptional regulator, ArsR family |
| contig_1001 | 132 | 269 | + | Tellurite resistance protein |
| contig_1001 | 421 | 561 | + | Tellurite resistance protein |
| contig_1001 | 726 | 851 | + | Tellurite resistance protein |
| contig_1001 | 848 | 967 | + | Tellurite resistance protein |
| contig_1001 | 992 | 1357 | + | FIG01225552: hypothetical protein |
| contig_1001 | 1380 | 1766 | + | FIG01225552: hypothetical protein |
| contig_1001 | 1717 | 1830 | + | FIG01225552: hypothetical protein |
| contig_1003 | 664 | 203 | - | putative PQQ enzyme repeat |
| contig_1003 | 914 | 630 | - | putative PQQ enzyme repeat |
| contig_1003 | 1126 | 992 | - | Transcriptional regulator, MarR family |
| contig_1003 | 1367 | 1203 | - | Transcriptional regulator, MarR family |
| contig_1004 | 73 | 630 | + | Phosphonate ABC transporter phosphate-binding periplasmic component (TC 3.A.1.9.1) |
| contig_1004 | 587 | 1081 | + | Phosphonate ABC transporter phosphate-binding periplasmic component (TC 3.A.1.9.1) |
| contig_1005 | 43 | 267 | + | Predicted N-ribosylNicotinamide CRP-like regulator |
| contig_1005 | 975 | 691 | - | Holin associated hypothetical protein |
| contig_1005 | 1139 | 1020 | - | Holin associated hypothetical protein |
| contig_1005 | 1394 | 1146 | - | Holin associated membrane protein 1 |
| contig_1005 | 1699 | 1490 | - | Transcriptional regulator, GntR family domain / Aspartate aminotransferase (EC 2.6.1.1) |
| contig_1005 | 2486 | 1659 | - | Transcriptional regulator, GntR family domain / Aspartate aminotransferase (EC 2.6.1.1) |
| contig_1005 | 2925 | 2563 | - | Transcriptional regulator, GntR family domain / Aspartate aminotransferase (EC 2.6.1.1) |
| contig_1005 | 2910 | 3185 | + | hypothetical protein |
| contig_1006 | 826 | 338 | - | CAAX amino terminal protease family protein |
| contig_1006 | 935 | 810 | - | hypothetical protein |
| contig_1006 | 1813 | 1010 | - | Nucleotidyltransferase (EC 2.7.7.-) |
| contig_1006 | 2237 | 1866 | - | YuzD-like protein |
| contig_1007 | 188 | 1024 | + | ABC-type Fe3+-siderophore transport system, permease component |
| contig_1007 | 1032 | 1193 | + | ABC-type Fe3+-siderophore transport system, permease component |
| contig_1007 | 1190 | 1567 | + | ABC-type Fe3+-siderophore transport system, permease 2 component |
| contig_1007 | 1567 | 2250 | + | ABC-type Fe3+-siderophore transport system, permease 2 component |
| contig_1008 | 186 | 446 | + | D-alanyl-D-alanine carboxypeptidase (EC 3.4.16.4) |
| contig_1009 | 1851 | 526 | - | Hypoxanthine/guanine permease PbuG |
| contig_101 | 600 | 406 | - | FIG01225877: hypothetical protein |
| contig_101 | 800 | 558 | - | FIG01225877: hypothetical protein |
| contig_101 | 1055 | 1654 | + | Lipoteichoic acid synthase LtaS Type IIIa |
| contig_101 | 1797 | 2105 | + | Lipoteichoic acid synthase LtaS Type IIIa |
| contig_101 | 2110 | 2943 | + | Lipoteichoic acid synthase LtaS Type IIIa |
| contig_101 | 3125 | 3826 | + | CDP-diacylglycerol--serine O-phosphatidyltransferase (EC 2.7.8.8) |
| contig_101 | 3978 | 3859 | - | Glyoxylate reductase (EC 1.1.1.79) / Glyoxylate reductase (EC 1.1.1.26) / Hydroxypyruvate reductase (EC 1.1.1.81) |
| contig_101 | 4189 | 3956 | - | Glyoxylate reductase (EC 1.1.1.79) / Glyoxylate reductase (EC 1.1.1.26) / Hydroxypyruvate reductase (EC 1.1.1.81) |
| contig_101 | 4639 | 4220 | - | Glyoxylate reductase (EC 1.1.1.79) / Glyoxylate reductase (EC 1.1.1.26) / Hydroxypyruvate reductase (EC 1.1.1.81) |
| contig_101 | 4886 | 4731 | - | Glyoxylate reductase (EC 1.1.1.79) / Glyoxylate reductase (EC 1.1.1.26) / Hydroxypyruvate reductase (EC 1.1.1.81) |
| contig_101 | 5152 | 4985 | - | FIG01227696: hypothetical protein |
| contig_101 | 6006 | 5221 | - | Histidinol-phosphatase (EC 3.1.3.15) |
| contig_101 | 6579 | 6433 | - | Phosphoribosyl-ATP pyrophosphatase (EC 3.6.1.31) |
| contig_101 | 6895 | 6590 | - | Phosphoribosyl-AMP cyclohydrolase (EC 3.5.4.19) |
| contig_101 | 7617 | 6892 | - | Imidazole glycerol phosphate synthase cyclase subunit (EC 4.1.3.-) |
| contig_101 | 8364 | 7645 | - | Phosphoribosylformimino-5-aminoimidazole carboxamide ribotide isomerase (EC 5.3.1.16) |
| contig_101 | 8968 | 8405 | - | Imidazole glycerol phosphate synthase amidotransferase subunit (EC 2.4.2.-) |
| contig_101 | 9403 | 8969 | - | Imidazoleglycerol-phosphate dehydratase (EC 4.2.1.19) |
| contig_101 | 9552 | 9433 | - | Imidazoleglycerol-phosphate dehydratase (EC 4.2.1.19) |
| contig_101 | 9842 | 9552 | - | Histidinol dehydrogenase (EC 1.1.1.23) |
| contig_101 | 10156 | 9941 | - | Histidinol dehydrogenase (EC 1.1.1.23) |
| contig_101 | 10536 | 10132 | - | Histidinol dehydrogenase (EC 1.1.1.23) |
| contig_1010 | 146 | 12 | - | Hemoglobin-like protein HbO |
| contig_1010 | 410 | 294 | - | Hemoglobin-like protein HbO |
| contig_1010 | 1150 | 566 | - | Adenylate cyclase |
| contig_1010 | 1297 | 1668 | + | FIG01228403: hypothetical protein |
| contig_1010 | 1699 | 2337 | + | GTP pyrophosphokinase (EC 2.7.6.5) |
| contig_1010 | 2356 | 2571 | + | NAD kinase (EC 2.7.1.23) |
| contig_1010 | 2529 | 3152 | + | NAD kinase (EC 2.7.1.23) |
| contig_1010 | 3219 | 3386 | + | Similar to ribosomal large subunit pseudouridine synthase D, Bacillus subtilis YjbO type |
| contig_1010 | 3355 | 4062 | + | Similar to ribosomal large subunit pseudouridine synthase D, Bacillus subtilis YjbO type |
| contig_1011 | 621 | 259 | - | Dihydroneopterin aldolase (EC 4.1.2.25) |
| contig_1011 | 1407 | 622 | - | Dihydropteroate synthase (EC 2.5.1.15) |
| contig_1011 | 1683 | 1480 | - | Aminodeoxychorismate lyase (EC 4.1.3.38) |
| contig_1012 | 266 | 51 | - | FIG01225227: hypothetical protein |
| contig_1013 | 86 | 316 | + | Cell division protein FtsI [Peptidoglycan synthetase] (EC 2.4.1.129) / Transpeptidase, Penicillin binding protein transpeptidase domain |
| contig_1013 | 639 | 1103 | + | Cell division protein FtsI [Peptidoglycan synthetase] (EC 2.4.1.129) / Transpeptidase, Penicillin binding protein transpeptidase domain |
| contig_1013 | 1186 | 1308 | + | Cell division protein FtsI [Peptidoglycan synthetase] (EC 2.4.1.129) / Transpeptidase, Penicillin binding protein transpeptidase domain |
| contig_1013 | 1379 | 1498 | + | hypothetical protein |
| contig_1013 | 1933 | 2070 | + | Beta-lactamase class A |
| contig_1013 | 2213 | 2761 | + | Beta-lactamase class A |
| contig_1014 | 1408 | 725 | - | FIG01225226: hypothetical protein |
| contig_1015 | 24 | 644 | + | ABC transporter, ATP-binding protein |
| contig_1015 | 717 | 875 | + | ABC transporter, ATP-binding protein |
| contig_1015 | 862 | 1743 | + | ABC transporter, ATP-binding protein |
| contig_1015 | 1759 | 2550 | + | ABC transporter, permease |
| contig_1016 | 262 | 56 | - | Quaternary ammonium compound-resistance protein SugE |
| contig_1017 | 240 | 1 | - | Flagellum-specific ATP synthase FliI |
| contig_1017 | 625 | 299 | - | Flagellum-specific ATP synthase FliI |
| contig_1017 | 1106 | 738 | - | Flagellum-specific ATP synthase FliI |
| contig_1017 | 1308 | 1120 | - | Flagellum-specific ATP synthase FliI |
| contig_1017 | 1466 | 1305 | - | Flagellar assembly protein FliH |
| contig_1017 | 1993 | 1544 | - | Flagellar assembly protein FliH |
| contig_1017 | 2984 | 1980 | - | Flagellar motor switch protein FliG |
| contig_1017 | 3453 | 2998 | - | Flagellar M-ring protein FliF |
| contig_1017 | 4222 | 3482 | - | Flagellar M-ring protein FliF |
| contig_1017 | 4460 | 4236 | - | Flagellar M-ring protein FliF |
| contig_1018 | 1789 | 98 | - | sensor histidine kinase/response regulator |
| contig_1019 | 448 | 89 | - | putative cytochrome P450 hydroxylase |
| contig_1019 | 661 | 530 | - | putative cytochrome P450 hydroxylase |
| contig_102 | 307 | 522 | + | membrane protein, MmpL family |
| contig_102 | 538 | 741 | + | membrane protein, MmpL family |
| contig_102 | 772 | 1506 | + | membrane protein, MmpL family |
| contig_102 | 1523 | 2320 | + | membrane protein, MmpL family |
| contig_1020 | 227 | 15 | - | membrane protein, putative |
| contig_1020 | 435 | 193 | - | membrane protein, putative |
| contig_1020 | 814 | 437 | - | Magnesium and cobalt efflux protein CorC |
| contig_1020 | 936 | 769 | - | hypothetical protein |
| contig_1020 | 1509 | 979 | - | Magnesium and cobalt efflux protein CorC |
| contig_1021 | 1050 | 589 | - | Ribonucleotide reductase transcriptional regulator NrdR |
| contig_1022 | 488 | 78 | - | FIG01226746: hypothetical protein |
| contig_1022 | 688 | 503 | - | Lactoylglutathione lyase (EC 4.4.1.5) |
| contig_1022 | 866 | 654 | - | Lactoylglutathione lyase (EC 4.4.1.5) |
| contig_1022 | 1016 | 1180 | + | Polypeptide composition of the spore coat; required for the assembly of CotJC |
| contig_1023 | 152 | 697 | + | internalin, putative |
| contig_1023 | 2830 | 2219 | - | FIG01226365: hypothetical protein |
| contig_1024 | 292 | 107 | - | Lipoteichoic acid synthase LtaS Type Ia |
| contig_1025 | 563 | 204 | - | enterotoxin / cell-wall binding protein |
| contig_1026 | 114 | 1106 | + | UDP-glucose 4-epimerase (EC 5.1.3.2) |
| contig_1026 | 1226 | 1885 | + | FIGfam009438: Two-component system DNA-binding response regulator |
| contig_1027 | 322 | 80 | - | Penicillin-binding protein 2B |
| contig_1027 | 639 | 373 | - | Penicillin-binding protein 2B |
| contig_1028 | 179 | 1060 | + | Pantothenate:Na+ symporter (TC 2.A.21.1.1) |
| contig_1028 | 1087 | 1545 | + | Pantothenate:Na+ symporter (TC 2.A.21.1.1) |
| contig_1028 | 1573 | 1986 | + | Inosine-uridine preferring nucleoside hydrolase (EC 3.2.2.1) |
| contig_1028 | 2190 | 2525 | + | Inosine-uridine preferring nucleoside hydrolase (EC 3.2.2.1) |
| contig_1029 | 418 | 119 | - | FIG007042: hypothetical protein |
| contig_1029 | 1208 | 495 | - | 3-oxoacyl-[acyl-carrier protein] reductase paralog (EC 1.1.1.100) in cluster with unspecified monosaccharide transporter |
| contig_103 | 40 | 231 | + | hypothetical protein |
| contig_103 | 322 | 615 | + | FIG01226365: hypothetical protein |
| contig_103 | 1408 | 1536 | + | hypothetical protein |
| contig_103 | 1868 | 2047 | + | FIG01249777: hypothetical protein |
| contig_103 | 2323 | 2207 | - | internalin, putative |
| contig_103 | 4347 | 2446 | - | internalin, putative |
| contig_1030 | 966 | 271 | - | FIG009210: peptidase, M16 family |
| contig_1031 | 158 | 427 | + | 1,4-dihydroxy-2-naphthoate polyprenyltransferase (EC 2.5.1.74) |
| contig_1031 | 1066 | 512 | - | Streptothricin acetyltransferase, Streptomyces lavendulae type |
| contig_1032 | 284 | 27 | - | Putative deoxyribonuclease similar to YcfH, type 3 |
| contig_1032 | 826 | 347 | - | Putative deoxyribonuclease similar to YcfH, type 3 |
| contig_1032 | 996 | 883 | - | hypothetical protein |
| contig_1032 | 1692 | 1570 | - | FIG01226481: hypothetical protein |
| contig_1032 | 1927 | 1697 | - | FIG01226481: hypothetical protein |
| contig_1033 | 64 | 579 | + | Uncharacterized protein Bsub YpbR |
| contig_1033 | 735 | 583 | - | FIG01225157: hypothetical protein |
| contig_1034 | 270 | 7 | - | FIG01225352: hypothetical protein |
| contig_1034 | 609 | 325 | - | FIG01225352: hypothetical protein |
| contig_1034 | 1292 | 903 | - | FIG01228982: hypothetical protein |
| contig_1034 | 1589 | 1410 | - | FIG01228982: hypothetical protein |
| contig_1035 | 350 | 547 | + | Sporulation kinase B (EC 2.7.13.3) |
| contig_1035 | 756 | 1526 | + | Sporulation kinase B (EC 2.7.13.3) |
| contig_1036 | 40 | 228 | + | FIG01227778: hypothetical protein |
| contig_1037 | 935 | 774 | - | tRNA nucleotidyltransferase (EC 2.7.7.21) (EC 2.7.7.25) |
| contig_1037 | 1041 | 916 | - | tRNA nucleotidyltransferase (EC 2.7.7.21) (EC 2.7.7.25) |
| contig_1038 | 819 | 67 | - | Oligoendopeptidase F (EC 3.4.24.-) |
| contig_1038 | 1574 | 1065 | - | Oligoendopeptidase F (EC 3.4.24.-) |
| contig_1038 | 1786 | 1610 | - | Oligoendopeptidase F (EC 3.4.24.-) |
| contig_1038 | 2470 | 1904 | - | N-hydroxyarylamine O-acetyltransferase (EC 2.3.1.118) |
| contig_1038 | 2612 | 2484 | - | N-hydroxyarylamine O-acetyltransferase (EC 2.3.1.118) |
| contig_1038 | 3161 | 3048 | - | hypothetical protein |
| contig_1038 | 3236 | 3454 | + | Clostridial MutS2-related protein |
| contig_1038 | 3423 | 3545 | + | Clostridial MutS2-related protein |
| contig_1038 | 3628 | 3876 | + | Clostridial MutS2-related protein |
| contig_1038 | 3950 | 4615 | + | Clostridial MutS2-related protein |
| contig_1038 | 4566 | 4874 | + | Clostridial MutS2-related protein |
| contig_1038 | 5163 | 5285 | + | acetyltransferase, GNAT family |
| contig_1038 | 5248 | 5688 | + | acetyltransferase, GNAT family |
| contig_1038 | 5850 | 6374 | + | Phosphoglycerate mutase family 1 |
| contig_1039 | 54 | 191 | + | conserved membrane-spanning protein |
| contig_1039 | 417 | 608 | + | Phosphonate ABC transporter phosphate-binding periplasmic component (TC 3.A.1.9.1) |
| contig_1039 | 580 | 1020 | + | Phosphonate ABC transporter phosphate-binding periplasmic component (TC 3.A.1.9.1) |
| contig_1039 | 986 | 1183 | + | Phosphonate ABC transporter phosphate-binding periplasmic component (TC 3.A.1.9.1) |
| contig_1039 | 1662 | 1330 | - | Phosphonate ABC transporter phosphate-binding periplasmic component (TC 3.A.1.9.1) |
| contig_104 | 246 | 737 | + | Dimeric dUTPase (EC 3.6.1.23) |
| contig_104 | 807 | 1493 | + | Peptidase, M42 family |
| contig_104 | 1573 | 1860 | + | Peptidase, M42 family |
| contig_104 | 2213 | 1935 | - | TetR family regulatory protein of MDR cluster |
| contig_104 | 2553 | 2218 | - | TetR family regulatory protein of MDR cluster |
| contig_104 | 2851 | 3387 | + | Multidrug resistance protein [function not yet clear] |
| contig_104 | 3371 | 3505 | + | Multidrug resistance protein [function not yet clear] |
| contig_104 | 3518 | 3637 | + | Membrane component of multidrug resistance system |
| contig_1040 | 27 | 146 | + | hypothetical protein |
| contig_1040 | 579 | 992 | + | Uncharacterized protein Bsub YpbR |
| contig_1041 | 13 | 126 | + | hypothetical protein |
| contig_1041 | 699 | 136 | - | internalin, putative |
| contig_1041 | 1660 | 983 | - | internalin, putative |
| contig_1041 | 1961 | 1812 | - | internalin, putative |
| contig_1042 | 61 | 909 | + | Stage V sporulation protein AD (SpoVAD) |
| contig_1042 | 906 | 1256 | + | Stage V sporulation protein AE (SpoVAE) |
| contig_1043 | 171 | 851 | + | Stage V sporulation protein AD (SpoVAD) |
| contig_1043 | 848 | 1198 | + | Stage V sporulation protein AE (SpoVAE) |
| contig_1044 | 516 | 875 | + | Beta-galactosidase (EC 3.2.1.23) |
| contig_1045 | 897 | 85 | - | Cell division protein FtsI [Peptidoglycan synthetase] (EC 2.4.1.129) / Transpeptidase, Penicillin binding protein transpeptidase domain |
| contig_1045 | 1527 | 1090 | - | Cell division protein FtsI [Peptidoglycan synthetase] (EC 2.4.1.129) / Transpeptidase, Penicillin binding protein transpeptidase domain |
| contig_1046 | 791 | 567 | - | Aspartate racemase (EC 5.1.1.13) |
| contig_1046 | 1146 | 826 | - | Aspartate racemase (EC 5.1.1.13) |
| contig_1046 | 1250 | 1128 | - | hypothetical protein |
| contig_1047 | 544 | 110 | - | Transcriptional repressor of the fructose operon, DeoR family |
| contig_1047 | 875 | 549 | - | Transcriptional repressor of the fructose operon, DeoR family |
| contig_1049 | 130 | 261 | + | Urea channel UreI |
| contig_1049 | 463 | 576 | + | Urea channel UreI |
| contig_1049 | 576 | 737 | + | HoxN/HupN/NixA family nickel/cobalt transporter |
| contig_1049 | 727 | 948 | + | HoxN/HupN/NixA family nickel/cobalt transporter |
| contig_1049 | 1100 | 1564 | + | HoxN/HupN/NixA family nickel/cobalt transporter |
| contig_1049 | 2836 | 2024 | - | N-acetylglutamate synthase (EC 2.3.1.1) |
| contig_1049 | 2989 | 3192 | + | FIG01226210: hypothetical protein |
| contig_1049 | 3577 | 3452 | - | hypothetical protein |
| contig_1049 | 3651 | 4358 | + | Transcriptional regulator, XRE family |
| contig_105 | 226 | 348 | + | Para-aminobenzoate synthase, aminase component (EC 2.6.1.85) |
| contig_105 | 368 | 1669 | + | Para-aminobenzoate synthase, aminase component (EC 2.6.1.85) |
| contig_105 | 1675 | 2262 | + | Para-aminobenzoate synthase, amidotransferase component (EC 2.6.1.85) @ Anthranilate synthase, amidotransferase component (EC 4.1.3.27) # PabAb@TrpAb |
| contig_105 | 2256 | 2771 | + | Aminodeoxychorismate lyase (EC 4.1.3.38) |
| contig_1050 | 1366 | 557 | - | UTP--glucose-1-phosphate uridylyltransferase (EC 2.7.7.9) |
| contig_1051 | 799 | 263 | - | Rrf2-linked NADH-flavin reductase |
| contig_1051 | 1001 | 1315 | + | FIG01225208: hypothetical protein |
| contig_1051 | 1443 | 1712 | + | FIG01225573: hypothetical protein |
| contig_1051 | 3673 | 1736 | - | Membrane component of multidrug resistance system |
| contig_1051 | 4512 | 3826 | - | Cytochrome c oxidase polypeptide I (EC 1.9.3.1) |
| contig_1051 | 5448 | 4546 | - | Cytochrome c oxidase polypeptide II (EC 1.9.3.1) |
| contig_1051 | 6037 | 5828 | - | Heme O synthase, protoheme IX farnesyltransferase (EC 2.5.1.-) COX10-CtaB |
| contig_1051 | 6618 | 6040 | - | Heme O synthase, protoheme IX farnesyltransferase (EC 2.5.1.-) COX10-CtaB |
| contig_1052 | 162 | 16 | - | Flagellar biosynthesis protein FliR |
| contig_1052 | 305 | 192 | - | Flagellar biosynthesis protein FliQ |
| contig_1052 | 469 | 272 | - | Flagellar biosynthesis protein FliQ |
| contig_1052 | 1144 | 503 | - | Flagellar biosynthesis protein FliP |
| contig_1052 | 1266 | 1153 | - | Flagellar biosynthesis protein FliP |
| contig_1052 | 1588 | 1475 | - | Flagellar motor switch protein FliN |
| contig_1052 | 1792 | 1601 | - | Flagellar motor switch protein FliN |
| contig_1053 | 664 | 158 | - | FIG007959: peptidase, M16 family |
| contig_1053 | 848 | 675 | - | FIG007959: peptidase, M16 family |
| contig_1053 | 1017 | 823 | - | FIG007959: peptidase, M16 family |
| contig_1053 | 1402 | 1028 | - | FIG007959: peptidase, M16 family |
| contig_1053 | 1948 | 1475 | - | FIG007013: polysaccharide deacetylase, putative |
| contig_1053 | 2396 | 1980 | - | FIG007013: polysaccharide deacetylase, putative |
| contig_1053 | 2523 | 2410 | - | hypothetical protein |
| contig_1055 | 103 | 1698 | + | pXO1-55 |
| contig_1055 | 1673 | 1903 | + | pXO1-55 |
| contig_1055 | 1970 | 2197 | + | FIG01227692: hypothetical protein |
| contig_1055 | 2701 | 2435 | - | Mobile element protein |
| contig_1055 | 3185 | 2769 | - | Mobile element protein |
| contig_1055 | 3442 | 3275 | - | Mobile element protein |
| contig_1055 | 3795 | 3478 | - | Mobile element protein |
| contig_1056 | 75 | 329 | + | RNA polymerase sigma-70 factor, ECF subfamily |
| contig_1056 | 369 | 623 | + | RNA polymerase sigma-70 factor, ECF subfamily |
| contig_1056 | 604 | 849 | + | FIG01230956: hypothetical protein |
| contig_1056 | 1225 | 1662 | + | Tyrosyl-tRNA synthetase (EC 6.1.1.1) |
| contig_1056 | 1668 | 2165 | + | Tyrosyl-tRNA synthetase (EC 6.1.1.1) |
| contig_1057 | 126 | 1 | - | FIG00732228: membrane protein |
| contig_1057 | 640 | 104 | - | FIG00732228: membrane protein |
| contig_1057 | 1244 | 675 | - | FIG00732228: membrane protein |
| contig_1057 | 1542 | 1336 | - | FIG01225319: hypothetical protein |
| contig_1058 | 969 | 166 | - | Macrolide efflux protein (EC 2.7.7.2) |
| contig_1058 | 1737 | 1402 | - | Azoreductase |
| contig_1058 | 1884 | 1768 | - | Azoreductase |
| contig_1058 | 2311 | 2144 | - | FIG01226109: hypothetical protein |
| contig_1059 | 15 | 134 | + | Transporter, LysE family |
| contig_1059 | 336 | 518 | + | Transporter, LysE family |
| contig_1059 | 538 | 831 | + | FIG01226580: hypothetical protein |
| contig_106 | 163 | 501 | + | Nudix hydrolase family protein |
| contig_106 | 498 | 815 | + | FIG01225718: hypothetical protein |
| contig_1060 | 58 | 561 | + | Lysophospholipase (EC 3.1.1.5); Monoglyceride lipase (EC 3.1.1.23); putative |
| contig_1060 | 542 | 904 | + | Lysophospholipase (EC 3.1.1.5); Monoglyceride lipase (EC 3.1.1.23); putative |
| contig_1060 | 973 | 857 | - | hypothetical protein |
| contig_1060 | 972 | 1145 | + | hypothetical protein |
| contig_1060 | 1267 | 1938 | + | hypothetical protein |
| contig_1060 | 2070 | 2252 | + | FIG01227295: hypothetical protein |
| contig_1061 | 99 | 1355 | + | 2',3'-cyclic-nucleotide 2'-phosphodiesterase (EC 3.1.4.16) |
| contig_1062 | 765 | 34 | - | Membrane protein involved in the export of O-antigen, teichoic acid lipoteichoic acids |
| contig_1063 | 5 | 394 | + | Cytochrome d ubiquinol oxidase subunit II (EC 1.10.3.-) |
| contig_1063 | 360 | 614 | + | Cytochrome d ubiquinol oxidase subunit II (EC 1.10.3.-) |
| contig_1063 | 695 | 898 | + | Trk system potassium uptake protein TrkA |
| contig_1063 | 1054 | 1329 | + | Trk system potassium uptake protein TrkA |
| contig_1064 | 812 | 315 | - | stage V sporulation protein K |
| contig_1064 | 1461 | 1006 | - | stage V sporulation protein K |
| contig_1065 | 219 | 103 | - | L-Proline/Glycine betaine transporter ProP |
| contig_1065 | 572 | 339 | - | FIG01227160: hypothetical protein |
| contig_1065 | 1019 | 633 | - | 3-Oxoadipate enol-lactonase, alpha/beta hydrolase fold family [EC:3.1.1.24] |
| contig_1065 | 1155 | 1033 | - | 3-Oxoadipate enol-lactonase, alpha/beta hydrolase fold family [EC:3.1.1.24] |
| contig_1067 | 1135 | 44 | - | acetyl-CoA hydrolase/transferase family protein |
| contig_1067 | 1560 | 1132 | - | acetyl-CoA hydrolase/transferase family protein |
| contig_1068 | 261 | 527 | + | transcriptional regulator, ArsR family |
| contig_1068 | 1096 | 773 | - | Transcriptional regulator, HxlR family |
| contig_1069 | 39 | 224 | + | Lysophospholipase (EC 3.1.1.5); Monoglyceride lipase (EC 3.1.1.23); putative |
| contig_107 | 478 | 62 | - | N-Acetyl-D-glucosamine ABC transport system, sugar-binding protein |
| contig_107 | 977 | 1552 | + | fis-type helix-turn-helix domain protein |
| contig_107 | 1575 | 1841 | + | fis-type helix-turn-helix domain protein |
| contig_1071 | 68 | 340 | + | pyridine nucleotide-disulphide oxidoreductase family protein |
| contig_1071 | 434 | 670 | + | Sarcosine oxidase alpha subunit (EC 1.5.3.1) |
| contig_1071 | 667 | 1665 | + | Sarcosine oxidase alpha subunit (EC 1.5.3.1) |
| contig_1072 | 426 | 16 | - | pfs protein, putative |
| contig_1072 | 688 | 476 | - | pfs protein, putative |
| contig_1072 | 2072 | 1332 | - | UPF0020, Putative RNA methylase family UPF0020 |
| contig_1072 | 2286 | 2050 | - | UPF0020, Putative RNA methylase family UPF0020 |
| contig_1072 | 2532 | 2419 | - | S-layer protein / N-acetylmuramoyl-L-alanine amidase (EC 3.5.1.28) |
| contig_1072 | 3312 | 2590 | - | S-layer protein / N-acetylmuramoyl-L-alanine amidase (EC 3.5.1.28) |
| contig_1072 | 3706 | 3332 | - | S-layer protein / N-acetylmuramoyl-L-alanine amidase (EC 3.5.1.28) |
| contig_1072 | 4579 | 4253 | - | Deblocking aminopeptidase (EC 3.4.11.-) |
| contig_1073 | 333 | 446 | + | 5'-methylthioadenosine/S-adenosylhomocysteine nucleosidase related protein BA2564 |
| contig_1074 | 219 | 791 | + | HigA protein (antitoxin to HigB) |
| contig_1074 | 1046 | 861 | - | Stress-responsive transcriptional regulator PspC |
| contig_1074 | 1228 | 1545 | + | Cytosine deaminase (EC 3.5.4.1) |
| contig_1074 | 1572 | 1874 | + | Cytosine deaminase (EC 3.5.4.1) |
| contig_1074 | 1927 | 2469 | + | Cytosine deaminase (EC 3.5.4.1) |
| contig_1075 | 664 | 74 | - | Tyrosine recombinase XerC |
| contig_1075 | 1471 | 1593 | + | hypothetical protein |
| contig_1075 | 1984 | 2463 | + | Sucrose operon repressor ScrR, LacI family |
| contig_1076 | 319 | 11 | - | FIG01227313: hypothetical protein |
| contig_1077 | 16 | 165 | + | DUF378 domain-containing protein |
| contig_1077 | 745 | 311 | - | SSU ribosomal protein S1p |
| contig_1077 | 1764 | 892 | - | Aspartate aminotransferase (EC 2.6.1.1) |
| contig_1077 | 2081 | 1773 | - | Aspartate aminotransferase (EC 2.6.1.1) |
| contig_1077 | 2575 | 2078 | - | Transcriptional regulator, AsnC family |
| contig_1078 | 392 | 276 | - | Exosporium protein A |
| contig_1078 | 564 | 343 | - | Exosporium protein A |
| contig_1079 | 391 | 101 | - | ABC transporter, permease |
| contig_1079 | 617 | 384 | - | ABC transporter, ATP-binding protein |
| contig_1079 | 921 | 652 | - | ABC transporter, ATP-binding protein |
| contig_1079 | 1228 | 947 | - | ABC transporter, ATP-binding protein |
| contig_108 | 252 | 1 | - | DNA-binding response regulator |
| contig_1081 | 224 | 1096 | + | Isoflavone_redu, Isoflavone reductase |
| contig_1081 | 1811 | 1185 | - | Manganese superoxide dismutase (EC 1.15.1.1) |
| contig_1082 | 843 | 664 | - | Microsomal dipeptidase (EC 3.4.13.19) |
| contig_1082 | 1375 | 803 | - | Microsomal dipeptidase (EC 3.4.13.19) |
| contig_1082 | 1590 | 1375 | - | Microsomal dipeptidase (EC 3.4.13.19) |
| contig_1082 | 1909 | 1649 | - | Stage V sporulation protein required for dehydratation of the spore core and assembly of the coat (SpoVS) |
| contig_1082 | 2406 | 2059 | - | FIG006542: Phosphoesterase |
| contig_1084 | 1130 | 312 | - | hypothetical protein |
| contig_1084 | 1287 | 1147 | - | hypothetical protein |
| contig_1084 | 1562 | 1284 | - | hypothetical protein |
| contig_1085 | 78 | 869 | + | Ku domain protein |
| contig_1086 | 239 | 553 | + | Cytidylate kinase (EC 2.7.4.25) |
| contig_1087 | 364 | 95 | - | alcohol dehydrogenase, iron-containing |
| contig_1087 | 773 | 369 | - | alcohol dehydrogenase, iron-containing |
| contig_1087 | 977 | 780 | - | Alcohol dehydrogenase (EC 1.1.1.1) |
| contig_1087 | 1249 | 950 | - | alcohol dehydrogenase, iron-containing |
| contig_1088 | 470 | 336 | - | Multimodular transpeptidase-transglycosylase (EC 2.4.1.129) (EC 3.4.-.-) |
| contig_1088 | 678 | 436 | - | Multimodular transpeptidase-transglycosylase (EC 2.4.1.129) (EC 3.4.-.-) |
| contig_1088 | 1127 | 690 | - | Multimodular transpeptidase-transglycosylase (EC 2.4.1.129) (EC 3.4.-.-) |
| contig_1088 | 1464 | 1183 | - | Multimodular transpeptidase-transglycosylase (EC 2.4.1.129) (EC 3.4.-.-) |
| contig_1088 | 1639 | 1475 | - | Multimodular transpeptidase-transglycosylase (EC 2.4.1.129) (EC 3.4.-.-) |
| contig_1091 | 966 | 427 | - | membrane protein, putative |
| contig_1091 | 1384 | 1118 | - | Glucose dehydrogenase [pyrroloquinoline-quinone] |
| contig_1091 | 1664 | 1362 | - | Glucose dehydrogenase [pyrroloquinoline-quinone] |
| contig_1092 | 193 | 53 | - | Glycine betaine ABC transport system, ATP-binding protein OpuAA (EC 3.6.3.32) |
| contig_1092 | 550 | 335 | - | Integral membrane protein |
| contig_1092 | 668 | 504 | - | Integral membrane protein |
| contig_1093 | 1097 | 195 | - | Cobalt-zinc-cadmium resistance protein CzcD |
| contig_1096 | 361 | 131 | - | Probable membrane protein YetF |
| contig_1098 | 13 | 1824 | + | Phage tail fiber protein |
| contig_1098 | 2727 | 2521 | - | Glutamate synthase [NADPH] large chain (EC 1.4.1.13) |
| contig_1098 | 3056 | 2724 | - | Glutamate synthase [NADPH] large chain (EC 1.4.1.13) |
| contig_1099 | 220 | 107 | - | COG1649 predicted glycoside hydrolase |
| contig_1099 | 751 | 230 | - | COG1649 predicted glycoside hydrolase |
| contig_1099 | 1023 | 1211 | + | PTS system, diacetylchitobiose-specific IIB component (EC 2.7.1.69) |
| contig_1099 | 1208 | 1330 | + | PTS system, diacetylchitobiose-specific IIB component (EC 2.7.1.69) |
| contig_1099 | 1622 | 1936 | + | PTS system, diacetylchitobiose-specific IIC component (EC 2.7.1.69) |
| contig_1099 | 2060 | 2629 | + | PTS system, diacetylchitobiose-specific IIC component (EC 2.7.1.69) |
| contig_1099 | 2709 | 2885 | + | FIG01225791: hypothetical protein |
| contig_1099 | 2968 | 3096 | + | Anhydro-N-acetylmuramic acid kinase (EC 2.7.1.-) |
| contig_1099 | 3267 | 3752 | + | Anhydro-N-acetylmuramic acid kinase (EC 2.7.1.-) |
| contig_1099 | 3797 | 4078 | + | Anhydro-N-acetylmuramic acid kinase (EC 2.7.1.-) |
| contig_1099 | 4089 | 4235 | + | Anhydro-N-acetylmuramic acid kinase (EC 2.7.1.-) |
| contig_11 | 314 | 559 | + | Molybdenum ABC transporter, periplasmic molybdenum-binding protein ModA (TC 3.A.1.8.1) |
| contig_11 | 761 | 543 | - | FIG01233829: hypothetical protein |
| contig_11 | 826 | 1239 | + | Molybdenum transport system permease protein ModB (TC 3.A.1.8.1) |
| contig_11 | 1675 | 2394 | + | transporter, putative |
| contig_11 | 2962 | 2462 | - | Ribosyl nicotinamide transporter, PnuC-like |
| contig_11 | 3113 | 2967 | - | Ribosyl nicotinamide transporter, PnuC-like |
| contig_11 | 3344 | 4105 | + | Pyrroline-5-carboxylate reductase (EC 1.5.1.2) |
| contig_11 | 4107 | 4280 | + | oxidoreductase of aldo/keto reductase family, subgroup 1 |
| contig_11 | 4528 | 4941 | + | oxidoreductase of aldo/keto reductase family, subgroup 1 |
| contig_11 | 5333 | 6385 | + | Oligopeptide ABC transporter, periplasmic oligopeptide-binding protein OppA (TC 3.A.1.5.1) |
| contig_11 | 6348 | 6542 | + | Oligopeptide ABC transporter, periplasmic oligopeptide-binding protein OppA (TC 3.A.1.5.1) |
| contig_11 | 6523 | 6975 | + | Oligopeptide ABC transporter, periplasmic oligopeptide-binding protein OppA (TC 3.A.1.5.1) |
| contig_11 | 7793 | 7020 | - | Glutamate synthase [NADPH] large chain (EC 1.4.1.13) |
| contig_11 | 8161 | 7901 | - | Glutamate synthase [NADPH] large chain (EC 1.4.1.13) |
| contig_11 | 8398 | 8219 | - | hypothetical protein |
| contig_11 | 8826 | 8545 | - | Transcriptional regulator, TetR family |
| contig_11 | 9075 | 8911 | - | Transcriptional regulator, AraC family |
| contig_110 | 682 | 113 | - | sensory box histidine kinase VicK, putative( EC:2.7.3.- ) |
| contig_110 | 946 | 833 | - | sensory box histidine kinase VicK, putative( EC:2.7.3.- ) |
| contig_110 | 1453 | 1136 | - | sensory box histidine kinase VicK, putative( EC:2.7.3.- ) |
| contig_110 | 1638 | 1453 | - | DNA-binding response regulator |
| contig_1101 | 49 | 408 | + | Biotin synthesis protein BioH |
| contig_1101 | 374 | 1000 | + | Biotin synthesis protein BioC |
| contig_1101 | 957 | 1190 | + | Biotin synthesis protein BioC |
| contig_1101 | 1251 | 2186 | + | Biotin synthase (EC 2.8.1.6) |
| contig_1102 | 549 | 184 | - | hypothetical protein |
| contig_1102 | 677 | 546 | - | hypothetical protein |
| contig_1104 | 158 | 45 | - | hypothetical protein |
| contig_1104 | 728 | 330 | - | 4Fe-4S ferredoxin, iron-sulfur binding |
| contig_1104 | 1762 | 725 | - | 4-hydroxyproline epimerase (EC 5.1.1.8) |
| contig_1104 | 1980 | 1759 | - | D-amino-acid oxidase (EC 1.4.3.3) |
| contig_1105 | 1063 | 386 | - | nitroreductase |
| contig_1105 | 1530 | 1093 | - | Streptolysin S biosynthesis protein D (SagD) |
| contig_1105 | 2387 | 1686 | - | Streptolysin S biosynthesis protein D (SagD) |
| contig_1105 | 3359 | 2664 | - | FIG01230282: hypothetical protein |
| contig_1106 | 15 | 131 | + | FIG01226798: hypothetical protein |
| contig_1108 | 28 | 837 | + | Flagellar motor rotation protein MotB |
| contig_1109 | 297 | 620 | + | Sodium-dependent phosphate transporter |
| contig_1109 | 575 | 1300 | + | Sodium-dependent phosphate transporter |
| contig_1109 | 1260 | 1955 | + | Sodium-dependent phosphate transporter |
| contig_111 | 779 | 624 | - | sensory box histidine kinase VicK, putative( EC:2.7.3.- ) |
| contig_111 | 1221 | 859 | - | sensory box histidine kinase VicK, putative( EC:2.7.3.- ) |
| contig_111 | 2000 | 1425 | - | sensory box histidine kinase VicK, putative( EC:2.7.3.- ) |
| contig_111 | 2475 | 2047 | - | sensory box histidine kinase VicK, putative( EC:2.7.3.- ) |
| contig_1110 | 417 | 22 | - | Alcohol dehydrogenase (EC 1.1.1.1) |
| contig_1110 | 796 | 395 | - | Alcohol dehydrogenase (EC 1.1.1.1) |
| contig_1110 | 1112 | 933 | - | Alcohol dehydrogenase (EC 1.1.1.1) |
| contig_1111 | 936 | 283 | - | Sucrose phosphorylase (EC 2.4.1.7) |
| contig_1111 | 1189 | 986 | - | Sucrose phosphorylase (EC 2.4.1.7) |
| contig_1111 | 1443 | 1186 | - | Sucrose phosphorylase (EC 2.4.1.7) |
| contig_1111 | 1664 | 1455 | - | FIG01225936: hypothetical protein |
| contig_1112 | 714 | 193 | - | Cytochrome c-type biogenesis protein ResA |
| contig_1112 | 1536 | 808 | - | Ribosomal large subunit pseudouridine synthase B (EC 4.2.1.70) |
| contig_1112 | 2143 | 1823 | - | Spore maturation protein B |
| contig_1112 | 2355 | 2140 | - | Spore maturation protein B |
| contig_1112 | 2897 | 2352 | - | Spore maturation protein A |
| contig_1112 | 3449 | 3234 | - | D-alanyl-D-alanine carboxypeptidase (EC 3.4.16.4) |
| contig_1112 | 4071 | 3433 | - | D-alanyl-D-alanine carboxypeptidase (EC 3.4.16.4) |
| contig_1112 | 4498 | 4277 | - | Superoxide dismutase [Fe] (EC 1.15.1.1) @ Exosporium SOD |
| contig_1113 | 1688 | 624 | - | Chitinase (EC 3.2.1.14) |
| contig_1113 | 2490 | 2365 | - | Glycerate kinase (EC 2.7.1.31) |
| contig_1113 | 2930 | 2568 | - | Glycerate kinase (EC 2.7.1.31) |
| contig_1113 | 3288 | 2899 | - | Glycerate kinase (EC 2.7.1.31) |
| contig_1113 | 3651 | 3397 | - | oxidoreductase of aldo/keto reductase family, subgroup 1 |
| contig_1115 | 344 | 201 | - | Transcriptional regulator, MarR family |
| contig_1115 | 503 | 649 | + | outer membrane protein CC2294 |
| contig_1115 | 676 | 1176 | + | outer membrane protein CC2294 |
| contig_1115 | 1512 | 1336 | - | FIG01237460: hypothetical protein |
| contig_1115 | 1511 | 1954 | + | Guanine-hypoxanthine permease |
| contig_1115 | 2094 | 2600 | + | Guanine-hypoxanthine permease |
| contig_1115 | 3585 | 2635 | - | Magnesium and cobalt transport protein CorA |
| contig_1115 | 3803 | 3660 | - | Pyrimidine-nucleoside phosphorylase (EC 2.4.2.2) |
| contig_1117 | 844 | 365 | - | permease, putative |
| contig_1118 | 589 | 125 | - | site-specific recombinase, resolvase family |
| contig_1118 | 680 | 1039 | + | SOS-response repressor and protease LexA (EC 3.4.21.88) |
| contig_1118 | 1086 | 1352 | + | SOS-response repressor and protease LexA (EC 3.4.21.88) |
| contig_1118 | 1649 | 1380 | - | FIG01226476: hypothetical protein |
| contig_1118 | 1894 | 1760 | - | FIG01226476: hypothetical protein |
| contig_1118 | 2180 | 1887 | - | FIG01226476: hypothetical protein |
| contig_1118 | 2805 | 2332 | - | FIG01226660: hypothetical protein |
| contig_1118 | 3127 | 2876 | - | Transcriptional regulator, PadR family |
| contig_1118 | 4672 | 3506 | - | Glutamine synthetase type I (EC 6.3.1.2) |
| contig_1118 | 4841 | 4650 | - | Glutamine synthetase type I (EC 6.3.1.2) |
| contig_1118 | 5024 | 4890 | - | Transcriptional regulator, MerR family |
| contig_1119 | 524 | 372 | - | Argininosuccinate lyase (EC 4.3.2.1) |
| contig_112 | 1032 | 127 | - | Dihydroxyacetone kinase, ATP-dependent (EC 2.7.1.29) |
| contig_112 | 1532 | 1131 | - | Dihydroxyacetone kinase, ATP-dependent (EC 2.7.1.29) |
| contig_112 | 1766 | 2065 | + | Transcriptional regulator, TetR family |
| contig_112 | 2062 | 2301 | + | Transcriptional regulator, TetR family |
| contig_112 | 2298 | 2498 | + | dihydroxyacetone kinase family protein |
| contig_112 | 2507 | 3298 | + | dihydroxyacetone kinase family protein |
| contig_112 | 3552 | 3397 | - | membrane protein, putative |
| contig_112 | 4034 | 3648 | - | membrane protein, putative |
| contig_1120 | 1584 | 22 | - | Glycosyl transferase, group 2 family protein |
| contig_1120 | 2542 | 1577 | - | dTDP-glucose 4,6-dehydratase (EC 4.2.1.46) |
| contig_1120 | 2691 | 2539 | - | UDP-glucose/GDP-mannose dehydrogenase family |
| contig_1120 | 2881 | 2693 | - | UDP-glucose 6-dehydrogenase |
| contig_1121 | 21 | 455 | + | L-Proline/Glycine betaine transporter ProP |
| contig_1121 | 469 | 606 | + | L-Proline/Glycine betaine transporter ProP |
| contig_1121 | 584 | 751 | + | L-Proline/Glycine betaine transporter ProP |
| contig_1121 | 885 | 1175 | + | L-Proline/Glycine betaine transporter ProP |
| contig_1121 | 1377 | 1258 | - | Phosphonate ABC transporter phosphate-binding periplasmic component (TC 3.A.1.9.1) |
| contig_1122 | 66 | 287 | + | Transcriptional repressor of the fructose operon, DeoR family |
| contig_1122 | 718 | 455 | - | Ribosomal small subunit pseudouridine synthase A (EC 4.2.1.70) |
| contig_1122 | 1002 | 679 | - | Ribosomal small subunit pseudouridine synthase A (EC 4.2.1.70) |
| contig_1123 | 504 | 665 | + | HD domain protein |
| contig_1123 | 730 | 1080 | + | HD domain protein |
| contig_1123 | 1077 | 1259 | + | COG0488: ATPase components of ABC transporters with duplicated ATPase domains |
| contig_1123 | 1256 | 1603 | + | COG0488: ATPase components of ABC transporters with duplicated ATPase domains |
| contig_1123 | 1560 | 1883 | + | COG0488: ATPase components of ABC transporters with duplicated ATPase domains |
| contig_1123 | 1843 | 2253 | + | COG0488: ATPase components of ABC transporters with duplicated ATPase domains |
| contig_1123 | 2258 | 2722 | + | COG0488: ATPase components of ABC transporters with duplicated ATPase domains |
| contig_1124 | 404 | 105 | - | Glyoxalase family protein |
| contig_1124 | 694 | 575 | - | hypothetical protein |
| contig_1124 | 1233 | 892 | - | ATP-dependent RNA helicase YfmL |
| contig_1124 | 1454 | 1248 | - | ATP-dependent RNA helicase YfmL |
| contig_1125 | 75 | 296 | + | hypothetical protein |
| contig_1125 | 997 | 1197 | + | COG1180: Radical SAM, Pyruvate-formate lyase-activating enzyme like |
| contig_1125 | 1163 | 1459 | + | COG1180: Radical SAM, Pyruvate-formate lyase-activating enzyme like |
| contig_1125 | 1578 | 1886 | + | COG1180: Radical SAM, Pyruvate-formate lyase-activating enzyme like |
| contig_1125 | 2313 | 2447 | + | Sterol-regulatory element binding protein (SREBP) site 2 protease family protein |
| contig_1125 | 2401 | 2535 | + | Sterol-regulatory element binding protein (SREBP) site 2 protease family protein |
| contig_1126 | 317 | 96 | - | Methionyl-tRNA formyltransferase (EC 2.1.2.9) |
| contig_1126 | 423 | 268 | - | Methionyl-tRNA formyltransferase (EC 2.1.2.9) |
| contig_1126 | 617 | 420 | - | Methionyl-tRNA formyltransferase (EC 2.1.2.9) |
| contig_1126 | 999 | 604 | - | Methionyl-tRNA formyltransferase (EC 2.1.2.9) |
| contig_1126 | 1242 | 1069 | - | Peptide deformylase (EC 3.5.1.88) |
| contig_1127 | 83 | 199 | + | Spo0E-like putative sporulation regulatory protein in grePA-PF operon |
| contig_1127 | 296 | 517 | + | Protein GerPA, required for proper assembly of spore coat, mutations lead to super-dormant spore |
| contig_1127 | 578 | 739 | + | Protein GerPB, required for proper assembly of spore coat, mutations lead to super-dormant spore |
| contig_1127 | 810 | 1013 | + | Protein GerPC, required for proper assembly of spore coat, mutations lead to super-dormant spore |
| contig_1127 | 1001 | 1429 | + | Protein GerPC, required for proper assembly of spore coat, mutations lead to super-dormant spore |
| contig_1127 | 1436 | 1630 | + | Protein GerPD, required for proper assembly of spore coat, mutations lead to super-dormant spore |
| contig_1127 | 1646 | 2032 | + | Protein GerPE, required for proper assembly of spore coat, mutations lead to super-dormant spore |
| contig_1127 | 2075 | 2290 | + | Protein GerPF, required for proper assembly of spore coat, mutations lead to super-dormant spore |
| contig_1129 | 14 | 241 | + | Na+ dependent nucleoside transporter NupC |
| contig_1129 | 213 | 842 | + | Nucleoside permease NupC |
| contig_1129 | 1165 | 1497 | + | Nucleoside permease NupC |
| contig_113 | 379 | 254 | - | hypothetical protein |
| contig_113 | 446 | 733 | + | Programmed cell death antitoxin YdcD |
| contig_113 | 738 | 1088 | + | Programmed cell death toxin YdcE |
| contig_1131 | 644 | 42 | - | HMP-PP hydrolase (pyridoxal phosphatase) Cof, detected in genetic screen for thiamin metabolic genes (PMID:15292217) |
| contig_1131 | 756 | 965 | + | Flagellar motor rotation protein MotA |
| contig_1132 | 104 | 337 | + | Possible caffeoyl-CoA O-methyltransferase (EC 2.1.1.104) |
| contig_1133 | 166 | 477 | + | Oligopeptide ABC transporter, periplasmic oligopeptide-binding protein OppA (TC 3.A.1.5.1) |
| contig_1134 | 440 | 30 | - | TPR repeat protein |
| contig_1135 | 238 | 11 | - | TPR repeat protein |
| contig_1135 | 612 | 337 | - | TPR repeat protein |
| contig_1136 | 142 | 576 | + | Aspartyl-tRNA synthetase (EC 6.1.1.12) @ Aspartyl-tRNA(Asn) synthetase (EC 6.1.1.23) |
| contig_1136 | 839 | 645 | - | putative transcription regulator |
| contig_1136 | 1551 | 1390 | - | Mobile element protein |
| contig_1137 | 454 | 8 | - | Nucleoside diphosphate kinase (EC 2.7.4.6) |
| contig_1137 | 717 | 580 | - | Heptaprenyl diphosphate synthase component II (EC 2.5.1.30) |
| contig_1137 | 1283 | 771 | - | Heptaprenyl diphosphate synthase component II (EC 2.5.1.30) |
| contig_1137 | 1545 | 1258 | - | Heptaprenyl diphosphate synthase component II (EC 2.5.1.30) |
| contig_1137 | 1750 | 1577 | - | 2-heptaprenyl-1,4-naphthoquinone methyltransferase (EC 2.1.1.163) |
| contig_1137 | 2291 | 1734 | - | 2-heptaprenyl-1,4-naphthoquinone methyltransferase (EC 2.1.1.163) |
| contig_1137 | 2458 | 2321 | - | Heptaprenyl diphosphate synthase component I (EC 2.5.1.30) |
| contig_1137 | 2880 | 2551 | - | Heptaprenyl diphosphate synthase component I (EC 2.5.1.30) |
| contig_1137 | 3070 | 2861 | - | Heptaprenyl diphosphate synthase component I (EC 2.5.1.30) |
| contig_1139 | 179 | 301 | + | ABC transporter permease protein |
| contig_1139 | 282 | 446 | + | ABC transporter permease protein |
| contig_1139 | 532 | 756 | + | ABC transporter permease protein |
| contig_1139 | 838 | 1047 | + | ABC transporter, ATP-binding protein |
| contig_1139 | 1071 | 1517 | + | ABC transporter, ATP-binding protein |
| contig_1139 | 1489 | 1671 | + | ABC transporter, ATP-binding protein |
| contig_1139 | 1650 | 1763 | + | ABC transporter, ATP-binding protein |
| contig_1139 | 1756 | 2874 | + | Bacitracin transport permease protein BCRB |
| contig_1139 | 3600 | 2839 | - | N-acetyl-L,L-diaminopimelate deacetylase homolog (EC 3.5.1.18) |
| contig_114 | 318 | 157 | - | FIG01225137: hypothetical protein |
| contig_114 | 597 | 358 | - | Oxygen-insensitive NAD(P)H nitroreductase (EC 1.-.-.-) / Dihydropteridine reductase (EC 1.5.1.34) |
| contig_114 | 826 | 575 | - | Oxygen-insensitive NAD(P)H nitroreductase (EC 1.-.-.-) / Dihydropteridine reductase (EC 1.5.1.34) |
| contig_114 | 1028 | 816 | - | FIG01226231: hypothetical protein |
| contig_114 | 1516 | 1025 | - | Hydroxymethylpyrimidine ABC transporter, ATPase component |
| contig_114 | 1845 | 1525 | - | Hydroxymethylpyrimidine ABC transporter, ATPase component |
| contig_114 | 2840 | 1842 | - | Hydroxymethylpyrimidine ABC transporter, substrate-binding component |
| contig_114 | 3604 | 2837 | - | Hydroxymethylpyrimidine ABC transporter, transmembrane component |
| contig_114 | 3707 | 3582 | - | FIG01226054: hypothetical protein |
| contig_114 | 3903 | 3670 | - | FIG01226054: hypothetical protein |
| contig_114 | 5495 | 4281 | - | methyl-accepting chemotaxis protein |
| contig_114 | 5637 | 5479 | - | methyl-accepting chemotaxis protein |
| contig_114 | 5776 | 5630 | - | FIG01227508: hypothetical protein |
| contig_114 | 6161 | 5964 | - | FIG01228921: hypothetical protein |
| contig_1140 | 128 | 310 | + | Flagellar biosynthesis protein FlhF |
| contig_1140 | 301 | 1116 | + | Flagellar biosynthesis protein FlhF |
| contig_1140 | 1159 | 1497 | + | Flagellar basal-body rod protein FlgG |
| contig_1140 | 1576 | 1935 | + | Flagellar basal-body rod protein FlgG |
| contig_1141 | 324 | 187 | - | FIG002343: hypothetical protein |
| contig_1142 | 411 | 43 | - | Copper-translocating P-type ATPase (EC 3.6.3.4) |
| contig_1142 | 1331 | 471 | - | Lead, cadmium, zinc and mercury transporting ATPase (EC 3.6.3.3) (EC 3.6.3.5); Copper-translocating P-type ATPase (EC 3.6.3.4) |
| contig_1142 | 1479 | 1318 | - | Lead, cadmium, zinc and mercury transporting ATPase (EC 3.6.3.3) (EC 3.6.3.5); Copper-translocating P-type ATPase (EC 3.6.3.4) |
| contig_1142 | 2222 | 1521 | - | Lead, cadmium, zinc and mercury transporting ATPase (EC 3.6.3.3) (EC 3.6.3.5); Copper-translocating P-type ATPase (EC 3.6.3.4) |
| contig_1143 | 162 | 49 | - | sensory box/GGDEF family protein |
| contig_1143 | 271 | 140 | - | sensory box/GGDEF family protein |
| contig_1143 | 606 | 385 | - | sensory box/GGDEF family protein |
| contig_1143 | 917 | 789 | - | sensory box/GGDEF family protein |
| contig_1143 | 1030 | 917 | - | sensory box/GGDEF family protein |
| contig_1143 | 1241 | 1035 | - | sensory box/GGDEF family protein |
| contig_1143 | 1887 | 1342 | - | sensory box/GGDEF family protein |
| contig_1145 | 386 | 39 | - | Resolvase |
| contig_1147 | 1425 | 934 | - | Lipase (EC 3.1.1.3) |
| contig_1148 | 1033 | 782 | - | Exopolyphosphatase (EC 3.6.1.11) |
| contig_1148 | 1360 | 1130 | - | Exopolyphosphatase (EC 3.6.1.11) |
| contig_1148 | 1551 | 1372 | - | Exopolyphosphatase (EC 3.6.1.11) |
| contig_1149 | 137 | 24 | - | Possible serine/threonine specific protein phosphatase (EC 3.1.3.16) |
| contig_1149 | 658 | 134 | - | Possible serine/threonine specific protein phosphatase (EC 3.1.3.16) |
| contig_115 | 11 | 724 | + | NAD(FAD)-utilizing dehydrogenases |
| contig_115 | 678 | 845 | + | NAD(FAD)-utilizing dehydrogenases |
| contig_1150 | 581 | 333 | - | Transaldolase (EC 2.2.1.2) |
| contig_1154 | 223 | 98 | - | Two-component response regulator |
| contig_1154 | 439 | 639 | + | FIG01226376: hypothetical protein |
| contig_1154 | 996 | 799 | - | acetyltransferase, GNAT family |
| contig_1156 | 181 | 53 | - | Phosphoribosylaminoimidazole-succinocarboxamide synthase (EC 6.3.2.6) |
| contig_1156 | 665 | 174 | - | Phosphoribosylaminoimidazole-succinocarboxamide synthase (EC 6.3.2.6) |
| contig_1156 | 1932 | 754 | - | Adenylosuccinate lyase (EC 4.3.2.2) |
| contig_1156 | 2098 | 1913 | - | Adenylosuccinate lyase (EC 4.3.2.2) |
| contig_1156 | 2199 | 2059 | - | Phosphoribosylaminoimidazole carboxylase ATPase subunit (EC 4.1.1.21) |
| contig_1156 | 3211 | 2153 | - | Phosphoribosylaminoimidazole carboxylase ATPase subunit (EC 4.1.1.21) |
| contig_1156 | 3693 | 3208 | - | Phosphoribosylaminoimidazole carboxylase catalytic subunit (EC 4.1.1.21) |
| contig_1157 | 35 | 541 | + | Ferritin-like protein 2 |
| contig_1157 | 799 | 506 | - | FIG01229159: hypothetical protein |
| contig_1158 | 17 | 157 | + | Xylulose kinase (EC 2.7.1.17) |
| contig_1158 | 182 | 322 | + | Xylulose kinase (EC 2.7.1.17) |
| contig_1158 | 494 | 1042 | + | D-xylose-specific 1-epimerase (mutarotase) |
| contig_116 | 281 | 18 | - | putative membrane protein |
| contig_116 | 655 | 446 | - | putative membrane protein |
| contig_116 | 900 | 784 | - | FIG01235760: hypothetical protein |
| contig_116 | 974 | 1201 | + | FIG01226828: hypothetical protein |
| contig_116 | 1296 | 1412 | + | hypothetical protein |
| contig_116 | 2035 | 1463 | - | Conserved protein YqhG |
| contig_1160 | 178 | 303 | + | FIG005590: DegV family protein |
| contig_1160 | 269 | 391 | + | FIG005590: DegV family protein |
| contig_1160 | 418 | 1023 | + | FIG005590: DegV family protein |
| contig_1160 | 1004 | 1126 | + | hypothetical protein |
| contig_1160 | 1197 | 1520 | + | Transcriptional regulator, HxlR family |
| contig_1161 | 41 | 529 | + | Polymyxin transporter PmxC |
| contig_1161 | 642 | 1004 | + | Polymyxin transporter PmxC |
| contig_1161 | 1064 | 1237 | + | 4'-phosphopantetheinyl transferase (EC 2.7.8.-) in polymyxin biosynthetic cluster |
| contig_1161 | 1282 | 1674 | + | 4'-phosphopantetheinyl transferase (EC 2.7.8.-) in polymyxin biosynthetic cluster |
| contig_1161 | 1853 | 1969 | + | Uncharacterized protein in polymyxin biosynthetic cluster |
| contig_1161 | 1962 | 2327 | + | Uncharacterized protein in polymyxin biosynthetic cluster |
| contig_1161 | 2709 | 2437 | - | DNA-binding protein HBsu |
| contig_1161 | 2897 | 3022 | + | FIG01226788: hypothetical protein |
| contig_1161 | 3090 | 3326 | + | FIG01226788: hypothetical protein |
| contig_1161 | 3487 | 3600 | + | FIG01226788: hypothetical protein |
| contig_1163 | 357 | 124 | - | DNA-binding response regulator |
| contig_1163 | 632 | 462 | - | Multidrug resistance ABC transporter ATP-binding and permease protein |
| contig_1163 | 1078 | 677 | - | Lipid A export ATP-binding/permease protein MsbA |
| contig_1163 | 1223 | 1029 | - | hypothetical protein |
| contig_1163 | 1878 | 1192 | - | Multidrug resistance ABC transporter ATP-binding and permease protein |
| contig_1163 | 2212 | 2039 | - | Lipid A export ATP-binding/permease protein MsbA |
| contig_1164 | 73 | 246 | + | FIG01236086: hypothetical protein |
| contig_1164 | 598 | 807 | + | FIG01236086: hypothetical protein |
| contig_1164 | 834 | 1070 | + | FIG01230325: hypothetical protein |
| contig_1164 | 1126 | 1467 | + | FIG01230325: hypothetical protein |
| contig_1164 | 2122 | 2009 | - | lipoprotein, NLP/P60 family |
| contig_1165 | 289 | 167 | - | ABC transporter, permease protein, putative |
| contig_1165 | 462 | 346 | - | ABC transporter, permease protein, putative |
| contig_1166 | 1933 | 662 | - | Chitinase (EC 3.2.1.14) |
| contig_1166 | 2175 | 2014 | - | Chitinase (EC 3.2.1.14) |
| contig_1168 | 718 | 302 | - | FIG01227869: hypothetical protein |
| contig_1168 | 948 | 1328 | + | Transcriptional regulator, MerR family |
| contig_1168 | 1468 | 1842 | + | Magnesium and cobalt efflux protein CorC |
| contig_1168 | 1878 | 2024 | + | Magnesium and cobalt efflux protein CorC |
| contig_1169 | 71 | 901 | + | hydrolase, alpha/beta fold family, putative |
| contig_1169 | 1388 | 2476 | + | Histidine permease YuiF |
| contig_1169 | 2887 | 2546 | - | Sugar-binding transcriptional regulator, LacI family, putative |
| contig_1169 | 3060 | 2944 | - | Sugar-binding transcriptional regulator, LacI family, putative |
| contig_1169 | 3382 | 3041 | - | Sugar-binding transcriptional regulator, LacI family, putative |
| contig_117 | 73 | 357 | + | hypothetical protein |
| contig_117 | 478 | 621 | + | hypothetical protein |
| contig_117 | 656 | 1765 | + | DNA primase/helicase, phage-associated |
| contig_117 | 1881 | 2882 | + | DNA primase/helicase, phage-associated |
| contig_117 | 3278 | 3706 | + | hypothetical protein |
| contig_117 | 3709 | 3957 | + | hypothetical protein |
| contig_117 | 4193 | 4032 | - | hypothetical protein |
| contig_117 | 4305 | 4625 | + | Phage-associated homing endonuclease |
| contig_117 | 4670 | 5092 | + | HNH endonuclease:HNH nuclease |
| contig_117 | 5342 | 5629 | + | Phage-related protein |
| contig_117 | 5631 | 5915 | + | ORF137 |
| contig_117 | 5996 | 6340 | + | hypothetical protein |
| contig_117 | 6401 | 6820 | + | Dimeric dUTPase (EC 3.6.1.23) |
| contig_117 | 6814 | 7035 | + | Dimeric dUTPase (EC 3.6.1.23) |
| contig_117 | 7128 | 7298 | + | hypothetical protein |
| contig_117 | 7285 | 7419 | + | hypothetical protein |
| contig_1170 | 439 | 305 | - | Flavin reductase (EC 1.5.1.30) |
| contig_1172 | 226 | 104 | - | ATP/GTP-binding protein, SA1392 homolog |
| contig_1172 | 888 | 256 | - | CBS domain protein, lmo1865 homolog |
| contig_1172 | 1642 | 1013 | - | DNA recombination and repair protein RecO |
| contig_1172 | 1923 | 1780 | - | hypothetical protein |
| contig_1172 | 2252 | 2127 | - | GTP-binding protein Era |
| contig_1172 | 3006 | 2296 | - | GTP-binding protein Era |
| contig_1172 | 3397 | 2999 | - | Cytidine deaminase (EC 3.5.4.5) |
| contig_1172 | 4225 | 3497 | - | Metal-dependent hydrolase YbeY, involved in rRNA and/or ribosome maturation and assembly |
| contig_1172 | 5290 | 4388 | - | Membrane protein containing HD superfamily hydrolase domain, YQFF ortholog |
| contig_1172 | 5865 | 5323 | - | Membrane protein containing HD superfamily hydrolase domain, YQFF ortholog |
| contig_1172 | 6097 | 5858 | - | Membrane protein containing HD superfamily hydrolase domain, YQFF ortholog |
| contig_1172 | 6464 | 6261 | - | Membrane protein containing HD superfamily hydrolase domain, YQFF ortholog |
| contig_1173 | 548 | 72 | - | Acid phosphatase (EC 3.1.3.2) |
| contig_1174 | 374 | 231 | - | 3-hydroxyanthranilate 3,4-dioxygenase (EC 1.13.11.6) |
| contig_1174 | 540 | 352 | - | 3-hydroxyanthranilate 3,4-dioxygenase (EC 1.13.11.6) |
| contig_1174 | 696 | 553 | - | Riboflavin kinase (EC 2.7.1.26) / FMN adenylyltransferase (EC 2.7.7.2) |
| contig_1175 | 8 | 349 | + | D-alanyl-D-alanine carboxypeptidase (EC 3.4.16.4) |
| contig_1175 | 1214 | 1032 | - | DUF1093 domain-containing protein |
| contig_1176 | 538 | 404 | - | Alcohol dehydrogenase (EC 1.1.1.1) |
| contig_1176 | 747 | 583 | - | Alcohol dehydrogenase (EC 1.1.1.1) |
| contig_1176 | 1072 | 770 | - | Alcohol dehydrogenase (EC 1.1.1.1) |
| contig_1176 | 1400 | 1254 | - | Alcohol dehydrogenase (EC 1.1.1.1) |
| contig_1176 | 2293 | 1700 | - | Two-component sensor histidine kinase |
| contig_1177 | 166 | 315 | + | Phosphate:acyl-ACP acyltransferase PlsX |
| contig_1177 | 338 | 829 | + | Phosphate:acyl-ACP acyltransferase PlsX |
| contig_1177 | 826 | 1818 | + | Phosphate:acyl-ACP acyltransferase PlsX |
| contig_1177 | 1833 | 2777 | + | Malonyl CoA-acyl carrier protein transacylase (EC 2.3.1.39) |
| contig_1177 | 2777 | 2890 | + | hypothetical protein |
| contig_1177 | 2991 | 3461 | + | 3-oxoacyl-[acyl-carrier protein] reductase (EC 1.1.1.100) |
| contig_1177 | 3589 | 3822 | + | Acyl carrier protein |
| contig_1177 | 4049 | 4627 | + | Ribonuclease III (EC 3.1.26.3) |
| contig_1177 | 4815 | 5582 | + | Chromosome partition protein smc |
| contig_1178 | 729 | 67 | - | Diaminopimelate decarboxylase (EC 4.1.1.20) |
| contig_1179 | 113 | 241 | + | ABC1 family protein |
| contig_1179 | 1188 | 703 | - | FIG01227965: hypothetical protein |
| contig_1179 | 1959 | 1381 | - | RNA polymerase sigma-70 factor, ECF subfamily |
| contig_1179 | 2165 | 2722 | + | Putative Zn-dependent oxidoreductase BA2113 |
| contig_1179 | 2682 | 3152 | + | Putative Zn-dependent oxidoreductase BA2113 |
| contig_1179 | 3182 | 3673 | + | FIG01225655: hypothetical protein |
| contig_1179 | 3648 | 3785 | + | FIG01225655: hypothetical protein |
| contig_118 | 284 | 33 | - | FIG01226547: hypothetical protein |
| contig_118 | 955 | 482 | - | FIG01226547: hypothetical protein |
| contig_118 | 1274 | 1426 | + | HesB-like, HesB-like domain |
| contig_118 | 1434 | 1598 | + | HesB-like, HesB-like domain |
| contig_1181 | 54 | 572 | + | sensor histidine kinase |
| contig_1181 | 682 | 843 | + | sensor histidine kinase |
| contig_1182 | 48 | 212 | + | Spore germination protein GerHA/GerIA |
| contig_1182 | 217 | 444 | + | Spore germination protein GerHA/GerIA |
| contig_1182 | 441 | 1418 | + | Spore germination protein GerHA/GerIA |
| contig_1182 | 1436 | 1630 | + | Spore germination protein GerHB/GerIB |
| contig_1182 | 1609 | 2130 | + | Spore germination protein GerHB/GerIB |
| contig_1182 | 2199 | 2537 | + | Spore germination protein GerHB/GerIB |
| contig_1182 | 2507 | 2695 | + | Spore germination protein GerHC/GerIC |
| contig_1183 | 665 | 441 | - | response regulator, putative |
| contig_1183 | 1704 | 1405 | - | Ribosome protection-type tetracycline resistance related proteins |
| contig_1183 | 2318 | 1728 | - | Ribosome protection-type tetracycline resistance related proteins |
| contig_1183 | 2696 | 2382 | - | Ribosome protection-type tetracycline resistance related proteins |
| contig_1183 | 3050 | 2892 | - | Ribosome protection-type tetracycline resistance related proteins |
| contig_1183 | 3303 | 3106 | - | Ribosome protection-type tetracycline resistance related proteins |
| contig_1184 | 1282 | 356 | - | S-layer protein, putative |
| contig_1185 | 211 | 8 | - | UPF0229 protein YeaH |
| contig_1186 | 887 | 723 | - | FIG01227274: hypothetical protein |
| contig_1186 | 2015 | 1860 | - | possible carbamoyl-phosphate synthase large chain |
| contig_1186 | 2182 | 2030 | - | possible carbamoyl-phosphate synthase large chain |
| contig_1187 | 20 | 196 | + | FIG01225390: hypothetical protein |
| contig_1187 | 237 | 359 | + | FIG01225390: hypothetical protein |
| contig_1188 | 417 | 1 | - | ATP-dependent DNA helicase RecQ |
| contig_1188 | 734 | 390 | - | ATP-dependent DNA helicase RecQ |
| contig_1188 | 1011 | 751 | - | ATP-dependent DNA helicase RecQ |
| contig_1189 | 1035 | 130 | - | CDS_ID OB3077 |
| contig_1189 | 2058 | 1204 | - | Coenzyme F420-dependent N5,N10-methylene tetrahydromethanopterin reductase and related flavin-dependent oxidoreductases; sulfonate monooxygenase |
| contig_1189 | 3228 | 2947 | - | FIG01227038: hypothetical protein |
| contig_119 | 416 | 817 | + | Catabolite control protein A |
| contig_119 | 817 | 1371 | + | Catabolite control protein A |
| contig_119 | 1699 | 1406 | - | FIG01226819: hypothetical protein |
| contig_119 | 2010 | 1774 | - | FIG01228570: hypothetical protein |
| contig_119 | 2309 | 2097 | - | MinD family ATPase domain protein |
| contig_119 | 2546 | 2818 | + | FIG01228994: hypothetical protein |
| contig_119 | 3265 | 2900 | - | Large-conductance mechanosensitive channel |
| contig_119 | 3840 | 3394 | - | Virulence factor MviM |
| contig_119 | 4321 | 3830 | - | Virulence factor MviM |
| contig_119 | 4690 | 4803 | + | hypothetical protein |
| contig_119 | 5186 | 4938 | - | hydrolase, alpha/beta fold family |
| contig_119 | 5778 | 5191 | - | hydrolase, alpha/beta fold family |
| contig_1190 | 576 | 109 | - | Glutamate transport membrane-spanning protein |
| contig_1190 | 766 | 647 | - | Glutamine ABC transporter, periplasmic glutamine-binding protein (TC 3.A.1.3.2) |
| contig_1190 | 1121 | 783 | - | Glutamine ABC transporter, periplasmic glutamine-binding protein (TC 3.A.1.3.2) |
| contig_1190 | 1479 | 1144 | - | Glutamine ABC transporter, periplasmic glutamine-binding protein (TC 3.A.1.3.2) |
| contig_1190 | 2280 | 1492 | - | Glutamate transport ATP-binding protein |
| contig_1190 | 2387 | 2238 | - | Glutamate transport ATP-binding protein |
| contig_1190 | 2625 | 2356 | - | sodium/alanine symporter family protein |
| contig_1191 | 421 | 272 | - | FIG01226901: hypothetical protein |
| contig_1191 | 844 | 506 | - | FIG01226901: hypothetical protein |
| contig_1193 | 76 | 522 | + | Magnesium and cobalt efflux protein CorC |
| contig_1193 | 503 | 619 | + | Magnesium and cobalt efflux protein CorC |
| contig_1196 | 431 | 78 | - | phage integrase family protein |
| contig_1196 | 610 | 437 | - | phage integrase family protein |
| contig_1196 | 1434 | 622 | - | phage integrase family protein |
| contig_1196 | 1670 | 1524 | - | FIG01235768: hypothetical protein |
| contig_1196 | 1991 | 1704 | - | FIG01235768: hypothetical protein |
| contig_1198 | 281 | 871 | + | Helix-turn-helix domain protein |
| contig_1198 | 1230 | 1042 | - | Ammonium transporter |
| contig_1198 | 2236 | 1256 | - | Ammonium transporter |
| contig_12 | 1497 | 193 | - | UDP-N-acetylglucosamine 1-carboxyvinyltransferase (EC 2.5.1.7) |
| contig_12 | 2040 | 1537 | - | FIG013354: hypothetical protein |
| contig_12 | 2242 | 2114 | - | FIG013354: hypothetical protein |
| contig_12 | 2530 | 2294 | - | FIG01225921: hypothetical protein |
| contig_12 | 4116 | 2743 | - | NADH-ubiquinone oxidoreductase chain N (EC 1.6.5.3) |
| contig_12 | 5768 | 4266 | - | NADH-ubiquinone oxidoreductase chain M (EC 1.6.5.3) |
| contig_12 | 6601 | 5765 | - | NADH-ubiquinone oxidoreductase chain L (EC 1.6.5.3) |
| contig_12 | 7628 | 6615 | - | NADH-ubiquinone oxidoreductase chain L (EC 1.6.5.3) |
| contig_12 | 7972 | 7658 | - | NADH-ubiquinone oxidoreductase chain K (EC 1.6.5.3) |
| contig_12 | 8489 | 7965 | - | NADH-ubiquinone oxidoreductase chain J (EC 1.6.5.3) |
| contig_12 | 8904 | 8590 | - | NADH-ubiquinone oxidoreductase chain I (EC 1.6.5.3) |
| contig_12 | 9127 | 8930 | - | NADH-ubiquinone oxidoreductase chain H (EC 1.6.5.3) |
| contig_12 | 9804 | 9145 | - | NADH-ubiquinone oxidoreductase chain H (EC 1.6.5.3) |
| contig_12 | 11032 | 9932 | - | NADH-ubiquinone oxidoreductase chain D (EC 1.6.5.3) |
| contig_12 | 11930 | 11037 | - | NADH-ubiquinone oxidoreductase chain C (EC 1.6.5.3) |
| contig_120 | 680 | 555 | - | MFS general substrate transporter |
| contig_120 | 1386 | 655 | - | MFS general substrate transporter |
| contig_1200 | 59 | 721 | + | Gluconokinase (EC 2.7.1.12) |
| contig_1200 | 788 | 1021 | + | Gluconokinase (EC 2.7.1.12) |
| contig_1200 | 1174 | 2139 | + | 6-phosphogluconolactonase (EC 3.1.1.31) |
| contig_1201 | 1068 | 607 | - | hypothetical protein |
| contig_1202 | 748 | 533 | - | hypothetical protein |
| contig_1203 | 922 | 125 | - | YbbM seven transmembrane helix protein |
| contig_1203 | 1343 | 906 | - | YbbL ABC transporter ATP-binding protein |
| contig_1203 | 1751 | 1596 | - | Stage 0 sporulation regulatory protein |
| contig_1205 | 788 | 378 | - | FIG012576: mutT/nudix family protein |
| contig_1205 | 888 | 760 | - | FIG012576: mutT/nudix family protein |
| contig_1205 | 1564 | 989 | - | FIG007491: hypothetical protein YeeN |
| contig_1207 | 1437 | 838 | - | ATP-binding transport protein NatA |
| contig_1208 | 337 | 2 | - | FIG01225872: hypothetical protein |
| contig_1208 | 569 | 387 | - | Flagellar hook protein FlgE |
| contig_1208 | 1074 | 610 | - | Flagellar hook protein FlgE |
| contig_1208 | 1333 | 1034 | - | Flagellar hook protein FlgE |
| contig_1208 | 1492 | 1358 | - | Flagellar hook protein FlgE |
| contig_1208 | 1616 | 1446 | - | Flagellar hook protein FlgE |
| contig_1208 | 1752 | 1633 | - | Flagellar basal-body rod modification protein FlgD |
| contig_1208 | 2041 | 1784 | - | Flagellar basal-body rod modification protein FlgD |
| contig_1208 | 2216 | 2052 | - | Flagellar basal-body rod modification protein FlgD |
| contig_1209 | 944 | 789 | - | glycosyl transferase, group 1 |
| contig_121 | 142 | 960 | + | Malate Na(+) symporter |
| contig_121 | 960 | 1085 | + | Malate Na(+) symporter |
| contig_1210 | 407 | 553 | + | hypothetical protein |
| contig_1210 | 1711 | 1115 | - | Transposase and inactivated derivatives-like protein |
| contig_1211 | 222 | 524 | + | Deoxyribose-phosphate aldolase (EC 4.1.2.4) |
| contig_1211 | 506 | 628 | + | Deoxyribose-phosphate aldolase (EC 4.1.2.4) |
| contig_1212 | 426 | 253 | - | FIG01228076: hypothetical protein |
| contig_1212 | 743 | 1168 | + | Pantothenate:Na+ symporter (TC 2.A.21.1.1) |
| contig_1213 | 449 | 36 | - | transcriptional regulator, MerR family |
| contig_1213 | 1128 | 403 | - | Transcriptional regulator, MerR family |
| contig_1214 | 1109 | 363 | - | Replicative DNA helicase (DnaB) (EC 3.6.4.12) |
| contig_1215 | 241 | 83 | - | hypothetical protein |
| contig_1215 | 610 | 353 | - | hypothetical protein |
| contig_1215 | 595 | 852 | + | SOS-response repressor and protease LexA (EC 3.4.21.88) |
| contig_1215 | 821 | 1087 | + | SOS-response repressor and protease LexA (EC 3.4.21.88) |
| contig_1215 | 1104 | 1403 | + | SOS-response repressor and protease LexA (EC 3.4.21.88) |
| contig_1216 | 437 | 147 | - | ATPase involved in DNA repair |
| contig_1216 | 651 | 418 | - | ATPase involved in DNA repair |
| contig_1217 | 316 | 80 | - | transcriptional regulator, MerR family |
| contig_1217 | 425 | 306 | - | transcriptional regulator, MerR family |
| contig_1217 | 868 | 431 | - | transcriptional regulator, MerR family |
| contig_1218 | 807 | 649 | - | Alpha/beta hydrolase |
| contig_1219 | 192 | 428 | + | ABC transporter, permease protein |
| contig_1219 | 559 | 1080 | + | ABC transporter, permease protein |
| contig_1219 | 1198 | 1569 | + | ABC transporter, permease protein |
| contig_1219 | 1621 | 1827 | + | ABC transporter, ATP-binding protein |
| contig_1219 | 1874 | 2068 | + | ABC transporter, ATP-binding protein |
| contig_1219 | 2040 | 2204 | + | ABC transporter, ATP-binding protein |
| contig_1219 | 2703 | 2521 | - | FIG01227055: hypothetical protein |
| contig_122 | 492 | 97 | - | Transcriptional regulator, MerR family |
| contig_122 | 923 | 537 | - | Transcriptional regulator, MerR family |
| contig_122 | 1217 | 966 | - | FIG01226506: hypothetical protein |
| contig_122 | 1641 | 1363 | - | FIG01226506: hypothetical protein |
| contig_122 | 1997 | 1608 | - | FIG01226506: hypothetical protein |
| contig_1220 | 324 | 184 | - | hypothetical protein |
| contig_1220 | 669 | 502 | - | Alkaline phosphatase (EC 3.1.3.1) |
| contig_1221 | 194 | 592 | + | Methylthioribulose-1-phosphate dehydratase (EC 4.2.1.109) |
| contig_1221 | 570 | 698 | + | 1,2-dihydroxy-3-keto-5-methylthiopentene dioxygenase (EC 1.13.11.54) |
| contig_1221 | 819 | 706 | - | hypothetical protein |
| contig_1221 | 1379 | 1654 | + | FIG01225975: hypothetical protein |
| contig_1221 | 2031 | 1693 | - | FIG01225420: hypothetical protein |
| contig_1221 | 2769 | 2032 | - | 2-hydroxy-3-oxopropionate reductase (EC 1.1.1.60) |
| contig_1221 | 2911 | 2753 | - | 2-hydroxy-3-oxopropionate reductase (EC 1.1.1.60) |
| contig_1221 | 3438 | 2983 | - | penicillin-binding protein, putative |
| contig_1222 | 86 | 373 | + | Thioredoxin |
| contig_1222 | 936 | 463 | - | FIG01228285: hypothetical protein |
| contig_1222 | 1650 | 1123 | - | Undecaprenyl-diphosphatase (EC 3.6.1.27) |
| contig_1222 | 1890 | 1750 | - | Undecaprenyl-diphosphatase (EC 3.6.1.27) |
| contig_1223 | 135 | 275 | + | hypothetical protein |
| contig_1223 | 340 | 849 | + | 3-oxoacyl-[acyl-carrier protein] reductase (EC 1.1.1.100) |
| contig_1223 | 1657 | 905 | - | Maltose/maltodextrin transport ATP-binding protein MalK (EC 3.6.3.19) |
| contig_1223 | 2006 | 1617 | - | Multiple sugar ABC transporter, ATP-binding protein |
| contig_1223 | 2269 | 2775 | + | Oligo-1,6-glucosidase (EC 3.2.1.10) |
| contig_1223 | 2847 | 3434 | + | Oligo-1,6-glucosidase (EC 3.2.1.10) |
| contig_1223 | 3427 | 3627 | + | Oligo-1,6-glucosidase (EC 3.2.1.10) |
| contig_1223 | 3754 | 3933 | + | Oligo-1,6-glucosidase (EC 3.2.1.10) |
| contig_1223 | 4999 | 4238 | - | Neopullulanase (EC 3.2.1.135) |
| contig_1223 | 5687 | 4953 | - | Neopullulanase (EC 3.2.1.135) |
| contig_1224 | 327 | 476 | + | hypothetical protein |
| contig_1224 | 1183 | 1815 | + | FIG01226365: hypothetical protein |
| contig_1224 | 1894 | 2280 | + | FIG01226365: hypothetical protein |
| contig_1224 | 2456 | 2608 | + | FIG01226099: hypothetical protein |
| contig_1224 | 3251 | 2901 | - | internalin, putative |
| contig_1225 | 459 | 310 | - | Pantothenate kinase type II, eukaryotic (EC 2.7.1.33) |
| contig_1225 | 1046 | 711 | - | Pantothenate kinase type II, eukaryotic (EC 2.7.1.33) |
| contig_1225 | 1433 | 1215 | - | 3-hydroxyacyl-[acyl-carrier-protein] dehydratase, FabZ form (EC 4.2.1.59) |
| contig_1226 | 98 | 484 | + | Integral membrane protein |
| contig_1226 | 1324 | 479 | - | sensory box/GGDEF family protein |
| contig_1226 | 1509 | 1279 | - | sensory box/GGDEF family protein |
| contig_1226 | 3268 | 1526 | - | sensory box/GGDEF family protein |
| contig_1226 | 3537 | 3905 | + | NADH ubiquinone oxidoreductase chain A (EC 1.6.5.3) |
| contig_1226 | 3896 | 4414 | + | NADH-ubiquinone oxidoreductase chain B (EC 1.6.5.3) |
| contig_1226 | 4411 | 4578 | + | NADH-ubiquinone oxidoreductase chain C (EC 1.6.5.3) |
| contig_1228 | 568 | 284 | - | Chemotaxis regulator - transmits chemoreceptor signals to flagelllar motor components CheY |
| contig_1228 | 962 | 531 | - | Chemotaxis regulator - transmits chemoreceptor signals to flagelllar motor components CheY |
| contig_1228 | 1154 | 981 | - | Ribonucleotide reductase of class III (anaerobic), activating protein (EC 1.97.1.4) |
| contig_1228 | 1432 | 1133 | - | Ribonucleotide reductase of class III (anaerobic), activating protein (EC 1.97.1.4) |
| contig_1228 | 2544 | 1429 | - | Ribonucleotide reductase of class III (anaerobic), large subunit (EC 1.17.4.2) |
| contig_1228 | 3289 | 2582 | - | Ribonucleotide reductase of class III (anaerobic), large subunit (EC 1.17.4.2) |
| contig_1228 | 3515 | 4111 | + | Acyl-phosphate:glycerol-3-phosphate O-acyltransferase PlsY |
| contig_1228 | 4221 | 4421 | + | FIG01226527: hypothetical protein |
| contig_1229 | 194 | 358 | + | FIG01226789: hypothetical protein |
| contig_123 | 857 | 156 | - | Aspartate aminotransferase (EC 2.6.1.1) |
| contig_123 | 1219 | 824 | - | Aspartate aminotransferase (EC 2.6.1.1) |
| contig_123 | 1337 | 1588 | + | DUF1871 domain-containing protein |
| contig_123 | 2393 | 1620 | - | Hydrolase, alpha/beta fold family |
| contig_123 | 2692 | 2570 | - | Glyoxylate reductase (EC 1.1.1.79) / Hydroxypyruvate reductase (EC 1.1.1.81) / 2-ketoaldonate reductase, broad specificity (EC 1.1.1.215) (EC 1.1.1.-) |
| contig_123 | 3563 | 2652 | - | Glyoxylate reductase (EC 1.1.1.79) / Hydroxypyruvate reductase (EC 1.1.1.81) / 2-ketoaldonate reductase, broad specificity (EC 1.1.1.215) (EC 1.1.1.-) |
| contig_1231 | 318 | 163 | - | Ferrochelatase, protoheme ferro-lyase (EC 4.99.1.1) |
| contig_1231 | 676 | 296 | - | Ferrochelatase, protoheme ferro-lyase (EC 4.99.1.1) |
| contig_1231 | 847 | 734 | - | Ferrochelatase, protoheme ferro-lyase (EC 4.99.1.1) |
| contig_1232 | 480 | 34 | - | FIG01226652: hypothetical protein |
| contig_1233 | 359 | 192 | - | hypothetical protein |
| contig_1233 | 832 | 506 | - | hypothetical protein |
| contig_1233 | 1211 | 1738 | + | Site-specific recombinase XerD |
| contig_1233 | 1830 | 2030 | + | hypothetical protein |
| contig_1234 | 322 | 158 | - | Amino acid permease |
| contig_1234 | 632 | 435 | - | Amino acid permease |
| contig_1234 | 1066 | 1194 | + | FIG01226446: hypothetical protein |
| contig_1234 | 1526 | 1251 | - | 2-dehydropantoate 2-reductase (EC 1.1.1.169) |
| contig_1234 | 1938 | 1534 | - | 2-dehydropantoate 2-reductase (EC 1.1.1.169) |
| contig_1234 | 2164 | 1991 | - | 2-dehydropantoate 2-reductase (EC 1.1.1.169) |
| contig_1234 | 2372 | 2602 | + | FIG01226212: hypothetical protein |
| contig_1234 | 3318 | 2668 | - | Microbial collagenase (EC 3.4.24.3) |
| contig_1234 | 3543 | 3418 | - | Microbial collagenase (EC 3.4.24.3) |
| contig_1234 | 3675 | 3547 | - | Microbial collagenase (EC 3.4.24.3) |
| contig_1234 | 4079 | 3744 | - | Microbial collagenase (EC 3.4.24.3) |
| contig_1234 | 4387 | 4133 | - | Microbial collagenase (EC 3.4.24.3) |
| contig_1234 | 4669 | 4463 | - | Microbial collagenase (EC 3.4.24.3) |
| contig_1234 | 5167 | 4694 | - | Microbial collagenase (EC 3.4.24.3) |
| contig_1234 | 5603 | 5160 | - | Microbial collagenase (EC 3.4.24.3) |
| contig_1235 | 13 | 726 | + | Immune inhibitor A, metalloprotease (EC 3.4.24.-) |
| contig_1235 | 1877 | 942 | - | multidrug resistance protein, putative |
| contig_1235 | 2047 | 1904 | - | multidrug resistance protein, putative |
| contig_1236 | 4 | 144 | + | FIG001621: Zinc protease |
| contig_1236 | 219 | 539 | + | FIG001621: Zinc protease |
| contig_1236 | 540 | 872 | + | FIG009210: peptidase, M16 family |
| contig_1237 | 193 | 348 | + | rRNA small subunit methyltransferase H |
| contig_1237 | 566 | 697 | + | rRNA small subunit methyltransferase H |
| contig_1237 | 807 | 959 | + | rRNA small subunit methyltransferase H |
| contig_1237 | 982 | 1128 | + | rRNA small subunit methyltransferase H |
| contig_1237 | 1217 | 1507 | + | Cell division protein FtsL |
| contig_1237 | 1675 | 2112 | + | Penicillin-binding protein 2B |
| contig_1237 | 2272 | 2556 | + | Penicillin-binding protein 2B |
| contig_1237 | 2634 | 2870 | + | Penicillin-binding protein 2B |
| contig_1237 | 2851 | 3687 | + | Penicillin-binding protein 2B |
| contig_1237 | 3769 | 4029 | + | Cell division protein FtsI [Peptidoglycan synthetase] (EC 2.4.1.129) / Stage V sporulation protein D (Sporulation-specific penicillin-binding protein) |
| contig_1237 | 4073 | 4825 | + | Cell division protein FtsI [Peptidoglycan synthetase] (EC 2.4.1.129) / Stage V sporulation protein D (Sporulation-specific penicillin-binding protein) |
| contig_1238 | 21 | 173 | + | Ribosome-binding factor A |
| contig_1238 | 137 | 271 | + | Ribosome-binding factor A |
| contig_1238 | 358 | 1059 | + | tRNA pseudouridine synthase B (EC 4.2.1.70) |
| contig_1238 | 1019 | 1156 | + | tRNA pseudouridine synthase B (EC 4.2.1.70) |
| contig_1238 | 1147 | 1284 | + | tRNA pseudouridine synthase B (EC 4.2.1.70) |
| contig_1238 | 1328 | 1567 | + | Riboflavin kinase (EC 2.7.1.26) / FMN adenylyltransferase (EC 2.7.7.2) |
| contig_1238 | 1609 | 1791 | + | Riboflavin kinase (EC 2.7.1.26) / FMN adenylyltransferase (EC 2.7.7.2) |
| contig_1238 | 1743 | 1964 | + | Riboflavin kinase (EC 2.7.1.26) / FMN adenylyltransferase (EC 2.7.7.2) |
| contig_1238 | 1915 | 2301 | + | Riboflavin kinase (EC 2.7.1.26) / FMN adenylyltransferase (EC 2.7.7.2) |
| contig_1238 | 2402 | 2671 | + | SSU ribosomal protein S15p (S13e) |
| contig_124 | 1125 | 106 | - | Flagellar motor switch protein FliM |
| contig_1240 | 143 | 556 | + | Macrolide glycosyltransferase (EC 2.4.1.-) |
| contig_1240 | 564 | 953 | + | Macrolide glycosyltransferase (EC 2.4.1.-) |
| contig_1240 | 931 | 1254 | + | Macrolide glycosyltransferase (EC 2.4.1.-) |
| contig_1240 | 1501 | 2157 | + | Phosphoenolpyruvate synthase (EC 2.7.9.2) |
| contig_1240 | 2115 | 2255 | + | Phosphoenolpyruvate synthase (EC 2.7.9.2) |
| contig_1241 | 837 | 88 | - | FIG145533: Methyltransferase (EC 2.1.1.-) |
| contig_1241 | 1190 | 834 | - | Ribosomal silencing factor RsfA (former Iojap) |
| contig_1241 | 1648 | 1187 | - | Hydrolase (HAD superfamily), YqeK |
| contig_1243 | 242 | 475 | + | acetyltransferase, GNAT family |
| contig_1243 | 645 | 1856 | + | Multidrug-efflux transporter, major facilitator superfamily (MFS) (TC 2.A.1) |
| contig_1243 | 2012 | 1893 | - | FIG074102: hypothetical protein |
| contig_1243 | 2504 | 2034 | - | FIG074102: hypothetical protein |
| contig_1243 | 2655 | 2488 | - | FIG074102: hypothetical protein |
| contig_1244 | 305 | 51 | - | Chromosome initiation inhibitor |
| contig_1246 | 198 | 617 | + | CBS domain protein |
| contig_1247 | 348 | 31 | - | FIG001454: Transglutaminase-like enzymes, putative cysteine proteases |
| contig_1247 | 544 | 335 | - | FIG001454: Transglutaminase-like enzymes, putative cysteine proteases |
| contig_1248 | 193 | 11 | - | Trp repressor binding protein |
| contig_1248 | 527 | 165 | - | Trp repressor binding protein |
| contig_1248 | 900 | 532 | - | Transcriptional regulator, MerR family |
| contig_1248 | 1203 | 952 | - | LysR family transcriptional regulator YeiE |
| contig_1248 | 1849 | 1181 | - | LysR family transcriptional regulator YeiE |
| contig_1248 | 1970 | 2191 | + | Putative membrane protein YeiH |
| contig_1250 | 878 | 81 | - | Carboxynorspermidine dehydrogenase, putative (EC 1.1.1.-) |
| contig_1250 | 1718 | 1041 | - | 2-aminoethylphosphonate:pyruvate aminotransferase (EC 2.6.1.37) |
| contig_1250 | 2139 | 1672 | - | 2-aminoethylphosphonate:pyruvate aminotransferase (EC 2.6.1.37) |
| contig_1250 | 2364 | 2155 | - | Phosphonoacetaldehyde hydrolase (EC 3.11.1.1) |
| contig_1250 | 2950 | 2336 | - | Phosphonoacetaldehyde hydrolase (EC 3.11.1.1) |
| contig_1251 | 436 | 323 | - | hypothetical protein |
| contig_1252 | 783 | 652 | - | hypothetical protein |
| contig_1253 | 706 | 503 | - | Multidrug resistance ABC transporter ATP-binding and permease protein |
| contig_1253 | 909 | 676 | - | Multidrug resistance ABC transporter ATP-binding and permease protein |
| contig_1253 | 1301 | 960 | - | ABC transporter, ATP-binding/permease protein |
| contig_1253 | 1671 | 1357 | - | Multidrug resistance ABC transporter ATP-binding and permease protein |
| contig_1253 | 2053 | 1886 | - | Lipid A export ATP-binding/permease protein MsbA |
| contig_1253 | 2225 | 2112 | - | Lipid A export ATP-binding/permease protein MsbA |
| contig_1253 | 2932 | 2495 | - | Lipid A export ATP-binding/permease protein MsbA |
| contig_1253 | 3214 | 3032 | - | Multidrug resistance ABC transporter ATP-binding and permease protein |
| contig_1254 | 922 | 224 | - | Aspartokinase (EC 2.7.2.4) |
| contig_1254 | 1207 | 1022 | - | Aspartokinase (EC 2.7.2.4) |
| contig_1254 | 1447 | 1211 | - | Aspartokinase (EC 2.7.2.4) |
| contig_1255 | 659 | 204 | - | DNA topoisomerase III (EC 5.99.1.2) |
| contig_1256 | 205 | 642 | + | Polysaccharide deacetylase |
| contig_1256 | 690 | 1568 | + | DNA-3-methyladenine glycosylase II (EC 3.2.2.21) |
| contig_1257 | 349 | 645 | + | Tellurium resistance protein TerD |
| contig_1258 | 34 | 168 | + | Transcriptional regulator, MarR family |
| contig_1258 | 205 | 327 | + | hypothetical protein |
| contig_1258 | 433 | 918 | + | multidrug resistance protein, putative |
| contig_1258 | 1024 | 1266 | + | multidrug resistance protein, putative |
| contig_1258 | 1461 | 1655 | + | multidrug resistance protein, putative |
| contig_1258 | 2599 | 1790 | - | Chromosome initiation inhibitor |
| contig_1259 | 564 | 403 | - | Two-component response regulator, controling glutamine utilization |
| contig_1259 | 1286 | 567 | - | Two-component sensor histidine kinase, controling glutamine utilization |
| contig_1260 | 222 | 58 | - | FIG01230943: hypothetical protein |
| contig_1260 | 925 | 377 | - | Serine transporter |
| contig_1260 | 1029 | 880 | - | Serine transporter |
| contig_1260 | 1553 | 1086 | - | Serine transporter |
| contig_1262 | 280 | 432 | + | hypothetical protein |
| contig_1262 | 607 | 720 | + | hypothetical protein |
| contig_1263 | 317 | 120 | - | FIG01226799: hypothetical protein |
| contig_1263 | 957 | 319 | - | Catalyzes the cleavage of p-aminobenzoyl-glutamate to p-aminobenzoate and glutamate, subunit A |
| contig_1263 | 1495 | 935 | - | Catalyzes the cleavage of p-aminobenzoyl-glutamate to p-aminobenzoate and glutamate, subunit A |
| contig_1263 | 2337 | 1627 | - | FIG01225665: hypothetical protein |
| contig_1264 | 42 | 290 | + | Stage V sporulation protein AC (SpoVAC) |
| contig_1264 | 291 | 437 | + | Stage V sporulation protein AD (SpoVAD) |
| contig_1265 | 42 | 290 | + | Stage V sporulation protein AC (SpoVAC) |
| contig_1266 | 278 | 538 | + | acetyltransferase, GNAT family |
| contig_1266 | 550 | 687 | + | Phenazine biosynthesis protein PhzF like |
| contig_1266 | 731 | 880 | + | Phenazine biosynthesis protein PhzF like |
| contig_1266 | 913 | 1299 | + | Phenazine biosynthesis protein PhzF like |
| contig_1266 | 1277 | 1585 | + | Phenazine biosynthesis protein PhzF like |
| contig_1267 | 214 | 396 | + | FIG01227238: hypothetical protein |
| contig_1267 | 537 | 424 | - | Formate efflux transporter (TC 2.A.44 family) |
| contig_1268 | 212 | 1012 | + | hydrolase, haloacid dehalogenase-like family |
| contig_1268 | 1199 | 1083 | - | Predicted transcriptional regulator of N-Acetylglucosamine utilization, GntR family |
| contig_1268 | 1761 | 1162 | - | Predicted transcriptional regulator of N-Acetylglucosamine utilization, GntR family |
| contig_1268 | 2032 | 1745 | - | Glucosamine-6-phosphate deaminase (EC 3.5.99.6) |
| contig_1268 | 2438 | 2325 | - | Glucosamine-6-phosphate deaminase (EC 3.5.99.6) |
| contig_1269 | 663 | 355 | - | Spore germination protein GerSB |
| contig_1269 | 1314 | 715 | - | Spore germination protein GerSA |
| contig_1269 | 1657 | 1331 | - | Spore germination protein GerSA |
| contig_1269 | 2307 | 2585 | + | FIG01228016: hypothetical protein |
| contig_1269 | 3008 | 2646 | - | Spore germination protein GerN (inosine-dependent germination), Na+/H+ antiporter |
| contig_1269 | 3613 | 3038 | - | Spore germination protein GerN (inosine-dependent germination), Na+/H+ antiporter |
| contig_1269 | 4166 | 4348 | + | Ribonuclease J2 (endoribonuclease in RNA processing) |
| contig_127 | 267 | 455 | + | hypothetical protein |
| contig_127 | 687 | 851 | + | hypothetical protein |
| contig_127 | 1092 | 1709 | + | FIG01226365: hypothetical protein |
| contig_1271 | 878 | 1111 | + | hypothetical protein |
| contig_1272 | 1075 | 527 | - | hypothetical protein |
| contig_1273 | 486 | 88 | - | Glycerate kinase (EC 2.7.1.31) |
| contig_1273 | 1000 | 572 | - | FIG01226693: hypothetical protein |
| contig_1273 | 1128 | 1580 | + | Leucine-responsive regulatory protein, regulator for leucine (or lrp) regulon and high-affinity branched-chain amino acid transport system |
| contig_1273 | 1686 | 1868 | + | FIG01226885: hypothetical protein |
| contig_1273 | 2035 | 1898 | - | transcriptional regulator, AbrB family |
| contig_1273 | 2277 | 2050 | - | transcriptional regulator, AbrB family |
| contig_1273 | 2577 | 2389 | - | transcriptional regulator, AbrB family |
| contig_1275 | 12 | 125 | + | Tn7-like transposition protein B |
| contig_1276 | 274 | 1077 | + | Transposon Tn7 transposition protein tnsC |
| contig_1277 | 720 | 535 | - | Putative CDP-glycerol:glycerophosphate glycerophosphotransferase (EC 2.7.8.-) |
| contig_1277 | 1274 | 735 | - | Putative CDP-glycerol:glycerophosphate glycerophosphotransferase (EC 2.7.8.-) |
| contig_1277 | 1551 | 1360 | - | Putative CDP-glycerol:glycerophosphate glycerophosphotransferase (EC 2.7.8.-) |
| contig_1277 | 1747 | 1574 | - | Glycosyltransferase (EC 2.4.1.-) |
| contig_1278 | 213 | 31 | - | Zwittermicin A resistance protein ZmaR |
| contig_1278 | 332 | 210 | - | Zwittermicin A resistance protein ZmaR |
| contig_1278 | 534 | 304 | - | Zwittermicin A resistance protein ZmaR |
| contig_1278 | 860 | 1300 | + | EMG2 protein |
| contig_1278 | 1586 | 1377 | - | FIG01229360: hypothetical protein |
| contig_1278 | 2163 | 1777 | - | FIG01226434: hypothetical protein |
| contig_1278 | 2497 | 2171 | - | FIG01226434: hypothetical protein |
| contig_1279 | 898 | 74 | - | amino acid permease family protein |
| contig_1279 | 1297 | 941 | - | amino acid permease family protein |
| contig_128 | 389 | 505 | + | hypothetical protein |
| contig_128 | 519 | 821 | + | FIG01227918: hypothetical protein |
| contig_128 | 1482 | 919 | - | forespore-specific protein, putative |
| contig_1280 | 592 | 263 | - | Conserved protein YqhG |
| contig_1280 | 1106 | 579 | - | Superfamily II DNA/RNA helicases, SNF2 family |
| contig_1281 | 304 | 137 | - | Superfamily II DNA/RNA helicases, SNF2 family |
| contig_1282 | 7 | 795 | + | GTP-binding protein EngA |
| contig_1282 | 867 | 1088 | + | GTP-binding protein EngA |
| contig_1282 | 1107 | 1469 | + | Glycerol-3-phosphate dehydrogenase [NAD(P)+] (EC 1.1.1.94) |
| contig_1282 | 1429 | 1716 | + | Glycerol-3-phosphate dehydrogenase [NAD(P)+] (EC 1.1.1.94) |
| contig_1283 | 203 | 793 | + | permease, putative |
| contig_1283 | 913 | 1089 | + | permease, putative |
| contig_1283 | 1079 | 1711 | + | Chloramphenicol acetyltransferase (EC 2.3.1.28) |
| contig_1284 | 313 | 113 | - | FIG01225966: hypothetical protein |
| contig_1286 | 100 | 915 | + | Formate efflux transporter (TC 2.A.44 family) |
| contig_1288 | 354 | 160 | - | Hypothetical protein DUF901, similar to C-terminal domain of ribosome protection-type Tc-resistance proteins |
| contig_1289 | 41 | 241 | + | L-Proline/Glycine betaine transporter ProP |
| contig_1289 | 207 | 458 | + | L-Proline/Glycine betaine transporter ProP |
| contig_1289 | 977 | 852 | - | FIG01225698: hypothetical protein |
| contig_1289 | 1704 | 1066 | - | FIG01225698: hypothetical protein |
| contig_1289 | 2017 | 1679 | - | FIG01225947: hypothetical protein |
| contig_129 | 294 | 530 | + | Molybdenum cofactor biosynthesis protein MoaE |
| contig_129 | 490 | 711 | + | Molybdenum cofactor biosynthesis protein MoaD |
| contig_129 | 807 | 1217 | + | FIG01227532: hypothetical protein |
| contig_129 | 1264 | 2031 | + | glucose uptake protein |
| contig_129 | 1985 | 2122 | + | glucose uptake protein |
| contig_129 | 2136 | 2396 | + | Glucose 1-dehydrogenase (EC 1.1.1.47) |
| contig_129 | 2447 | 2692 | + | Glucose 1-dehydrogenase (EC 1.1.1.47) |
| contig_1290 | 367 | 1758 | + | glycolate oxidase, subunit GlcD, putative |
| contig_1290 | 1763 | 1939 | + | Bile acid sodium symporter |
| contig_1292 | 531 | 181 | - | CDS_ID OB2928 |
| contig_1292 | 925 | 1461 | + | Transposase |
| contig_1292 | 1500 | 1655 | + | Transposase |
| contig_1293 | 222 | 347 | + | Phosphocarrier protein of PTS system |
| contig_1296 | 638 | 33 | - | Cytochrome c-type biogenesis protein CcsA/ResC |
| contig_1296 | 967 | 701 | - | Cytochrome c-type biogenesis protein CcsA/ResC |
| contig_1296 | 1802 | 1209 | - | Cytochrome c-type biogenesis protein Ccs1/ResB |
| contig_1296 | 2836 | 2129 | - | Cytochrome c-type biogenesis protein Ccs1/ResB |
| contig_1297 | 55 | 285 | + | Trehalose-6-phosphate hydrolase (EC 3.2.1.93) |
| contig_1297 | 955 | 770 | - | Spore germination protein GerKC |
| contig_1298 | 14 | 673 | + | N-acetylglucosamine-6-phosphate deacetylase (EC 3.5.1.25) |
| contig_1298 | 678 | 1523 | + | Tagatose 1,6-bisphosphate aldolase (EC 4.1.2.40) |
| contig_130 | 876 | 538 | - | Cytoplasmic axial filament protein CafA and Ribonuclease G (EC 3.1.4.-) |
| contig_130 | 1117 | 977 | - | Cytoplasmic axial filament protein CafA and Ribonuclease G (EC 3.1.4.-) |
| contig_1300 | 424 | 143 | - | Wall-associated protein precursor |
| contig_1301 | 192 | 10 | - | FIG01226514: hypothetical protein |
| contig_1301 | 304 | 594 | + | FIG01227372: hypothetical protein |
| contig_1302 | 213 | 19 | - | D-alanyl-D-alanine carboxypeptidase (EC 3.4.16.4) |
| contig_1302 | 1148 | 435 | - | sensor histidine kinase |
| contig_1302 | 1503 | 1120 | - | sensor histidine kinase |
| contig_1302 | 1666 | 1535 | - | Phosphate regulon transcriptional regulatory protein PhoB (SphR) |
| contig_1302 | 1956 | 1663 | - | Phosphate regulon transcriptional regulatory protein PhoB (SphR) |
| contig_1302 | 2236 | 1979 | - | Phosphate regulon transcriptional regulatory protein PhoB (SphR) |
| contig_1303 | 431 | 267 | - | Signal peptidase I (EC 3.4.21.89) |
| contig_1303 | 598 | 455 | - | Cytoplasmic copper homeostasis protein CutC |
| contig_1303 | 713 | 564 | - | hypothetical protein |
| contig_1303 | 847 | 716 | - | hypothetical protein |
| contig_1303 | 1073 | 834 | - | Cytoplasmic copper homeostasis protein CutC |
| contig_1304 | 119 | 403 | + | Protein ecsB |
| contig_1304 | 822 | 959 | + | FIG01228419: hypothetical protein |
| contig_1305 | 1013 | 375 | - | membrane protein, putative |
| contig_1305 | 1669 | 1112 | - | Transcriptional regulator, TetR family |
| contig_1305 | 2395 | 1673 | - | metallo-beta-lactamase family protein |
| contig_1306 | 187 | 456 | + | Acetolactate synthase large subunit (EC 2.2.1.6) |
| contig_1306 | 446 | 697 | + | Acetolactate synthase large subunit (EC 2.2.1.6) |
| contig_1306 | 694 | 924 | + | hypothetical protein |
| contig_1307 | 133 | 423 | + | FIG01225669: hypothetical protein |
| contig_1307 | 1150 | 506 | - | Fumarate hydratase class II (EC 4.2.1.2) |
| contig_1307 | 1280 | 1161 | - | Fumarate hydratase class II (EC 4.2.1.2) |
| contig_1308 | 33 | 698 | + | Spore germination protein GerYA |
| contig_1308 | 767 | 1567 | + | Spore germination protein GerYC |
| contig_1308 | 1794 | 2180 | + | Spore germination protein GerYB |
| contig_1308 | 2341 | 2916 | + | Spore germination protein GerYB |
| contig_1308 | 2955 | 3467 | + | Phosphonate ABC transporter phosphate-binding periplasmic component (TC 3.A.1.9.1) |
| contig_1308 | 3584 | 3730 | + | FIG01225983: hypothetical protein |
| contig_1308 | 3886 | 3719 | - | hypothetical protein |
| contig_1308 | 4003 | 4422 | + | Disulfide bond formation protein (EC 1.8.4.-), BdbC-like |
| contig_1308 | 4403 | 4531 | + | hypothetical protein |
| contig_1308 | 4503 | 4853 | + | Thioredoxin |
| contig_1308 | 4926 | 5141 | + | FIG01228236: hypothetical protein |
| contig_1308 | 5299 | 5159 | - | hypothetical protein |
| contig_1308 | 5421 | 5284 | - | membrane protein, putative |
| contig_1308 | 6294 | 5533 | - | Protein erfK/srfK |
| contig_1308 | 7240 | 6362 | - | oxidoreductase, short chain dehydrogenase/reductase family |
| contig_1309 | 11 | 154 | + | Transcriptional regulator, PadR family |
| contig_1309 | 111 | 368 | + | Transcriptional regulator, PadR family |
| contig_1309 | 418 | 1092 | + | drug resistance transporter, EmrB/QacA family |
| contig_1309 | 1100 | 1240 | + | drug resistance transporter, EmrB/QacA family |
| contig_1309 | 1326 | 1520 | + | drug resistance transporter, EmrB/QacA family |
| contig_1309 | 2022 | 2147 | + | drug resistance transporter, EmrB/QacA family |
| contig_131 | 397 | 519 | + | Acetyl-coenzyme A carboxyl transferase alpha chain (EC 6.4.1.2) |
| contig_131 | 661 | 825 | + | Acetyl-coenzyme A carboxyl transferase alpha chain (EC 6.4.1.2) |
| contig_1310 | 780 | 298 | - | DNA polymerase III alpha subunit (EC 2.7.7.7) |
| contig_1310 | 944 | 831 | - | DNA polymerase III alpha subunit (EC 2.7.7.7) |
| contig_1310 | 1251 | 934 | - | DNA polymerase III alpha subunit (EC 2.7.7.7) |
| contig_1311 | 585 | 46 | - | hypothetical protein |
| contig_1311 | 1742 | 1512 | - | FIG01236150: hypothetical protein |
| contig_1311 | 2197 | 1763 | - | FIG01239297: hypothetical protein |
| contig_1312 | 459 | 719 | + | FIG01226706: hypothetical protein |
| contig_1313 | 863 | 375 | - | Immune inhibitor A precursor |
| contig_1314 | 245 | 697 | + | Alkanesulfonates ABC transporter ATP-binding protein |
| contig_1316 | 122 | 346 | + | Isopentenyl-diphosphate delta-isomerase, FMN-dependent (EC 5.3.3.2) |
| contig_1316 | 318 | 650 | + | Isopentenyl-diphosphate delta-isomerase, FMN-dependent (EC 5.3.3.2) |
| contig_1316 | 641 | 988 | + | Isopentenyl-diphosphate delta-isomerase, FMN-dependent (EC 5.3.3.2) |
| contig_1316 | 1059 | 1184 | + | Isopentenyl-diphosphate delta-isomerase, FMN-dependent (EC 5.3.3.2) |
| contig_1318 | 84 | 227 | + | acetyltransferase, GNAT family |
| contig_1318 | 234 | 695 | + | FIG01230629: hypothetical protein |
| contig_1318 | 676 | 795 | + | FIG01230629: hypothetical protein |
| contig_1318 | 866 | 1051 | + | acetyltransferase, GNAT family |
| contig_1319 | 472 | 194 | - | 3-oxoacyl-[acyl-carrier protein] reductase paralog (EC 1.1.1.100) |
| contig_1319 | 672 | 493 | - | 3-oxoacyl-[acyl-carrier protein] reductase paralog (EC 1.1.1.100) |
| contig_1319 | 988 | 683 | - | 3-oxoacyl-[acyl-carrier protein] reductase paralog (EC 1.1.1.100) |
| contig_1319 | 1229 | 1053 | - | Hydroxyacylglutathione hydrolase (EC 3.1.2.6) |
| contig_1319 | 1498 | 1226 | - | Hydroxyacylglutathione hydrolase (EC 3.1.2.6) |
| contig_132 | 34 | 150 | + | FIG01225604: hypothetical protein |
| contig_132 | 322 | 176 | - | FIG01226854: hypothetical protein |
| contig_132 | 452 | 333 | - | FIG01226854: hypothetical protein |
| contig_132 | 768 | 466 | - | FIG01225318: hypothetical protein |
| contig_132 | 1922 | 927 | - | GTP-binding protein TypA/BipA |
| contig_132 | 2638 | 1937 | - | GTP-binding protein TypA/BipA |
| contig_1320 | 898 | 194 | - | Predicted dinucleotide-binding enzymes |
| contig_1320 | 1824 | 979 | - | Threonine dehydrogenase and related Zn-dependent dehydrogenases |
| contig_1321 | 495 | 259 | - | Chemotaxis protein CheV (EC 2.7.3.-) |
| contig_1321 | 805 | 488 | - | Chemotaxis protein CheV (EC 2.7.3.-) |
| contig_1322 | 214 | 14 | - | Cell envelope-associated transcriptional attenuator LytR-CpsA-Psr, subfamily F2 (as in PMID19099556) |
| contig_1323 | 1182 | 100 | - | Two-component sensor histidine kinase, malate (EC 2.7.3.-) |
| contig_1323 | 1392 | 1261 | - | methyl-accepting chemotaxis protein |
| contig_1324 | 1053 | 289 | - | Carboxylic ester hydrolase |
| contig_1324 | 1459 | 1103 | - | Carboxylic ester hydrolase |
| contig_1325 | 165 | 608 | + | Ribose 5-phosphate isomerase B (EC 5.3.1.6) |
| contig_1325 | 657 | 1247 | + | Hypothetical protein ywlG |
| contig_1325 | 1424 | 2707 | + | Serine hydroxymethyltransferase (EC 2.1.2.1) |
| contig_1327 | 364 | 215 | - | ABC transporter permease protein |
| contig_1328 | 450 | 169 | - | hypothetical protein |
| contig_1329 | 241 | 116 | - | Tn554-related, transposase C |
| contig_133 | 33 | 2507 | + | 1,4-alpha-glucan (glycogen) branching enzyme, GH-13-type (EC 2.4.1.18) |
| contig_133 | 2485 | 2598 | + | hypothetical protein |
| contig_133 | 2627 | 3574 | + | Glycogen biosynthesis protein GlgD, glucose-1-phosphate adenylyltransferase family |
| contig_133 | 3775 | 4209 | + | Glycogen synthase, ADP-glucose transglucosylase (EC 2.4.1.21) |
| contig_133 | 4268 | 4702 | + | Glycogen synthase, ADP-glucose transglucosylase (EC 2.4.1.21) |
| contig_133 | 4668 | 5207 | + | Glycogen synthase, ADP-glucose transglucosylase (EC 2.4.1.21) |
| contig_133 | 5226 | 6752 | + | Glycogen phosphorylase (EC 2.4.1.1) |
| contig_133 | 6784 | 6990 | + | Glycogen phosphorylase (EC 2.4.1.1) |
| contig_133 | 6972 | 7637 | + | Glycogen phosphorylase (EC 2.4.1.1) |
| contig_133 | 8709 | 8017 | - | Lipase (EC 3.1.1.3) |
| contig_133 | 8850 | 9620 | + | transcriptional regulator, merR family |
| contig_133 | 9871 | 9644 | - | DUF124 domain-containing protein |
| contig_133 | 10427 | 9855 | - | DUF124 domain-containing protein |
| contig_133 | 10819 | 11019 | + | Cold shock protein CspD |
| contig_133 | 11414 | 11064 | - | DNA binding protein, DksA/TraR family |
| contig_133 | 11796 | 11374 | - | DNA binding protein, DksA/TraR family |
| contig_133 | 12136 | 11906 | - | 1,4-dihydroxy-2-naphthoate polyprenyltransferase (EC 2.5.1.74) |
| contig_133 | 12860 | 12231 | - | 1,4-dihydroxy-2-naphthoate polyprenyltransferase (EC 2.5.1.74) |
| contig_133 | 13056 | 14363 | + | Menaquinone-specific isochorismate synthase (EC 5.4.4.2) |
| contig_133 | 14446 | 14724 | + | 2-succinyl-5-enolpyruvyl-6-hydroxy-3-cyclohexene-1-carboxylic-acid synthase (EC 2.2.1.9) |
| contig_133 | 14741 | 15406 | + | 2-succinyl-5-enolpyruvyl-6-hydroxy-3-cyclohexene-1-carboxylic-acid synthase (EC 2.2.1.9) |
| contig_133 | 15498 | 16202 | + | 2-succinyl-5-enolpyruvyl-6-hydroxy-3-cyclohexene-1-carboxylic-acid synthase (EC 2.2.1.9) |
| contig_133 | 16199 | 16630 | + | 2-succinyl-6-hydroxy-2,4-cyclohexadiene-1-carboxylate synthase (EC 4.2.99.20) |
| contig_133 | 16651 | 17013 | + | 2-succinyl-6-hydroxy-2,4-cyclohexadiene-1-carboxylate synthase (EC 4.2.99.20) |
| contig_133 | 17083 | 17343 | + | Naphthoate synthase (EC 4.1.3.36) |
| contig_133 | 17417 | 17941 | + | Naphthoate synthase (EC 4.1.3.36) |
| contig_133 | 18067 | 18972 | + | O-succinylbenzoic acid--CoA ligase (EC 6.2.1.26) |
| contig_133 | 18966 | 19514 | + | O-succinylbenzoic acid--CoA ligase (EC 6.2.1.26) |
| contig_133 | 19515 | 20621 | + | O-succinylbenzoate synthase (EC 4.2.1.113) |
| contig_1330 | 1568 | 336 | - | Cell division protein FtsA |
| contig_1330 | 2247 | 1972 | - | Cell division protein FtsQ |
| contig_1330 | 2548 | 2312 | - | Cell division protein FtsQ |
| contig_1331 | 178 | 53 | - | ABC transporter permease protein |
| contig_1332 | 300 | 76 | - | sodium-dependent transporter |
| contig_1332 | 532 | 275 | - | sodium-dependent transporter |
| contig_1332 | 1415 | 534 | - | sodium-dependent transporter |
| contig_1332 | 2221 | 1991 | - | Preprotein translocase secY subunit (TC 3.A.5.1.1) |
| contig_1332 | 2369 | 2079 | - | Preprotein translocase secY subunit (TC 3.A.5.1.1) |
| contig_1333 | 899 | 243 | - | Selenoprotein O and cysteine-containing homologs |
| contig_1334 | 288 | 121 | - | Spore cortex-lytic enzyme, N-acetylglucosaminidase SleL (EC 3.2.1.-) |
| contig_1334 | 481 | 263 | - | Spore cortex-lytic enzyme, N-acetylglucosaminidase SleL (EC 3.2.1.-) |
| contig_1335 | 301 | 441 | + | Methylthioribulose-1-phosphate dehydratase related protein |
| contig_1335 | 494 | 745 | + | Methylthioribulose-1-phosphate dehydratase related protein |
| contig_1336 | 606 | 460 | - | Stage II sporulation protein B |
| contig_1336 | 2077 | 713 | - | Dihydrofolate synthase (EC 6.3.2.12) @ Folylpolyglutamate synthase (EC 6.3.2.17) |
| contig_1337 | 152 | 1465 | + | Anaerobic C4-dicarboxylate transporter |
| contig_1337 | 1994 | 1563 | - | Thiamin-phosphate pyrophosphorylase (EC 2.5.1.3) |
| contig_1339 | 522 | 379 | - | Non-hemolytic enterotoxin A |
| contig_1339 | 1304 | 645 | - | Non-hemolytic enterotoxin A |
| contig_134 | 939 | 118 | - | FIG01229635: hypothetical protein |
| contig_134 | 1766 | 942 | - | FIG01239104: hypothetical protein |
| contig_1341 | 129 | 518 | + | Membrane protein, distant similarity to thiosulphate:quinone oxidoreductase DoxD |
| contig_1341 | 626 | 1237 | + | Nitrilotriacetate monooxygenase component B (EC 1.14.13.-) |
| contig_1341 | 2193 | 1693 | - | FIG01233139: hypothetical protein |
| contig_1341 | 2442 | 2290 | - | FIG01225386: hypothetical protein |
| contig_1344 | 1030 | 155 | - | Transcriptional regulator, MecI family |
| contig_1344 | 1268 | 1125 | - | FIG01228041: hypothetical protein |
| contig_1345 | 74 | 307 | + | FIG01229573: hypothetical protein |
| contig_1345 | 433 | 630 | + | FIG01225957: hypothetical protein |
| contig_1346 | 547 | 1092 | + | FIG01243492: hypothetical protein |
| contig_1347 | 197 | 550 | + | Sodium/glycine symporter GlyP |
| contig_1347 | 644 | 760 | + | Sodium/glycine symporter GlyP |
| contig_1347 | 994 | 1344 | + | alkaline serine protease, subtilase family |
| contig_1347 | 1460 | 2050 | + | alkaline serine protease, subtilase family |
| contig_135 | 221 | 54 | - | FIG01227138: hypothetical protein |
| contig_135 | 517 | 359 | - | Purple acid phosphatase/fibronectin domain protein |
| contig_135 | 984 | 586 | - | Purple acid phosphatase/fibronectin domain protein |
| contig_135 | 1645 | 1049 | - | Purple acid phosphatase/fibronectin domain protein |
| contig_135 | 2802 | 1816 | - | Purple acid phosphatase/fibronectin domain protein |
| contig_1350 | 441 | 313 | - | Methyltransferase (EC 2.1.1.-) |
| contig_1350 | 1057 | 536 | - | Methyltransferase (EC 2.1.1.-) |
| contig_1350 | 1373 | 1230 | - | FIG01238195: hypothetical protein |
| contig_1350 | 1249 | 1527 | + | FIG01225388: hypothetical protein |
| contig_1350 | 2116 | 1601 | - | Putative Xaa-Pro dipeptidyl-peptidase (EC 3.4.14.11) (X-Pro dipeptidyl-peptidase) (X-prolyl-dipeptidyl aminopeptidase) (X-PDAP) |
| contig_1350 | 2316 | 2107 | - | Putative Xaa-Pro dipeptidyl-peptidase (EC 3.4.14.11) (X-Pro dipeptidyl-peptidase) (X-prolyl-dipeptidyl aminopeptidase) (X-PDAP) |
| contig_1350 | 3099 | 2476 | - | Putative Xaa-Pro dipeptidyl-peptidase (EC 3.4.14.11) (X-Pro dipeptidyl-peptidase) (X-prolyl-dipeptidyl aminopeptidase) (X-PDAP) |
| contig_1350 | 3330 | 3142 | - | Putative Xaa-Pro dipeptidyl-peptidase (EC 3.4.14.11) (X-Pro dipeptidyl-peptidase) (X-prolyl-dipeptidyl aminopeptidase) (X-PDAP) |
| contig_1351 | 1715 | 1371 | - | Methionine aminopeptidase (EC 3.4.11.18) |
| contig_1352 | 426 | 31 | - | N-acetylmannosamine kinase (EC 2.7.1.60) |
| contig_1352 | 910 | 401 | - | N-acetylmannosamine kinase (EC 2.7.1.60) |
| contig_1353 | 476 | 162 | - | Dihydrolipoamide dehydrogenase of acetoin dehydrogenase (EC 1.8.1.4) |
| contig_1353 | 941 | 489 | - | Dihydrolipoamide dehydrogenase of acetoin dehydrogenase (EC 1.8.1.4) |
| contig_1354 | 1110 | 643 | - | Lysine 2,3-aminomutase (EC 5.4.3.2) |
| contig_1354 | 1481 | 1347 | - | hypothetical protein |
| contig_1354 | 1879 | 1439 | - | Lysine 2,3-aminomutase (EC 5.4.3.2) |
| contig_1354 | 2425 | 2126 | - | L-lysine aminomutase regulator |
| contig_1354 | 3020 | 2418 | - | L-lysine aminomutase regulator |
| contig_1354 | 3479 | 3075 | - | L-lysine aminomutase regulator |
| contig_1354 | 4094 | 3585 | - | Beta-lysine acetyltransferase (EC 2.3.1.-) |
| contig_1355 | 440 | 6 | - | Rrf2 family transcriptional regulator, group III |
| contig_1355 | 1702 | 593 | - | membrane protein, putative |
| contig_1355 | 2671 | 1787 | - | ABC transporter, ATP-binding protein |
| contig_1356 | 454 | 642 | + | Magnesium and cobalt transport protein CorA |
| contig_1357 | 246 | 19 | - | Cell envelope-associated transcriptional attenuator LytR-CpsA-Psr, subfamily F2 (as in PMID19099556) |
| contig_1358 | 912 | 76 | - | Thiol-activated cytolysin # pneumolysin |
| contig_1358 | 1339 | 896 | - | Thiol-activated cytolysin # perfringolysin O |
| contig_1359 | 231 | 19 | - | 4-hydroxy-tetrahydrodipicolinate synthase (EC 4.3.3.7) |
| contig_1359 | 462 | 313 | - | Aspartokinase (EC 2.7.2.4) |
| contig_136 | 211 | 80 | - | Transcriptional regulator, GntR family |
| contig_136 | 807 | 256 | - | Transcriptional regulator, GntR family |
| contig_136 | 1034 | 807 | - | carbohydrate kinase, PfkB family |
| contig_136 | 1824 | 1003 | - | carbohydrate kinase, PfkB family |
| contig_136 | 2572 | 1817 | - | Protein of unknown function DUF1341 |
| contig_136 | 3677 | 2574 | - | D-Glucosaminate-6-phosphate ammonia-lyase (EC 4.3.1.-) |
| contig_136 | 4719 | 3655 | - | Metallo-dependent hydrolases, subgroup B |
| contig_1360 | 1428 | 745 | - | FIG01226006: hypothetical protein |
| contig_1361 | 659 | 27 | - | Predicted transcriptional regulator of N-Acetylglucosamine utilization, GntR family |
| contig_1361 | 747 | 634 | - | Predicted transcriptional regulator of N-Acetylglucosamine utilization, GntR family |
| contig_1361 | 1278 | 913 | - | oxidoreductase family protein |
| contig_1361 | 2178 | 1309 | - | oxidoreductase family protein |
| contig_1361 | 2961 | 2233 | - | N-Acetyl-D-glucosamine ABC transport system, permease protein 2 |
| contig_1361 | 3226 | 3089 | - | N-Acetyl-D-glucosamine ABC transport system, permease protein 1 |
| contig_1362 | 139 | 777 | + | internalin, putative |
| contig_1362 | 1660 | 1079 | - | acetyltransferase, CYSE/LACA/LPXA/NODL family |
| contig_1362 | 2015 | 2194 | + | FIG01226086: hypothetical protein |
| contig_1362 | 2382 | 2600 | + | Nudix hydrolase family protein |
| contig_1363 | 1151 | 1717 | + | D-alanyl-D-alanine carboxypeptidase (EC 3.4.16.4) |
| contig_1363 | 1701 | 1871 | + | D-alanyl-D-alanine carboxypeptidase (EC 3.4.16.4) |
| contig_1363 | 1898 | 2107 | + | D-alanyl-D-alanine carboxypeptidase (EC 3.4.16.4) |
| contig_1363 | 2331 | 2456 | + | hypothetical protein |
| contig_1363 | 2453 | 2596 | + | Nitroreductase family protein |
| contig_1363 | 2614 | 2781 | + | Nitroreductase family protein |
| contig_1363 | 2753 | 2959 | + | Nitroreductase family protein |
| contig_1364 | 63 | 194 | + | hypothetical protein |
| contig_1364 | 1016 | 1501 | + | conserved hypothetical protein TIGR00052 |
| contig_1364 | 1913 | 2038 | + | hypothetical protein |
| contig_1365 | 1270 | 623 | - | Transcriptional regulator BkdR of isoleucine and valine catabolism operon |
| contig_1365 | 1899 | 1519 | - | Transcriptional regulator BkdR of isoleucine and valine catabolism operon |
| contig_1367 | 1155 | 613 | - | FIG002344: Hydrolase (HAD superfamily) |
| contig_1367 | 1781 | 1296 | - | FIG002344: Hydrolase (HAD superfamily) |
| contig_1367 | 2118 | 1798 | - | FIG002344: Hydrolase (HAD superfamily) |
| contig_1367 | 2952 | 2665 | - | RecA protein |
| contig_1369 | 123 | 476 | + | Probable poly(beta-D-mannuronate) O-acetylase (EC 2.3.1.-) |
| contig_1369 | 508 | 840 | + | Probable poly(beta-D-mannuronate) O-acetylase (EC 2.3.1.-) |
| contig_1369 | 857 | 1126 | + | FIG01228344: hypothetical protein |
| contig_137 | 167 | 42 | - | hypothetical protein |
| contig_137 | 524 | 300 | - | Putative pheromone cAM373 precursor lipoprotein CamS |
| contig_137 | 837 | 607 | - | Putative pheromone cAM373 precursor lipoprotein CamS |
| contig_137 | 979 | 794 | - | Putative pheromone cAM373 precursor lipoprotein CamS |
| contig_137 | 1495 | 1154 | - | Putative pheromone cAM373 precursor lipoprotein CamS |
| contig_137 | 1765 | 1586 | - | DNA ligase (EC 6.5.1.2) |
| contig_137 | 2495 | 1743 | - | DNA ligase (EC 6.5.1.2) |
| contig_137 | 3523 | 2663 | - | DNA ligase (EC 6.5.1.2) |
| contig_137 | 3896 | 3528 | - | ATP-dependent DNA helicase UvrD/PcrA |
| contig_137 | 4746 | 3904 | - | ATP-dependent DNA helicase UvrD/PcrA |
| contig_137 | 5194 | 4724 | - | ATP-dependent DNA helicase UvrD/PcrA |
| contig_137 | 5405 | 5220 | - | ATP-dependent DNA helicase UvrD/PcrA |
| contig_137 | 5787 | 5413 | - | ATP-dependent DNA helicase UvrD/PcrA |
| contig_137 | 6489 | 5800 | - | (S)-3-O-geranylgeranylglyceryl phosphate synthase |
| contig_137 | 6826 | 7203 | + | FIG01226624: hypothetical protein |
| contig_1370 | 284 | 436 | + | Oligopeptide ABC transporter, periplasmic oligopeptide-binding protein OppA (TC 3.A.1.5.1) |
| contig_1371 | 217 | 56 | - | Sodium/pantothenate symporter |
| contig_1371 | 278 | 159 | - | Sodium/pantothenate symporter |
| contig_1371 | 623 | 2038 | + | Aldehyde dehydrogenase (EC 1.2.1.3) |
| contig_1371 | 2223 | 2089 | - | 3-oxoacyl-[acyl-carrier protein] reductase (EC 1.1.1.100) |
| contig_1375 | 285 | 49 | - | Glycerol-3-phosphate dehydrogenase [NAD(P)+] (EC 1.1.1.94) |
| contig_1375 | 428 | 309 | - | Glycerol-3-phosphate dehydrogenase [NAD(P)+] (EC 1.1.1.94) |
| contig_1375 | 722 | 501 | - | Glycerol-3-phosphate dehydrogenase [NAD(P)+] (EC 1.1.1.94) |
| contig_1376 | 357 | 214 | - | L-Proline/Glycine betaine transporter ProP |
| contig_1376 | 575 | 354 | - | L-Proline/Glycine betaine transporter ProP |
| contig_1376 | 658 | 527 | - | L-Proline/Glycine betaine transporter ProP |
| contig_1376 | 936 | 655 | - | L-Proline/Glycine betaine transporter ProP |
| contig_1376 | 1348 | 983 | - | L-Proline/Glycine betaine transporter ProP |
| contig_1377 | 88 | 1260 | + | L-gulono-1,4-lactone oxidase (EC 1.1.3.8) |
| contig_1377 | 1248 | 1457 | + | oxidoreductase, FAD-binding |
| contig_1378 | 497 | 345 | - | Isovaleryl-CoA dehydrogenase (EC 1.3.8.4) |
| contig_1378 | 1158 | 457 | - | Isovaleryl-CoA dehydrogenase (EC 1.3.8.4) |
| contig_1378 | 1289 | 1146 | - | Isovaleryl-CoA dehydrogenase (EC 1.3.8.4) |
| contig_1378 | 2028 | 1549 | - | COG1683: Uncharacterized conserved protein / FIG143828: Hypothetical protein YbgA |
| contig_1378 | 2272 | 2042 | - | COG1683: Uncharacterized conserved protein / FIG143828: Hypothetical protein YbgA |
| contig_1379 | 378 | 872 | + | membrane protein, putative |
| contig_1379 | 905 | 1135 | + | membrane protein, putative |
| contig_1379 | 1313 | 1477 | + | Phosphonate ABC transporter phosphate-binding periplasmic component (TC 3.A.1.9.1) |
| contig_1379 | 1542 | 1694 | + | Phosphonate ABC transporter phosphate-binding periplasmic component (TC 3.A.1.9.1) |
| contig_1379 | 1748 | 1876 | + | FIG01227908: hypothetical protein |
| contig_1379 | 2468 | 2094 | - | Integral membrane protein |
| contig_1379 | 2643 | 2437 | - | Integral membrane protein |
| contig_138 | 306 | 512 | + | FIG002343: hypothetical protein |
| contig_138 | 502 | 714 | + | FIG002343: hypothetical protein |
| contig_1380 | 4 | 228 | + | FIG01226133: hypothetical protein |
| contig_1380 | 933 | 256 | - | Uracil-DNA glycosylase, family 1 |
| contig_1380 | 1285 | 953 | - | ABC transporter, permease protein |
| contig_1380 | 1971 | 1342 | - | ABC transporter, permease protein |
| contig_1380 | 2713 | 1940 | - | ABC transporter, ATP-binding protein |
| contig_1382 | 179 | 3 | - | ABC transporter, permease protein |
| contig_1382 | 465 | 319 | - | ABC transporter, permease protein |
| contig_1382 | 565 | 449 | - | hypothetical protein |
| contig_1383 | 377 | 54 | - | FIG01225447: hypothetical protein |
| contig_1384 | 34 | 291 | + | UDP-glucose 4-epimerase (EC 5.1.3.2) |
| contig_1384 | 257 | 391 | + | UDP-glucose 4-epimerase (EC 5.1.3.2) |
| contig_1384 | 794 | 360 | - | FIG01235336: hypothetical protein |
| contig_1385 | 330 | 911 | + | Probable transcriptional regulator |
| contig_1386 | 834 | 487 | - | Tn7-like transposition protein A |
| contig_1386 | 1321 | 953 | - | Tn7-like transposition protein A |
| contig_1387 | 254 | 75 | - | Molybdopterin biosynthesis protein MoeB |
| contig_1387 | 518 | 369 | - | Formate efflux transporter (TC 2.A.44 family) |
| contig_1387 | 961 | 647 | - | Formate efflux transporter (TC 2.A.44 family) |
| contig_1387 | 1171 | 1019 | - | Formate efflux transporter (TC 2.A.44 family) |
| contig_1389 | 511 | 77 | - | Aspartate ammonia-lyase (EC 4.3.1.1) |
| contig_139 | 241 | 44 | - | Pyrrolidone-carboxylate peptidase (EC 3.4.19.3) |
| contig_139 | 1304 | 417 | - | FIG001614: Membrane protein |
| contig_139 | 1934 | 1365 | - | FIG015373: Membrane protein |
| contig_139 | 2091 | 1957 | - | hypothetical protein |
| contig_139 | 2756 | 2112 | - | Lactam utilization protein LamB |
| contig_139 | 2993 | 2814 | - | Allophanate hydrolase 2 subunit 2 (EC 3.5.1.54) |
| contig_1390 | 463 | 47 | - | FIG01235603: hypothetical protein |
| contig_1391 | 312 | 136 | - | FIG01227976: hypothetical protein |
| contig_1391 | 689 | 423 | - | Integral membrane protein |
| contig_1392 | 611 | 486 | - | Pyrimidine-nucleoside phosphorylase (EC 2.4.2.2) |
| contig_1395 | 789 | 1463 | + | Flagellar motor rotation protein MotA |
| contig_1396 | 53 | 454 | + | PhnO protein |
| contig_1397 | 45 | 302 | + | HigA protein (antitoxin to HigB) |
| contig_1397 | 436 | 549 | + | FIG01226305: hypothetical protein |
| contig_1397 | 1423 | 782 | - | Sortase A, LPXTG specific |
| contig_1397 | 1603 | 1460 | - | Sortase A, LPXTG specific |
| contig_1397 | 2129 | 1782 | - | Sortase A, LPXTG specific |
| contig_1398 | 125 | 544 | + | MutT/Nudix family protein |
| contig_1398 | 894 | 604 | - | Nicotinamidase (EC 3.5.1.19) |
| contig_1398 | 1158 | 997 | - | Nicotinamidase (EC 3.5.1.19) |
| contig_1399 | 118 | 795 | + | DNA-binding response regulator ResD |
| contig_1399 | 871 | 1041 | + | Sensor histidine kinase ResE (EC 2.7.3.-) |
| contig_14 | 200 | 63 | - | Transketolase (EC 2.2.1.1) |
| contig_14 | 1104 | 160 | - | Transketolase (EC 2.2.1.1) |
| contig_140 | 356 | 30 | - | Cysteinyl-tRNA synthetase (EC 6.1.1.16) |
| contig_140 | 1428 | 364 | - | Cysteinyl-tRNA synthetase (EC 6.1.1.16) |
| contig_140 | 2050 | 1409 | - | Serine acetyltransferase (EC 2.3.1.30) |
| contig_140 | 2980 | 2507 | - | Glutamyl-tRNA synthetase (EC 6.1.1.17) @ Glutamyl-tRNA(Gln) synthetase (EC 6.1.1.24) |
| contig_140 | 3538 | 3113 | - | Glutamyl-tRNA synthetase (EC 6.1.1.17) @ Glutamyl-tRNA(Gln) synthetase (EC 6.1.1.24) |
| contig_140 | 3779 | 3585 | - | Glutamyl-tRNA synthetase (EC 6.1.1.17) @ Glutamyl-tRNA(Gln) synthetase (EC 6.1.1.24) |
| contig_140 | 3984 | 3814 | - | Glutamyl-tRNA synthetase (EC 6.1.1.17) @ Glutamyl-tRNA(Gln) synthetase (EC 6.1.1.24) |
| contig_140 | 4533 | 4156 | - | 2-C-methyl-D-erythritol 2,4-cyclodiphosphate synthase (EC 4.6.1.12) |
| contig_140 | 5183 | 4650 | - | 2-C-methyl-D-erythritol 4-phosphate cytidylyltransferase (EC 2.7.7.60) |
| contig_140 | 5331 | 5173 | - | 2-C-methyl-D-erythritol 4-phosphate cytidylyltransferase (EC 2.7.7.60) |
| contig_140 | 6457 | 5348 | - | Membrane-associated protein containing RNA-binding TRAM domain and ribonuclease PIN-domain, YacL B.subtilis ortholog |
| contig_140 | 7691 | 6618 | - | DNA integrity scanning protein DisA |
| contig_140 | 9011 | 7695 | - | DNA repair protein RadA |
| contig_140 | 11606 | 9168 | - | ATP-dependent Clp protease, ATP-binding subunit ClpC / Negative regulator of genetic competence clcC/mecB |
| contig_140 | 12027 | 11629 | - | Putative ATP:guanido phosphotransferase YacI (EC 2.7.3.-) |
| contig_140 | 12694 | 12059 | - | Putative ATP:guanido phosphotransferase YacI (EC 2.7.3.-) |
| contig_140 | 13249 | 12851 | - | Nucleotide excision repair protein, with UvrB/UvrC motif |
| contig_140 | 13884 | 13423 | - | Transcriptional regulator CtsR |
| contig_1400 | 340 | 453 | + | FIG01227112: hypothetical protein |
| contig_1402 | 721 | 575 | - | Stage II sporulation protein P |
| contig_1402 | 889 | 776 | - | Stage II sporulation protein P |
| contig_1404 | 1022 | 717 | - | Flagellar hook-length control protein FliK |
| contig_1406 | 569 | 363 | - | ABC transporter, permease protein, putative |
| contig_1406 | 946 | 632 | - | ABC transporter, permease protein, putative |
| contig_1406 | 1133 | 897 | - | ABC transporter, permease protein, putative |
| contig_1406 | 1885 | 1133 | - | ABC transporter, ATP-binding protein |
| contig_1407 | 153 | 356 | + | Cobalt-zinc-cadmium resistance protein |
| contig_1407 | 452 | 889 | + | Cobalt-zinc-cadmium resistance protein |
| contig_1407 | 915 | 1034 | + | Cobalt-zinc-cadmium resistance protein |
| contig_1407 | 1520 | 1029 | - | Phosphatidylglycerophosphatase B (EC 3.1.3.27) |
| contig_1407 | 1825 | 1661 | - | hypothetical protein |
| contig_1407 | 2065 | 1892 | - | Sporulation control protein Spo0M |
| contig_1407 | 2587 | 2150 | - | Serine/threonine protein kinase (EC 2.7.1.37) |
| contig_1407 | 2802 | 2596 | - | Serine/threonine protein kinase (EC 2.7.1.37) |
| contig_1407 | 3081 | 2938 | - | FIG01226893: hypothetical protein |
| contig_1407 | 3315 | 3199 | - | FIG01227129: hypothetical protein |
| contig_1407 | 3530 | 3697 | + | FIG01225147: hypothetical protein |
| contig_1407 | 3857 | 3702 | - | IG hypothetical 18022 |
| contig_1408 | 970 | 185 | - | Choloylglycine hydrolase (EC 3.5.1.24) |
| contig_1408 | 1481 | 1239 | - | DNA mismatch repair protein MutL |
| contig_1409 | 389 | 505 | + | Guanine-hypoxanthine permease |
| contig_141 | 481 | 801 | + | Histidine triad (HIT) nucleotide-binding protein, similarity with At5g48545 and yeast YDL125C (HNT1) |
| contig_141 | 777 | 917 | + | Histidine triad (HIT) nucleotide-binding protein, similarity with At5g48545 and yeast YDL125C (HNT1) |
| contig_141 | 1307 | 1456 | + | Hypothetical protein SAV1839 |
| contig_141 | 1757 | 2050 | + | Protease production regulatory protein Hpr (ScoC) |
| contig_141 | 2007 | 2315 | + | Protease production regulatory protein Hpr (ScoC) |
| contig_141 | 2655 | 2317 | - | FIG01226172: hypothetical protein |
| contig_141 | 2774 | 2968 | + | FIG01227217: hypothetical protein |
| contig_141 | 3394 | 4257 | + | Foldase protein PrsA precursor (EC 5.2.1.8) @ Foldase clustered with pyrimidine conversion |
| contig_141 | 4504 | 4292 | - | stage V sporulation protein K |
| contig_141 | 4895 | 4497 | - | stage V sporulation protein K |
| contig_141 | 5760 | 4855 | - | stage V sporulation protein K |
| contig_1410 | 235 | 47 | - | hypothetical protein |
| contig_1411 | 211 | 23 | - | Phage protein |
| contig_1412 | 53 | 208 | + | Putative metal chaperone, involved in Zn homeostasis, GTPase of COG0523 family |
| contig_1412 | 499 | 666 | + | Putative metal chaperone, involved in Zn homeostasis, GTPase of COG0523 family |
| contig_1412 | 626 | 847 | + | Putative metal chaperone, involved in Zn homeostasis, GTPase of COG0523 family |
| contig_1412 | 852 | 998 | + | Putative metal chaperone, involved in Zn homeostasis, GTPase of COG0523 family |
| contig_1412 | 1407 | 1114 | - | Spermine/spermidine acetyltransferase (EC 2.3.1.57) |
| contig_1412 | 2210 | 2097 | - | Zinc ABC transporter, periplasmic-binding protein ZnuA |
| contig_1412 | 2682 | 2521 | - | Zinc ABC transporter, periplasmic-binding protein ZnuA |
| contig_1413 | 82 | 564 | + | acetyltransferase, GNAT family |
| contig_1413 | 718 | 876 | + | FIG01226177: hypothetical protein |
| contig_1413 | 943 | 1110 | + | FIG01226177: hypothetical protein |
| contig_1413 | 1370 | 1257 | - | SAM-dependent methyltransferase (EC 2.1.1.-) |
| contig_1413 | 1758 | 1531 | - | SAM-dependent methyltransferase (EC 2.1.1.-) |
| contig_1414 | 423 | 686 | + | Superfamily II DNA/RNA helicases, SNF2 family |
| contig_1414 | 643 | 1239 | + | Superfamily II DNA/RNA helicases, SNF2 family |
| contig_1415 | 408 | 22 | - | FIG01225448: hypothetical protein |
| contig_1416 | 41 | 265 | + | Ku domain protein |
| contig_1416 | 417 | 283 | - | FIG01226746: hypothetical protein |
| contig_1416 | 757 | 401 | - | FIG01226746: hypothetical protein |
| contig_1417 | 381 | 196 | - | Isoleucyl-tRNA synthetase (EC 6.1.1.5) |
| contig_1417 | 715 | 356 | - | Isoleucyl-tRNA synthetase (EC 6.1.1.5) |
| contig_1417 | 1270 | 1106 | - | TPR-repeat-containing protein |
| contig_1417 | 1601 | 1281 | - | TPR-repeat-containing protein |
| contig_1418 | 608 | 39 | - | GTP cyclohydrolase I (EC 3.5.4.16) type 1 |
| contig_1418 | 1001 | 729 | - | DNA-binding protein HBsu |
| contig_1418 | 1483 | 1301 | - | Stage IV sporulation protein A |
| contig_1418 | 1882 | 1520 | - | Stage IV sporulation protein A |
| contig_1418 | 2234 | 1953 | - | Stage IV sporulation protein A |
| contig_142 | 1239 | 583 | - | Homoserine dehydrogenase (EC 1.1.1.3) |
| contig_142 | 1674 | 1381 | - | Homoserine O-succinyltransferase (EC 2.3.1.46) |
| contig_142 | 1904 | 1671 | - | Homoserine O-succinyltransferase (EC 2.3.1.46) |
| contig_142 | 2284 | 1931 | - | Homoserine O-succinyltransferase (EC 2.3.1.46) |
| contig_142 | 3566 | 2268 | - | O-acetylhomoserine sulfhydrylase (EC 2.5.1.49) / O-succinylhomoserine sulfhydrylase (EC 2.5.1.48) |
| contig_142 | 3809 | 3669 | - | hypothetical protein |
| contig_142 | 4512 | 4036 | - | 5'-nucleotidase YjjG (EC 3.1.3.5) |
| contig_142 | 4733 | 4587 | - | 5'-nucleotidase YjjG (EC 3.1.3.5) |
| contig_142 | 4840 | 5511 | + | Predicted N-ribosylNicotinamide CRP-like regulator |
| contig_142 | 6205 | 5579 | - | FMN-dependent NADH-azoreductase |
| contig_142 | 7042 | 6395 | - | DNA-binding response regulator, LuxR family |
| contig_142 | 8407 | 7058 | - | sensor histidine kinase, putative |
| contig_142 | 8635 | 8477 | - | sensor histidine kinase, putative |
| contig_1420 | 9 | 296 | + | Glyoxalase family protein |
| contig_1420 | 613 | 350 | - | Sortase A, LPXTG specific |
| contig_1420 | 1167 | 652 | - | Sortase A, LPXTG specific |
| contig_1420 | 1639 | 1286 | - | Collagen adhesion protein |
| contig_1420 | 1775 | 1593 | - | Collagen adhesion protein |
| contig_1420 | 2016 | 1762 | - | Collagen adhesion protein |
| contig_1422 | 95 | 799 | + | Pantothenate:Na+ symporter (TC 2.A.21.1.1) |
| contig_1422 | 1091 | 966 | - | FIG01230062: hypothetical protein |
| contig_1422 | 1146 | 1361 | + | FIG01226811: hypothetical protein |
| contig_1422 | 1508 | 1386 | - | lipoprotein, putative |
| contig_1422 | 1683 | 1498 | - | FIG01234173: hypothetical protein |
| contig_1423 | 828 | 709 | - | Inosine-uridine preferring nucleoside hydrolase (EC 3.2.2.1) in exosporium |
| contig_1423 | 1148 | 930 | - | Inosine-uridine preferring nucleoside hydrolase (EC 3.2.2.1) in exosporium |
| contig_1426 | 52 | 201 | + | Mg(2+) transport ATPase protein C |
| contig_1427 | 1559 | 1248 | - | Cardiolipin synthetase (EC 2.7.8.-) |
| contig_1427 | 1797 | 1964 | + | hypothetical protein |
| contig_1427 | 1995 | 2309 | + | bll5579; hypothetical protein |
| contig_143 | 291 | 112 | - | Glycine dehydrogenase [decarboxylating] (glycine cleavage system P2 protein) (EC 1.4.4.2) |
| contig_143 | 922 | 347 | - | Glycine dehydrogenase [decarboxylating] (glycine cleavage system P2 protein) (EC 1.4.4.2) |
| contig_143 | 1565 | 939 | - | Glycine dehydrogenase [decarboxylating] (glycine cleavage system P2 protein) (EC 1.4.4.2) |
| contig_143 | 1816 | 1562 | - | Glycine dehydrogenase [decarboxylating] (glycine cleavage system P1 protein) (EC 1.4.4.2) |
| contig_143 | 2906 | 1797 | - | Glycine dehydrogenase [decarboxylating] (glycine cleavage system P1 protein) (EC 1.4.4.2) |
| contig_143 | 4029 | 3031 | - | Aminomethyltransferase (glycine cleavage system T protein) (EC 2.1.2.10) |
| contig_1430 | 95 | 337 | + | hypothetical protein |
| contig_1430 | 621 | 794 | + | hypothetical protein |
| contig_1431 | 304 | 20 | - | Penicillin-binding protein 3 |
| contig_1431 | 401 | 267 | - | Penicillin-binding protein 3 |
| contig_1432 | 265 | 26 | - | Rrf2 family transcriptional regulator, group III |
| contig_1432 | 422 | 231 | - | Rrf2 family transcriptional regulator, group III |
| contig_1432 | 1215 | 787 | - | Possible arsenate reductase (Glutaredoxin) (EC 1.20.4.1) |
| contig_1433 | 696 | 37 | - | FIG01225986: hypothetical protein |
| contig_1434 | 21 | 212 | + | Acetyltransferase, GNAT family (EC 2.3.1.-) |
| contig_1437 | 277 | 636 | + | Isochorismatase (EC 3.3.2.1) |
| contig_1437 | 626 | 841 | + | Isochorismatase (EC 3.3.2.1) |
| contig_1437 | 2520 | 1393 | - | Delta-1-pyrroline-5-carboxylate dehydrogenase (EC 1.2.1.88) |
| contig_1439 | 840 | 472 | - | N-acetylglucosamine kinase of eukaryotic type (EC 2.7.1.59) |
| contig_144 | 781 | 272 | - | Threonine dehydratase biosynthetic (EC 4.3.1.19) |
| contig_144 | 1223 | 768 | - | Threonine dehydratase biosynthetic (EC 4.3.1.19) |
| contig_144 | 1534 | 1340 | - | Threonine dehydratase biosynthetic (EC 4.3.1.19) |
| contig_144 | 1710 | 1570 | - | hypothetical protein |
| contig_144 | 3241 | 1763 | - | Dihydroxy-acid dehydratase (EC 4.2.1.9) |
| contig_144 | 3479 | 3288 | - | Ketol-acid reductoisomerase (EC 1.1.1.86) |
| contig_144 | 4180 | 3476 | - | Ketol-acid reductoisomerase (EC 1.1.1.86) |
| contig_1440 | 42 | 167 | + | hypothetical protein |
| contig_1441 | 162 | 344 | + | Sodium-dependent transporter |
| contig_1441 | 368 | 553 | + | Sodium-dependent transporter |
| contig_1441 | 642 | 1211 | + | Sodium-dependent transporter |
| contig_1443 | 286 | 609 | + | 4-carboxymuconolactone decarboxylase (EC 4.1.1.44) |
| contig_1443 | 837 | 1211 | + | Transcriptional regulator, MarR family |
| contig_1443 | 1331 | 1627 | + | Bacterial luciferase family protein |
| contig_1443 | 1665 | 2390 | + | Bacterial luciferase family protein |
| contig_1443 | 3265 | 3912 | + | Cytochrome d ubiquinol oxidase subunit I (EC 1.10.3.-) |
| contig_1443 | 3909 | 4400 | + | Cytochrome d ubiquinol oxidase subunit I (EC 1.10.3.-) |
| contig_1443 | 4387 | 4821 | + | Cytochrome d ubiquinol oxidase subunit II (EC 1.10.3.-) |
| contig_1443 | 4889 | 5404 | + | Cytochrome d ubiquinol oxidase subunit II (EC 1.10.3.-) |
| contig_1443 | 5404 | 5871 | + | Transport ATP-binding protein CydC |
| contig_1444 | 253 | 927 | + | L-Proline/Glycine betaine transporter ProP |
| contig_1444 | 890 | 1189 | + | L-Proline/Glycine betaine transporter ProP |
| contig_1446 | 189 | 314 | + | 2,3-dihydro-2,3-dihydroxybenzoate dehydrogenase (EC 1.3.1.28) [bacillibactin] siderophore @ 2,3-dihydro-2,3-dihydroxybenzoate dehydrogenase (EC 1.3.1.28) of siderophore biosynthesis |
| contig_1446 | 335 | 682 | + | 2,3-dihydro-2,3-dihydroxybenzoate dehydrogenase (EC 1.3.1.28) [bacillibactin] siderophore @ 2,3-dihydro-2,3-dihydroxybenzoate dehydrogenase (EC 1.3.1.28) of siderophore biosynthesis |
| contig_1446 | 1077 | 1238 | + | Isochorismate synthase (EC 5.4.4.2) [bacillibactin] siderophore @ Isochorismate synthase (EC 5.4.4.2) of siderophore biosynthesis |
| contig_1446 | 1177 | 1779 | + | Isochorismate synthase (EC 5.4.4.2) [bacillibactin] siderophore @ Isochorismate synthase (EC 5.4.4.2) of siderophore biosynthesis |
| contig_1446 | 1773 | 1982 | + | Isochorismate synthase (EC 5.4.4.2) [bacillibactin] siderophore @ Isochorismate synthase (EC 5.4.4.2) of siderophore biosynthesis |
| contig_1446 | 1995 | 2588 | + | 2,3-dihydroxybenzoate-AMP ligase (EC 2.7.7.58) of siderophore biosynthesis |
| contig_1446 | 2674 | 3633 | + | 2,3-dihydroxybenzoate-AMP ligase (EC 2.7.7.58) of siderophore biosynthesis |
| contig_1446 | 3750 | 4532 | + | Isochorismatase (EC 3.3.2.1) [bacillibactin] siderophore @ Isochorismatase (EC 3.3.2.1) of siderophore biosynthesis |
| contig_1446 | 4566 | 4988 | + | Siderophore biosynthesis non-ribosomal peptide synthetase modules @ Bacillibactin synthetase component F (EC 2.7.7.-) |
| contig_1446 | 4988 | 5509 | + | Siderophore biosynthesis non-ribosomal peptide synthetase modules @ Bacillibactin synthetase component F (EC 2.7.7.-) |
| contig_1447 | 361 | 92 | - | Serine protease, DegP/HtrA, do-like (EC 3.4.21.-) |
| contig_1449 | 167 | 313 | + | peptidase, M23/M37 family |
| contig_1449 | 292 | 483 | + | Peptidase, M23/M37 family |
| contig_1449 | 809 | 1324 | + | Peptidase, M23/M37 family |
| contig_1449 | 1330 | 1524 | + | FIG013457: hypothetical protein |
| contig_1449 | 1515 | 1661 | + | FIG013457: hypothetical protein |
| contig_1449 | 2732 | 1887 | - | FIG014736: hypothetical protein |
| contig_145 | 146 | 343 | + | FIG011871: Hypothetical protein |
| contig_145 | 330 | 491 | + | FIG011871: Hypothetical protein |
| contig_145 | 792 | 911 | + | Methionine ABC transporter ATP-binding protein |
| contig_145 | 908 | 1507 | + | Methionine ABC transporter ATP-binding protein |
| contig_145 | 1509 | 1934 | + | Methionine ABC transporter ATP-binding protein |
| contig_145 | 1927 | 2286 | + | Methionine ABC transporter permease protein |
| contig_145 | 2298 | 2594 | + | Methionine ABC transporter permease protein |
| contig_145 | 2617 | 2826 | + | Methionine ABC transporter substrate-binding protein |
| contig_145 | 3012 | 3215 | + | Methionine ABC transporter substrate-binding protein |
| contig_145 | 3504 | 3653 | + | Methionine ABC transporter substrate-binding protein |
| contig_1450 | 16 | 165 | + | sensory box histidine kinase |
| contig_1450 | 998 | 396 | - | Substrate-specific component ThiT of thiamin ECF transporter |
| contig_1450 | 1422 | 1649 | + | Ferrous iron transport protein A |
| contig_1450 | 1646 | 3634 | + | Ferrous iron transport protein B |
| contig_1451 | 55 | 489 | + | Flavodoxin |
| contig_1451 | 516 | 845 | + | BNR repeat domain protein |
| contig_1451 | 814 | 930 | + | BNR repeat domain protein |
| contig_1452 | 80 | 478 | + | PBS lyase HEAT-like repeat domain protein |
| contig_1452 | 482 | 868 | + | Thioredoxin |
| contig_1452 | 999 | 877 | - | hypothetical protein |
| contig_1453 | 94 | 507 | + | ABC1 family protein |
| contig_1453 | 515 | 1039 | + | ABC1 family protein |
| contig_1454 | 771 | 151 | - | sensor histidine kinase |
| contig_1454 | 1004 | 843 | - | sensor histidine kinase |
| contig_1455 | 2 | 127 | + | Phosphoesterase |
| contig_1455 | 133 | 474 | + | Phosphoesterase |
| contig_1456 | 17 | 130 | + | acetyltransferase, GNAT family |
| contig_1457 | 364 | 83 | - | ATP-dependent RNA helicase BA2475 |
| contig_1457 | 794 | 357 | - | ATP-dependent RNA helicase BA2475 |
| contig_1457 | 935 | 798 | - | hypothetical protein |
| contig_1458 | 213 | 413 | + | Transporter |
| contig_1458 | 961 | 758 | - | membrane protein, putative |
| contig_1458 | 1295 | 1161 | - | Similar to ribosomal large subunit pseudouridine synthase D, Bacillus subtilis YhcT type |
| contig_1458 | 1739 | 1521 | - | Similar to ribosomal large subunit pseudouridine synthase D, Bacillus subtilis YhcT type |
| contig_1458 | 1879 | 1697 | - | Similar to ribosomal large subunit pseudouridine synthase D, Bacillus subtilis YhcT type |
| contig_1458 | 2088 | 1906 | - | Similar to ribosomal large subunit pseudouridine synthase D, Bacillus subtilis YhcT type |
| contig_1458 | 2602 | 2186 | - | FIG01228725: hypothetical protein |
| contig_1459 | 85 | 405 | + | Transcriptional regulator, ArsR family |
| contig_1459 | 473 | 646 | + | Arginine/ornithine antiporter ArcD |
| contig_1459 | 621 | 743 | + | Arginine/ornithine antiporter ArcD |
| contig_1459 | 1151 | 744 | - | hypothetical protein |
| contig_1459 | 1135 | 1401 | + | Arginine/ornithine antiporter ArcD |
| contig_1459 | 1522 | 1737 | + | bypass-of-forespore protein C, putative |
| contig_1459 | 1835 | 2053 | + | bypass-of-forespore protein C, putative |
| contig_1459 | 2254 | 2700 | + | Holliday junction DNA helicase RuvA |
| contig_1459 | 2669 | 2872 | + | Holliday junction DNA helicase RuvA |
| contig_1459 | 2878 | 3480 | + | Holliday junction DNA helicase RuvB |
| contig_1459 | 3449 | 3880 | + | Holliday junction DNA helicase RuvB |
| contig_146 | 54 | 251 | + | CbiN domain protein |
| contig_146 | 895 | 1080 | + | GTP-binding protein HflX |
| contig_146 | 1118 | 1312 | + | hypothetical protein |
| contig_146 | 1266 | 1475 | + | GTP-binding protein HflX |
| contig_146 | 1538 | 2158 | + | GTP-binding protein related to HflX |
| contig_1460 | 666 | 85 | - | Salicylate hydroxylase (EC 1.14.13.1) |
| contig_1461 | 388 | 143 | - | Multimodular transpeptidase-transglycosylase (EC 2.4.1.129) (EC 3.4.-.-) / Penicillin-binding protein 1A/1B (PBP1) |
| contig_1461 | 882 | 409 | - | Multimodular transpeptidase-transglycosylase (EC 2.4.1.129) (EC 3.4.-.-) / Penicillin-binding protein 1A/1B (PBP1) |
| contig_1461 | 1496 | 939 | - | Multimodular transpeptidase-transglycosylase (EC 2.4.1.129) (EC 3.4.-.-) / Penicillin-binding protein 1A/1B (PBP1) |
| contig_1461 | 1836 | 1510 | - | Multimodular transpeptidase-transglycosylase (EC 2.4.1.129) (EC 3.4.-.-) / Penicillin-binding protein 1A/1B (PBP1) |
| contig_1462 | 229 | 110 | - | N-acetylmannosaminyltransferase (EC 2.4.1.187) |
| contig_1463 | 298 | 140 | - | Superoxide dismutase [Cu-Zn] precursor (EC 1.15.1.1) |
| contig_1463 | 680 | 270 | - | Superoxide dismutase [Cu-Zn] precursor (EC 1.15.1.1) |
| contig_1463 | 925 | 749 | - | FIG01226254: hypothetical protein |
| contig_1464 | 926 | 222 | - | Methyltransferase (EC 2.1.1.-) |
| contig_1464 | 1115 | 972 | - | hypothetical protein |
| contig_1464 | 1440 | 1285 | - | PhnB protein; putative DNA binding 3-demethylubiquinone-9 3-methyltransferase domain protein |
| contig_1464 | 1630 | 1490 | - | PhnB protein; putative DNA binding 3-demethylubiquinone-9 3-methyltransferase domain protein |
| contig_1464 | 2028 | 1750 | - | Methyltransferase (EC 2.1.1.-) |
| contig_1465 | 262 | 38 | - | Transporter, LysE family |
| contig_1465 | 545 | 375 | - | Transporter, LysE family |
| contig_1468 | 220 | 513 | + | FIG01229271: hypothetical protein |
| contig_1468 | 547 | 930 | + | FIG01229271: hypothetical protein |
| contig_1468 | 977 | 1105 | + | FIG01225858: hypothetical protein |
| contig_1469 | 213 | 61 | - | Glycerate kinase (EC 2.7.1.31) |
| contig_1469 | 533 | 222 | - | Glycerate kinase (EC 2.7.1.31) |
| contig_1469 | 1095 | 541 | - | Glycerate kinase (EC 2.7.1.31) |
| contig_1472 | 501 | 133 | - | Microcin C7 self-immunity protein mccF |
| contig_1472 | 724 | 452 | - | Microcin C7 self-immunity protein mccF |
| contig_1472 | 833 | 705 | - | Microcin C7 self-immunity protein mccF |
| contig_1472 | 1170 | 820 | - | Microcin C7 self-immunity protein mccF |
| contig_1473 | 750 | 1175 | + | Ser/Thr protein phosphatase family protein |
| contig_1474 | 678 | 289 | - | Peptidase T (EC 3.4.11.4) |
| contig_1474 | 1174 | 662 | - | Peptidase T (EC 3.4.11.4) |
| contig_1474 | 1433 | 1302 | - | Ribosomal-protein-S5p-alanine acetyltransferase |
| contig_1474 | 1845 | 1618 | - | Ribosomal-protein-S5p-alanine acetyltransferase |
| contig_1474 | 2444 | 2205 | - | FIG01227344: hypothetical protein |
| contig_1474 | 3074 | 2562 | - | Protein erfK/srfK |
| contig_1474 | 3561 | 3091 | - | Integral membrane protein |
| contig_1474 | 3727 | 3584 | - | Integral membrane protein |
| contig_1475 | 397 | 143 | - | Biotin carboxyl carrier protein |
| contig_1476 | 724 | 74 | - | hypothetical protein |
| contig_1478 | 116 | 352 | + | peptidase, M23/M37 family |
| contig_1479 | 131 | 694 | + | Thiaminase II (EC 3.5.99.2) |
| contig_148 | 256 | 501 | + | S-layer protein EA1 |
| contig_148 | 762 | 598 | - | FIG01226780: hypothetical protein |
| contig_148 | 1010 | 750 | - | FIG01226780: hypothetical protein |
| contig_148 | 1210 | 1061 | - | FIG01226780: hypothetical protein |
| contig_148 | 1793 | 1392 | - | FIG01226780: hypothetical protein |
| contig_148 | 2726 | 1818 | - | Probable poly(beta-D-mannuronate) O-acetylase (EC 2.3.1.-) |
| contig_148 | 3237 | 2686 | - | Probable poly(beta-D-mannuronate) O-acetylase (EC 2.3.1.-) |
| contig_1483 | 588 | 382 | - | FIG01226089: hypothetical protein |
| contig_1484 | 366 | 49 | - | N-acetylmuramoyl-L-alanine amidase |
| contig_1484 | 1012 | 590 | - | Serine/threonine protein phosphatase (EC 3.1.3.16) |
| contig_1484 | 1305 | 1039 | - | Serine/threonine protein phosphatase (EC 3.1.3.16) |
| contig_1485 | 704 | 576 | - | Nitrate/nitrite sensor protein (EC 2.7.3.-) |
| contig_1488 | 253 | 119 | - | hypothetical protein |
| contig_1489 | 1 | 343 | + | Small Subunit Ribosomal RNA; ssuRNA; SSU rRNA |
| contig_1489 | 474 | 550 | + | tRNA-Ile-GAT |
| contig_149 | 12 | 449 | + | NADH dehydrogenase (EC 1.6.99.3) |
| contig_149 | 446 | 1003 | + | NADH dehydrogenase (EC 1.6.99.3) |
| contig_149 | 1107 | 1229 | + | hypothetical protein |
| contig_149 | 1240 | 1371 | + | Nudix hydrolase family protein |
| contig_1490 | 76 | 369 | + | Hypothetical protein in cluster with penicillin-binding protein PBP1, Bacillus type |
| contig_1491 | 160 | 279 | + | hypothetical protein |
| contig_1491 | 257 | 400 | + | hypothetical protein |
| contig_1491 | 487 | 720 | + | Phosphoadenylyl-sulfate reductase [thioredoxin] (EC 1.8.4.8) / Adenylyl-sulfate reductase [thioredoxin] (EC 1.8.4.10) |
| contig_1491 | 677 | 1192 | + | Phosphoadenylyl-sulfate reductase [thioredoxin] (EC 1.8.4.8) / Adenylyl-sulfate reductase [thioredoxin] (EC 1.8.4.10) |
| contig_1491 | 1231 | 1608 | + | Sulfate adenylyltransferase, dissimilatory-type (EC 2.7.7.4) |
| contig_1491 | 1622 | 2368 | + | Sulfate adenylyltransferase, dissimilatory-type (EC 2.7.7.4) |
| contig_1491 | 2381 | 2782 | + | Adenylylsulfate kinase (EC 2.7.1.25) |
| contig_1491 | 2797 | 2976 | + | Adenylylsulfate kinase (EC 2.7.1.25) |
| contig_1492 | 524 | 123 | - | Phenylalanyl-tRNA synthetase beta chain (EC 6.1.1.20) |
| contig_1492 | 847 | 536 | - | Phenylalanyl-tRNA synthetase beta chain (EC 6.1.1.20) |
| contig_1492 | 1566 | 922 | - | Phenylalanyl-tRNA synthetase beta chain (EC 6.1.1.20) |
| contig_1492 | 2484 | 1585 | - | Phenylalanyl-tRNA synthetase alpha chain (EC 6.1.1.20) |
| contig_1493 | 1093 | 329 | - | Cell division protein FtsK |
| contig_1493 | 1659 | 1315 | - | FIG01228887: hypothetical protein |
| contig_1493 | 2004 | 1855 | - | Phenylalanyl-tRNA synthetase domain protein (Bsu YtpR) |
| contig_1493 | 2471 | 2208 | - | Phenylalanyl-tRNA synthetase domain protein (Bsu YtpR) |
| contig_1493 | 2848 | 2468 | - | FIG002434: Uncharacterized protein YtpQ |
| contig_1493 | 3056 | 2808 | - | FIG002434: Uncharacterized protein YtpQ |
| contig_1493 | 3246 | 3049 | - | FIG002434: Uncharacterized protein YtpQ |
| contig_1494 | 181 | 330 | + | PTS system, N-acetylmuramic acid-specific IIB component (EC 2.7.1.69) / PTS system, N-acetylmuramic acid-specific IIC component |
| contig_1494 | 338 | 481 | + | PTS system, IIB component (EC 2.7.1.69) / PTS system, IIC component (EC 2.7.1.69) |
| contig_1494 | 632 | 1546 | + | PTS system, N-acetylmuramic acid-specific IIB component (EC 2.7.1.69) / PTS system, N-acetylmuramic acid-specific IIC component |
| contig_1495 | 418 | 224 | - | Transcriptional regulator, TetR family |
| contig_1495 | 596 | 372 | - | Transcriptional regulator, TetR family |
| contig_1495 | 1219 | 1067 | - | Regulatory sensor-transducer, BlaR1/MecR1 family |
| contig_1496 | 16 | 174 | + | Fumarylacetoacetate hydrolase family protein |
| contig_1496 | 229 | 573 | + | Fumarylacetoacetate hydrolase family protein |
| contig_1496 | 551 | 847 | + | Fumarylacetoacetate hydrolase family protein |
| contig_1496 | 1672 | 1409 | - | Ornithine aminotransferase (EC 2.6.1.13) |
| contig_1496 | 1835 | 1638 | - | Ornithine aminotransferase (EC 2.6.1.13) |
| contig_1496 | 2472 | 1792 | - | Ornithine aminotransferase (EC 2.6.1.13) |
| contig_1496 | 2623 | 2937 | + | Uncharacterized protein UPF0344 |
| contig_1496 | 3242 | 3051 | - | FIG00673530: hypothetical protein |
| contig_1496 | 3375 | 3247 | - | FIG00673530: hypothetical protein |
| contig_1496 | 3509 | 3363 | - | FIG00673530: hypothetical protein |
| contig_1496 | 3609 | 3472 | - | FIG00673530: hypothetical protein |
| contig_1497 | 783 | 103 | - | Substrate-specific component BioY of biotin ECF transporter |
| contig_1497 | 1744 | 929 | - | Medium-chain-fatty-acid--CoA ligase (EC 6.2.1.-) |
| contig_1497 | 1913 | 1731 | - | Medium-chain-fatty-acid--CoA ligase (EC 6.2.1.-) |
| contig_1498 | 369 | 488 | + | hypothetical protein |
| contig_1498 | 657 | 956 | + | Glyoxalase family protein |
| contig_1498 | 943 | 1125 | + | Glyoxalase family protein |
| contig_1498 | 1133 | 1300 | + | Macrolide-efflux protein |
| contig_1499 | 1130 | 57 | - | BNR repeat domain protein |
| contig_15 | 982 | 344 | - | Uridine kinase (EC 2.7.1.48) [C1] |
| contig_15 | 1602 | 1000 | - | peptidase, U32 family large subunit [C1] |
| contig_15 | 1932 | 1699 | - | peptidase, U32 family large subunit [C1] |
| contig_15 | 2092 | 1898 | - | peptidase, U32 family large subunit [C1] |
| contig_15 | 2636 | 2505 | - | peptidase, U32 family small subunit [C1] |
| contig_15 | 3231 | 2644 | - | peptidase, U32 family small subunit [C1] |
| contig_15 | 3676 | 3239 | - | FIG011945: O-methyltransferase family protein |
| contig_15 | 3881 | 3684 | - | FIG011945: O-methyltransferase family protein |
| contig_15 | 4864 | 4085 | - | FIG004453: protein YceG like |
| contig_15 | 5491 | 5372 | - | Ortholog of S. aureus MRSA252 (BX571856) SAR1694 |
| contig_15 | 5651 | 5526 | - | Ortholog of S. aureus MRSA252 (BX571856) SAR1694 |
| contig_15 | 5885 | 5664 | - | Putative Holliday junction resolvase YqgF |
| contig_15 | 6076 | 5933 | - | Putative Holliday junction resolvase YqgF |
| contig_15 | 6346 | 6080 | - | FIG01055109: hypothetical protein |
| contig_15 | 6689 | 6423 | - | Alanyl-tRNA synthetase (EC 6.1.1.7) |
| contig_15 | 7143 | 6682 | - | Alanyl-tRNA synthetase (EC 6.1.1.7) |
| contig_150 | 667 | 89 | - | proton/sodium-glutamate symport protein |
| contig_150 | 1307 | 708 | - | proton/sodium-glutamate symport protein |
| contig_150 | 1532 | 1834 | + | PTS system, cellobiose-specific IIB component (EC 2.7.1.69) |
| contig_150 | 1849 | 2322 | + | PTS system, cellobiose-specific IIC component (EC 2.7.1.69) |
| contig_150 | 2362 | 3150 | + | PTS system, cellobiose-specific IIC component (EC 2.7.1.69) |
| contig_150 | 3160 | 3477 | + | PTS system, cellobiose-specific IIA component (EC 2.7.1.69) |
| contig_150 | 4099 | 3542 | - | FIG01231863: hypothetical protein |
| contig_150 | 4371 | 4216 | - | RNA polymerase sigma-70 factor |
| contig_150 | 4459 | 4328 | - | hypothetical protein |
| contig_150 | 4642 | 4517 | - | RNA polymerase sigma-70 factor |
| contig_1500 | 985 | 32 | - | N-acetylmuramoyl-L-alanine amidase |
| contig_1500 | 1427 | 1116 | - | N-acetylmuramoyl-L-alanine amidase |
| contig_1500 | 1809 | 1495 | - | N-acetylmuramoyl-L-alanine amidase |
| contig_1501 | 535 | 158 | - | putative cytochrome P450 hydroxylase |
| contig_1501 | 701 | 498 | - | putative cytochrome P450 hydroxylase |
| contig_1501 | 980 | 813 | - | putative cytochrome P450 hydroxylase |
| contig_1501 | 1685 | 1545 | - | FIG01225786: hypothetical protein |
| contig_1502 | 230 | 1666 | + | drug resistance transporter, EmrB/QacA family |
| contig_1502 | 2022 | 1699 | - | Transcriptional regulator, ArsR family |
| contig_1502 | 2460 | 2023 | - | FIG01226157: hypothetical protein |
| contig_1502 | 2896 | 3123 | + | FIG01225868: hypothetical protein |
| contig_1503 | 252 | 470 | + | FIG01230158: hypothetical protein |
| contig_1504 | 127 | 459 | + | Lactoylglutathione lyase and related lyases |
| contig_1504 | 1172 | 1399 | + | Copper resistance protein CopC / Copper resistance protein CopD |
| contig_1504 | 1488 | 1691 | + | Copper resistance protein CopC / Copper resistance protein CopD |
| contig_1504 | 2057 | 1860 | - | hypothetical protein |
| contig_1505 | 15 | 191 | + | Lon-like protease with PDZ domain |
| contig_1505 | 160 | 297 | + | Lon-like protease with PDZ domain |
| contig_1505 | 1095 | 466 | - | FIG007079: UPF0348 protein family |
| contig_1505 | 1259 | 1098 | - | FIG007079: UPF0348 protein family |
| contig_1505 | 1665 | 1234 | - | FIG007079: UPF0348 protein family |
| contig_1505 | 1965 | 1846 | - | hypothetical protein |
| contig_1505 | 1913 | 2227 | + | COG1399 protein in cluster with ribosomal protein L32p, Firmicutes subfamily |
| contig_1505 | 2289 | 2462 | + | LSU ribosomal protein L32p |
| contig_1507 | 90 | 533 | + | membrane protein, putative |
| contig_1507 | 622 | 840 | + | membrane protein, putative |
| contig_1507 | 812 | 940 | + | membrane protein, putative |
| contig_1507 | 1624 | 1010 | - | Proline iminopeptidase (EC 3.4.11.5) (PIP) (Prolyl aminopeptidase) (PAP) |
| contig_1508 | 427 | 11 | - | NADH-dependent butanol dehydrogenase A (EC 1.1.1.-) |
| contig_1508 | 984 | 499 | - | NADH-dependent butanol dehydrogenase A (EC 1.1.1.-) |
| contig_1509 | 317 | 54 | - | thiJ/pfpI family protein |
| contig_1509 | 712 | 834 | + | Leucine-responsive regulatory protein, regulator for leucine (or lrp) regulon and high-affinity branched-chain amino acid transport system |
| contig_1509 | 1527 | 3032 | + | Gluconokinase (EC 2.7.1.12) |
| contig_1509 | 3045 | 3683 | + | Gluconate transporter family protein |
| contig_1509 | 3887 | 4429 | + | Gluconate transporter family protein |
| contig_1509 | 4484 | 5332 | + | 6-phosphogluconate dehydrogenase, decarboxylating (EC 1.1.1.44) |
| contig_1510 | 426 | 313 | - | Phage T7 exclusion protein |
| contig_1510 | 745 | 545 | - | Phage T7 exclusion protein |
| contig_1511 | 527 | 414 | - | Microbial collagenase (EC 3.4.24.3) |
| contig_1511 | 1089 | 493 | - | Microbial collagenase (EC 3.4.24.3) |
| contig_1511 | 1379 | 1855 | + | Cell wall hydrolase |
| contig_1511 | 1833 | 2081 | + | Cell wall hydrolase |
| contig_1511 | 2038 | 2178 | + | Cell wall hydrolase |
| contig_1512 | 491 | 183 | - | site-specific recombinase, phage integrase family |
| contig_1512 | 841 | 548 | - | site-specific recombinase, phage integrase family |
| contig_1512 | 1186 | 869 | - | sensor histidine kinase |
| contig_1514 | 354 | 139 | - | Serine/threonine protein kinases |
| contig_1514 | 505 | 317 | - | Serine/threonine protein kinases |
| contig_1514 | 666 | 508 | - | Serine/threonine protein kinases |
| contig_1517 | 251 | 21 | - | Mobile element protein |
| contig_1519 | 514 | 119 | - | LPXTG-motif cell wall anchor domain protein |
| contig_152 | 309 | 181 | - | Transcriptional regulator, DeoR family |
| contig_152 | 1121 | 348 | - | Transcriptional regulator, DeoR family |
| contig_152 | 1321 | 1434 | + | sugar-binding transcriptional regulator, LacI family |
| contig_152 | 1510 | 1959 | + | sugar-binding transcriptional regulator, LacI family |
| contig_1520 | 63 | 326 | + | FIG01231026: hypothetical protein |
| contig_1520 | 345 | 614 | + | FIG01226421: hypothetical protein |
| contig_1521 | 940 | 149 | - | Cardiolipin synthetase (EC 2.7.8.-) |
| contig_1522 | 27 | 224 | + | FIG01228102: hypothetical protein |
| contig_1522 | 1190 | 534 | - | Multidrug ABC transporter, permease |
| contig_1522 | 1802 | 1680 | - | Streptolysin S export transmembrane permease (SagH) |
| contig_1522 | 1908 | 1780 | - | Streptolysin S export transmembrane permease (SagH) |
| contig_1523 | 328 | 176 | - | Sensor histidine kinase |
| contig_1523 | 525 | 373 | - | Sensor histidine kinase |
| contig_1523 | 705 | 592 | - | Sensor histidine kinase |
| contig_1523 | 952 | 1317 | + | FIG00743012: hypothetical protein |
| contig_1523 | 1603 | 1466 | - | aldehyde dehydrogenase family protein |
| contig_1523 | 2891 | 1593 | - | aldehyde dehydrogenase family protein |
| contig_1523 | 3461 | 3024 | - | Magnesium and cobalt efflux protein CorC |
| contig_1523 | 3487 | 3888 | + | hypothetical protein |
| contig_1523 | 4141 | 3971 | - | Magnesium and cobalt efflux protein CorC |
| contig_1524 | 298 | 984 | + | FIG01226960: hypothetical protein |
| contig_1525 | 110 | 247 | + | Phage tail length tape-measure protein |
| contig_1526 | 395 | 114 | - | Recombinational DNA repair protein RecT (prophage associated) |
| contig_1527 | 125 | 646 | + | cell wall-associated protein |
| contig_1528 | 1544 | 405 | - | Histidyl-tRNA synthetase, archaeal-type paralog (EC 6.1.1.21) |
| contig_1529 | 489 | 373 | - | hypothetical protein |
| contig_153 | 744 | 442 | - | FIG01227184: hypothetical protein |
| contig_153 | 1619 | 1434 | - | acetyltransferase, GNAT family |
| contig_153 | 1836 | 1699 | - | acetyltransferase, GNAT family |
| contig_153 | 2381 | 1977 | - | Arsenate reductase (EC 1.20.4.1) |
| contig_153 | 3078 | 2407 | - | Arsenical-resistance protein ACR3 |
| contig_1531 | 175 | 768 | + | Beta-galactosidase (EC 3.2.1.23) |
| contig_1531 | 803 | 967 | + | Beta-galactosidase (EC 3.2.1.23) |
| contig_1532 | 57 | 182 | + | FIG01225131: hypothetical protein |
| contig_1532 | 656 | 234 | - | Holin associated hypothetical protein |
| contig_1532 | 859 | 1170 | + | Oxidoreductase ucpA (EC 1.-.-.-) |
| contig_1532 | 1160 | 1369 | + | Oxidoreductase ucpA (EC 1.-.-.-) |
| contig_1532 | 1348 | 1656 | + | Oxidoreductase ucpA (EC 1.-.-.-) |
| contig_1532 | 1879 | 2001 | + | FIG01225811: hypothetical protein |
| contig_1532 | 2170 | 2364 | + | FIG01227199: hypothetical protein |
| contig_1532 | 2361 | 2846 | + | FIG01227199: hypothetical protein |
| contig_1533 | 371 | 117 | - | Cell division protein FtsK |
| contig_1535 | 72 | 203 | + | glycosyl transferase, putative |
| contig_1535 | 221 | 472 | + | glycosyl transferase, putative |
| contig_1535 | 715 | 861 | + | glycosyl transferase, putative |
| contig_1535 | 896 | 1018 | + | hypothetical protein |
| contig_1536 | 19 | 246 | + | FIG001943: hypothetical protein YajQ |
| contig_1537 | 59 | 685 | + | RecA protein |
| contig_1537 | 1133 | 1285 | + | hypothetical protein |
| contig_1537 | 1249 | 1425 | + | RecA protein |
| contig_1538 | 20 | 163 | + | FIG01231443: hypothetical protein |
| contig_1539 | 398 | 165 | - | Preprotein translocase subunit SecG (TC 3.A.5.1.1) |
| contig_1539 | 1080 | 493 | - | CidA-associated membrane protein CidB |
| contig_1539 | 1552 | 1292 | - | Holin-like protein 2 |
| contig_154 | 66 | 248 | + | Transcriptional regulator, MerR family |
| contig_154 | 220 | 537 | + | Transcriptional regulator, MerR family |
| contig_154 | 534 | 995 | + | Integral membrane protein |
| contig_154 | 946 | 1185 | + | Integral membrane protein |
| contig_154 | 1253 | 1378 | + | hypothetical protein |
| contig_154 | 1430 | 2212 | + | Excinuclease ABC subunit B |
| contig_154 | 2209 | 3213 | + | Excinuclease ABC subunit B |
| contig_154 | 3173 | 3409 | + | Excinuclease ABC subunit B |
| contig_154 | 3415 | 6291 | + | Excinuclease ABC subunit A |
| contig_154 | 6351 | 6800 | + | FIG01225272: hypothetical protein |
| contig_154 | 6892 | 7281 | + | Arginine/ornithine antiporter ArcD |
| contig_1544 | 1071 | 106 | - | Immune inhibitor A precursor |
| contig_1546 | 22 | 324 | + | Butyryl-CoA dehydrogenase (EC 1.3.8.1) |
| contig_1546 | 321 | 677 | + | 3-hydroxyisobutyrate dehydrogenase (EC 1.1.1.31) |
| contig_1546 | 793 | 1221 | + | 3-hydroxyisobutyrate dehydrogenase (EC 1.1.1.31) |
| contig_1546 | 1244 | 2047 | + | Methylmalonate-semialdehyde dehydrogenase (EC 1.2.1.27) |
| contig_1546 | 2028 | 2402 | + | Methylmalonate-semialdehyde dehydrogenase (EC 1.2.1.27) |
| contig_1547 | 251 | 60 | - | ABC transporter, ATP-binding protein |
| contig_1548 | 264 | 34 | - | Lipase, alpha/beta hydrolase fold family (EC 3.1.1.3) |
| contig_1549 | 238 | 789 | + | membrane protein, putative |
| contig_1549 | 793 | 912 | + | membrane protein, putative |
| contig_1549 | 1354 | 971 | - | Lipoteichoic acid synthase LtaS Type Ia |
| contig_1549 | 1567 | 1451 | - | hypothetical protein |
| contig_155 | 830 | 213 | - | RNA-binding protein Jag |
| contig_155 | 1567 | 827 | - | Inner membrane protein translocase component YidC, short form OxaI-like |
| contig_155 | 1809 | 1648 | - | Ribonuclease P protein component (EC 3.1.26.5) |
| contig_155 | 1996 | 1775 | - | Ribonuclease P protein component (EC 3.1.26.5) |
| contig_155 | 3098 | 4111 | + | Chromosomal replication initiator protein DnaA |
| contig_155 | 4339 | 4560 | + | DNA polymerase III beta subunit (EC 2.7.7.7) |
| contig_155 | 4595 | 5428 | + | DNA polymerase III beta subunit (EC 2.7.7.7) |
| contig_155 | 5554 | 5766 | + | FIG002958: hypothetical protein |
| contig_1550 | 337 | 158 | - | 2-oxoglutarate oxidoreductase, beta subunit (EC 1.2.7.3) |
| contig_1550 | 531 | 334 | - | 2-oxoglutarate oxidoreductase, beta subunit (EC 1.2.7.3) |
| contig_1550 | 958 | 509 | - | 2-oxoglutarate oxidoreductase, beta subunit (EC 1.2.7.3) |
| contig_1550 | 1128 | 1015 | - | 2-oxoglutarate oxidoreductase, alpha subunit (EC 1.2.7.3) |
| contig_1550 | 1403 | 1146 | - | 2-oxoglutarate oxidoreductase, alpha subunit (EC 1.2.7.3) |
| contig_1550 | 1825 | 1580 | - | 2-oxoglutarate oxidoreductase, alpha subunit (EC 1.2.7.3) |
| contig_1551 | 386 | 246 | - | Alkaline phosphatase (EC 3.1.3.1) |
| contig_1551 | 511 | 389 | - | Alkaline phosphatase (EC 3.1.3.1) |
| contig_1551 | 668 | 489 | - | Alkaline phosphatase (EC 3.1.3.1) |
| contig_1551 | 1218 | 688 | - | Alkaline phosphatase (EC 3.1.3.1) |
| contig_1551 | 1538 | 1699 | + | putative membrane protein |
| contig_1552 | 158 | 9 | - | L-Proline/Glycine betaine transporter ProP |
| contig_1552 | 401 | 174 | - | Transcriptional regulator, MarR family |
| contig_1553 | 52 | 435 | + | FIG01229320: hypothetical protein |
| contig_1556 | 35 | 685 | + | Cell surface protein IsdA, transfers heme from hemoglobin to apo-IsdC |
| contig_1556 | 985 | 1098 | + | Heme transporter IsdDEF, lipoprotein IsdE |
| contig_1556 | 1167 | 1466 | + | Heme transporter IsdDEF, lipoprotein IsdE |
| contig_1556 | 1599 | 1871 | + | Heme transporter IsdDEF, lipoprotein IsdE |
| contig_1556 | 1899 | 2882 | + | Heme transporter IsdDEF, permease component IsdF |
| contig_1556 | 2872 | 3207 | + | Heme transporter analogous to IsdDEF, ATP-binding protein |
| contig_1556 | 3164 | 3643 | + | Heme transporter analogous to IsdDEF, ATP-binding protein |
| contig_1557 | 1271 | 804 | - | Thiazole biosynthesis protein ThiG |
| contig_1557 | 1577 | 1320 | - | Thiazole biosynthesis protein ThiG |
| contig_1558 | 862 | 176 | - | FIG01229057: hypothetical protein |
| contig_1558 | 1471 | 1040 | - | FIG01229057: hypothetical protein |
| contig_1559 | 188 | 307 | + | hypothetical protein |
| contig_1559 | 372 | 584 | + | Spore germination protein GerSC |
| contig_1559 | 559 | 1086 | + | Spore germination protein GerSC |
| contig_1559 | 1064 | 1189 | + | Spore germination protein GerSC |
| contig_1559 | 1403 | 1269 | - | hypothetical protein |
| contig_1559 | 2909 | 1461 | - | Chromosome segregation ATPases |
| contig_1559 | 3450 | 3007 | - | Chromosome segregation ATPases |
| contig_1559 | 3611 | 3495 | - | Chromosome segregation ATPases |
| contig_1559 | 3714 | 3598 | - | Chromosome segregation ATPases |
| contig_1559 | 4045 | 3704 | - | Chromosome segregation ATPases |
| contig_156 | 517 | 317 | - | Pyridoxamine 5'-phosphate oxidase (EC 1.4.3.5) |
| contig_156 | 660 | 1613 | + | aminopeptidase |
| contig_156 | 1654 | 1776 | + | aminopeptidase |
| contig_156 | 2245 | 1811 | - | lipoprotein, putative |
| contig_156 | 2374 | 2261 | - | FIG01233788: hypothetical protein |
| contig_156 | 2844 | 2440 | - | FIG01225926: hypothetical protein |
| contig_156 | 3114 | 2947 | - | Protein export cytoplasm protein SecA ATPase RNA helicase (TC 3.A.5.1.1) |
| contig_156 | 3413 | 3072 | - | Protein export cytoplasm protein SecA ATPase RNA helicase (TC 3.A.5.1.1) |
| contig_156 | 3761 | 3510 | - | Ribosomal-protein-L7p-serine acetyltransferase |
| contig_156 | 3961 | 3758 | - | Ribosomal-protein-L7p-serine acetyltransferase |
| contig_156 | 4337 | 4023 | - | membrane protein, putative |
| contig_156 | 4927 | 4388 | - | membrane protein, putative |
| contig_156 | 5331 | 4924 | - | transcriptional regulator, MerR family |
| contig_156 | 5537 | 5821 | + | Transcriptional regulator, PadR family |
| contig_156 | 5793 | 6011 | + | FIG01225992: hypothetical protein |
| contig_156 | 6164 | 6454 | + | FIG01225992: hypothetical protein |
| contig_156 | 6891 | 6508 | - | UDP-N-acetylmuramate--alanine ligase (EC 6.3.2.8) |
| contig_156 | 7821 | 6979 | - | UDP-N-acetylmuramate--alanine ligase (EC 6.3.2.8) |
| contig_156 | 9192 | 8074 | - | Nicotinate phosphoribosyltransferase (EC 2.4.2.11) |
| contig_156 | 10843 | 9254 | - | Cell division protein FtsK |
| contig_1560 | 161 | 48 | - | FIG01230700: hypothetical protein |
| contig_1561 | 696 | 538 | - | FIG01226300: hypothetical protein |
| contig_1561 | 905 | 780 | - | FIG01226300: hypothetical protein |
| contig_1562 | 72 | 500 | + | Oligopeptide ABC transporter, periplasmic oligopeptide-binding protein OppA (TC 3.A.1.5.1) |
| contig_1563 | 530 | 57 | - | Isoleucyl-tRNA synthetase (EC 6.1.1.5) |
| contig_1563 | 1306 | 608 | - | Isoleucyl-tRNA synthetase (EC 6.1.1.5) |
| contig_1563 | 1391 | 1269 | - | Isoleucyl-tRNA synthetase (EC 6.1.1.5) |
| contig_1563 | 1645 | 1439 | - | Isoleucyl-tRNA synthetase (EC 6.1.1.5) |
| contig_1563 | 1820 | 1620 | - | Isoleucyl-tRNA synthetase (EC 6.1.1.5) |
| contig_1563 | 2408 | 1833 | - | Isoleucyl-tRNA synthetase (EC 6.1.1.5) |
| contig_1565 | 199 | 29 | - | RNA-binding protein Hfq |
| contig_1565 | 1179 | 427 | - | tRNA dimethylallyltransferase (EC 2.5.1.75) |
| contig_1565 | 1362 | 1571 | + | BRCA1 |
| contig_1566 | 861 | 745 | - | hypothetical protein |
| contig_1566 | 1073 | 858 | - | FIG01239590: hypothetical protein |
| contig_1566 | 2471 | 1332 | - | FIG01239590: hypothetical protein |
| contig_1566 | 2578 | 2462 | - | ABC transporter, ATP-binding protein |
| contig_1567 | 212 | 373 | + | Leucine-responsive regulatory protein, regulator for leucine (or lrp) regulon and high-affinity branched-chain amino acid transport system |
| contig_1568 | 439 | 603 | + | Transcriptional regulator, AraC family |
| contig_1568 | 605 | 754 | + | hypothetical protein |
| contig_1568 | 1126 | 1275 | + | Bacillolysin (EC 3.4.24.28) |
| contig_1571 | 47 | 523 | + | Integral membrane protein |
| contig_1571 | 1075 | 560 | - | FIG01225487: hypothetical protein |
| contig_1572 | 150 | 37 | - | Electron transfer flavoprotein, beta subunit |
| contig_1572 | 586 | 119 | - | Electron transfer flavoprotein, beta subunit |
| contig_1572 | 1441 | 665 | - | Enoyl-CoA hydratase (EC 4.2.1.17) |
| contig_1572 | 1989 | 1453 | - | Fatty acid degradation regulator YsiA, TetR family |
| contig_1573 | 402 | 73 | - | Penicillin-binding protein 3 |
| contig_1573 | 612 | 427 | - | hypothetical protein |
| contig_1573 | 811 | 680 | - | Penicillin-binding protein 3 |
| contig_1575 | 541 | 257 | - | Transcriptional regulator, AraC family |
| contig_1575 | 888 | 769 | - | acetyltransferase, GNAT family |
| contig_1575 | 1117 | 872 | - | acetyltransferase, GNAT family |
| contig_1576 | 968 | 633 | - | FIG01227825: hypothetical protein |
| contig_1577 | 48 | 176 | + | hypothetical protein |
| contig_1577 | 604 | 314 | - | COG1565: Uncharacterized conserved protein |
| contig_1577 | 761 | 621 | - | COG1565: Uncharacterized conserved protein |
| contig_1577 | 967 | 746 | - | COG1565: Uncharacterized conserved protein |
| contig_1579 | 262 | 435 | + | NADPH-dependent glutamate synthase beta chain and related oxidoreductases |
| contig_1579 | 439 | 813 | + | NADPH-dependent glutamate synthase beta chain and related oxidoreductases |
| contig_158 | 683 | 123 | - | FIG01227158: hypothetical protein |
| contig_158 | 1989 | 787 | - | Prolyl oligopeptidase family (Peptidase_S9) (EC 3.4.21.26) |
| contig_158 | 2125 | 1949 | - | Prolyl oligopeptidase family (Peptidase_S9) (EC 3.4.21.26) |
| contig_158 | 2243 | 2088 | - | Prolyl oligopeptidase family (Peptidase_S9) (EC 3.4.21.26) |
| contig_158 | 2748 | 2230 | - | Prolyl oligopeptidase family (Peptidase_S9) (EC 3.4.21.26) |
| contig_1580 | 259 | 8 | - | Cardiolipin synthetase (EC 2.7.8.-) |
| contig_1581 | 247 | 74 | - | Tagatose-6-phosphate kinase (EC 2.7.1.144) / 1-phosphofructokinase (EC 2.7.1.56) |
| contig_1583 | 390 | 31 | - | N-acetylglucosamine-6-phosphate deacetylase (EC 3.5.1.25) |
| contig_1583 | 576 | 421 | - | Galactosamine-6-phosphate isomerase (EC 5.3.1.-) |
| contig_1585 | 182 | 48 | - | ATP-dependent Clp protease proteolytic subunit (EC 3.4.21.92) |
| contig_1586 | 182 | 48 | - | ATP-dependent Clp protease proteolytic subunit (EC 3.4.21.92) |
| contig_1587 | 256 | 62 | - | 4-oxalocrotonate decarboxylase (EC 4.1.1.77) |
| contig_1587 | 411 | 253 | - | 4-oxalocrotonate decarboxylase (EC 4.1.1.77) |
| contig_1587 | 524 | 402 | - | hypothetical protein |
| contig_1587 | 721 | 521 | - | 4-hydroxy-2-oxovalerate aldolase (EC 4.1.3.39) |
| contig_1588 | 745 | 41 | - | Poly(glycerophosphate chain) D-alanine transfer protein DltD |
| contig_1588 | 981 | 742 | - | D-alanine--poly(phosphoribitol) ligase subunit 2 (EC 6.1.1.13) |
| contig_1588 | 1539 | 1180 | - | D-alanyl transfer protein DltB |
| contig_1588 | 2218 | 1541 | - | D-alanyl transfer protein DltB |
| contig_1588 | 2377 | 2228 | - | D-alanine--poly(phosphoribitol) ligase subunit 1 (EC 6.1.1.13) |
| contig_1588 | 3440 | 2343 | - | D-alanine--poly(phosphoribitol) ligase subunit 1 (EC 6.1.1.13) |
| contig_1588 | 3571 | 3449 | - | D-alanine--poly(phosphoribitol) ligase subunit 1 (EC 6.1.1.13) |
| contig_1589 | 17 | 292 | + | Uncharacterized protein YppC |
| contig_1589 | 351 | 584 | + | Uncharacterized protein YppC |
| contig_1589 | 907 | 794 | - | FIG01229128: hypothetical protein |
| contig_1589 | 1039 | 1155 | + | FIG01226565: hypothetical protein |
| contig_1589 | 1109 | 1252 | + | FIG01226565: hypothetical protein |
| contig_1589 | 1333 | 1470 | + | FIG01226164: hypothetical protein |
| contig_1589 | 1454 | 1600 | + | FIG01226164: hypothetical protein |
| contig_1589 | 1815 | 1636 | - | FIG01225233: hypothetical protein |
| contig_1589 | 2147 | 2347 | + | Spore coat protein |
| contig_159 | 1367 | 180 | - | DNA gyrase subunit A (EC 5.99.1.3) |
| contig_159 | 2433 | 1339 | - | DNA gyrase subunit A (EC 5.99.1.3) |
| contig_159 | 2674 | 2522 | - | DNA gyrase subunit B (EC 5.99.1.3) |
| contig_159 | 3279 | 2761 | - | DNA gyrase subunit B (EC 5.99.1.3) |
| contig_159 | 4444 | 3257 | - | DNA gyrase subunit B (EC 5.99.1.3) |
| contig_159 | 5022 | 4483 | - | DNA recombination and repair protein RecF |
| contig_159 | 5148 | 5026 | - | DNA recombination and repair protein RecF |
| contig_1591 | 510 | 854 | + | membrane protein, putative |
| contig_1591 | 838 | 1143 | + | membrane protein, putative |
| contig_1592 | 499 | 377 | - | Tryptophan 2,3-dioxygenase (EC 1.13.11.11) |
| contig_1592 | 1300 | 842 | - | Kynurenine formamidase, bacterial (EC 3.5.1.9) |
| contig_1592 | 1759 | 1580 | - | Kynureninase (EC 3.7.1.3) |
| contig_1592 | 2068 | 1817 | - | Kynureninase (EC 3.7.1.3) |
| contig_1592 | 2249 | 2028 | - | Kynureninase (EC 3.7.1.3) |
| contig_1594 | 130 | 11 | - | hypothetical protein |
| contig_1595 | 428 | 12 | - | putative esterase |
| contig_1595 | 816 | 559 | - | D-tyrosyl-tRNA(Tyr) deacylase (EC 3.6.1.n1) |
| contig_1597 | 287 | 153 | - | Sensor histidine kinase (EC 2.7.3.-) |
| contig_1597 | 1062 | 262 | - | Sensor histidine kinase (EC 2.7.3.-) |
| contig_1598 | 652 | 14 | - | Transcriptional antiterminator of lichenan operon, BglG family |
| contig_1598 | 1605 | 643 | - | Transcriptional antiterminator of lichenan operon, BglG family |
| contig_1599 | 42 | 191 | + | Predicted glycolate dehydrogenase, 2-subunit type (EC 1.1.99.14), iron-sulfur subunit GlcD |
| contig_1599 | 188 | 865 | + | Predicted glycolate dehydrogenase, 2-subunit type (EC 1.1.99.14), iron-sulfur subunit GlcF |
| contig_1599 | 1209 | 1520 | + | Predicted glycolate dehydrogenase, 2-subunit type (EC 1.1.99.14), iron-sulfur subunit GlcF |
| contig_16 | 700 | 389 | - | Transketolase (EC 2.2.1.1) |
| contig_16 | 1807 | 1040 | - | Phosphonate ABC transporter permease protein phnE1 (TC 3.A.1.9.1) |
| contig_16 | 2496 | 1807 | - | Phosphonate ABC transporter permease protein phnE2 (TC 3.A.1.9.1) |
| contig_16 | 2816 | 2598 | - | Phosphonate ABC transporter ATP-binding protein (TC 3.A.1.9.1) |
| contig_16 | 2955 | 2797 | - | Phosphonate ABC transporter ATP-binding protein (TC 3.A.1.9.1) |
| contig_160 | 1226 | 996 | - | FIG01226722: hypothetical protein |
| contig_160 | 1501 | 1223 | - | FIG01226722: hypothetical protein |
| contig_160 | 1680 | 1543 | - | FIG01226635: hypothetical protein |
| contig_160 | 2812 | 1985 | - | DinG family ATP-dependent helicase YoaA |
| contig_160 | 3128 | 2802 | - | DinG family ATP-dependent helicase YoaA |
| contig_160 | 3616 | 3248 | - | DinG family ATP-dependent helicase YoaA |
| contig_160 | 3950 | 3582 | - | DinG family ATP-dependent helicase YoaA |
| contig_160 | 4195 | 4007 | - | DinG family ATP-dependent helicase YoaA |
| contig_160 | 4395 | 4246 | - | DinG family ATP-dependent helicase YoaA |
| contig_160 | 4523 | 4401 | - | DinG family ATP-dependent helicase YoaA |
| contig_160 | 4755 | 4618 | - | DinG family ATP-dependent helicase YoaA |
| contig_160 | 5268 | 4885 | - | Aspartate 1-decarboxylase (EC 4.1.1.11) |
| contig_160 | 5787 | 5281 | - | Pantoate--beta-alanine ligase (EC 6.3.2.1) |
| contig_160 | 6131 | 5808 | - | Pantoate--beta-alanine ligase (EC 6.3.2.1) |
| contig_160 | 6526 | 6131 | - | 3-methyl-2-oxobutanoate hydroxymethyltransferase (EC 2.1.2.11) |
| contig_160 | 6941 | 6495 | - | 3-methyl-2-oxobutanoate hydroxymethyltransferase (EC 2.1.2.11) |
| contig_1600 | 22 | 147 | + | sensor histidine kinase |
| contig_1600 | 212 | 517 | + | sensor histidine kinase |
| contig_1600 | 477 | 698 | + | sensor histidine kinase |
| contig_1602 | 330 | 193 | - | Probable 5'-3' exonuclease Bsu YpcP |
| contig_1602 | 873 | 409 | - | Probable 5'-3' exonuclease Bsu YpcP |
| contig_1602 | 1061 | 885 | - | Probable 5'-3' exonuclease Bsu YpcP |
| contig_1602 | 1746 | 1189 | - | Cation transporter, putative |
| contig_1603 | 6 | 149 | + | Predicted tyrosine transporter, NhaC family |
| contig_1603 | 112 | 249 | + | Predicted tyrosine transporter, NhaC family |
| contig_1603 | 372 | 509 | + | Predicted tyrosine transporter, NhaC family |
| contig_1604 | 296 | 649 | + | MFS general substrate transporter |
| contig_1604 | 702 | 827 | + | MFS general substrate transporter |
| contig_1605 | 284 | 54 | - | FIG00458121: hypothetical protein |
| contig_1606 | 175 | 62 | - | hypothetical protein |
| contig_1607 | 128 | 826 | + | Glutamate transport membrane-spanning protein |
| contig_1607 | 1193 | 1429 | + | Arginine permease RocE |
| contig_1607 | 1519 | 2403 | + | Arginine permease RocE |
| contig_1607 | 2378 | 2578 | + | OsmC/Ohr family protein |
| contig_1607 | 2559 | 2756 | + | OsmC/Ohr family protein |
| contig_1607 | 2941 | 3150 | + | HIT family protein |
| contig_1608 | 1546 | 170 | - | UDP-N-acetylmuramoylalanyl-D-glutamyl-2,6-diaminopimelate--D-alanyl-D-alanine ligase (EC 6.3.2.10) |
| contig_161 | 408 | 13 | - | S-adenosylmethionine decarboxylase proenzyme (EC 4.1.1.50), prokaryotic class 1B |
| contig_1610 | 520 | 296 | - | Polysaccharide deacetylase |
| contig_1611 | 97 | 225 | + | hypothetical protein |
| contig_1613 | 264 | 1 | - | UDP-N-acetylglucosamine 4,6-dehydratase (EC 4.2.1.-) |
| contig_1614 | 1143 | 253 | - | Arsenic efflux pump protein |
| contig_1615 | 18 | 173 | + | FIG01225309: hypothetical protein |
| contig_1615 | 422 | 183 | - | Predicted regulator PutR for proline utilization, GntR family |
| contig_1615 | 630 | 454 | - | Predicted regulator PutR for proline utilization, GntR family |
| contig_1615 | 904 | 635 | - | Predicted regulator PutR for proline utilization, GntR family |
| contig_1615 | 1373 | 921 | - | transporter, EamA family |
| contig_1617 | 535 | 416 | - | FIG01228233: hypothetical protein |
| contig_1619 | 627 | 16 | - | Phosphoglycerate mutase family 1 |
| contig_1619 | 888 | 640 | - | adh_short, short chain dehydrogenase |
| contig_162 | 307 | 486 | + | transcriptional regulator, Bla/Mec family |
| contig_162 | 877 | 704 | - | FIG01227471: hypothetical protein |
| contig_162 | 1136 | 948 | - | Multi antimicrobial extrusion protein (Na(+)/drug antiporter), MATE family of MDR efflux pumps |
| contig_1620 | 203 | 54 | - | 2-methylcitrate synthase (EC 2.3.3.5) |
| contig_1620 | 730 | 257 | - | 2-methylcitrate synthase (EC 2.3.3.5) |
| contig_1621 | 184 | 801 | + | Na+/H+ antiporter |
| contig_1622 | 554 | 165 | - | Permease of the drug/metabolite transporter (DMT) superfamily |
| contig_1623 | 14 | 457 | + | FIG001454: Transglutaminase-like enzymes, putative cysteine proteases |
| contig_1623 | 634 | 783 | + | FIG001454: Transglutaminase-like enzymes, putative cysteine proteases |
| contig_1626 | 1113 | 745 | - | FIG01237015: hypothetical protein |
| contig_1626 | 1220 | 1609 | + | General stress protein 17M |
| contig_1626 | 1652 | 1786 | + | FIG01226778: hypothetical protein |
| contig_1626 | 1921 | 2172 | + | FIG01226018: hypothetical protein |
| contig_1626 | 2553 | 2353 | - | FIG01228408: hypothetical protein |
| contig_1627 | 477 | 238 | - | FIG00267293: hypothetical protein |
| contig_1629 | 10 | 153 | + | oxidoreductase, aldo/keto reductase family |
| contig_1629 | 792 | 394 | - | acetyltransferase, GNAT family |
| contig_1629 | 1455 | 868 | - | Tryptophan 2-monooxygenase (EC 1.13.12.3) |
| contig_1629 | 2138 | 2025 | - | Tryptophan 2-monooxygenase (EC 1.13.12.3) |
| contig_1629 | 2337 | 2140 | - | Tryptophan 2-monooxygenase (EC 1.13.12.3) |
| contig_1629 | 2532 | 2723 | + | FIG01226883: hypothetical protein |
| contig_1629 | 3062 | 3529 | + | Coenzyme F420-dependent oxidoreductase |
| contig_1629 | 3684 | 3866 | + | Coenzyme F420-dependent oxidoreductase |
| contig_163 | 193 | 71 | - | Multimodular transpeptidase-transglycosylase (EC 2.4.1.129) (EC 3.4.-.-) |
| contig_163 | 992 | 201 | - | Multimodular transpeptidase-transglycosylase (EC 2.4.1.129) (EC 3.4.-.-) |
| contig_163 | 1173 | 1000 | - | Multimodular transpeptidase-transglycosylase (EC 2.4.1.129) (EC 3.4.-.-) |
| contig_163 | 2038 | 1205 | - | Multimodular transpeptidase-transglycosylase (EC 2.4.1.129) (EC 3.4.-.-) |
| contig_163 | 2178 | 2050 | - | Multimodular transpeptidase-transglycosylase (EC 2.4.1.129) (EC 3.4.-.-) |
| contig_163 | 2619 | 2257 | - | Glycerophosphoryl diester phosphodiesterase (EC 3.1.4.46) |
| contig_163 | 2984 | 2739 | - | Glycerophosphoryl diester phosphodiesterase (EC 3.1.4.46) |
| contig_163 | 3291 | 3545 | + | Transcriptional regulator, MerR family |
| contig_163 | 3551 | 4090 | + | multidrug resistance protein, putative |
| contig_163 | 4087 | 4266 | + | multidrug resistance protein, putative |
| contig_163 | 4360 | 4719 | + | multidrug resistance protein, putative |
| contig_163 | 4863 | 5711 | + | Spermidine synthase (EC 2.5.1.16) |
| contig_163 | 5929 | 6801 | + | Agmatinase (EC 3.5.3.11) |
| contig_163 | 7581 | 6979 | - | Perfringolysin O regulator protein PfoR |
| contig_1630 | 442 | 609 | + | Branched-chain amino acid transport system carrier protein |
| contig_1631 | 8 | 187 | + | FIG01238213: hypothetical protein |
| contig_1631 | 454 | 269 | - | Phosphonate ABC transporter phosphate-binding periplasmic component (TC 3.A.1.9.1) |
| contig_1631 | 665 | 405 | - | Phosphonate ABC transporter phosphate-binding periplasmic component (TC 3.A.1.9.1) |
| contig_1631 | 1023 | 1259 | + | FIG01227906: hypothetical protein |
| contig_1631 | 1300 | 1689 | + | FIG01227906: hypothetical protein |
| contig_1631 | 1964 | 1812 | - | FIG01225806: hypothetical protein |
| contig_1631 | 2145 | 1942 | - | FIG01225806: hypothetical protein |
| contig_1634 | 325 | 909 | + | Cell surface protein IsdA, transfers heme from hemoglobin to apo-IsdC |
| contig_1634 | 903 | 1028 | + | Cell surface protein IsdA, transfers heme from hemoglobin to apo-IsdC |
| contig_1635 | 237 | 749 | + | Malate Na(+) symporter |
| contig_1635 | 772 | 888 | + | Malate Na(+) symporter |
| contig_1635 | 872 | 1141 | + | Malate Na(+) symporter |
| contig_1636 | 1223 | 1083 | - | DNA topoisomerase III (EC 5.99.1.2) |
| contig_1637 | 109 | 1044 | + | Integral membrane protein |
| contig_164 | 925 | 260 | - | Ribosomal RNA small subunit methyltransferase C (EC 2.1.1.52) |
| contig_164 | 1319 | 960 | - | LSU ribosomal protein L7/L12 (P1/P2) |
| contig_164 | 1887 | 1387 | - | LSU ribosomal protein L10p (P0) |
| contig_164 | 2812 | 2120 | - | LSU ribosomal protein L1p (L10Ae) |
| contig_164 | 3416 | 2991 | - | LSU ribosomal protein L11p (L12e) |
| contig_164 | 4034 | 3585 | - | Transcription antitermination protein NusG |
| contig_1640 | 314 | 637 | + | Arsenate reductase (EC 1.20.4.1) |
| contig_1640 | 719 | 1102 | + | Glycine cleavage system H protein |
| contig_1640 | 1362 | 1231 | - | FIG01229090: hypothetical protein |
| contig_1640 | 1430 | 1744 | + | Toprim domain protein |
| contig_1641 | 396 | 635 | + | unknown |
| contig_1642 | 435 | 178 | - | Glutamyl-tRNA reductase (EC 1.2.1.70) |
| contig_1642 | 1027 | 464 | - | Glutamyl-tRNA reductase (EC 1.2.1.70) |
| contig_1642 | 1516 | 1145 | - | Glutamyl-tRNA reductase (EC 1.2.1.70) |
| contig_1643 | 75 | 206 | + | Cell division protein FtsI [Peptidoglycan synthetase] (EC 2.4.1.129) |
| contig_1643 | 218 | 586 | + | Cell division protein FtsI [Peptidoglycan synthetase] (EC 2.4.1.129) |
| contig_1643 | 586 | 843 | + | Cell division protein FtsI [Peptidoglycan synthetase] (EC 2.4.1.129) |
| contig_1646 | 484 | 215 | - | Extracellular protein |
| contig_1646 | 723 | 1160 | + | Peptidyl-prolyl cis-trans isomerase (EC 5.2.1.8) |
| contig_1646 | 1161 | 1277 | + | hypothetical protein |
| contig_1646 | 1678 | 1920 | + | RibT protein |
| contig_1646 | 1927 | 2058 | + | hypothetical protein |
| contig_1646 | 2218 | 2096 | - | DUF309 domain-containing protein |
| contig_1647 | 68 | 1294 | + | hypothetical protein |
| contig_1647 | 1331 | 1504 | + | hypothetical protein |
| contig_1647 | 1473 | 1748 | + | FIG01250146: hypothetical protein |
| contig_1647 | 1832 | 1957 | + | FIG01250146: hypothetical protein |
| contig_1649 | 106 | 723 | + | Transcriptional regulator, TetR family |
| contig_165 | 407 | 84 | - | Stage II sporulation protein P |
| contig_165 | 724 | 452 | - | Stage II sporulation protein P |
| contig_165 | 1064 | 948 | - | acyl-CoA dehydrogenase, short-chain specific |
| contig_165 | 1964 | 1218 | - | Butyryl-CoA dehydrogenase (EC 1.3.8.1) |
| contig_1652 | 392 | 168 | - | FIG139598: Potential ribosomal protein |
| contig_1652 | 736 | 563 | - | LSU ribosomal protein L21p |
| contig_1652 | 872 | 723 | - | LSU ribosomal protein L21p |
| contig_1652 | 1168 | 1040 | - | Cytoplasmic axial filament protein CafA and Ribonuclease G (EC 3.1.4.-) |
| contig_1652 | 1162 | 1284 | + | hypothetical protein |
| contig_1654 | 417 | 22 | - | FIG01227619: hypothetical protein |
| contig_1654 | 551 | 414 | - | hypothetical protein |
| contig_1656 | 880 | 188 | - | Adenylosuccinate synthetase (EC 6.3.4.4) |
| contig_1656 | 1478 | 873 | - | Adenylosuccinate synthetase (EC 6.3.4.4) |
| contig_1657 | 1292 | 879 | - | FIG01226365: hypothetical protein |
| contig_1657 | 1496 | 1305 | - | hypothetical protein |
| contig_1657 | 1664 | 1846 | + | hypothetical protein |
| contig_1658 | 351 | 124 | - | N-methyl-transferase-related protein |
| contig_1658 | 521 | 375 | - | N-methyl-transferase-related protein |
| contig_1658 | 748 | 590 | - | N-methyl-transferase-related protein |
| contig_1658 | 1255 | 1058 | - | btrG family protein |
| contig_1658 | 1385 | 1230 | - | btrG family protein |
| contig_1659 | 52 | 351 | + | Multidrug resistance ABC transporter ATP-binding and permease protein |
| contig_166 | 26 | 322 | + | Alpha-acetolactate decarboxylase (EC 4.1.1.5) |
| contig_1660 | 214 | 71 | - | FIG01163484: hypothetical protein |
| contig_1660 | 826 | 509 | - | FIG01163484: hypothetical protein |
| contig_1660 | 1099 | 986 | - | hypothetical protein |
| contig_1660 | 1981 | 1112 | - | Similar to CDP-glucose 4,6-dehydratase (EC 4.2.1.45) |
| contig_1660 | 2169 | 2008 | - | Similar to CDP-glucose 4,6-dehydratase (EC 4.2.1.45) |
| contig_1660 | 2866 | 2171 | - | Glucose-1-phosphate cytidylyltransferase (EC 2.7.7.33) |
| contig_1661 | 1092 | 877 | - | 1-hydroxy-2-methyl-2-(E)-butenyl 4-diphosphate synthase (EC 1.17.7.1) |
| contig_1662 | 565 | 11 | - | Glycerate kinase (EC 2.7.1.31) |
| contig_1663 | 249 | 91 | - | Phosphonate ABC transporter phosphate-binding periplasmic component (TC 3.A.1.9.1) |
| contig_1663 | 760 | 521 | - | ABC transporter permease protein YvcS |
| contig_1664 | 95 | 574 | + | Sporulation initiation phosphotransferase B (Spo0B) |
| contig_1665 | 591 | 370 | - | Ferredoxin |
| contig_1666 | 582 | 178 | - | PTS system, fructose-specific IIA component (EC 2.7.1.69) / PTS system, fructose-specific IIB component (EC 2.7.1.69) / PTS system, fructose-specific IIC component (EC 2.7.1.69) |
| contig_1666 | 1081 | 572 | - | PTS system, fructose-specific IIA component (EC 2.7.1.69) / PTS system, fructose-specific IIB component (EC 2.7.1.69) / PTS system, fructose-specific IIC component (EC 2.7.1.69) |
| contig_1667 | 271 | 14 | - | hypothetical protein |
| contig_1667 | 469 | 347 | - | FIG01226969: hypothetical protein |
| contig_1667 | 732 | 496 | - | FIG01226969: hypothetical protein |
| contig_1667 | 1160 | 912 | - | FIG014087: hypothetical protein |
| contig_1667 | 1462 | 1334 | - | FIG009439: Cytosolic protein containing multiple CBS domains |
| contig_1667 | 1817 | 1413 | - | FIG009439: Cytosolic protein containing multiple CBS domains |
| contig_1667 | 2052 | 1798 | - | FIG009439: Cytosolic protein containing multiple CBS domains |
| contig_1667 | 2389 | 2021 | - | FIG009439: Cytosolic protein containing multiple CBS domains |
| contig_1667 | 2558 | 2352 | - | FIG009439: Cytosolic protein containing multiple CBS domains |
| contig_1667 | 2892 | 3053 | + | FIG015032: hypothetical protein |
| contig_1667 | 3777 | 3094 | - | FIG002379: metal-dependent hydrolase |
| contig_1667 | 3927 | 4982 | + | Proline dipeptidase (EC 3.4.13.9) |
| contig_1667 | 5468 | 5274 | - | membrane protein, putative |
| contig_1667 | 5749 | 5522 | - | membrane protein, putative |
| contig_1667 | 6083 | 6352 | + | DNA topoisomerase III (EC 5.99.1.2) |
| contig_1667 | 6357 | 6536 | + | DNA topoisomerase III (EC 5.99.1.2) |
| contig_1667 | 6664 | 6864 | + | DNA topoisomerase III (EC 5.99.1.2) |
| contig_1669 | 84 | 596 | + | FIG01225869: hypothetical protein |
| contig_1669 | 970 | 629 | - | Glyoxalase family protein |
| contig_167 | 1310 | 678 | - | Poly(glycerol-phosphate) alpha-glucosyltransferase (EC 2.4.1.52) |
| contig_167 | 1752 | 1318 | - | Poly(glycerol-phosphate) alpha-glucosyltransferase (EC 2.4.1.52) |
| contig_1670 | 648 | 82 | - | Spore germination protein |
| contig_1670 | 1331 | 657 | - | FIG01234092: hypothetical protein |
| contig_1673 | 392 | 93 | - | Flagellar hook-basal body complex protein FliE |
| contig_1673 | 834 | 538 | - | Flagellar basal-body rod protein FlgC |
| contig_1675 | 299 | 21 | - | Niacin transporter NiaP |
| contig_1676 | 168 | 25 | - | Niacin transporter NiaP |
| contig_1677 | 961 | 347 | - | ABC transporter, permease protein |
| contig_1678 | 346 | 83 | - | Long-chain-fatty-acid--CoA ligase (EC 6.2.1.3) |
| contig_1678 | 944 | 309 | - | Long-chain-fatty-acid--CoA ligase (EC 6.2.1.3) |
| contig_1679 | 362 | 45 | - | Succinylornithine transaminase, putative |
| contig_1679 | 661 | 425 | - | Succinylornithine transaminase, putative |
| contig_1679 | 1224 | 886 | - | Succinylornithine transaminase, putative |
| contig_1679 | 1500 | 1748 | + | Catalase (EC 1.11.1.6) |
| contig_1679 | 1798 | 1914 | + | Catalase (EC 1.11.1.6) |
| contig_1679 | 1853 | 2386 | + | Catalase (EC 1.11.1.6) |
| contig_168 | 1166 | 348 | - | minor extracellular protease VpR |
| contig_168 | 1557 | 1324 | - | minor extracellular protease VpR |
| contig_168 | 1760 | 1560 | - | minor extracellular protease VpR |
| contig_1680 | 214 | 98 | - | 5'-nucleotidase (EC 3.1.3.5) |
| contig_1680 | 364 | 248 | - | hypothetical protein |
| contig_1680 | 1078 | 386 | - | 5'-nucleotidase (EC 3.1.3.5) |
| contig_1681 | 227 | 367 | + | FIG01238580: hypothetical protein |
| contig_1682 | 60 | 179 | + | hypothetical protein |
| contig_1684 | 14 | 208 | + | alternate gene name: ipa-20r |
| contig_1684 | 409 | 248 | - | LSU m5C1962 methyltransferase RlmI |
| contig_1684 | 1353 | 478 | - | LSU m5C1962 methyltransferase RlmI |
| contig_1685 | 59 | 274 | + | Prespore specific transcriptional activator RsfA |
| contig_1685 | 389 | 700 | + | Prespore specific transcriptional activator RsfA |
| contig_1686 | 326 | 54 | - | Permease of the drug/metabolite transporter (DMT) superfamily |
| contig_1687 | 177 | 566 | + | putative PQQ enzyme repeat |
| contig_1687 | 934 | 1182 | + | hypothetical protein |
| contig_1687 | 1300 | 1476 | + | hypothetical protein |
| contig_1687 | 1755 | 1961 | + | hypothetical protein |
| contig_1687 | 1976 | 2098 | + | taurine ABC transporter, ATP-binding protein |
| contig_1688 | 288 | 1 | - | FIG01233927: hypothetical protein |
| contig_1688 | 509 | 285 | - | FIG01233927: hypothetical protein |
| contig_1689 | 182 | 571 | + | neutral metalloprotease, putative |
| contig_169 | 102 | 458 | + | hypothetical protein |
| contig_169 | 899 | 1327 | + | FIG01242675: hypothetical protein |
| contig_169 | 1652 | 1353 | - | Leucyl-tRNA synthetase (EC 6.1.1.4) |
| contig_169 | 1825 | 1691 | - | hypothetical protein |
| contig_169 | 3194 | 1803 | - | Leucyl-tRNA synthetase (EC 6.1.1.4) |
| contig_1690 | 103 | 405 | + | potassium uptake protein, TrkH family |
| contig_1691 | 27 | 719 | + | Maltose/maltodextrin ABC transporter, permease protein MalF |
| contig_1691 | 799 | 1329 | + | Maltose/maltodextrin ABC transporter, permease protein MalF |
| contig_1691 | 1330 | 1704 | + | Maltose/maltodextrin ABC transporter, permease protein MalG |
| contig_1691 | 1791 | 1922 | + | Maltose/maltodextrin ABC transporter, permease protein MalG |
| contig_1691 | 1898 | 2170 | + | Maltose/maltodextrin ABC transporter, permease protein MalG |
| contig_1692 | 10 | 126 | + | Proline dehydrogenase (EC 1.5.99.8) (Proline oxidase) |
| contig_1692 | 187 | 354 | + | Proline dehydrogenase (EC 1.5.99.8) (Proline oxidase) |
| contig_1692 | 748 | 377 | - | D-alanyl-D-alanine carboxypeptidase (EC 3.4.16.4) |
| contig_1692 | 863 | 717 | - | D-alanyl-D-alanine carboxypeptidase (EC 3.4.16.4) |
| contig_1692 | 1063 | 938 | - | D-alanyl-D-alanine carboxypeptidase (EC 3.4.16.4) |
| contig_1693 | 487 | 146 | - | Transcriptional regulator, AraC family |
| contig_1693 | 497 | 646 | + | hypothetical protein |
| contig_1693 | 1551 | 751 | - | Thioredoxin reductase (EC 1.8.1.9) |
| contig_1693 | 1747 | 1631 | - | Thioredoxin reductase (EC 1.8.1.9) |
| contig_1694 | 596 | 249 | - | lipoprotein, NLP/P60 family |
| contig_1695 | 217 | 468 | + | FIG002540: Haloacid dehalogenase-like hydrolase |
| contig_1695 | 516 | 665 | + | FIG002540: Haloacid dehalogenase-like hydrolase |
| contig_1695 | 736 | 855 | + | FIG01225827: hypothetical protein |
| contig_1695 | 969 | 844 | - | hypothetical protein |
| contig_1696 | 62 | 202 | + | hypothetical protein |
| contig_1696 | 884 | 507 | - | ErfK/YbiS/YcfS/YnhG family protein |
| contig_1696 | 1417 | 959 | - | ErfK/YbiS/YcfS/YnhG family protein |
| contig_1696 | 1827 | 1417 | - | ErfK/YbiS/YcfS/YnhG family protein |
| contig_1697 | 158 | 706 | + | transcriptional regulator, ArsR family |
| contig_1697 | 804 | 971 | + | Macrolide efflux protein |
| contig_17 | 191 | 1126 | + | FIG01234884: hypothetical protein |
| contig_170 | 165 | 46 | - | Biotin carboxyl carrier protein |
| contig_170 | 417 | 806 | + | citrate transporter, CitM family |
| contig_170 | 772 | 1074 | + | citrate transporter, CitM family |
| contig_170 | 1067 | 1723 | + | citrate transporter, CitM family |
| contig_170 | 2458 | 1766 | - | Transcriptional regulatory protein CitB, DpiA |
| contig_170 | 3030 | 2458 | - | Two-component sensor histidine kinase, malate (EC 2.7.3.-) |
| contig_170 | 3922 | 3044 | - | Two-component sensor histidine kinase, malate (EC 2.7.3.-) |
| contig_1700 | 236 | 12 | - | Nucleoside-diphosphate-sugar epimerases |
| contig_1700 | 650 | 294 | - | Nucleoside-diphosphate-sugar epimerases |
| contig_1700 | 790 | 668 | - | Nucleoside-diphosphate-sugar epimerases |
| contig_1702 | 38 | 241 | + | Ethidium bromide-methyl viologen resistance protein EmrE |
| contig_1702 | 348 | 515 | + | FIG01227962: hypothetical protein |
| contig_1702 | 547 | 666 | + | FIG01227962: hypothetical protein |
| contig_1702 | 1104 | 946 | - | 6-aminohexanoate-dimer hydrolase, putative |
| contig_1702 | 1397 | 1203 | - | 6-aminohexanoate-dimer hydrolase, putative |
| contig_1703 | 873 | 79 | - | penicillin-binding protein, putative |
| contig_1703 | 1066 | 953 | - | hypothetical protein |
| contig_1703 | 1421 | 1023 | - | sensory box/GGDEF family protein, putative |
| contig_1703 | 1879 | 1400 | - | sensory box/GGDEF family protein, putative |
| contig_1706 | 257 | 430 | + | D-alanyl-D-alanine carboxypeptidase (EC 3.4.16.4) |
| contig_1707 | 683 | 432 | - | MutT/nudix family protein |
| contig_1707 | 963 | 808 | - | MutT/nudix family protein |
| contig_1707 | 1269 | 1451 | + | Cephalosporin hydroxylase |
| contig_1707 | 1572 | 1685 | + | Cephalosporin hydroxylase |
| contig_1707 | 2117 | 1821 | - | Signal peptidase I (EC 3.4.21.89) |
| contig_1707 | 2239 | 2114 | - | Signal peptidase I (EC 3.4.21.89) |
| contig_1708 | 149 | 361 | + | NPQTN cell wall anchored protein IsdC |
| contig_1709 | 420 | 160 | - | hypothetical protein |
| contig_1709 | 784 | 536 | - | hypothetical protein |
| contig_1709 | 1010 | 879 | - | hypothetical protein |
| contig_171 | 1127 | 552 | - | Phosphogluconate repressor HexR, RpiR family |
| contig_171 | 1387 | 1265 | - | Beta-lactamase (EC 3.5.2.6) |
| contig_171 | 1583 | 1428 | - | Beta-lactamase (EC 3.5.2.6) |
| contig_171 | 2574 | 1738 | - | Beta-lactamase (EC 3.5.2.6) |
| contig_171 | 2775 | 2650 | - | DNA-binding response regulator, LuxR family |
| contig_171 | 3043 | 2741 | - | DNA-binding response regulator, LuxR family |
| contig_171 | 3224 | 3003 | - | DNA-binding response regulator, LuxR family |
| contig_171 | 3570 | 3427 | - | Nitrate/nitrite sensor protein (EC 2.7.3.-) |
| contig_1710 | 13 | 138 | + | hypothetical protein |
| contig_1710 | 146 | 274 | + | Branched-chain amino acid transport system permease protein LivM (TC 3.A.1.4.1) |
| contig_1710 | 375 | 509 | + | Branched-chain amino acid transport system permease protein LivM (TC 3.A.1.4.1) |
| contig_1710 | 535 | 675 | + | Branched-chain amino acid transport system permease protein LivM (TC 3.A.1.4.1) |
| contig_1710 | 1197 | 751 | - | Chromosome initiation inhibitor |
| contig_1710 | 1665 | 1817 | + | FIG01226153: hypothetical protein |
| contig_1711 | 771 | 100 | - | Unspecified monosaccharide ABC transport system, substrate-binding component |
| contig_1711 | 1365 | 925 | - | Transcriptional regulator in cluster with Zn-dependent hydrolase |
| contig_1714 | 691 | 167 | - | FIG011501: YycH protein |
| contig_1715 | 558 | 343 | - | Mlc, transcriptional repressor of MalT (the transcriptional activator of maltose regulon) and manXYZ operon |
| contig_1715 | 731 | 591 | - | Mlc, transcriptional repressor of MalT (the transcriptional activator of maltose regulon) and manXYZ operon |
| contig_1715 | 1072 | 845 | - | Mlc, transcriptional repressor of MalT (the transcriptional activator of maltose regulon) and manXYZ operon |
| contig_1715 | 1376 | 1242 | - | hypothetical protein |
| contig_1717 | 178 | 41 | - | penicillin-binding protein |
| contig_1717 | 476 | 363 | - | penicillin-binding protein |
| contig_1717 | 816 | 532 | - | penicillin-binding protein |
| contig_1718 | 78 | 359 | + | Zinc ABC transporter, ATP-binding protein ZnuC |
| contig_1718 | 359 | 616 | + | Zinc ABC transporter, inner membrane permease protein ZnuB |
| contig_1719 | 73 | 444 | + | Bacitracin transport permease protein BCRC |
| contig_172 | 1056 | 748 | - | hypothetical protein |
| contig_172 | 1355 | 1164 | - | hypothetical protein |
| contig_172 | 1833 | 1393 | - | Dimeric dUTPase (EC 3.6.1.23) |
| contig_172 | 2081 | 1914 | - | hypothetical protein |
| contig_172 | 2277 | 2092 | - | Glutaredoxin-like protein NrdH, required for reduction of Ribonucleotide reductase class Ib |
| contig_172 | 2601 | 2410 | - | hypothetical protein |
| contig_172 | 2728 | 2594 | - | hypothetical protein |
| contig_172 | 3103 | 2738 | - | FIG01244689: hypothetical protein |
| contig_172 | 3474 | 3145 | - | Phage protein |
| contig_172 | 3616 | 3485 | - | Phage protein |
| contig_172 | 4153 | 3887 | - | Replicative DNA helicase (EC 3.6.1.-) [SA14-24] |
| contig_172 | 5085 | 4855 | - | Replicative DNA helicase (EC 3.6.1.-) [SA14-24] |
| contig_172 | 5367 | 5101 | - | hypothetical protein |
| contig_172 | 6249 | 5683 | - | Phage replication initiation protein |
| contig_172 | 6471 | 6250 | - | FIG01234598: hypothetical protein |
| contig_172 | 6802 | 6641 | - | Recombinational DNA repair protein RecT (prophage associated) |
| contig_172 | 7469 | 6840 | - | Recombinational DNA repair protein RecT (prophage associated) |
| contig_172 | 8418 | 7483 | - | Protein gp47, recombination-related [Bacteriophage A118] |
| contig_172 | 8620 | 8420 | - | FIG01238624: hypothetical protein |
| contig_172 | 8876 | 8613 | - | hypothetical protein |
| contig_1720 | 757 | 506 | - | ATP-dependent DNA helicase rep (EC 3.6.1.-) |
| contig_1720 | 1379 | 813 | - | ATP-dependent DNA helicase rep (EC 3.6.1.-) |
| contig_1721 | 690 | 487 | - | Phosphopantothenoylcysteine synthetase (EC 6.3.2.5) |
| contig_1721 | 926 | 687 | - | Phosphopantothenoylcysteine synthetase (EC 6.3.2.5) |
| contig_1722 | 395 | 252 | - | hypothetical protein |
| contig_1722 | 1261 | 1022 | - | hypothetical protein |
| contig_1723 | 660 | 148 | - | sensor histidine kinase |
| contig_1723 | 1212 | 814 | - | DNA-binding response regulator |
| contig_1723 | 1478 | 1212 | - | DNA-binding response regulator |
| contig_1724 | 236 | 93 | - | Putative transcriptional antiterminator, BglG family / PTS system, mannitol/fructose-specific IIA component (EC 2.7.1.69) |
| contig_1725 | 260 | 120 | - | FIG01228610: hypothetical protein |
| contig_1725 | 641 | 507 | - | FIG01228610: hypothetical protein |
| contig_1728 | 857 | 399 | - | PTS system, fructose-specific IIA component (EC 2.7.1.69) / PTS system, fructose-specific IIB component (EC 2.7.1.69) / PTS system, fructose-specific IIC component (EC 2.7.1.69) |
| contig_1728 | 1089 | 871 | - | 1-phosphofructokinase (EC 2.7.1.56) |
| contig_1728 | 1513 | 1055 | - | 1-phosphofructokinase (EC 2.7.1.56) |
| contig_1728 | 1784 | 1629 | - | 1-phosphofructokinase (EC 2.7.1.56) |
| contig_173 | 284 | 123 | - | hypothetical protein |
| contig_173 | 1050 | 481 | - | thermonuclease family protein, (pXO1-141) |
| contig_1731 | 449 | 243 | - | Stage III sporulation protein D |
| contig_1732 | 116 | 688 | + | Ubiquinone/menaquinone biosynthesis methyltransferase UBIE (EC 2.1.1.-) |
| contig_1732 | 715 | 1014 | + | COG1720: Uncharacterized conserved protein |
| contig_1732 | 1113 | 1304 | + | hypothetical protein |
| contig_1732 | 1400 | 1257 | - | hypothetical protein |
| contig_1733 | 41 | 361 | + | Acetylornithine deacetylase (EC 3.5.1.16) |
| contig_1733 | 371 | 733 | + | Beta-lysine acetyltransferase (EC 2.3.1.-) |
| contig_1734 | 300 | 172 | - | probable membrane protein STY4873 |
| contig_1734 | 523 | 281 | - | probable membrane protein STY4873 |
| contig_1734 | 930 | 610 | - | probable membrane protein STY4873 |
| contig_1734 | 1426 | 920 | - | protein of unknown function DUF445 |
| contig_1734 | 1729 | 1583 | - | D-serine dehydratase (EC 4.3.1.18) |
| contig_1734 | 2127 | 1792 | - | D-serine dehydratase (EC 4.3.1.18) |
| contig_1735 | 281 | 544 | + | Arginine utilization protein RocB |
| contig_1735 | 998 | 672 | - | Pyrroline-5-carboxylate reductase (EC 1.5.1.2) |
| contig_1735 | 1497 | 1132 | - | Pyrroline-5-carboxylate reductase (EC 1.5.1.2) |
| contig_1736 | 383 | 93 | - | Ribonucleotide reductase of class Ib (aerobic), beta subunit (EC 1.17.4.1) |
| contig_1736 | 786 | 370 | - | Ribonucleotide reductase of class Ib (aerobic), beta subunit (EC 1.17.4.1) |
| contig_1736 | 1073 | 750 | - | Ribonucleotide reductase of class Ib (aerobic), beta subunit (EC 1.17.4.1) |
| contig_1736 | 2073 | 1228 | - | Ribonucleotide reductase of class Ib (aerobic), alpha subunit (EC 1.17.4.1) |
| contig_1736 | 2799 | 2560 | - | Ribonucleotide reductase of class Ib (aerobic), alpha subunit (EC 1.17.4.1) |
| contig_174 | 1032 | 373 | - | 2-hydroxy-3-keto-5-methylthiopentenyl-1-phosphate phosphatase |
| contig_174 | 1844 | 1029 | - | 2,3-diketo-5-methylthiopentyl-1-phosphate enolase (EC 5.3.2.5) |
| contig_174 | 2169 | 1966 | - | 2,3-diketo-5-methylthiopentyl-1-phosphate enolase (EC 5.3.2.5) |
| contig_174 | 2761 | 2519 | - | Aliphatic amidase AmiE (EC 3.5.1.4) |
| contig_174 | 3213 | 2797 | - | Aliphatic amidase AmiE (EC 3.5.1.4) |
| contig_174 | 3443 | 3892 | + | Glutamine-dependent 2-keto-4-methylthiobutyrate transaminase |
| contig_174 | 3892 | 4473 | + | Glutamine-dependent 2-keto-4-methylthiobutyrate transaminase |
| contig_174 | 4534 | 4767 | + | Ferredoxin |
| contig_1742 | 269 | 129 | - | Threonyl-tRNA synthetase (EC 6.1.1.3) |
| contig_1742 | 418 | 266 | - | Threonyl-tRNA synthetase (EC 6.1.1.3) |
| contig_1742 | 842 | 450 | - | Threonyl-tRNA synthetase (EC 6.1.1.3) |
| contig_1742 | 1496 | 1359 | - | Threonyl-tRNA synthetase (EC 6.1.1.3) |
| contig_1744 | 250 | 119 | - | Autoinducer 2 (AI-2) ABC transport system, periplasmic AI-2 binding protein LsrB |
| contig_1744 | 404 | 243 | - | Autoinducer 2 (AI-2) ABC transport system, periplasmic AI-2 binding protein LsrB |
| contig_1744 | 719 | 579 | - | Autoinducer 2 (AI-2) ABC transport system, periplasmic AI-2 binding protein LsrB |
| contig_1744 | 1029 | 688 | - | Autoinducer 2 (AI-2) ABC transport system, periplasmic AI-2 binding protein LsrB |
| contig_1744 | 1688 | 1026 | - | Autoinducer 2 (AI-2) ABC transport system, membrane channel protein LsrD |
| contig_1744 | 2018 | 1905 | - | Autoinducer 2 (AI-2) ABC transport system, membrane channel protein LsrD |
| contig_1745 | 405 | 779 | + | Polypeptide composition of the spore coat protein CotJC |
| contig_1747 | 503 | 129 | - | Catalyzes the cleavage of p-aminobenzoyl-glutamate to p-aminobenzoate and glutamate, subunit A |
| contig_1747 | 1335 | 478 | - | Catalyzes the cleavage of p-aminobenzoyl-glutamate to p-aminobenzoate and glutamate, subunit A |
| contig_1747 | 2436 | 1600 | - | Glycerate kinase (EC 2.7.1.31) |
| contig_1747 | 3011 | 2424 | - | Glycerate kinase (EC 2.7.1.31) |
| contig_1748 | 471 | 43 | - | Purine nucleoside phosphorylase (EC 2.4.2.1) |
| contig_1748 | 647 | 1072 | + | FIG01226386: hypothetical protein |
| contig_1748 | 1169 | 1435 | + | FIG01226386: hypothetical protein |
| contig_1748 | 1598 | 1440 | - | L-O-lysylphosphatidylglycerol synthase (EC 2.3.2.3) |
| contig_1748 | 1740 | 1555 | - | L-O-lysylphosphatidylglycerol synthase (EC 2.3.2.3) |
| contig_1748 | 3109 | 1697 | - | L-O-lysylphosphatidylglycerol synthase (EC 2.3.2.3) |
| contig_1748 | 3363 | 3202 | - | L-O-lysylphosphatidylglycerol synthase (EC 2.3.2.3) |
| contig_1748 | 3814 | 3482 | - | L-O-lysylphosphatidylglycerol synthase (EC 2.3.2.3) |
| contig_1748 | 4324 | 4617 | + | FIG01229729: hypothetical protein |
| contig_1748 | 4640 | 4858 | + | FIG01226807: hypothetical protein |
| contig_1748 | 4852 | 5130 | + | FIG01226807: hypothetical protein |
| contig_1749 | 835 | 443 | - | Metallo-beta-lactamase family protein |
| contig_1750 | 257 | 42 | - | FIG01234092: hypothetical protein |
| contig_1750 | 595 | 362 | - | FIG01234092: hypothetical protein |
| contig_1750 | 841 | 963 | + | hypothetical protein |
| contig_1751 | 93 | 278 | + | Transcriptional regulator, ArsR family |
| contig_1751 | 289 | 402 | + | Transcriptional regulator, ArsR family |
| contig_1751 | 1393 | 443 | - | Intracellular serine protease |
| contig_1753 | 599 | 228 | - | Protein-glutamine gamma-glutamyltransferase (EC 2.3.2.13) |
| contig_1753 | 960 | 724 | - | Protein-glutamine gamma-glutamyltransferase (EC 2.3.2.13) |
| contig_1753 | 1578 | 1036 | - | Arginine decarboxylase (EC 4.1.1.19) |
| contig_1753 | 2509 | 1622 | - | Arginine decarboxylase (EC 4.1.1.19) |
| contig_1754 | 132 | 254 | + | Chitin binding protein |
| contig_1755 | 431 | 108 | - | hypothetical protein |
| contig_1755 | 651 | 475 | - | hypothetical protein |
| contig_1755 | 866 | 732 | - | aminopeptidase |
| contig_1755 | 952 | 800 | - | aminopeptidase |
| contig_1755 | 1328 | 930 | - | aminopeptidase |
| contig_1755 | 1527 | 1336 | - | aminopeptidase |
| contig_1755 | 1943 | 1653 | - | O-Methyltransferase involved in polyketide biosynthesis |
| contig_1758 | 1091 | 441 | - | Transcriptional regulator, DeoR family |
| contig_1758 | 1382 | 1266 | - | Carboxynorspermidine dehydrogenase, putative (EC 1.1.1.-) |
| contig_1759 | 863 | 663 | - | acetyltransferase, GNAT family |
| contig_176 | 65 | 577 | + | Aspartate ammonia-lyase (EC 4.3.1.1) |
| contig_176 | 561 | 752 | + | Aspartate ammonia-lyase (EC 4.3.1.1) |
| contig_176 | 718 | 999 | + | Aspartate ammonia-lyase (EC 4.3.1.1) |
| contig_176 | 1075 | 1287 | + | NAD-dependent malic enzyme (EC 1.1.1.38) |
| contig_176 | 1373 | 2788 | + | NAD-dependent malic enzyme (EC 1.1.1.38) |
| contig_176 | 2863 | 3090 | + | Two-component sensor histidine kinase, controling glutamine utilization |
| contig_176 | 2960 | 3196 | + | Two-component sensor histidine kinase, controling glutamine utilization |
| contig_1760 | 747 | 31 | - | MFS general substrate transporter |
| contig_1762 | 37 | 606 | + | Transcriptional regulator, GntR family domain / Aspartate aminotransferase (EC 2.6.1.1) |
| contig_1762 | 676 | 1089 | + | Transcriptional regulator, GntR family domain / Aspartate aminotransferase (EC 2.6.1.1) |
| contig_1762 | 1155 | 1427 | + | Transcriptional regulator, GntR family domain / Aspartate aminotransferase (EC 2.6.1.1) |
| contig_1765 | 302 | 63 | - | Rhodanese domain protein UPF0176, Firmicutes subgroup |
| contig_1766 | 284 | 144 | - | DNA-binding response regulator |
| contig_1766 | 474 | 250 | - | DNA-binding response regulator |
| contig_1767 | 375 | 683 | + | drug resistance transporter, Bcr/CflA family |
| contig_1767 | 643 | 1095 | + | drug resistance transporter, Bcr/CflA family |
| contig_1767 | 1047 | 1214 | + | drug resistance transporter, Bcr/CflA family |
| contig_1767 | 1321 | 1452 | + | drug resistance transporter, Bcr/CflA family |
| contig_1769 | 570 | 193 | - | FIG01226156: hypothetical protein |
| contig_1769 | 728 | 573 | - | HigA protein (antitoxin to HigB) |
| contig_177 | 110 | 1576 | + | Catalase (EC 1.11.1.6) |
| contig_177 | 1693 | 1917 | + | FIG01249960: hypothetical protein |
| contig_1770 | 739 | 1092 | + | D-alanyl-D-alanine carboxypeptidase (EC 3.4.16.4) |
| contig_1773 | 601 | 125 | - | DNA topoisomerase III (EC 5.99.1.2) |
| contig_1773 | 783 | 631 | - | DNA topoisomerase III (EC 5.99.1.2) |
| contig_1773 | 1375 | 965 | - | DNA topoisomerase III (EC 5.99.1.2) |
| contig_1774 | 209 | 90 | - | hypothetical protein |
| contig_1774 | 402 | 268 | - | SinI protein, antagonist of SinR |
| contig_1775 | 15 | 965 | + | UPF0118 membrane protein YrrI |
| contig_1775 | 1346 | 1005 | - | FIG01227946: hypothetical protein |
| contig_1775 | 1830 | 3023 | + | Alanyl-tRNA synthetase (EC 6.1.1.7) |
| contig_1776 | 665 | 15 | - | Vancomycin B-type resistance protein VanW |
| contig_1778 | 105 | 389 | + | FIG01226300: hypothetical protein |
| contig_178 | 1518 | 568 | - | FIG01229902: hypothetical protein |
| contig_178 | 3659 | 1827 | - | Retron-type RNA-directed DNA polymerase (EC 2.7.7.49) |
| contig_178 | 5337 | 4375 | - | FIG01268515: hypothetical protein |
| contig_1780 | 484 | 332 | - | glycosyltransferase, putative |
| contig_1781 | 764 | 543 | - | Wall-associated protein precursor |
| contig_1783 | 477 | 322 | - | FIG01225473: hypothetical protein |
| contig_1783 | 983 | 1552 | + | drug resistance transporter, EmrB/QacA family |
| contig_1784 | 259 | 41 | - | ABC transporter, ATP-binding protein |
| contig_1784 | 457 | 299 | - | ABC transporter, ATP-binding protein |
| contig_1787 | 37 | 681 | + | Hypothetical protein, ydbT homolog |
| contig_1788 | 165 | 22 | - | Mg(2+) transport ATPase protein C |
| contig_1788 | 293 | 162 | - | Mg(2+) transport ATPase protein C |
| contig_1789 | 251 | 688 | + | Phosphoenolpyruvate-protein phosphotransferase of PTS system (EC 2.7.3.9) |
| contig_1789 | 843 | 995 | + | Phosphoenolpyruvate-protein phosphotransferase of PTS system (EC 2.7.3.9) |
| contig_1789 | 970 | 1449 | + | Phosphoenolpyruvate-protein phosphotransferase of PTS system (EC 2.7.3.9) |
| contig_1789 | 1545 | 1808 | + | Phosphoenolpyruvate-protein phosphotransferase of PTS system (EC 2.7.3.9) |
| contig_1789 | 1760 | 1972 | + | Phosphoenolpyruvate-protein phosphotransferase of PTS system (EC 2.7.3.9) |
| contig_179 | 34 | 405 | + | Two-component response regulator YvcP |
| contig_179 | 408 | 587 | + | Two-component sensor kinase YvcQ |
| contig_179 | 589 | 1302 | + | Two-component sensor kinase YvcQ |
| contig_179 | 1489 | 1322 | - | hypothetical protein |
| contig_179 | 1724 | 2344 | + | Bacitracin export permease protein BceB |
| contig_179 | 2404 | 2607 | + | Bacitracin export permease protein BceB |
| contig_179 | 2588 | 3505 | + | Bacitracin export permease protein BceB |
| contig_1790 | 101 | 502 | + | FIG01227163: hypothetical protein |
| contig_1792 | 310 | 143 | - | Uncharacterized protein, homolog of B.subtilis yhgC |
| contig_1793 | 8 | 577 | + | Spore germination protein GerXB (on pXO1) |
| contig_1794 | 670 | 350 | - | Arginyl-tRNA synthetase (EC 6.1.1.19) |
| contig_1794 | 998 | 657 | - | Arginyl-tRNA synthetase (EC 6.1.1.19) |
| contig_1795 | 762 | 325 | - | S1 RNA binding domain |
| contig_1795 | 1180 | 767 | - | S1 RNA binding domain |
| contig_1796 | 210 | 557 | + | acetyltransferase, GNAT family |
| contig_1796 | 550 | 726 | + | acetyltransferase, GNAT family |
| contig_1796 | 1061 | 1183 | + | FIG01225338: hypothetical protein |
| contig_1797 | 298 | 104 | - | FIG01229769: hypothetical protein |
| contig_1797 | 528 | 295 | - | pXO1-83 |
| contig_1797 | 692 | 579 | - | pXO1-83 |
| contig_1797 | 1417 | 842 | - | pXO1-82 |
| contig_1797 | 2689 | 1517 | - | peptidase, M23/M37 family protein |
| contig_1797 | 3071 | 2694 | - | peptidase, M23/M37 family protein |
| contig_1799 | 459 | 91 | - | D-amino-acid oxidase (EC 1.4.3.3) |
| contig_1799 | 931 | 479 | - | D-amino-acid oxidase (EC 1.4.3.3) |
| contig_1799 | 1223 | 999 | - | D-amino-acid oxidase (EC 1.4.3.3) |
| contig_18 | 28 | 192 | + | Cell division trigger factor (EC 5.2.1.8) |
| contig_18 | 218 | 976 | + | Cell division trigger factor (EC 5.2.1.8) |
| contig_18 | 1042 | 1164 | + | Cell division trigger factor (EC 5.2.1.8) |
| contig_18 | 1519 | 2175 | + | ATP-dependent Clp protease ATP-binding subunit ClpX |
| contig_18 | 2175 | 2690 | + | ATP-dependent Clp protease ATP-binding subunit ClpX |
| contig_18 | 2795 | 4291 | + | ATP-dependent protease La (EC 3.4.21.53) LonB Type I |
| contig_18 | 4329 | 4469 | + | ATP-dependent protease La (EC 3.4.21.53) LonB Type I |
| contig_18 | 4653 | 4838 | + | ATP-dependent protease La (EC 3.4.21.53) Type I |
| contig_18 | 4789 | 4908 | + | ATP-dependent protease La (EC 3.4.21.53) Type I |
| contig_18 | 4901 | 5128 | + | ATP-dependent protease La (EC 3.4.21.53) Type I |
| contig_18 | 5103 | 5360 | + | ATP-dependent protease La (EC 3.4.21.53) Type I |
| contig_18 | 5318 | 6388 | + | ATP-dependent protease La (EC 3.4.21.53) Type I |
| contig_18 | 6447 | 6989 | + | ATP-dependent protease La (EC 3.4.21.53) Type I |
| contig_18 | 6986 | 7582 | + | GTP-binding protein EngB |
| contig_18 | 7939 | 7616 | - | Organic hydroperoxide resistance protein |
| contig_18 | 8489 | 8217 | - | Organic hydroperoxide resistance transcriptional regulator |
| contig_180 | 425 | 147 | - | hypothetical protein |
| contig_180 | 604 | 437 | - | hypothetical protein |
| contig_1800 | 318 | 115 | - | FIG01227138: hypothetical protein |
| contig_1801 | 1217 | 621 | - | FIG01226496: hypothetical protein |
| contig_1801 | 1734 | 1219 | - | FIG01227949: hypothetical protein |
| contig_1802 | 21 | 182 | + | gamma-aminobutyrate (GABA) permease |
| contig_1803 | 838 | 80 | - | hypothetical protein |
| contig_1803 | 1094 | 900 | - | transcriptional regulator, Fur family |
| contig_1803 | 1398 | 1195 | - | transcriptional regulator, Fur family |
| contig_1803 | 1600 | 1355 | - | transcriptional regulator, Fur family |
| contig_1804 | 166 | 369 | + | Molybdenum cofactor biosynthesis protein MoaA |
| contig_1804 | 748 | 1176 | + | taurine ABC transporter, permease protein |
| contig_1804 | 1212 | 1592 | + | taurine ABC transporter, permease protein |
| contig_1804 | 1877 | 1656 | - | Catalase (EC 1.11.1.6) |
| contig_1804 | 2089 | 1910 | - | Catalase (EC 1.11.1.6) |
| contig_1804 | 2208 | 2077 | - | hypothetical protein |
| contig_1805 | 801 | 232 | - | Transcriptional regulator, TetR family |
| contig_1806 | 45 | 791 | + | Transcriptional regulator, IclR family |
| contig_1807 | 1064 | 384 | - | Malate:quinone oxidoreductase (EC 1.1.5.4) |
| contig_1807 | 1434 | 1042 | - | Malate:quinone oxidoreductase (EC 1.1.5.4) |
| contig_1808 | 164 | 361 | + | ABC transporter, permease |
| contig_1809 | 140 | 343 | + | FIG01227891: hypothetical protein |
| contig_1809 | 772 | 473 | - | Sensor histidine kinase |
| contig_1809 | 899 | 786 | - | Sensor histidine kinase |
| contig_181 | 1578 | 1129 | - | Dihydrolipoamide succinyltransferase component (E2) of 2-oxoglutarate dehydrogenase complex (EC 2.3.1.61) |
| contig_181 | 1723 | 1547 | - | Dihydrolipoamide succinyltransferase component (E2) of 2-oxoglutarate dehydrogenase complex (EC 2.3.1.61) |
| contig_181 | 2404 | 1733 | - | Dihydrolipoamide succinyltransferase component (E2) of 2-oxoglutarate dehydrogenase complex (EC 2.3.1.61) |
| contig_181 | 3809 | 2538 | - | 2-oxoglutarate dehydrogenase E1 component (EC 1.2.4.2) |
| contig_181 | 5406 | 3907 | - | 2-oxoglutarate dehydrogenase E1 component (EC 1.2.4.2) |
| contig_1810 | 1148 | 873 | - | FIG01227245: hypothetical protein |
| contig_1810 | 1647 | 1135 | - | FIG01227245: hypothetical protein |
| contig_1810 | 2118 | 1672 | - | Flavodoxin |
| contig_1810 | 2287 | 2111 | - | FIG01231177: hypothetical protein |
| contig_1813 | 782 | 423 | - | Glycerate kinase (EC 2.7.1.31) |
| contig_1813 | 964 | 1107 | + | Lactoylglutathione lyase and related lyases |
| contig_1815 | 841 | 695 | - | Exopolyphosphatase (EC 3.6.1.11) |
| contig_1815 | 1019 | 1261 | + | FIG01226255: hypothetical protein |
| contig_1816 | 362 | 171 | - | Tn554-related, transposase B |
| contig_1816 | 691 | 569 | - | Tn554-related, transposase A |
| contig_1816 | 1109 | 708 | - | Tn554-related, transposase A |
| contig_1817 | 204 | 82 | - | uvrD/Rep helicase family protein |
| contig_1817 | 295 | 182 | - | uvrD/Rep helicase family protein |
| contig_1817 | 676 | 392 | - | uvrD/Rep helicase family protein |
| contig_1817 | 1006 | 770 | - | uvrD/Rep helicase family protein |
| contig_1819 | 133 | 252 | + | hypothetical protein |
| contig_1819 | 249 | 818 | + | oxalate:formate antiporter, putative |
| contig_1819 | 778 | 1233 | + | oxalate:formate antiporter, putative |
| contig_1819 | 1244 | 1402 | + | oxalate:formate antiporter, putative |
| contig_182 | 216 | 404 | + | Phosphate transport system permease protein PstA (TC 3.A.1.7.1) |
| contig_182 | 430 | 723 | + | Phosphate transport system permease protein PstA (TC 3.A.1.7.1) |
| contig_182 | 728 | 841 | + | Phosphate transport system permease protein PstA (TC 3.A.1.7.1) |
| contig_182 | 843 | 1685 | + | Phosphate transport ATP-binding protein PstB (TC 3.A.1.7.1) |
| contig_182 | 1822 | 2199 | + | Phosphate transport system regulatory protein PhoU |
| contig_182 | 2189 | 2506 | + | Phosphate transport system regulatory protein PhoU |
| contig_1820 | 700 | 236 | - | DnaD domain protein |
| contig_1822 | 166 | 20 | - | RNA polymerase sigma-70 factor, ECF subfamily |
| contig_1822 | 465 | 226 | - | RNA polymerase sigma-70 factor, ECF subfamily |
| contig_1823 | 1102 | 326 | - | FIG01227842: hypothetical protein |
| contig_1825 | 195 | 380 | + | Isochorismatase (EC 3.3.2.1) |
| contig_1826 | 384 | 160 | - | Delta5 acyl-lipid desaturase (EC 1.14.99.-) |
| contig_1826 | 703 | 365 | - | Delta5 acyl-lipid desaturase (EC 1.14.99.-) |
| contig_1826 | 1068 | 946 | - | Delta5 acyl-lipid desaturase (EC 1.14.99.-) |
| contig_1827 | 66 | 299 | + | Spore germination protein GerXC (on pXO1) |
| contig_1829 | 86 | 412 | + | neutral metalloprotease, putative |
| contig_1829 | 384 | 740 | + | neutral metalloprotease, putative |
| contig_1829 | 766 | 1353 | + | neutral metalloprotease, putative |
| contig_183 | 753 | 226 | - | sensory box sigma-54 dependent DNA-binding response regulator, in GABA cluster |
| contig_183 | 1595 | 774 | - | sensory box sigma-54 dependent DNA-binding response regulator, in GABA cluster |
| contig_183 | 2795 | 1890 | - | Gamma-aminobutyrate:alpha-ketoglutarate aminotransferase (EC 2.6.1.19) |
| contig_1830 | 16 | 1215 | + | Predicted ATPase related to phosphate starvation-inducible protein PhoH |
| contig_1830 | 1897 | 2184 | + | FIG00774243: hypothetical protein YlaN |
| contig_1830 | 2385 | 2579 | + | Cell division protein FtsW |
| contig_1830 | 2546 | 2764 | + | Cell division protein FtsW |
| contig_1830 | 2767 | 3291 | + | Cell division protein FtsW |
| contig_1830 | 3257 | 3421 | + | Cell division protein FtsW |
| contig_1831 | 688 | 320 | - | FIG01226392: hypothetical protein |
| contig_1831 | 860 | 708 | - | FIG01226392: hypothetical protein |
| contig_1832 | 223 | 552 | + | TPR-repeat-containing protein |
| contig_1832 | 647 | 766 | + | TPR-repeat-containing protein |
| contig_1832 | 906 | 1022 | + | TPR-repeat-containing protein |
| contig_1832 | 1121 | 1597 | + | TPR-repeat-containing protein |
| contig_1832 | 1581 | 3347 | + | TPR-repeat-containing protein |
| contig_1833 | 719 | 255 | - | UPF0028 protein YchK |
| contig_1835 | 178 | 441 | + | YqkD |
| contig_1836 | 1017 | 742 | - | Nucleoside-diphosphate-sugar epimerases |
| contig_1837 | 42 | 155 | + | Phosphate transport regulator (distant homolog of PhoU) |
| contig_1837 | 812 | 1117 | + | Probable low-affinity inorganic phosphate transporter |
| contig_1838 | 167 | 610 | + | acetyltransferase, GNAT family |
| contig_1838 | 638 | 964 | + | FIG01225937: hypothetical protein |
| contig_1838 | 1052 | 1807 | + | S-adenosylhomocysteine deaminase (EC 3.5.4.28); Methylthioadenosine deaminase |
| contig_1838 | 1783 | 2316 | + | S-adenosylhomocysteine deaminase (EC 3.5.4.28); Methylthioadenosine deaminase |
| contig_1838 | 3056 | 2535 | - | N-formylglutamate deformylase (EC 3.5.1.68) |
| contig_1838 | 3711 | 3160 | - | N-formylglutamate deformylase (EC 3.5.1.68) |
| contig_1839 | 61 | 222 | + | hypothetical protein |
| contig_1839 | 420 | 208 | - | Signal recognition particle receptor protein FtsY (=alpha subunit) (TC 3.A.5.1.1) |
| contig_1839 | 536 | 423 | - | Signal recognition particle receptor protein FtsY (=alpha subunit) (TC 3.A.5.1.1) |
| contig_1839 | 1064 | 699 | - | Enoyl-[acyl-carrier-protein] reductase [NADH] (EC 1.3.1.9) |
| contig_184 | 553 | 425 | - | FIG01226497: hypothetical protein |
| contig_184 | 762 | 1244 | + | Transcriptional regulator, MarR family |
| contig_184 | 1322 | 2458 | + | Arsenical pump-driving ATPase (EC 3.6.3.16) |
| contig_184 | 2497 | 2688 | + | FIG01225417: hypothetical protein |
| contig_184 | 2747 | 3214 | + | FIG01228601: hypothetical protein |
| contig_184 | 4095 | 3388 | - | Methionine ABC transporter substrate-binding protein |
| contig_184 | 4931 | 4227 | - | Methionine ABC transporter permease protein |
| contig_184 | 5685 | 4921 | - | Methionine ABC transporter ATP-binding protein |
| contig_1840 | 33 | 203 | + | thiJ/pfpI family protein |
| contig_1840 | 946 | 305 | - | methyltransferase type 11 |
| contig_1841 | 3 | 215 | + | FIG01225999: hypothetical protein |
| contig_1841 | 687 | 307 | - | membrane protein, putative |
| contig_1842 | 10 | 642 | + | Arginine permease RocE |
| contig_1842 | 707 | 1228 | + | Arginine permease RocE |
| contig_1843 | 778 | 5 | - | RNA polymerase sigma factor SigB |
| contig_1843 | 902 | 744 | - | Serine-protein kinase RsbW (EC 2.7.11.1) |
| contig_1843 | 1041 | 925 | - | Serine-protein kinase RsbW (EC 2.7.11.1) |
| contig_1843 | 1153 | 1007 | - | Serine-protein kinase RsbW (EC 2.7.11.1) |
| contig_1847 | 348 | 566 | + | Respiratory nitrate reductase alpha chain (EC 1.7.99.4) |
| contig_1847 | 576 | 884 | + | Respiratory nitrate reductase alpha chain (EC 1.7.99.4) |
| contig_1847 | 934 | 1188 | + | Respiratory nitrate reductase alpha chain (EC 1.7.99.4) |
| contig_1847 | 1185 | 1562 | + | Respiratory nitrate reductase alpha chain (EC 1.7.99.4) |
| contig_1847 | 1574 | 1804 | + | Respiratory nitrate reductase alpha chain (EC 1.7.99.4) |
| contig_1848 | 392 | 75 | - | Phosphoglycolate phosphatase (EC 3.1.3.18) |
| contig_1848 | 590 | 1009 | + | membrane protein, putative |
| contig_1848 | 1012 | 1383 | + | membrane protein, putative |
| contig_1848 | 1408 | 1929 | + | FIG01228809: hypothetical protein |
| contig_1848 | 2311 | 2024 | - | hydrolase, alpha/beta fold family |
| contig_1848 | 2476 | 2342 | - | hydrolase, alpha/beta fold family |
| contig_1849 | 410 | 105 | - | acetyltransferase, GNAT family |
| contig_1849 | 579 | 454 | - | acetyltransferase, GNAT family |
| contig_185 | 902 | 780 | - | Hydroxymethylpyrimidine ABC transporter, ATPase component |
| contig_185 | 1079 | 966 | - | Hydroxymethylpyrimidine ABC transporter, ATPase component |
| contig_185 | 1548 | 1069 | - | Hydroxymethylpyrimidine ABC transporter, ATPase component |
| contig_185 | 2516 | 1560 | - | ABC transporter, substrate-binding protein, putative |
| contig_185 | 2691 | 2987 | + | FIG01226274: hypothetical protein |
| contig_185 | 3019 | 3228 | + | FIG01227268: hypothetical protein |
| contig_185 | 4715 | 3507 | - | FIG01228831: hypothetical protein |
| contig_185 | 5184 | 5372 | + | Cyclic beta-1,2-glucan modification transmembrane protein |
| contig_185 | 5497 | 5730 | + | Cyclic beta-1,2-glucan modification transmembrane protein |
| contig_185 | 5814 | 5954 | + | Cyclic beta-1,2-glucan modification transmembrane protein |
| contig_185 | 5962 | 7125 | + | Cyclic beta-1,2-glucan modification transmembrane protein |
| contig_1850 | 490 | 633 | + | FIG01226578: hypothetical protein |
| contig_1851 | 178 | 65 | - | Adenosylmethionine-8-amino-7-oxononanoate aminotransferase (EC 2.6.1.62) |
| contig_1852 | 171 | 25 | - | pXO1-52 |
| contig_1852 | 290 | 168 | - | hypothetical protein |
| contig_1854 | 119 | 6 | - | Stage II sporulation protein P |
| contig_1854 | 927 | 76 | - | Stage II sporulation protein P |
| contig_1854 | 1192 | 1314 | + | FIG01226802: hypothetical protein |
| contig_1854 | 1319 | 1468 | + | hypothetical protein |
| contig_1854 | 1465 | 1623 | + | FIG01230437: hypothetical protein |
| contig_1854 | 2123 | 1596 | - | FIG01231786: hypothetical protein |
| contig_1854 | 2444 | 2142 | - | Protein secretion chaperonin CsaA |
| contig_1855 | 661 | 152 | - | Teicoplanin resistance protein vanZ |
| contig_1855 | 1202 | 900 | - | Transcriptional regulator, PadR family |
| contig_1856 | 591 | 49 | - | FIG01227673: hypothetical protein |
| contig_1857 | 386 | 198 | - | hypothetical protein |
| contig_1858 | 561 | 10 | - | permease, putative |
| contig_1859 | 597 | 79 | - | Tn7-like transposition protein B |
| contig_1859 | 1022 | 627 | - | Tn7-like transposition protein B |
| contig_1859 | 1506 | 1171 | - | Tn7-like transposition protein B |
| contig_186 | 839 | 81 | - | Transcriptional regulator, TrmB family |
| contig_1860 | 503 | 156 | - | S-layer protein / N-acetylmuramoyl-L-alanine amidase (EC 3.5.1.28) |
| contig_1861 | 457 | 20 | - | Hypothetical radical SAM family enzyme in heat shock gene cluster, similarity with CPO of BS HemN-type |
| contig_1861 | 758 | 510 | - | Transcriptional regulator, HxlR family |
| contig_1863 | 697 | 203 | - | 1-deoxy-D-xylulose 5-phosphate synthase (EC 2.2.1.7) |
| contig_1863 | 1070 | 759 | - | 1-deoxy-D-xylulose 5-phosphate synthase (EC 2.2.1.7) |
| contig_1864 | 222 | 536 | + | conserved hypothetical integral membrane protein, putative |
| contig_1864 | 626 | 805 | + | conserved hypothetical integral membrane protein, putative |
| contig_1865 | 1026 | 190 | - | Beta-lactamase (EC 3.5.2.6) |
| contig_1866 | 198 | 323 | + | hypothetical protein |
| contig_1866 | 307 | 510 | + | Phosphonate ABC transporter phosphate-binding periplasmic component (TC 3.A.1.9.1) |
| contig_1867 | 204 | 13 | - | SSU ribosomal protein S1p |
| contig_1868 | 593 | 240 | - | FIG01226521: hypothetical protein |
| contig_1869 | 154 | 2 | - | collagen adhesion protein |
| contig_1869 | 302 | 156 | - | collagen adhesion protein |
| contig_1869 | 825 | 256 | - | Cell wall surface anchor family protein, LPXTG motif |
| contig_1869 | 1170 | 958 | - | Cell wall surface anchor family protein, LPXTG motif |
| contig_187 | 221 | 337 | + | Na+/H+ antiporter |
| contig_187 | 351 | 1535 | + | Na+/H+ antiporter |
| contig_187 | 1625 | 1846 | + | FIG01226288: hypothetical protein |
| contig_187 | 1861 | 2298 | + | acetyltransferase, GNAT family |
| contig_187 | 2563 | 2384 | - | FIG01226364: hypothetical protein |
| contig_187 | 2801 | 2550 | - | FIG01226364: hypothetical protein |
| contig_187 | 4178 | 3108 | - | Guanine-hypoxanthine permease |
| contig_187 | 4401 | 4204 | - | Guanine-hypoxanthine permease |
| contig_187 | 4682 | 5404 | + | D-serine/D-alanine/glycine transporter |
| contig_187 | 5451 | 6047 | + | D-serine/D-alanine/glycine transporter |
| contig_1870 | 207 | 386 | + | acetyltransferase, GNAT family |
| contig_1870 | 447 | 710 | + | acetyltransferase, GNAT family |
| contig_1872 | 974 | 543 | - | Tetratricopeptide repeat family protein |
| contig_1873 | 726 | 340 | - | Tetratricopeptide repeat family protein |
| contig_1874 | 711 | 118 | - | Pullulanase (EC 3.2.1.41) |
| contig_1875 | 261 | 97 | - | collagen adhesion protein |
| contig_1875 | 418 | 230 | - | collagen adhesion protein |
| contig_1875 | 1218 | 436 | - | collagen adhesion protein |
| contig_1877 | 347 | 502 | + | Ribosomal-protein-alanine acetyltransferase (EC 2.3.1.128) |
| contig_1878 | 875 | 381 | - | Adenosylmethionine-8-amino-7-oxononanoate aminotransferase (EC 2.6.1.62) |
| contig_1878 | 1231 | 971 | - | Putative symporter YjcG |
| contig_188 | 499 | 110 | - | Ferric iron ABC transporter, permease protein |
| contig_188 | 687 | 538 | - | Ferric iron ABC transporter, permease protein |
| contig_188 | 1072 | 773 | - | Ferric iron ABC transporter, permease protein |
| contig_188 | 1583 | 1092 | - | Ferric iron ABC transporter, permease protein |
| contig_188 | 3045 | 1714 | - | Ferric iron ABC transporter, permease protein |
| contig_188 | 3761 | 3159 | - | Ferric iron ABC transporter, iron-binding protein |
| contig_188 | 3877 | 3746 | - | Ferric iron ABC transporter, iron-binding protein |
| contig_188 | 4088 | 3855 | - | Ferric iron ABC transporter, iron-binding protein |
| contig_188 | 4680 | 4066 | - | Ferric iron ABC transporter, ATP-binding protein |
| contig_1880 | 225 | 88 | - | sensor histidine kinase |
| contig_1880 | 1048 | 245 | - | sensor histidine kinase |
| contig_1880 | 1448 | 1215 | - | response regulator DrrA |
| contig_1880 | 1543 | 1331 | - | response regulator DrrA |
| contig_1880 | 1923 | 1624 | - | response regulator DrrA |
| contig_1881 | 130 | 17 | - | hypothetical protein |
| contig_1881 | 394 | 224 | - | ABC transporter, ATP-binding protein, putative |
| contig_1881 | 624 | 391 | - | ABC transporter, ATP-binding protein, putative |
| contig_1882 | 406 | 173 | - | hydrolase, alpha/beta fold family |
| contig_1884 | 567 | 217 | - | tRNA pseudouridine synthase A (EC 4.2.1.70) |
| contig_1885 | 19 | 294 | + | Transcriptional regulator, PadR family |
| contig_1885 | 647 | 844 | + | Deoxyribodipyrimidine photolyase (EC 4.1.99.3) |
| contig_1885 | 810 | 1055 | + | Deoxyribodipyrimidine photolyase (EC 4.1.99.3) |
| contig_1885 | 1121 | 1261 | + | Deoxyribodipyrimidine photolyase (EC 4.1.99.3) |
| contig_1886 | 233 | 108 | - | FIG01229155: hypothetical protein |
| contig_1887 | 351 | 61 | - | ATP phosphoribosyltransferase (EC 2.4.2.17) |
| contig_1887 | 577 | 326 | - | ATP phosphoribosyltransferase (EC 2.4.2.17) |
| contig_1887 | 1140 | 553 | - | ATP phosphoribosyltransferase regulatory subunit (EC 2.4.2.17) |
| contig_1887 | 1814 | 1182 | - | ATP phosphoribosyltransferase regulatory subunit (EC 2.4.2.17) |
| contig_1888 | 207 | 28 | - | FIG01225853: hypothetical protein |
| contig_1889 | 480 | 307 | - | pXO1-135 |
| contig_189 | 119 | 379 | + | FIG011501: YycH protein |
| contig_189 | 360 | 560 | + | hypothetical protein SA_21 |
| contig_189 | 632 | 979 | + | hypothetical protein SA_21 |
| contig_189 | 1189 | 1983 | + | Zn-dependent hydrolase YycJ/WalJ, required for cell wall metabolism and coordination of cell division with DNA replication |
| contig_189 | 2047 | 3144 | + | Serine protease, DegP/HtrA, do-like (EC 3.4.21.-) |
| contig_189 | 3277 | 3462 | + | FIG01225978: hypothetical protein |
| contig_189 | 3522 | 3728 | + | LSU m3Psi1915 methyltransferase RlmH |
| contig_189 | 4139 | 5017 | + | Molybdenum cofactor biosynthesis enzyme and related Fe-S oxidoreductases |
| contig_189 | 6872 | 5055 | - | Oligoendopeptidase F (EC 3.4.24.-) |
| contig_189 | 7312 | 7010 | - | GMP reductase (EC 1.7.1.7) |
| contig_1890 | 1122 | 589 | - | Probable GTPase related to EngC |
| contig_1890 | 1469 | 1314 | - | FIG01228117: hypothetical protein |
| contig_1890 | 1830 | 1603 | - | FIG01228117: hypothetical protein |
| contig_1890 | 2024 | 2224 | + | FIG01226337: hypothetical protein |
| contig_1892 | 623 | 435 | - | FIG01231443: hypothetical protein |
| contig_1893 | 340 | 164 | - | Endonuclease III (EC 4.2.99.18) |
| contig_1894 | 383 | 147 | - | RecU Holliday junction resolvase |
| contig_1894 | 532 | 380 | - | RecU Holliday junction resolvase |
| contig_1895 | 437 | 90 | - | Beta-lactamase (EC 3.5.2.6) |
| contig_1895 | 638 | 501 | - | Beta-lactamase (EC 3.5.2.6) |
| contig_1895 | 804 | 619 | - | Beta-lactamase (EC 3.5.2.6) |
| contig_1898 | 277 | 143 | - | hypothetical protein |
| contig_1898 | 553 | 338 | - | response regulator, putative |
| contig_1898 | 1199 | 672 | - | response regulator, putative |
| contig_1899 | 437 | 285 | - | hypothetical protein |
| contig_19 | 3305 | 18 | - | Carbamoyl-phosphate synthase large chain (EC 6.3.5.5) |
| contig_19 | 4108 | 3302 | - | Dihydroorotase (EC 3.5.2.3) |
| contig_19 | 4280 | 4140 | - | Dihydroorotase (EC 3.5.2.3) |
| contig_19 | 4590 | 4255 | - | Dihydroorotase (EC 3.5.2.3) |
| contig_19 | 5494 | 4574 | - | Aspartate carbamoyltransferase (EC 2.1.3.2) |
| contig_19 | 6931 | 5615 | - | Uracil permease |
| contig_19 | 7256 | 7077 | - | Uracil phosphoribosyltransferase (EC 2.4.2.9) / Pyrimidine operon regulatory protein PyrR |
| contig_19 | 7623 | 7435 | - | Uracil phosphoribosyltransferase (EC 2.4.2.9) / Pyrimidine operon regulatory protein PyrR |
| contig_19 | 8545 | 7826 | - | Ribosomal large subunit pseudouridine synthase D (EC 4.2.1.70) |
| contig_190 | 470 | 180 | - | Inhibitor of pro-sigmaK processing BofA |
| contig_190 | 885 | 568 | - | FIG015094: hypothetical protein |
| contig_190 | 1496 | 900 | - | Recombination protein RecR |
| contig_190 | 1840 | 1511 | - | FIG000557: hypothetical protein co-occurring with RecR |
| contig_190 | 3469 | 1895 | - | DNA polymerase III subunits gamma and tau (EC 2.7.7.7) |
| contig_190 | 4351 | 4031 | - | tRNA-specific adenosine-34 deaminase (EC 3.5.4.-) |
| contig_190 | 4518 | 5180 | + | Nicotinamidase (EC 3.5.1.19) |
| contig_190 | 5306 | 5698 | + | Deoxyadenosine kinase (EC 2.7.1.76) / Deoxyguanosine kinase (EC 2.7.1.113) |
| contig_190 | 5727 | 5942 | + | Deoxyadenosine kinase (EC 2.7.1.76) / Deoxyguanosine kinase (EC 2.7.1.113) |
| contig_190 | 5945 | 6613 | + | Deoxyadenosine kinase (EC 2.7.1.76) / Deoxyguanosine kinase (EC 2.7.1.113) |
| contig_190 | 6762 | 6649 | - | FIG01226642: hypothetical protein |
| contig_190 | 7042 | 6794 | - | FIG01226642: hypothetical protein |
| contig_190 | 7300 | 7208 | - | tRNA-Ser-TGA |
| contig_190 | 8728 | 7457 | - | Seryl-tRNA synthetase (EC 6.1.1.11) |
| contig_190 | 9651 | 9061 | - | Pyridoxine biosynthesis glutamine amidotransferase, glutaminase subunit (EC 2.4.2.-) |
| contig_190 | 10122 | 9670 | - | Pyridoxine biosynthesis glutamine amidotransferase, synthase subunit (EC 2.4.2.-) |
| contig_190 | 10429 | 10196 | - | Pyridoxine biosynthesis glutamine amidotransferase, synthase subunit (EC 2.4.2.-) |
| contig_190 | 10849 | 10724 | - | D-alanyl-D-alanine carboxypeptidase (EC 3.4.16.4) |
| contig_190 | 11727 | 10837 | - | D-alanyl-D-alanine carboxypeptidase (EC 3.4.16.4) |
| contig_190 | 13141 | 12146 | - | Inosine-5'-monophosphate dehydrogenase (EC 1.1.1.205) / CBS domain |
| contig_190 | 13610 | 13092 | - | Inosine-5'-monophosphate dehydrogenase (EC 1.1.1.205) / CBS domain |
| contig_190 | 13726 | 13962 | + | Acetoacetyl-CoA reductase (EC 1.1.1.36) |
| contig_190 | 14045 | 14764 | + | Polyhydroxyalkanoic acid synthase |
| contig_190 | 14874 | 15131 | + | Polyhydroxyalkanoic acid synthase |
| contig_190 | 15226 | 15459 | + | FIG01225455: hypothetical protein |
| contig_1900 | 185 | 72 | - | hydrolase, haloacid dehalogenase-like family |
| contig_1900 | 300 | 157 | - | hydrolase, haloacid dehalogenase-like family |
| contig_1900 | 533 | 354 | - | Mn-dependent transcriptional regulator MntR |
| contig_1900 | 786 | 628 | - | Mn-dependent transcriptional regulator MntR |
| contig_1900 | 1245 | 931 | - | Spore photoproduct lyase (EC 4.1.99.-) |
| contig_1900 | 1957 | 1340 | - | Spore photoproduct lyase (EC 4.1.99.-) |
| contig_1901 | 500 | 60 | - | Cysteine desulfurase (EC 2.8.1.7) |
| contig_1901 | 1169 | 645 | - | Cysteine desulfurase (EC 2.8.1.7) |
| contig_1902 | 347 | 111 | - | FIG01228597: hypothetical protein |
| contig_1902 | 604 | 843 | + | Thymidylate_kin, Thymidylate kinase |
| contig_1902 | 887 | 1228 | + | Thymidylate_kin, Thymidylate kinase |
| contig_1903 | 344 | 583 | + | N-acetylmannosaminyltransferase (EC 2.4.1.187) |
| contig_1904 | 156 | 344 | + | Formamidopyrimidine-DNA glycosylase (EC 3.2.2.23) |
| contig_1904 | 417 | 686 | + | membrane protein, putative |
| contig_1905 | 65 | 187 | + | SSU ribosomal protein S20p |
| contig_1905 | 544 | 272 | - | DNA polymerase III delta subunit (EC 2.7.7.7) |
| contig_1906 | 289 | 173 | - | Selenoprotein O and cysteine-containing homologs |
| contig_1907 | 646 | 80 | - | N-acetylglucosamine-6-phosphate deacetylase (EC 3.5.1.25) |
| contig_1909 | 324 | 55 | - | Hypothetical hydrolase |
| contig_1909 | 415 | 293 | - | Hypothetical hydrolase |
| contig_1909 | 791 | 564 | - | Hypothetical hydrolase |
| contig_191 | 527 | 661 | + | Phage shock protein A |
| contig_191 | 675 | 857 | + | Phage shock protein A |
| contig_191 | 913 | 1113 | + | Phage shock protein A |
| contig_191 | 1194 | 1598 | + | surface protein |
| contig_191 | 1620 | 2126 | + | FIG01226022: hypothetical protein |
| contig_191 | 2139 | 2432 | + | Phosphonate ABC transporter phosphate-binding periplasmic component (TC 3.A.1.9.1) |
| contig_191 | 2413 | 2601 | + | Phosphonate ABC transporter phosphate-binding periplasmic component (TC 3.A.1.9.1) |
| contig_191 | 2564 | 2962 | + | Phosphonate ABC transporter phosphate-binding periplasmic component (TC 3.A.1.9.1) |
| contig_191 | 3053 | 3271 | + | FIG01225617: hypothetical protein |
| contig_191 | 4239 | 3376 | - | Uncharacterized membrane protein Bsu0528 (YdeO) |
| contig_191 | 5131 | 4427 | - | Glycerate kinase (EC 2.7.1.31) |
| contig_191 | 5466 | 5164 | - | Glycerate kinase (EC 2.7.1.31) |
| contig_1910 | 289 | 119 | - | hypothetical protein |
| contig_1911 | 222 | 7 | - | Protein ydhR precursor |
| contig_1911 | 514 | 314 | - | Probable bifunctional P-450/NADPH-P450 reductase CypD |
| contig_1912 | 261 | 37 | - | Non-hemolytic enterotoxin A |
| contig_1914 | 245 | 367 | + | FIG01226548: hypothetical protein |
| contig_1915 | 1086 | 805 | - | Phosphonate ABC transporter phosphate-binding periplasmic component (TC 3.A.1.9.1) |
| contig_1916 | 147 | 272 | + | FIG01226147: hypothetical protein |
| contig_1916 | 371 | 706 | + | FIG01226147: hypothetical protein |
| contig_1917 | 254 | 117 | - | hypothetical protein |
| contig_1919 | 178 | 306 | + | Stage IV sporulation protein FA (SpoIVFA) |
| contig_1919 | 299 | 811 | + | Stage IV sporulation pro-sigma-K processing enzyme (SpoIVFB) |
| contig_1919 | 846 | 965 | + | hypothetical protein |
| contig_1919 | 919 | 1161 | + | Stage IV sporulation pro-sigma-K processing enzyme (SpoIVFB) |
| contig_192 | 444 | 217 | - | membrane protein, putative |
| contig_192 | 774 | 517 | - | membrane protein, putative |
| contig_192 | 1367 | 882 | - | membrane protein, putative |
| contig_192 | 1673 | 1407 | - | PXO1-87 |
| contig_192 | 2451 | 2564 | + | hypothetical protein |
| contig_1920 | 370 | 948 | + | Stage IV sporulation protein A |
| contig_1922 | 502 | 209 | - | hypothetical protein |
| contig_1923 | 687 | 286 | - | Ribosomal-protein-alanine acetyltransferase (EC 2.3.1.128) |
| contig_1923 | 962 | 807 | - | Oligopeptide ABC transporter, periplasmic oligopeptide-binding protein OppA (TC 3.A.1.5.1) |
| contig_1924 | 312 | 193 | - | UDP-N-acetylmuramoylalanyl-D-glutamyl-2,6-diaminopimelate--D-alanyl-D-alanine ligase (EC 6.3.2.10) |
| contig_1926 | 453 | 220 | - | FIG01227990: hypothetical protein |
| contig_1926 | 852 | 496 | - | Flagellar biosynthesis protein FliS |
| contig_1926 | 1621 | 977 | - | Flagellar hook-associated protein FliD |
| contig_1926 | 1977 | 1735 | - | Flagellar hook-associated protein FliD |
| contig_1927 | 151 | 444 | + | Transcriptional regulator, ArsR family |
| contig_1927 | 938 | 753 | - | RNA-binding protein Hfq |
| contig_1927 | 1576 | 1202 | - | hypothetical protein |
| contig_1928 | 28 | 264 | + | FIG012639: hypothetical Membrane Spanning Protein |
| contig_193 | 143 | 433 | + | Penicillin acylase II (EC 3.5.1.11) |
| contig_193 | 427 | 765 | + | Penicillin acylase II (EC 3.5.1.11) |
| contig_193 | 2370 | 985 | - | Two-component protein Kinase |
| contig_193 | 3431 | 2889 | - | FIG01237481: hypothetical protein |
| contig_1930 | 506 | 249 | - | membrane protein, putative |
| contig_1933 | 421 | 296 | - | Aminoglycoside N3'-acetyltransferase (EC 2.3.1.81) |
| contig_1933 | 717 | 403 | - | Aminoglycoside N3'-acetyltransferase (EC 2.3.1.81) |
| contig_1933 | 958 | 758 | - | Aminoglycoside N3'-acetyltransferase (EC 2.3.1.81) |
| contig_1934 | 251 | 138 | - | hypothetical protein |
| contig_1934 | 601 | 266 | - | Putative stomatin/prohibitin-family membrane protease subunit YbbK |
| contig_1934 | 836 | 660 | - | Putative stomatin/prohibitin-family membrane protease subunit YbbK |
| contig_1934 | 1111 | 833 | - | Putative stomatin/prohibitin-family membrane protease subunit YbbK |
| contig_1934 | 1418 | 1245 | - | Putative activity regulator of membrane protease YbbK |
| contig_1934 | 1672 | 1433 | - | Putative activity regulator of membrane protease YbbK |
| contig_1934 | 1946 | 2353 | + | membrane protein, putative |
| contig_1934 | 2507 | 2394 | - | hypothetical protein |
| contig_1934 | 2557 | 2916 | + | membrane protein, putative |
| contig_1934 | 2894 | 3121 | + | membrane protein, putative |
| contig_1935 | 72 | 545 | + | Shikimate kinase I (EC 2.7.1.71) |
| contig_1935 | 611 | 838 | + | FIG01225342: hypothetical protein |
| contig_1937 | 148 | 282 | + | hypothetical protein |
| contig_1938 | 253 | 95 | - | Excinuclease ABC subunit A domain protein |
| contig_1938 | 713 | 258 | - | Excinuclease ABC subunit A domain protein |
| contig_1938 | 1002 | 808 | - | Excinuclease ABC subunit A domain protein |
| contig_1938 | 1078 | 959 | - | Excinuclease ABC subunit A domain protein |
| contig_1939 | 219 | 91 | - | Transcriptional regulator, MerR family |
| contig_194 | 205 | 59 | - | Transcriptional regulator, MarR family |
| contig_194 | 1138 | 791 | - | Threonine dehydratase, catabolic (EC 4.3.1.19) |
| contig_194 | 1472 | 1122 | - | Threonine dehydratase, catabolic (EC 4.3.1.19) |
| contig_194 | 1784 | 1551 | - | Threonine dehydratase, catabolic (EC 4.3.1.19) |
| contig_194 | 2149 | 1871 | - | Protein export cytoplasm protein SecA ATPase RNA helicase (TC 3.A.5.1.1) |
| contig_194 | 2456 | 2154 | - | Protein export cytoplasm protein SecA ATPase RNA helicase (TC 3.A.5.1.1) |
| contig_1940 | 413 | 9 | - | D-alanyl-D-alanine carboxypeptidase (EC 3.4.16.4) |
| contig_1940 | 758 | 633 | - | D-alanyl-D-alanine carboxypeptidase (EC 3.4.16.4) |
| contig_1944 | 168 | 1259 | + | tRNA-t(6)A37 methylthiotransferase |
| contig_1945 | 431 | 264 | - | FIG01230473: hypothetical protein |
| contig_1945 | 591 | 412 | - | FIG01230473: hypothetical protein |
| contig_1946 | 1700 | 213 | - | N-acetylmuramoyl-L-alanine amidase |
| contig_1946 | 2123 | 1914 | - | FIG01225778: hypothetical protein |
| contig_1946 | 2321 | 2178 | - | FIG01225778: hypothetical protein |
| contig_1947 | 1036 | 506 | - | Long-chain-fatty-acid--CoA ligase (EC 6.2.1.3) |
| contig_1947 | 1720 | 1103 | - | Long-chain-fatty-acid--CoA ligase (EC 6.2.1.3) |
| contig_1948 | 535 | 95 | - | Nitrate/nitrite sensor protein (EC 2.7.3.-) |
| contig_195 | 220 | 771 | + | Aldehyde dehydrogenase (EC 1.2.1.3) in 4-hydroxyproline catabolic gene cluster |
| contig_195 | 901 | 1149 | + | Aldehyde dehydrogenase (EC 1.2.1.3) in 4-hydroxyproline catabolic gene cluster |
| contig_195 | 1325 | 1849 | + | sodium/alanine symporter family protein |
| contig_195 | 1869 | 2546 | + | sodium/alanine symporter family protein |
| contig_1951 | 443 | 688 | + | hypothetical protein |
| contig_1952 | 488 | 123 | - | PBS lyase HEAT-like repeat domain protein |
| contig_1952 | 678 | 466 | - | PBS lyase HEAT-like repeat domain protein |
| contig_1953 | 354 | 202 | - | FIG01236894: hypothetical protein |
| contig_1953 | 928 | 629 | - | Transcriptional regulatory protein ComA (Bsu) |
| contig_1954 | 264 | 611 | + | 2-dehydropantoate 2-reductase (EC 1.1.1.169) |
| contig_1954 | 574 | 777 | + | 2-dehydropantoate 2-reductase (EC 1.1.1.169) |
| contig_1954 | 798 | 1154 | + | 2-dehydropantoate 2-reductase (EC 1.1.1.169) |
| contig_1955 | 221 | 72 | - | FIG002540: Haloacid dehalogenase-like hydrolase |
| contig_1955 | 706 | 467 | - | Cysteine dioxygenase |
| contig_1956 | 103 | 480 | + | Mobile element protein |
| contig_1956 | 840 | 601 | - | hypothetical protein |
| contig_1958 | 224 | 111 | - | Putative transcriptional antiterminator, BglG family / PTS system, mannitol/fructose-specific IIA component (EC 2.7.1.69) |
| contig_1958 | 297 | 184 | - | Putative transcriptional antiterminator, BglG family / PTS system, mannitol/fructose-specific IIA component (EC 2.7.1.69) |
| contig_1959 | 884 | 255 | - | CAAX amino terminal protease family protein |
| contig_1959 | 1317 | 1475 | + | hypothetical protein |
| contig_196 | 74 | 208 | + | hypothetical protein |
| contig_196 | 294 | 422 | + | hypothetical protein |
| contig_196 | 2122 | 953 | - | putative |
| contig_196 | 2595 | 2182 | - | hypothetical protein |
| contig_196 | 2753 | 2607 | - | Hypothetical SAV0808 homolog, near pathogenicity islands SaPI att-site |
| contig_196 | 3262 | 2840 | - | Phage protein |
| contig_196 | 4113 | 4226 | + | hypothetical protein |
| contig_1960 | 998 | 723 | - | General stress protein 17M |
| contig_1964 | 485 | 132 | - | Wall-associated protein precursor |
| contig_1967 | 274 | 492 | + | Methyltransferase (EC 2.1.1.-) |
| contig_1967 | 552 | 719 | + | Methyltransferase (EC 2.1.1.-) |
| contig_1968 | 133 | 20 | - | peptidase M3 family protein |
| contig_1969 | 1039 | 155 | - | Phosphatidylinositol-specific phospholipase C (EC 4.6.1.13) |
| contig_1969 | 1145 | 996 | - | Phosphatidylinositol-specific phospholipase C (EC 4.6.1.13) |
| contig_197 | 1158 | 664 | - | Type III restriction-modification system StyLTI enzyme res (EC 3.1.21.5) |
| contig_197 | 1495 | 1112 | - | Type III restriction-modification system StyLTI enzyme res (EC 3.1.21.5) |
| contig_197 | 1766 | 1500 | - | Type III restriction-modification system StyLTI enzyme res (EC 3.1.21.5) |
| contig_197 | 2255 | 1812 | - | Type III restriction-modification system StyLTI enzyme res (EC 3.1.21.5) |
| contig_197 | 3535 | 2354 | - | Type III restriction-modification system StyLTI enzyme res (EC 3.1.21.5) |
| contig_197 | 5000 | 3654 | - | Type III restriction-modification system methylation subunit (EC 2.1.1.72) |
| contig_197 | 5185 | 5066 | - | Type III restriction-modification system methylation subunit (EC 2.1.1.72) |
| contig_197 | 5369 | 5211 | - | Type III restriction-modification system methylation subunit (EC 2.1.1.72) |
| contig_197 | 5683 | 5567 | - | Type III restriction-modification system methylation subunit (EC 2.1.1.72) |
| contig_197 | 6498 | 6376 | - | hypothetical protein |
| contig_197 | 8291 | 6525 | - | 5-methylcytosine-specific restriction related enzyme |
| contig_197 | 9105 | 8281 | - | 5-methylcytosine-specific restriction related enzyme |
| contig_197 | 9410 | 9120 | - | 5-methylcytosine-specific restriction related enzyme |
| contig_197 | 9621 | 9391 | - | 5-methylcytosine-specific restriction related enzyme |
| contig_1970 | 6 | 332 | + | Rhodanese domain protein UPF0176, Firmicutes subgroup |
| contig_1971 | 130 | 264 | + | FIG01226568: hypothetical protein |
| contig_1972 | 313 | 170 | - | Oxidoreductase, FAD/FMN-binding |
| contig_1972 | 623 | 291 | - | Oxidoreductase, FAD/FMN-binding |
| contig_1972 | 1320 | 700 | - | Oxidoreductase, FAD/FMN-binding |
| contig_1973 | 364 | 179 | - | Alkanesulfonates-binding protein |
| contig_1974 | 266 | 105 | - | FIG01226707: hypothetical protein |
| contig_1974 | 702 | 286 | - | FIG01226707: hypothetical protein |
| contig_1975 | 199 | 729 | + | Microbial collagenase (EC 3.4.24.3) |
| contig_1977 | 12 | 194 | + | Protein of unknown function DUF1447 |
| contig_198 | 482 | 655 | + | hypothetical protein |
| contig_198 | 1020 | 901 | - | hypothetical protein |
| contig_198 | 1138 | 1025 | - | hypothetical protein |
| contig_198 | 1382 | 1140 | - | hypothetical protein |
| contig_198 | 2070 | 1519 | - | hypothetical protein |
| contig_198 | 2501 | 2070 | - | hypothetical protein |
| contig_198 | 3706 | 3509 | - | hypothetical protein |
| contig_198 | 4394 | 3675 | - | hypothetical protein |
| contig_198 | 5032 | 4541 | - | Tn7-like transposition protein D |
| contig_198 | 5327 | 5061 | - | Tn7-like transposition protein D |
| contig_198 | 6289 | 5321 | - | Tn7-like transposition protein D |
| contig_198 | 7186 | 6293 | - | Tn7-like transposition protein C |
| contig_198 | 7925 | 7158 | - | Tn7-like transposition protein C |
| contig_198 | 8138 | 7959 | - | Transposon Tn7 transposition protein tnsB |
| contig_198 | 8691 | 8335 | - | Transposon Tn7 transposition protein tnsB |
| contig_198 | 9235 | 8819 | - | Transposon Tn7 transposition protein tnsB |
| contig_1980 | 562 | 293 | - | Tellurium resistance protein TerD |
| contig_1981 | 575 | 264 | - | pXO1-08 |
| contig_1982 | 309 | 163 | - | FIG01226332: hypothetical protein |
| contig_1984 | 389 | 216 | - | BH2577 unknown conserved protein in B. subtilis |
| contig_1986 | 560 | 297 | - | FIG01225287: hypothetical protein |
| contig_1987 | 301 | 720 | + | CBS domain containing protein |
| contig_1988 | 60 | 638 | + | acetyltransferase, GNAT family |
| contig_1988 | 638 | 754 | + | Uridine kinase (EC 2.7.1.48) |
| contig_1988 | 764 | 928 | + | Uridine kinase (EC 2.7.1.48) |
| contig_1989 | 501 | 61 | - | Galactosamine-6-phosphate isomerase (EC 5.3.1.-) |
| contig_1989 | 661 | 491 | - | Galactosamine-6-phosphate isomerase (EC 5.3.1.-) |
| contig_199 | 643 | 239 | - | Transcriptional regulator, TetR family |
| contig_199 | 1156 | 746 | - | Transcriptional regulator, LacI family |
| contig_199 | 1596 | 1180 | - | Transcriptional regulator, LacI family |
| contig_199 | 2367 | 1993 | - | Rhodanese-like domain protein |
| contig_199 | 2540 | 3184 | + | Lipoate-protein ligase A |
| contig_199 | 4201 | 3407 | - | FIG01228778: hypothetical protein |
| contig_199 | 4518 | 4369 | - | hypothetical protein |
| contig_1990 | 51 | 770 | + | Autoinducer 2 (AI-2) ABC transport system, membrane channel protein LsrC |
| contig_1990 | 1007 | 1192 | + | Autoinducer 2 (AI-2) ABC transport system, membrane channel protein LsrD |
| contig_1991 | 627 | 466 | - | FIG01225177: hypothetical protein |
| contig_1992 | 632 | 15 | - | N,N'-diacetylchitobiose-specific regulator ChbR, AraC family |
| contig_1993 | 521 | 739 | + | FIG01241249: hypothetical protein |
| contig_1993 | 1390 | 1103 | - | Foldase protein PrsA precursor (EC 5.2.1.8) |
| contig_1993 | 1648 | 1526 | - | hypothetical protein |
| contig_1993 | 1859 | 1635 | - | Foldase protein PrsA precursor (EC 5.2.1.8) |
| contig_1994 | 129 | 395 | + | FIG01235027: hypothetical protein |
| contig_1994 | 419 | 643 | + | FIG01235736: hypothetical protein |
| contig_1994 | 904 | 1188 | + | pXO1-28 |
| contig_1994 | 1205 | 1369 | + | FIG01234056: hypothetical protein |
| contig_1994 | 1378 | 1512 | + | FIG01234056: hypothetical protein |
| contig_1995 | 247 | 53 | - | hypothetical protein |
| contig_1995 | 359 | 222 | - | hypothetical protein |
| contig_1995 | 739 | 918 | + | pXO1-28 |
| contig_1996 | 413 | 249 | - | Bacillolysin (EC 3.4.24.28) |
| contig_1996 | 643 | 416 | - | Bacillolysin (EC 3.4.24.28) |
| contig_1996 | 833 | 612 | - | Bacillolysin (EC 3.4.24.28) |
| contig_1997 | 387 | 142 | - | FIG01227637: hypothetical protein |
| contig_1997 | 567 | 427 | - | wall-associated protein |
| contig_1997 | 943 | 629 | - | wall-associated protein |
| contig_1998 | 437 | 231 | - | Monofunctional biosynthetic peptidoglycan transglycosylase (EC 2.4.2.-) |
| contig_2 | 510 | 55 | - | AmiS_UreI, AmiS/UreI family transporter |
| contig_2 | 1746 | 946 | - | FIG01238016: hypothetical protein |
| contig_2 | 2056 | 2673 | + | UDP-glucose 4-epimerase (EC 5.1.3.2) |
| contig_2 | 2925 | 3281 | + | UDP-glucose 4-epimerase (EC 5.1.3.2) |
| contig_2 | 3281 | 3769 | + | FIG01233530: hypothetical protein |
| contig_2 | 3732 | 4202 | + | FIG01233530: hypothetical protein |
| contig_2 | 4367 | 4672 | + | FIG01225173: hypothetical protein |
| contig_2 | 4669 | 5037 | + | FIG01225173: hypothetical protein |
| contig_20 | 116 | 412 | + | FIG01108140: hypothetical protein |
| contig_20 | 502 | 654 | + | hypothetical protein |
| contig_20 | 1318 | 929 | - | ComK regulator |
| contig_20 | 1766 | 1521 | - | Predicted broad substrate specificity phosphatase |
| contig_20 | 2010 | 1747 | - | Predicted broad substrate specificity phosphatase |
| contig_20 | 2338 | 2093 | - | BH2600 unknown conserved protein in B. subtilis |
| contig_20 | 2463 | 2350 | - | BH2601 unknown conserved protein in B. subtilis |
| contig_200 | 647 | 24 | - | membrane protein, putative |
| contig_2000 | 190 | 14 | - | nucleotidyltransferase domain protein |
| contig_2002 | 241 | 14 | - | Transcriptional regulator, Cro/CI family |
| contig_2002 | 447 | 256 | - | Transcriptional regulator, Cro/CI family |
| contig_2005 | 179 | 394 | + | Tyrosine recombinase XerC |
| contig_2005 | 697 | 584 | - | hypothetical protein |
| contig_2006 | 73 | 228 | + | FIG004556: membrane metalloprotease |
| contig_2007 | 35 | 226 | + | FIG004556: membrane metalloprotease |
| contig_2008 | 67 | 228 | + | hypothetical protein |
| contig_201 | 52 | 183 | + | hypothetical protein |
| contig_201 | 3345 | 340 | - | internalin, putative |
| contig_201 | 5314 | 3329 | - | internalin, putative |
| contig_201 | 6296 | 5271 | - | internalin, putative |
| contig_201 | 8877 | 6271 | - | internalin, putative |
| contig_201 | 9289 | 8888 | - | internalin, putative |
| contig_201 | 9462 | 9671 | + | FIG01230289: hypothetical protein |
| contig_201 | 10407 | 9703 | - | internalin, putative |
| contig_2010 | 233 | 424 | + | hypothetical protein |
| contig_2010 | 421 | 930 | + | 3-oxoacyl-[acyl-carrier protein] reductase (EC 1.1.1.100) |
| contig_2010 | 1079 | 1369 | + | O-methyltransferase (EC 2.1.1.-) |
| contig_2010 | 1335 | 1517 | + | O-methyltransferase (EC 2.1.1.-) |
| contig_2010 | 1600 | 1752 | + | O-methyltransferase (EC 2.1.1.-) |
| contig_2010 | 1885 | 2241 | + | Aminoglycoside 6-adenylyltransferase (EC 2.7.7.-) |
| contig_2010 | 2375 | 2755 | + | Aminoglycoside 6-adenylyltransferase (EC 2.7.7.-) |
| contig_2010 | 2873 | 3268 | + | Peptidoglycan N-acetylglucosamine deacetylase (EC 3.5.1.-) |
| contig_2010 | 3219 | 3383 | + | Peptidoglycan N-acetylglucosamine deacetylase (EC 3.5.1.-) |
| contig_2010 | 3467 | 3706 | + | Peptidoglycan N-acetylglucosamine deacetylase (EC 3.5.1.-) |
| contig_2010 | 3779 | 4117 | + | FIG01230671: hypothetical protein |
| contig_2010 | 4706 | 4170 | - | Phosphohydrolase (MutT/nudix family protein) |
| contig_2010 | 4823 | 4942 | + | hypothetical protein |
| contig_2013 | 218 | 472 | + | Urease beta subunit (EC 3.5.1.5) |
| contig_2013 | 486 | 2069 | + | Urease alpha subunit (EC 3.5.1.5) |
| contig_2013 | 2056 | 2208 | + | Urease alpha subunit (EC 3.5.1.5) |
| contig_2014 | 19 | 177 | + | Urease alpha subunit (EC 3.5.1.5) |
| contig_2016 | 157 | 318 | + | ThiJ/PfpI family protein |
| contig_2016 | 312 | 710 | + | ThiJ/PfpI family protein |
| contig_2016 | 1212 | 1568 | + | cgeb protein |
| contig_2016 | 1622 | 1996 | + | cgeb protein |
| contig_2017 | 662 | 87 | - | FAD-binding monooxygenase, PheA/TfdB family |
| contig_2017 | 1611 | 697 | - | FAD-binding monooxygenase, PheA/TfdB family |
| contig_2018 | 99 | 866 | + | 3-dehydroquinate synthase (EC 4.2.3.4) |
| contig_2018 | 988 | 1698 | + | Biosynthetic Aromatic amino acid aminotransferase beta (EC 2.6.1.57) @ Histidinol-phosphate aminotransferase (EC 2.6.1.9) |
| contig_2018 | 1811 | 2101 | + | Biosynthetic Aromatic amino acid aminotransferase beta (EC 2.6.1.57) @ Histidinol-phosphate aminotransferase (EC 2.6.1.9) |
| contig_2019 | 726 | 244 | - | FIG01231047: hypothetical protein |
| contig_2019 | 1130 | 1014 | - | membrane protein, putative |
| contig_2019 | 1377 | 1258 | - | hypothetical protein |
| contig_202 | 805 | 20 | - | Uncharacterized protein Bsub YpbR |
| contig_202 | 1284 | 853 | - | Uncharacterized protein Bsub YpbR |
| contig_202 | 1964 | 1281 | - | Uncharacterized protein Bsub YpbR |
| contig_202 | 2429 | 2268 | - | FIG01226880: hypothetical protein |
| contig_202 | 2614 | 3009 | + | membrane protein, putative |
| contig_202 | 3092 | 3670 | + | membrane protein, putative |
| contig_2020 | 532 | 335 | - | Periplasmic thiol:disulfide interchange protein DsbA |
| contig_2022 | 59 | 637 | + | CotS-related protein |
| contig_2023 | 185 | 823 | + | putative membrane protein |
| contig_2023 | 888 | 1259 | + | putative membrane protein |
| contig_2024 | 662 | 255 | - | D-alanyl-D-alanine carboxypeptidase (EC 3.4.16.4) |
| contig_2024 | 846 | 667 | - | D-alanyl-D-alanine carboxypeptidase (EC 3.4.16.4) |
| contig_2024 | 1210 | 1040 | - | acetyltransferase, GNAT family |
| contig_2028 | 167 | 21 | - | Flagellar motor rotation protein MotB |
| contig_2028 | 701 | 420 | - | Flagellar motor rotation protein MotB |
| contig_2028 | 840 | 724 | - | Flagellar motor rotation protein MotA |
| contig_2028 | 904 | 791 | - | Flagellar motor rotation protein MotA |
| contig_2028 | 1158 | 886 | - | Flagellar motor rotation protein MotA |
| contig_203 | 156 | 860 | + | Cellobiose phosphotransferase system YdjC-like protein |
| contig_203 | 2082 | 901 | - | Chromate transport protein ChrA |
| contig_203 | 2272 | 2475 | + | FIG01226154: hypothetical protein |
| contig_203 | 2457 | 2582 | + | FIG01226154: hypothetical protein |
| contig_203 | 2539 | 2673 | + | FIG01226154: hypothetical protein |
| contig_203 | 3313 | 2867 | - | NtrC family Transcriptional regulator, ATPase domain |
| contig_203 | 3831 | 3436 | - | NtrC family Transcriptional regulator, ATPase domain |
| contig_203 | 4588 | 3851 | - | NtrC family Transcriptional regulator, ATPase domain |
| contig_203 | 5348 | 4593 | - | NtrC family Transcriptional regulator, ATPase domain |
| contig_2031 | 590 | 156 | - | Outer membrane protein romA |
| contig_2031 | 1017 | 1148 | + | hypothetical protein |
| contig_2034 | 350 | 183 | - | hypothetical protein |
| contig_2034 | 669 | 340 | - | Glycosyl transferase, group 2 family protein |
| contig_2034 | 1340 | 690 | - | Glycosyl transferase, group 2 family protein |
| contig_2034 | 1921 | 1397 | - | Glycosyl transferase, group 2 family protein |
| contig_2034 | 2096 | 1902 | - | FIG01225183: hypothetical protein |
| contig_2034 | 2262 | 2113 | - | FIG01226904: hypothetical protein |
| contig_2035 | 510 | 286 | - | Putative metal-dependent hydrolase BCE33L2441 (EC 3.-.-.-) |
| contig_2035 | 845 | 642 | - | Putative metal-dependent hydrolase BCE33L2441 (EC 3.-.-.-) |
| contig_2036 | 278 | 159 | - | Protein of unknown function DUF419 |
| contig_2036 | 840 | 670 | - | GCN5-related N-acetyltransferase |
| contig_2036 | 950 | 834 | - | GCN5-related N-acetyltransferase |
| contig_2037 | 159 | 419 | + | FIG01225193: hypothetical protein |
| contig_2037 | 400 | 573 | + | FIG01225193: hypothetical protein |
| contig_2037 | 566 | 703 | + | FIG01225193: hypothetical protein |
| contig_2037 | 1066 | 857 | - | Chemotaxis protein methyltransferase CheR (EC 2.1.1.80) |
| contig_2037 | 1643 | 1350 | - | Chemotaxis protein methyltransferase CheR (EC 2.1.1.80) |
| contig_2037 | 1870 | 1730 | - | FIG01227463: hypothetical protein |
| contig_2038 | 2041 | 1361 | - | membrane protein, MmpL family |
| contig_2039 | 325 | 149 | - | GTP-binding protein HflX |
| contig_2039 | 418 | 699 | + | membrane protein, putative |
| contig_2039 | 699 | 923 | + | membrane protein, putative |
| contig_204 | 41 | 112 | + | tRNA-Gly-GCC |
| contig_204 | 123 | 195 | + | tRNA-Ala-TGC |
| contig_204 | 312 | 1457 | + | Glycerate kinase (EC 2.7.1.31) |
| contig_204 | 1666 | 2559 | + | Arginase (EC 3.5.3.1) |
| contig_2040 | 429 | 121 | - | FIG01226315: hypothetical protein |
| contig_2040 | 704 | 1213 | + | Hydroxylamine reductase (EC 1.7.-.-) |
| contig_2041 | 651 | 463 | - | amidase family protein |
| contig_2041 | 1108 | 854 | - | amidase family protein |
| contig_2042 | 313 | 182 | - | Protoporphyrinogen IX oxidase, aerobic, HemY (EC 1.3.3.4) |
| contig_2042 | 1585 | 656 | - | Protoporphyrinogen IX oxidase, aerobic, HemY (EC 1.3.3.4) |
| contig_2042 | 1872 | 1630 | - | hydrolase, alpha/beta fold family |
| contig_2042 | 2069 | 1923 | - | hydrolase, alpha/beta fold family |
| contig_2043 | 294 | 61 | - | RNA polymerase sigma-70 factor, ECF subfamily |
| contig_2046 | 763 | 122 | - | Transcriptional regulator TetR family |
| contig_2049 | 500 | 105 | - | Two-component sensor kinase YvcQ |
| contig_205 | 948 | 76 | - | Glutamate racemase (EC 5.1.1.3) |
| contig_205 | 1462 | 1830 | + | Arginine/ornithine antiporter ArcD |
| contig_205 | 1827 | 2774 | + | Arginine/ornithine antiporter ArcD |
| contig_2050 | 277 | 161 | - | Transcriptional regulator, TetR family |
| contig_2050 | 700 | 332 | - | Transcriptional regulator, TetR family |
| contig_2051 | 140 | 421 | + | Bile acid sodium symporter |
| contig_2051 | 806 | 1321 | + | Uncharacterised protein family UPF0157 (COG2320) |
| contig_2052 | 280 | 399 | + | FIG022979: MoxR-like ATPases |
| contig_2052 | 408 | 833 | + | FIG022979: MoxR-like ATPases |
| contig_2053 | 198 | 31 | - | bacitracin ABC transporter, ATP-binding protein |
| contig_2054 | 18 | 260 | + | Cell wall surface anchor family protein |
| contig_2058 | 693 | 28 | - | FIG01249387: hypothetical protein |
| contig_2059 | 89 | 451 | + | Mannose-6-phosphate isomerase (EC 5.3.1.8) |
| contig_2059 | 458 | 808 | + | Autoinducer 2 (AI-2) aldolase LsrF (EC 4.2.1.-) |
| contig_2059 | 780 | 914 | + | Autoinducer 2 (AI-2) aldolase LsrF (EC 4.2.1.-) |
| contig_2059 | 889 | 1242 | + | Autoinducer 2 (AI-2) aldolase LsrF (EC 4.2.1.-) |
| contig_2059 | 1855 | 1352 | - | Ribosomal-protein-alanine acetyltransferase( EC:2.3.1.128 ) |
| contig_206 | 364 | 963 | + | Sporulation kinase B homolog 2 |
| contig_206 | 1319 | 1627 | + | Sporulation kinase B homolog 2 |
| contig_206 | 2060 | 1818 | - | FIG01225854: hypothetical protein |
| contig_206 | 2280 | 2113 | - | FIG01225854: hypothetical protein |
| contig_206 | 3028 | 2495 | - | Oligoendopeptidase F (EC 3.4.24.-) |
| contig_206 | 3312 | 3127 | - | Oligoendopeptidase F (EC 3.4.24.-) |
| contig_206 | 3743 | 3438 | - | Oligoendopeptidase F (EC 3.4.24.-) |
| contig_206 | 4036 | 3740 | - | Oligoendopeptidase F (EC 3.4.24.-) |
| contig_206 | 4786 | 4361 | - | Transcriptional regulator, MarR family |
| contig_206 | 4992 | 5111 | + | sensor histidine kinase KinD |
| contig_2060 | 46 | 294 | + | N-acetyl-L,L-diaminopimelate aminotransferase (EC 2.6.1.-) |
| contig_2060 | 799 | 299 | - | Maltose operon transcriptional repressor MalR, LacI family |
| contig_2061 | 283 | 131 | - | Acetamidase (EC 3.5.1.4) |
| contig_2061 | 428 | 258 | - | Acetamidase (EC 3.5.1.4) |
| contig_2062 | 328 | 489 | + | Excinuclease ABC subunit A domain protein |
| contig_2062 | 494 | 763 | + | Excinuclease ABC subunit A domain protein |
| contig_2063 | 427 | 1509 | + | amino acid permease family protein |
| contig_2064 | 408 | 848 | + | FIG01228339: hypothetical protein |
| contig_2065 | 48 | 182 | + | hypothetical protein |
| contig_2065 | 356 | 195 | - | enterotoxin / cell-wall binding protein |
| contig_2066 | 188 | 670 | + | Response regulator of the competence regulon ComE |
| contig_2067 | 31 | 171 | + | Multi antimicrobial extrusion protein (Na(+)/drug antiporter), MATE family of MDR efflux pumps |
| contig_2068 | 26 | 556 | + | Formate--tetrahydrofolate ligase (EC 6.3.4.3) |
| contig_2068 | 788 | 928 | + | Formate--tetrahydrofolate ligase (EC 6.3.4.3) |
| contig_2068 | 1187 | 1447 | + | Bacillus cereus group-specific protein, uncharacterized |
| contig_2068 | 1449 | 1574 | + | Bacillus cereus group-specific protein, uncharacterized |
| contig_2068 | 1543 | 1716 | + | Bacillus cereus group-specific protein, uncharacterized |
| contig_207 | 84 | 209 | + | Scaffold protein for [4Fe-4S] cluster assembly ApbC, MRP-like |
| contig_207 | 306 | 758 | + | Scaffold protein for [4Fe-4S] cluster assembly ApbC, MRP-like |
| contig_207 | 802 | 1137 | + | Scaffold protein for [4Fe-4S] cluster assembly ApbC, MRP-like |
| contig_2070 | 200 | 9 | - | Thioredoxin |
| contig_2072 | 381 | 509 | + | conserved membrane-spanning protein |
| contig_2074 | 37 | 195 | + | L-serine dehydratase, alpha subunit (EC 4.3.1.17) |
| contig_2074 | 171 | 446 | + | L-serine dehydratase, alpha subunit (EC 4.3.1.17) |
| contig_2075 | 539 | 270 | - | Ribonucleotide reductase of class Ib (aerobic), alpha subunit (EC 1.17.4.1) |
| contig_2075 | 687 | 562 | - | Ribonucleotide reduction protein NrdI |
| contig_2075 | 923 | 705 | - | Ribonucleotide reduction protein NrdI |
| contig_2075 | 1725 | 1408 | - | FIG01225430: hypothetical protein |
| contig_2075 | 1946 | 1737 | - | FIG01225327: hypothetical protein |
| contig_2075 | 3256 | 2816 | - | FIG01227049: hypothetical protein |
| contig_2075 | 3416 | 3240 | - | FIG01227049: hypothetical protein |
| contig_2075 | 3666 | 3496 | - | hypothetical protein |
| contig_2077 | 141 | 446 | + | membrane protein, putative |
| contig_2077 | 505 | 642 | + | membrane protein, putative |
| contig_2077 | 805 | 987 | + | Aquaporin Z |
| contig_2077 | 1034 | 1447 | + | Aquaporin Z |
| contig_2078 | 193 | 402 | + | FIG01226725: hypothetical protein |
| contig_2078 | 1224 | 805 | - | SAM-dependent methyltransferase BA1462 (UbiE paralog) |
| contig_2079 | 165 | 19 | - | 2,3-butanediol dehydrogenase, R-alcohol forming, (R)- and (S)-acetoin-specific (EC 1.1.1.4) |
| contig_208 | 266 | 60 | - | COG0553: Superfamily II DNA/RNA helicases, SNF2 family |
| contig_208 | 1024 | 428 | - | COG0553: Superfamily II DNA/RNA helicases, SNF2 family |
| contig_208 | 2118 | 1258 | - | COG0553: Superfamily II DNA/RNA helicases, SNF2 family |
| contig_208 | 2635 | 2102 | - | COG0553: Superfamily II DNA/RNA helicases, SNF2 family |
| contig_208 | 3182 | 2628 | - | COG0553: Superfamily II DNA/RNA helicases, SNF2 family |
| contig_208 | 3291 | 3160 | - | COG0553: Superfamily II DNA/RNA helicases, SNF2 family |
| contig_2082 | 584 | 255 | - | penicillin-binding protein, putative |
| contig_2083 | 343 | 143 | - | FIG01226181: hypothetical protein |
| contig_2083 | 538 | 1722 | + | DNA polymerase IV (EC 2.7.7.7) |
| contig_2083 | 2070 | 1846 | - | Mg(2+) transport ATPase protein C |
| contig_2083 | 2470 | 2039 | - | Mg(2+) transport ATPase protein C |
| contig_2084 | 75 | 308 | + | FIG01225489: hypothetical protein |
| contig_2086 | 678 | 478 | - | hypothetical protein |
| contig_2086 | 1577 | 1690 | + | hypothetical protein |
| contig_2088 | 443 | 111 | - | Stage IV sporulation protein |
| contig_2088 | 558 | 418 | - | Stage IV sporulation protein |
| contig_2088 | 754 | 530 | - | Stage IV sporulation protein |
| contig_209 | 58 | 312 | + | Inosine-uridine preferring nucleoside hydrolase (EC 3.2.2.1) |
| contig_209 | 532 | 302 | - | Enolase (EC 4.2.1.11) |
| contig_209 | 1598 | 687 | - | Enolase (EC 4.2.1.11) |
| contig_209 | 1988 | 1629 | - | 2,3-bisphosphoglycerate-independent phosphoglycerate mutase (EC 5.4.2.1) |
| contig_209 | 3160 | 1994 | - | 2,3-bisphosphoglycerate-independent phosphoglycerate mutase (EC 5.4.2.1) |
| contig_209 | 3912 | 3157 | - | Triosephosphate isomerase (EC 5.3.1.1) |
| contig_209 | 5129 | 3945 | - | Phosphoglycerate kinase (EC 2.7.2.3) |
| contig_209 | 5883 | 5269 | - | NAD-dependent glyceraldehyde-3-phosphate dehydrogenase (EC 1.2.1.12) |
| contig_209 | 6274 | 6161 | - | NAD-dependent glyceraldehyde-3-phosphate dehydrogenase (EC 1.2.1.12) |
| contig_209 | 7329 | 6301 | - | Central glycolytic genes regulator |
| contig_209 | 7669 | 7517 | - | glutaredoxin family protein |
| contig_209 | 7827 | 7666 | - | RNA polymerase sigma-54 factor RpoN |
| contig_209 | 9031 | 7874 | - | RNA polymerase sigma-54 factor RpoN |
| contig_209 | 9227 | 9526 | + | HlyC domain protein |
| contig_209 | 10160 | 9543 | - | FIG01226721: hypothetical protein |
| contig_209 | 10321 | 10437 | + | FIG01226246: hypothetical protein |
| contig_2090 | 163 | 26 | - | pXO1-52 |
| contig_2091 | 656 | 18 | - | FIG038982: hypothetical protein |
| contig_2091 | 820 | 653 | - | FIG004131: Transcriptional regulator, ArsR family |
| contig_2093 | 659 | 138 | - | hypothetical protein |
| contig_2095 | 392 | 273 | - | hypothetical protein |
| contig_2096 | 1131 | 232 | - | Niacin transporter NiaP |
| contig_2097 | 79 | 363 | + | UDP-galactose-lipid carrier transferase (EC 2.-.-.-) |
| contig_2097 | 353 | 754 | + | UDP-galactose-lipid carrier transferase (EC 2.-.-.-) |
| contig_2099 | 100 | 222 | + | hypothetical protein |
| contig_2099 | 877 | 188 | - | Probable lipase/esterase |
| contig_2099 | 1100 | 981 | - | Probable lipase/esterase |
| contig_21 | 649 | 182 | - | Transcriptional regulator, GntR family domain / Aspartate aminotransferase (EC 2.6.1.1) |
| contig_21 | 1314 | 619 | - | Transcriptional regulator, GntR family domain / Aspartate aminotransferase (EC 2.6.1.1) |
| contig_21 | 1433 | 1582 | + | Transporter, LysE family |
| contig_21 | 1596 | 2048 | + | Transporter, LysE family |
| contig_21 | 2491 | 2102 | - | Siderophore transport protein |
| contig_210 | 141 | 425 | + | FIGfam010146: Two component system histidine kinase |
| contig_210 | 491 | 1273 | + | FIGfam012970: transmembrane protein |
| contig_210 | 1270 | 2304 | + | FIGfam013899: ABC transporter substrate-binding protein |
| contig_210 | 2324 | 3466 | + | Phosphoglycerate transporter protein PgtP |
| contig_210 | 3481 | 3663 | + | Phosphoglycerate transporter protein PgtP |
| contig_210 | 3902 | 4864 | + | periplasmic component of efflux system |
| contig_210 | 4882 | 5094 | + | periplasmic component of efflux system |
| contig_210 | 5091 | 5771 | + | Cell division transporter, ATP-binding protein FtsE (TC 3.A.5.1.1) |
| contig_210 | 5768 | 6106 | + | ABC transporter, permease protein |
| contig_210 | 6088 | 6969 | + | ABC transporter, permease protein |
| contig_210 | 7083 | 7400 | + | hypothetical Membrane Spanning Protein |
| contig_210 | 7357 | 7674 | + | hypothetical Membrane Spanning Protein |
| contig_210 | 7649 | 7765 | + | hypothetical Membrane Spanning Protein |
| contig_210 | 7984 | 8346 | + | RNA polymerase sigma factor |
| contig_210 | 9016 | 8669 | - | Competence transcription factor, degenerate |
| contig_210 | 9212 | 9610 | + | Bacillus cereus group-specific protein, uncharacterized |
| contig_2102 | 279 | 55 | - | hypothetical protein |
| contig_2102 | 442 | 302 | - | FIG01248155: hypothetical protein |
| contig_2104 | 390 | 145 | - | D-alanyl-D-alanine carboxypeptidase (EC 3.4.16.4) |
| contig_2105 | 737 | 1024 | + | drug resistance transporter, Bcr/CflA family |
| contig_2105 | 982 | 1122 | + | hypothetical protein |
| contig_2108 | 734 | 129 | - | Outer surface protein of unknown function, cellobiose operon |
| contig_2109 | 220 | 71 | - | Glutathione-dependent formaldehyde dehydrogenase |
| contig_211 | 300 | 1382 | + | Amino acid permease family protein |
| contig_211 | 2023 | 1529 | - | permease, putative |
| contig_2110 | 98 | 319 | + | hydrolase, alpha/beta fold family |
| contig_2112 | 206 | 78 | - | glyoxalase family protein, putative |
| contig_2113 | 63 | 236 | + | Exosporium protein Y @ Spore coat protein Y |
| contig_2115 | 453 | 629 | + | hypothetical protein |
| contig_2116 | 654 | 118 | - | Protein-L-isoD(D-D) O-methyltransferase |
| contig_2116 | 895 | 617 | - | Protein-L-isoD(D-D) O-methyltransferase |
| contig_2116 | 1355 | 945 | - | FIG01226173: hypothetical protein |
| contig_2119 | 235 | 71 | - | SH3 domain protein |
| contig_212 | 171 | 31 | - | Thioredoxin |
| contig_212 | 737 | 588 | - | FIG074102: hypothetical protein |
| contig_2120 | 568 | 383 | - | Glycosyl transferase ,group 2 family, anthrose biosynthesis |
| contig_2121 | 199 | 2 | - | sensor histidine kinase |
| contig_2121 | 629 | 189 | - | Phosphate regulon transcriptional regulatory protein PhoB (SphR) |
| contig_2121 | 891 | 763 | - | Phosphate regulon transcriptional regulatory protein PhoB (SphR) |
| contig_2121 | 1084 | 1566 | + | HMP-PP hydrolase (pyridoxal phosphatase) Cof, detected in genetic screen for thiamin metabolic genes (PMID:15292217) |
| contig_2122 | 19 | 297 | + | GTP-binding protein HflX |
| contig_2123 | 206 | 33 | - | hypothetical protein |
| contig_2124 | 284 | 6 | - | Cytidine deaminase (EC 3.5.4.5) |
| contig_2125 | 55 | 195 | + | hypothetical protein |
| contig_2125 | 622 | 416 | - | hypothetical protein |
| contig_2126 | 161 | 6 | - | Ribonuclease Z (EC 3.1.26.11) |
| contig_2126 | 531 | 115 | - | Ribonuclease Z (EC 3.1.26.11) |
| contig_2128 | 27 | 173 | + | MaoC like domain protein |
| contig_213 | 181 | 447 | + | ABC transporter ATP-binding protein uup |
| contig_213 | 513 | 728 | + | ABC transporter ATP-binding protein uup |
| contig_213 | 760 | 993 | + | ABC transporter ATP-binding protein uup |
| contig_213 | 1050 | 1742 | + | ABC transporter ATP-binding protein uup |
| contig_213 | 1814 | 1954 | + | ABC transporter ATP-binding protein uup |
| contig_213 | 1926 | 2132 | + | ABC transporter ATP-binding protein uup |
| contig_213 | 3509 | 2553 | - | TsaD/Kae1/Qri7 protein, required for threonylcarbamoyladenosine t(6)A37 formation in tRNA |
| contig_213 | 4014 | 3571 | - | Ribosomal-protein-S18p-alanine acetyltransferase (EC 2.3.1.-) |
| contig_213 | 4720 | 4028 | - | TsaB protein, required for threonylcarbamoyladenosine (t(6)A) formation in tRNA |
| contig_213 | 5015 | 4701 | - | TsaE protein, required for threonylcarbamoyladenosine t(6)A37 formation in tRNA |
| contig_2132 | 911 | 561 | - | Transcriptional regulator, PadR family |
| contig_2134 | 201 | 70 | - | Acetylornithine deacetylase (EC 3.5.1.16) |
| contig_2137 | 52 | 456 | + | sodium-dependent transporter, putative |
| contig_2137 | 503 | 730 | + | sodium-dependent transporter, putative |
| contig_2137 | 747 | 1148 | + | sodium-dependent transporter, putative |
| contig_2138 | 1428 | 46 | - | Long-chain-fatty-acid--CoA ligase (EC 6.2.1.3) |
| contig_2138 | 1936 | 1691 | - | NAD(P)H oxidoreductase YRKL (EC 1.6.99.-) @ Putative NADPH-quinone reductase (modulator of drug activity B) @ Flavodoxin 2 |
| contig_2138 | 2223 | 1972 | - | NAD(P)H oxidoreductase YRKL (EC 1.6.99.-) @ Putative NADPH-quinone reductase (modulator of drug activity B) @ Flavodoxin 2 |
| contig_2139 | 207 | 79 | - | hypothetical protein |
| contig_2139 | 822 | 403 | - | Deoxyribodipyrimidine photolyase (EC 4.1.99.3) |
| contig_214 | 1438 | 8 | - | Mobile element protein |
| contig_2141 | 372 | 4 | - | FIG01227415: hypothetical protein |
| contig_2141 | 718 | 335 | - | FIG01225154: hypothetical protein |
| contig_2141 | 929 | 681 | - | FIG01225154: hypothetical protein |
| contig_2141 | 1335 | 955 | - | FIG01225154: hypothetical protein |
| contig_2142 | 277 | 942 | + | Multidrug-efflux transporter, major facilitator superfamily (MFS) (TC 2.A.1); Efflux pump Lde |
| contig_2142 | 1100 | 1306 | + | Multidrug-efflux transporter, major facilitator superfamily (MFS) (TC 2.A.1); Efflux pump Lde |
| contig_2142 | 1700 | 1539 | - | COGs COG3339 |
| contig_2143 | 1345 | 1076 | - | Alpha-glucosidase (EC 3.2.1.20) |
| contig_2145 | 247 | 119 | - | Negative regulator of genetic competence MecA |
| contig_2146 | 983 | 588 | - | Cytochrome oxidase biogenesis protein Sco1/SenC/PrrC, putative copper metallochaperone |
| contig_2149 | 310 | 23 | - | 3-oxoacyl-[acyl-carrier protein] reductase paralog (EC 1.1.1.100) |
| contig_215 | 167 | 21 | - | hypothetical protein |
| contig_215 | 3449 | 2919 | - | DNA integration/recombination/inversion protein |
| contig_215 | 3534 | 3418 | - | DNA integration/recombination/inversion protein |
| contig_215 | 4267 | 4416 | + | putative membrane protein |
| contig_215 | 4426 | 4656 | + | Transcriptional regulator, Cro/CI family |
| contig_215 | 4754 | 5473 | + | FIG01231988: hypothetical protein |
| contig_2151 | 26 | 184 | + | Cys regulon transcriptional activator CysB |
| contig_2151 | 216 | 863 | + | Cys regulon transcriptional activator CysB |
| contig_2153 | 674 | 21 | - | Flavohemoprotein (Hemoglobin-like protein) (Flavohemoglobin) (Nitric oxide dioxygenase) (EC 1.14.12.17) |
| contig_2153 | 1202 | 984 | - | acetyl esterase, putative |
| contig_2153 | 1648 | 1199 | - | acetyl esterase, putative |
| contig_2154 | 428 | 177 | - | FIG01228316: hypothetical protein |
| contig_2155 | 26 | 841 | + | 3-hydroxyisobutyryl-CoA hydrolase (EC 3.1.2.4) |
| contig_2157 | 322 | 182 | - | Methyltransferase (EC 2.1.1.-) |
| contig_2158 | 676 | 173 | - | Dipicolinate synthase subunit B |
| contig_2158 | 1564 | 821 | - | Dipicolinate synthase subunit A (EC 4.2.1.52) |
| contig_2158 | 1667 | 1828 | + | FIG01237453: hypothetical protein |
| contig_2158 | 1990 | 1814 | - | FIG007697: hypothetical protein |
| contig_216 | 720 | 61 | - | Acetyl-coenzyme A carboxyl transferase alpha chain (EC 6.4.1.2) |
| contig_216 | 1034 | 711 | - | Acetyl-coenzyme A carboxyl transferase alpha chain (EC 6.4.1.2) |
| contig_216 | 1891 | 1022 | - | Acetyl-coenzyme A carboxyl transferase beta chain (EC 6.4.1.2) |
| contig_216 | 2722 | 2078 | - | Transcriptional regulator, GntR family |
| contig_216 | 3606 | 2737 | - | NADP-dependent malic enzyme (EC 1.1.1.40) |
| contig_216 | 3938 | 3630 | - | NADP-dependent malic enzyme (EC 1.1.1.40) |
| contig_216 | 6836 | 4083 | - | DNA polymerase III alpha subunit (EC 2.7.7.7) |
| contig_216 | 7306 | 7163 | - | DNA polymerase III alpha subunit (EC 2.7.7.7) |
| contig_216 | 7425 | 7303 | - | DNA polymerase III alpha subunit (EC 2.7.7.7) |
| contig_216 | 7537 | 7866 | + | FIG012070: hypothetical protein |
| contig_216 | 7880 | 8005 | + | FIG013210: hypothetical protein |
| contig_216 | 7968 | 8252 | + | FIG013210: hypothetical protein |
| contig_216 | 8427 | 8296 | - | hypothetical protein |
| contig_216 | 8741 | 8436 | - | FIG146085: 3'-to-5' oligoribonuclease A, Bacillus type |
| contig_216 | 9369 | 8713 | - | FIG146085: 3'-to-5' oligoribonuclease A, Bacillus type |
| contig_216 | 9575 | 9399 | - | FIG01241355: hypothetical protein |
| contig_2161 | 223 | 65 | - | Arginine deiminase (EC 3.5.3.6) |
| contig_2161 | 991 | 674 | - | Transcriptional regulator ArcR essential for anaerobic expression of the ADI pathway, Crp/Fnr family |
| contig_2163 | 64 | 222 | + | 3-oxoacyl-[acyl-carrier protein] reductase (EC 1.1.1.100) |
| contig_2164 | 332 | 75 | - | LPXTG-motif cell wall anchor domain protein |
| contig_2165 | 945 | 1058 | + | FIG01229044: hypothetical protein |
| contig_2165 | 1139 | 1810 | + | FIG01229044: hypothetical protein |
| contig_2165 | 1773 | 2051 | + | FIG01229044: hypothetical protein |
| contig_2168 | 1304 | 1002 | - | multidrug ABC transporter, ATP-binding protein |
| contig_2168 | 1611 | 1321 | - | multidrug ABC transporter, ATP-binding protein |
| contig_2168 | 1714 | 1601 | - | multidrug ABC transporter, ATP-binding protein |
| contig_217 | 1 | 309 | + | Duplicated ATPase component CbrU of energizing module of predicted cobalamin ECF transporter |
| contig_217 | 309 | 1001 | + | Duplicated ATPase component CbrU of energizing module of predicted cobalamin ECF transporter |
| contig_217 | 992 | 1402 | + | Duplicated ATPase component CbrU of energizing module of predicted cobalamin ECF transporter |
| contig_2170 | 960 | 694 | - | RNA polymerase sigma factor SigZ |
| contig_2170 | 1259 | 1080 | - | RNA polymerase sigma factor SigZ |
| contig_2172 | 512 | 192 | - | Butyryl-CoA dehydrogenase (EC 1.3.8.1) |
| contig_2172 | 553 | 675 | + | FIG01232305: hypothetical protein |
| contig_2172 | 926 | 669 | - | Methylisocitrate lyase (EC 4.1.3.30) |
| contig_2172 | 1068 | 904 | - | Methylisocitrate lyase (EC 4.1.3.30) |
| contig_2172 | 1496 | 1098 | - | Methylisocitrate lyase (EC 4.1.3.30) |
| contig_2172 | 2484 | 1597 | - | 2-methylcitrate dehydratase (EC 4.2.1.79) |
| contig_2173 | 460 | 125 | - | Flagellar biosynthesis protein FlhB |
| contig_2173 | 701 | 471 | - | Flagellar biosynthesis protein FliR |
| contig_2174 | 331 | 11 | - | Cell envelope-associated transcriptional attenuator LytR-CpsA-Psr, subfamily F2 (as in PMID19099556) |
| contig_2174 | 738 | 349 | - | ECF-type sigma factor negative effector |
| contig_2174 | 976 | 827 | - | ECF-type sigma factor negative effector |
| contig_2174 | 1287 | 1024 | - | ECF-type sigma factor negative effector |
| contig_2175 | 13 | 168 | + | Methylmalonate-semialdehyde dehydrogenase (EC 1.2.1.27) |
| contig_2175 | 149 | 337 | + | Methylmalonate-semialdehyde dehydrogenase (EC 1.2.1.27) |
| contig_2175 | 920 | 435 | - | Putative threonine efflux protein |
| contig_2176 | 322 | 8 | - | Pullulanase (EC 3.2.1.41) |
| contig_2176 | 672 | 328 | - | Pullulanase (EC 3.2.1.41) |
| contig_2178 | 430 | 8 | - | Polyphosphate kinase (EC 2.7.4.1) |
| contig_2178 | 641 | 423 | - | Polyphosphate kinase (EC 2.7.4.1) |
| contig_2178 | 1345 | 734 | - | Polyphosphate kinase (EC 2.7.4.1) |
| contig_218 | 65 | 307 | + | regulatory protein RecX |
| contig_218 | 317 | 634 | + | Transcription regulator CDS_ID OB0894 |
| contig_218 | 854 | 681 | - | FIG01225538: hypothetical protein |
| contig_218 | 1073 | 954 | - | FIG01227190: hypothetical protein |
| contig_218 | 1174 | 1329 | + | Conserved protein YfhJ |
| contig_218 | 1322 | 1441 | + | Conserved protein YfhJ |
| contig_2180 | 682 | 50 | - | 2',3'-cyclic-nucleotide 2'-phosphodiesterase (EC 3.1.4.16) |
| contig_2182 | 790 | 623 | - | heat shock protein, Hsp20 family |
| contig_2183 | 321 | 100 | - | Autoinducer 2 (AI-2) ABC transport system, fused AI2 transporter subunits and ATP-binding component |
| contig_219 | 485 | 57 | - | Peroxide stress regulator PerR, FUR family |
| contig_219 | 1243 | 836 | - | Thiol peroxidase, Bcp-type (EC 1.11.1.15) |
| contig_219 | 1569 | 1288 | - | Potassium channel protein |
| contig_219 | 1702 | 1586 | - | Potassium channel protein |
| contig_219 | 2551 | 1766 | - | ABC transporter, permease protein, putative |
| contig_219 | 3104 | 2556 | - | Daunorubicin resistance transmembrane protein |
| contig_219 | 3348 | 3127 | - | Daunorubicin resistance transmembrane protein |
| contig_219 | 3865 | 3341 | - | ABC transporter, ATP-binding protein |
| contig_219 | 4355 | 3897 | - | ABC transporter, ATP-binding protein |
| contig_219 | 4477 | 5778 | + | Glutamate-1-semialdehyde aminotransferase (EC 5.4.3.8) |
| contig_2193 | 538 | 119 | - | membrane protein, MmpL family |
| contig_2193 | 818 | 621 | - | membrane protein, MmpL family |
| contig_2194 | 203 | 63 | - | FIG01228356: hypothetical protein |
| contig_2194 | 316 | 200 | - | FIG01228356: hypothetical protein |
| contig_2195 | 218 | 12 | - | Protein export cytoplasm protein SecA ATPase RNA helicase (TC 3.A.5.1.1) |
| contig_2195 | 467 | 354 | - | Protein export cytoplasm protein SecA ATPase RNA helicase (TC 3.A.5.1.1) |
| contig_2196 | 702 | 238 | - | Arsenic efflux pump protein |
| contig_2197 | 194 | 9 | - | fructose response regulator of fruA and EII fructose/mannose |
| contig_2198 | 195 | 82 | - | hypothetical protein |
| contig_2199 | 94 | 207 | + | Chloramphenicol acetyltransferase (EC 2.3.1.28) |
| contig_22 | 953 | 720 | - | pXO1-50 |
| contig_22 | 1675 | 1022 | - | pXO1-50 |
| contig_220 | 819 | 586 | - | FIG01236131: hypothetical protein |
| contig_220 | 997 | 809 | - | FIG01234619: hypothetical protein |
| contig_220 | 1106 | 981 | - | FIG01234619: hypothetical protein |
| contig_220 | 4179 | 1120 | - | FIG01229354: hypothetical protein |
| contig_2200 | 226 | 498 | + | Multiple sugar ABC transporter, ATP-binding protein |
| contig_2200 | 565 | 864 | + | Maltose/maltodextrin transport ATP-binding protein MalK (EC 3.6.3.19); Multiple sugar ABC transporter, ATP-binding protein |
| contig_2204 | 306 | 25 | - | DNA polymerase IV (EC 2.7.7.7) |
| contig_2204 | 675 | 481 | - | DNA polymerase IV (EC 2.7.7.7) |
| contig_2206 | 274 | 50 | - | FIG01226212: hypothetical protein |
| contig_2207 | 284 | 580 | + | Na+ driven multidrug efflux pump |
| contig_2208 | 249 | 55 | - | FIG01227853: hypothetical protein |
| contig_2210 | 138 | 275 | + | Transcriptional regulator, PadR family |
| contig_2212 | 716 | 483 | - | Aerobic C4-dicarboxylate transporter for fumarate, L-malate, D-malate, succunate, aspartate |
| contig_2212 | 932 | 747 | - | Aerobic C4-dicarboxylate transporter for fumarate, L-malate, D-malate, succunate, aspartate |
| contig_2212 | 1305 | 1018 | - | Aerobic C4-dicarboxylate transporter for fumarate, L-malate, D-malate, succunate, aspartate |
| contig_2213 | 414 | 1 | - | Multimodular transpeptidase-transglycosylase (EC 2.4.1.129) (EC 3.4.-.-) / Penicillin-binding protein 1A/1B (PBP1) |
| contig_2214 | 569 | 189 | - | Beta-lactamase (EC 3.5.2.6) |
| contig_2215 | 606 | 283 | - | hypothetical protein |
| contig_2216 | 994 | 221 | - | Multimodular transpeptidase-transglycosylase (EC 2.4.1.129) (EC 3.4.-.-) |
| contig_2217 | 278 | 111 | - | FIG01225991: hypothetical protein |
| contig_2217 | 540 | 364 | - | FIG01225991: hypothetical protein |
| contig_2219 | 395 | 189 | - | penicillin-binding protein, putative |
| contig_2219 | 702 | 352 | - | penicillin-binding protein, putative |
| contig_2220 | 412 | 137 | - | Spore coat protein X |
| contig_2220 | 651 | 427 | - | Spore coat protein W |
| contig_2221 | 38 | 262 | + | COG1272: Predicted membrane protein hemolysin III homolog |
| contig_2222 | 29 | 148 | + | COG1272: Predicted membrane protein hemolysin III homolog |
| contig_2222 | 114 | 230 | + | hypothetical protein |
| contig_2223 | 852 | 268 | - | Cobalt-zinc-cadmium resistance protein CzcD |
| contig_2223 | 979 | 833 | - | Cobalt-zinc-cadmium resistance protein CzcD |
| contig_2223 | 1220 | 921 | - | Cobalt-zinc-cadmium resistance protein CzcD |
| contig_2224 | 398 | 114 | - | Oligopeptide ABC transporter, periplasmic oligopeptide-binding protein OppA (TC 3.A.1.5.1) |
| contig_2225 | 737 | 195 | - | Oligopeptide ABC transporter, periplasmic oligopeptide-binding protein OppA (TC 3.A.1.5.1) |
| contig_2226 | 255 | 91 | - | Response regulator LiaR |
| contig_2227 | 37 | 225 | + | metallo-beta-lactamase family protein |
| contig_2227 | 297 | 554 | + | metallo-beta-lactamase family protein |
| contig_2228 | 866 | 711 | - | Cell envelope-associated transcriptional attenuator LytR-CpsA-Psr, subfamily F2 (as in PMID19099556) |
| contig_2229 | 40 | 333 | + | Protein of unknown function identified by role in sporulation (SpoVG) |
| contig_223 | 233 | 96 | - | Sporulation kinase (EC 2.7.3.-) |
| contig_223 | 435 | 274 | - | Sporulation kinase (EC 2.7.3.-) |
| contig_2230 | 699 | 70 | - | FIG01227722: hypothetical protein |
| contig_2230 | 805 | 683 | - | hypothetical protein |
| contig_2235 | 192 | 13 | - | hypothetical protein |
| contig_2237 | 8 | 202 | + | mutT/nudix family protein |
| contig_2237 | 641 | 459 | - | Transcriptional regulator, TetR family |
| contig_2237 | 852 | 670 | - | Transcriptional regulator, TetR family |
| contig_2237 | 1005 | 1316 | + | Kynureninase (EC 3.7.1.3) |
| contig_2238 | 383 | 177 | - | Molybdate metabolism regulator |
| contig_2238 | 1022 | 546 | - | Molybdate metabolism regulator |
| contig_2239 | 162 | 34 | - | mutT/nudix family protein |
| contig_2239 | 846 | 451 | - | D-amino acid dehydrogenase small subunit (EC 1.4.99.1) |
| contig_2239 | 1216 | 821 | - | D-amino acid dehydrogenase small subunit (EC 1.4.99.1) |
| contig_2239 | 1483 | 1241 | - | D-amino acid dehydrogenase small subunit (EC 1.4.99.1) |
| contig_2239 | 1571 | 1452 | - | hypothetical protein |
| contig_224 | 713 | 159 | - | FIG01225334: hypothetical protein |
| contig_2240 | 409 | 35 | - | FIG01229422: hypothetical protein |
| contig_2240 | 760 | 614 | - | FIG01226135: hypothetical protein |
| contig_2241 | 206 | 69 | - | Putative metal chaperone, involved in Zn homeostasis, GTPase of COG0523 family |
| contig_2241 | 423 | 271 | - | Putative metal chaperone, involved in Zn homeostasis, GTPase of COG0523 family |
| contig_2241 | 555 | 430 | - | hypothetical protein |
| contig_2241 | 850 | 527 | - | Putative metal chaperone, involved in Zn homeostasis, GTPase of COG0523 family |
| contig_2241 | 1004 | 867 | - | hypothetical protein |
| contig_2241 | 2097 | 1069 | - | Tryptophan 2-monooxygenase (EC 1.13.12.3) |
| contig_2246 | 681 | 85 | - | N-Acetyl-D-glucosamine ABC transport system, sugar-binding protein |
| contig_2247 | 516 | 223 | - | Succinyl-CoA ligase [ADP-forming] alpha chain (EC 6.2.1.5) |
| contig_225 | 76 | 558 | + | Cardiolipin synthetase (EC 2.7.8.-) |
| contig_225 | 773 | 931 | + | Cardiolipin synthetase (EC 2.7.8.-) |
| contig_225 | 1580 | 1080 | - | Uridine monophosphate kinase (EC 2.7.4.22) |
| contig_225 | 1840 | 1607 | - | Uridine monophosphate kinase (EC 2.7.4.22) |
| contig_2250 | 1047 | 1 | - | Topoisomerase IV subunit A (EC 5.99.1.-) |
| contig_2251 | 491 | 378 | - | FIG01226494: hypothetical protein |
| contig_2251 | 730 | 560 | - | FIG01226494: hypothetical protein |
| contig_2251 | 858 | 727 | - | FIG01226494: hypothetical protein |
| contig_2253 | 214 | 65 | - | Wall-associated protein precursor |
| contig_2256 | 6 | 122 | + | potassium uptake protein, TrkH family |
| contig_2259 | 173 | 54 | - | hypothetical protein |
| contig_226 | 333 | 175 | - | hypothetical protein |
| contig_226 | 667 | 518 | - | hypothetical protein |
| contig_226 | 1739 | 1539 | - | FIG01227620: hypothetical protein |
| contig_226 | 1945 | 1748 | - | L-Proline/Glycine betaine transporter ProP |
| contig_226 | 2597 | 1932 | - | L-Proline/Glycine betaine transporter ProP |
| contig_2260 | 188 | 9 | - | Penicillin-binding protein 2B |
| contig_2260 | 414 | 301 | - | Penicillin-binding protein 2B |
| contig_2261 | 569 | 24 | - | drug resistance transporter, EmrB/QacA family |
| contig_2261 | 936 | 610 | - | drug resistance transporter, EmrB/QacA family |
| contig_2261 | 1315 | 1013 | - | drug resistance transporter, EmrB/QacA family |
| contig_2261 | 1412 | 1287 | - | drug resistance transporter, EmrB/QacA family |
| contig_2262 | 351 | 109 | - | NAD-specific glutamate dehydrogenase (EC 1.4.1.2) |
| contig_2262 | 970 | 305 | - | NAD-specific glutamate dehydrogenase (EC 1.4.1.2) |
| contig_2263 | 397 | 209 | - | FIG01227559: hypothetical protein |
| contig_2265 | 424 | 257 | - | Protein distantly related to SAM-dependent methyltransferases |
| contig_2265 | 802 | 506 | - | Protein distantly related to SAM-dependent methyltransferases |
| contig_2265 | 926 | 1513 | + | hypothetical protein |
| contig_2267 | 33 | 356 | + | 2-methylcitrate synthase (EC 2.3.3.5) |
| contig_2267 | 455 | 976 | + | 2-methylcitrate dehydratase (EC 4.2.1.79) |
| contig_2268 | 370 | 257 | - | hypothetical protein |
| contig_2269 | 181 | 555 | + | FIG01234345: hypothetical protein |
| contig_227 | 1654 | 422 | - | L-Proline/Glycine betaine transporter ProP |
| contig_227 | 2215 | 1721 | - | Transcriptional regulator, TetR family |
| contig_227 | 2528 | 2385 | - | hypothetical protein |
| contig_227 | 2750 | 2619 | - | hypothetical protein |
| contig_2270 | 327 | 190 | - | COG1683: Uncharacterized conserved protein / FIG143828: Hypothetical protein YbgA |
| contig_2270 | 539 | 336 | - | COG1683: Uncharacterized conserved protein / FIG143828: Hypothetical protein YbgA |
| contig_2272 | 469 | 26 | - | Transamidase GatB domain protein |
| contig_2272 | 658 | 485 | - | SSU ribosomal protein S21p |
| contig_2274 | 469 | 257 | - | TnpA transposase |
| contig_2276 | 453 | 166 | - | Phage lysin; N-acetylmuramoyl-L-alanine amidase, family 3 (EC:3.5.1.28) |
| contig_228 | 841 | 572 | - | hypothetical protein |
| contig_2281 | 283 | 110 | - | Aminoglycoside phosphotransferase |
| contig_2281 | 514 | 296 | - | FIG01225599: hypothetical protein |
| contig_2281 | 602 | 486 | - | FIG01225599: hypothetical protein |
| contig_2281 | 933 | 652 | - | FIG01225599: hypothetical protein |
| contig_2281 | 1097 | 939 | - | FIG01225599: hypothetical protein |
| contig_2282 | 245 | 93 | - | hypothetical protein |
| contig_2282 | 744 | 1256 | + | NADH dehydrogenase, subunit 5 |
| contig_2282 | 1293 | 1571 | + | NADH dehydrogenase, subunit 5 |
| contig_2283 | 358 | 176 | - | Ribonuclease J2 (endoribonuclease in RNA processing) |
| contig_2285 | 8 | 298 | + | Glyoxalase family protein |
| contig_2285 | 268 | 774 | + | glyoxylase family protein |
| contig_2285 | 818 | 1066 | + | Carboxylesterase (EC 3.1.1.1) |
| contig_2285 | 1225 | 1428 | + | Carboxylesterase (EC 3.1.1.1) |
| contig_2285 | 1771 | 1484 | - | sporulation kinase A |
| contig_2285 | 2216 | 1776 | - | sporulation kinase A |
| contig_2285 | 2607 | 2242 | - | sporulation kinase A |
| contig_2285 | 2923 | 2594 | - | sporulation kinase A |
| contig_2287 | 78 | 365 | + | Outer surface protein of unknown function, cellobiose operon |
| contig_2287 | 322 | 621 | + | Outer surface protein of unknown function, cellobiose operon |
| contig_2287 | 781 | 1185 | + | putative membrane protein |
| contig_2288 | 229 | 101 | - | Dihydrolipoamide acetyltransferase component (E2) of acetoin dehydrogenase complex (EC 2.3.1.-) |
| contig_2288 | 545 | 207 | - | Dihydrolipoamide acetyltransferase component (E2) of acetoin dehydrogenase complex (EC 2.3.1.-) |
| contig_2288 | 886 | 626 | - | Dihydrolipoamide acetyltransferase component (E2) of acetoin dehydrogenase complex (EC 2.3.1.-) |
| contig_2289 | 155 | 6 | - | transcriptional regulator, ArsR family |
| contig_2289 | 496 | 326 | - | transcriptional regulator, ArsR family |
| contig_229 | 338 | 3 | - | ABC transporter, permease protein |
| contig_229 | 752 | 600 | - | ABC transporter, permease protein |
| contig_229 | 2043 | 1039 | - | ABC transporter, permease protein |
| contig_229 | 2239 | 2111 | - | ABC transporter, permease protein |
| contig_229 | 2624 | 2379 | - | ABC transporter, permease protein, putative |
| contig_229 | 3305 | 3048 | - | ABC transporter, permease protein, putative |
| contig_229 | 3286 | 3453 | + | FIG01233509: hypothetical protein |
| contig_2290 | 274 | 101 | - | Glycerophosphoryl diester phosphodiesterase, periplasmic (EC 3.1.4.46) |
| contig_2290 | 455 | 267 | - | Glycerophosphoryl diester phosphodiesterase, periplasmic (EC 3.1.4.46) |
| contig_2290 | 688 | 575 | - | acetyltransferase, GNAT family |
| contig_2290 | 835 | 692 | - | acetyltransferase, GNAT family |
| contig_2290 | 947 | 792 | - | acetyltransferase, GNAT family |
| contig_2290 | 1308 | 973 | - | acetyltransferase, GNAT family |
| contig_2292 | 247 | 17 | - | ABC transporter, ATP-binding protein, putative |
| contig_2292 | 756 | 544 | - | ABC transporter, ATP-binding protein, putative |
| contig_2293 | 313 | 176 | - | Bona fide RidA/YjgF/TdcF/RutC subgroup |
| contig_2293 | 551 | 306 | - | Bona fide RidA/YjgF/TdcF/RutC subgroup |
| contig_2295 | 205 | 86 | - | Similar to eukaryotic Peptidyl prolyl 4-hydroxylase, alpha subunit (EC 1.14.11.2) |
| contig_2297 | 93 | 215 | + | Foldase protein PrsA precursor (EC 5.2.1.8) |
| contig_2297 | 202 | 378 | + | Foldase protein PrsA precursor (EC 5.2.1.8) |
| contig_2298 | 384 | 226 | - | putative phosphohydrolases, Icc family |
| contig_2298 | 772 | 341 | - | putative phosphohydrolases, Icc family |
| contig_2299 | 408 | 121 | - | wall-associated protein, putative |
| contig_23 | 161 | 30 | - | Aconitate hydratase (EC 4.2.1.3) @ 2-methylisocitrate dehydratase (EC 4.2.1.99) |
| contig_23 | 1497 | 139 | - | Aconitate hydratase (EC 4.2.1.3) @ 2-methylisocitrate dehydratase (EC 4.2.1.99) |
| contig_23 | 2296 | 1520 | - | Aconitate hydratase (EC 4.2.1.3) @ 2-methylisocitrate dehydratase (EC 4.2.1.99) |
| contig_23 | 2660 | 2250 | - | Aconitate hydratase (EC 4.2.1.3) @ 2-methylisocitrate dehydratase (EC 4.2.1.99) |
| contig_230 | 303 | 803 | + | hypothetical protein |
| contig_230 | 1311 | 763 | - | 1-deoxy-D-xylulose 5-phosphate reductoisomerase (EC 1.1.1.267) |
| contig_230 | 1871 | 1497 | - | 1-deoxy-D-xylulose 5-phosphate reductoisomerase (EC 1.1.1.267) |
| contig_230 | 2116 | 1895 | - | Phosphatidate cytidylyltransferase (EC 2.7.7.41) |
| contig_230 | 2280 | 2158 | - | Phosphatidate cytidylyltransferase (EC 2.7.7.41) |
| contig_230 | 2686 | 2246 | - | Phosphatidate cytidylyltransferase (EC 2.7.7.41) |
| contig_230 | 3480 | 2704 | - | Undecaprenyl diphosphate synthase (EC 2.5.1.31) |
| contig_230 | 4036 | 3566 | - | Ribosome recycling factor |
| contig_230 | 4127 | 4011 | - | Ribosome recycling factor |
| contig_230 | 4657 | 4130 | - | Uridine monophosphate kinase (EC 2.7.4.22) |
| contig_230 | 4853 | 4659 | - | Uridine monophosphate kinase (EC 2.7.4.22) |
| contig_230 | 5088 | 4954 | - | Translation elongation factor Ts |
| contig_230 | 5449 | 5060 | - | Translation elongation factor Ts |
| contig_230 | 5811 | 5446 | - | Translation elongation factor Ts |
| contig_230 | 6331 | 5915 | - | SSU ribosomal protein S2p (SAe) |
| contig_230 | 6615 | 6325 | - | SSU ribosomal protein S2p (SAe) |
| contig_230 | 7290 | 6964 | - | GTP-sensing transcriptional pleiotropic repressor codY |
| contig_230 | 7654 | 7436 | - | GTP-sensing transcriptional pleiotropic repressor codY |
| contig_230 | 8915 | 7809 | - | ATP-dependent hsl protease ATP-binding subunit HslU |
| contig_230 | 9063 | 8893 | - | ATP-dependent hsl protease ATP-binding subunit HslU |
| contig_230 | 9797 | 9255 | - | ATP-dependent protease HslV (EC 3.4.25.-) |
| contig_230 | 10478 | 9840 | - | Site-specific tyrosine recombinase |
| contig_230 | 10743 | 10534 | - | Site-specific tyrosine recombinase |
| contig_230 | 11234 | 10884 | - | tRNA:m(5)U-54 MTase gid |
| contig_230 | 12018 | 11311 | - | tRNA:m(5)U-54 MTase gid |
| contig_230 | 12118 | 11978 | - | tRNA:m(5)U-54 MTase gid |
| contig_230 | 12330 | 12193 | - | DNA topoisomerase I (EC 5.99.1.2) |
| contig_230 | 13004 | 12384 | - | DNA topoisomerase I (EC 5.99.1.2) |
| contig_230 | 13662 | 12982 | - | DNA topoisomerase I (EC 5.99.1.2) |
| contig_230 | 13867 | 13718 | - | DNA topoisomerase I (EC 5.99.1.2) |
| contig_230 | 14261 | 13947 | - | DNA topoisomerase I (EC 5.99.1.2) |
| contig_230 | 14663 | 14406 | - | Rossmann fold nucleotide-binding protein Smf possibly involved in DNA uptake |
| contig_230 | 15276 | 14617 | - | Rossmann fold nucleotide-binding protein Smf possibly involved in DNA uptake |
| contig_230 | 15651 | 15364 | - | Succinyl-CoA ligase [ADP-forming] alpha chain (EC 6.2.1.5) |
| contig_2300 | 127 | 465 | + | Group-specific protein |
| contig_2301 | 647 | 486 | - | FIG01225755: hypothetical protein |
| contig_2303 | 396 | 235 | - | UDP-glucose 4-epimerase (EC 5.1.3.2) |
| contig_2303 | 703 | 371 | - | UDP-glucose 4-epimerase (EC 5.1.3.2) |
| contig_2303 | 941 | 687 | - | UDP-glucose 4-epimerase (EC 5.1.3.2) |
| contig_2303 | 1072 | 935 | - | UDP-glucose 4-epimerase-like protein |
| contig_2303 | 1253 | 1140 | - | HMP-PP hydrolase (pyridoxal phosphatase) Cof, detected in genetic screen for thiamin metabolic genes (PMID:15292217) |
| contig_2304 | 468 | 247 | - | hypothetical protein |
| contig_2307 | 949 | 536 | - | Phosphonate ABC transporter phosphate-binding periplasmic component (TC 3.A.1.9.1) |
| contig_2308 | 211 | 14 | - | reticulocyte binding protein |
| contig_231 | 398 | 225 | - | hypothetical protein |
| contig_231 | 682 | 545 | - | hypothetical protein |
| contig_2310 | 688 | 254 | - | Uncharacterised protein family UPF0157 (COG2320) |
| contig_2310 | 842 | 639 | - | Uncharacterised protein family UPF0157 (COG2320) |
| contig_2313 | 191 | 42 | - | Transcriptional regulator, PadR family |
| contig_2315 | 153 | 317 | + | Adenosylmethionine-8-amino-7-oxononanoate aminotransferase (EC 2.6.1.62) |
| contig_2315 | 289 | 435 | + | Adenosylmethionine-8-amino-7-oxononanoate aminotransferase (EC 2.6.1.62) |
| contig_2317 | 269 | 129 | - | Phosphonate ABC transporter phosphate-binding periplasmic component (TC 3.A.1.9.1) |
| contig_2317 | 546 | 418 | - | Phosphonate ABC transporter phosphate-binding periplasmic component (TC 3.A.1.9.1) |
| contig_2319 | 107 | 406 | + | sensory box/GGDEF family protein |
| contig_2319 | 721 | 1131 | + | sensory box/GGDEF family protein |
| contig_2319 | 1178 | 1417 | + | sensory box/GGDEF family protein |
| contig_2319 | 1537 | 2193 | + | sensory box/GGDEF family protein |
| contig_232 | 133 | 588 | + | Glyoxalase family protein |
| contig_2323 | 206 | 21 | - | Deblocking aminopeptidase (EC 3.4.11.-) |
| contig_2324 | 893 | 567 | - | FIG01228166: hypothetical protein |
| contig_2324 | 1084 | 911 | - | FIG01227716: hypothetical protein |
| contig_2325 | 285 | 536 | + | Predicted transcriptional regulator of pyridoxine metabolism |
| contig_2327 | 282 | 94 | - | Phosphoglycerate mutase family 2 |
| contig_2327 | 660 | 439 | - | FIG01226501: hypothetical protein |
| contig_2327 | 866 | 714 | - | FIG01226501: hypothetical protein |
| contig_2327 | 1083 | 925 | - | FIG01226501: hypothetical protein |
| contig_2327 | 1345 | 1145 | - | conserved domain protein |
| contig_2328 | 143 | 613 | + | Uncharacterized protein conserved in bacteria |
| contig_2328 | 663 | 791 | + | Uncharacterized protein conserved in bacteria |
| contig_2328 | 1133 | 855 | - | Prephenate dehydratase (EC 4.2.1.51) |
| contig_233 | 169 | 609 | + | CAAX amino terminal protease family protein |
| contig_233 | 593 | 739 | + | CAAX amino terminal protease family protein |
| contig_233 | 1047 | 877 | - | RNA polymerase sigma factor SigZ |
| contig_233 | 1192 | 1019 | - | hypothetical protein |
| contig_2330 | 426 | 175 | - | O-acetyl transferase (EC 2.3.1.-) |
| contig_2330 | 737 | 594 | - | O-acetyl transferase (EC 2.3.1.-) |
| contig_2334 | 211 | 20 | - | Pyruvate decarboxylase (EC 4.1.1.1); Indole-3-pyruvate decarboxylase (EC 4.1.1.74) |
| contig_2334 | 1219 | 347 | - | Pyruvate decarboxylase (EC 4.1.1.1); Alpha-keto-acid decarboxylase (EC 4.1.1.-) |
| contig_2334 | 1671 | 1312 | - | Pyruvate decarboxylase (EC 4.1.1.1); Alpha-keto-acid decarboxylase (EC 4.1.1.-) |
| contig_2335 | 630 | 508 | - | Cell surface protein IsdA, transfers heme from hemoglobin to apo-IsdC |
| contig_2336 | 338 | 186 | - | Phage T7 exclusion protein |
| contig_2337 | 580 | 434 | - | Transcriptional regulator, AraC family |
| contig_2338 | 138 | 260 | + | Glycine betaine ABC transport system, glycine betaine-binding protein OpuAC |
| contig_2338 | 886 | 305 | - | ATP-dependent Clp protease proteolytic subunit (EC 3.4.21.92) |
| contig_2339 | 364 | 29 | - | Alpha-1,3-N-acetylgalactosamine transferase PglA (EC 2.4.1.-) |
| contig_2339 | 609 | 361 | - | Alpha-1,3-N-acetylgalactosamine transferase PglA (EC 2.4.1.-) |
| contig_2339 | 1042 | 677 | - | Alpha-1,3-N-acetylgalactosamine transferase PglA (EC 2.4.1.-) |
| contig_2339 | 1130 | 1017 | - | Alpha-1,3-N-acetylgalactosamine transferase PglA (EC 2.4.1.-) |
| contig_234 | 217 | 83 | - | rRNA small subunit methyltransferase I |
| contig_234 | 446 | 186 | - | COG2827: putative endonuclease containing a URI domain |
| contig_234 | 801 | 463 | - | tRNA (adenine37-N(6))-methyltransferase TrmN6 (EC 2.1.1.223) |
| contig_234 | 1202 | 798 | - | tRNA (adenine37-N(6))-methyltransferase TrmN6 (EC 2.1.1.223) |
| contig_234 | 1520 | 1323 | - | DNA replication intiation control protein YabA |
| contig_234 | 1675 | 1517 | - | DNA replication intiation control protein YabA |
| contig_234 | 2517 | 1690 | - | Signal peptidase-like protein / Stage 0 sporulation protein YaaT |
| contig_234 | 2807 | 2523 | - | DNA polymerase III delta prime subunit (EC 2.7.7.7) |
| contig_234 | 3506 | 2820 | - | DNA polymerase III delta prime subunit (EC 2.7.7.7) |
| contig_234 | 3667 | 3542 | - | Thymidylate kinase (EC 2.7.4.9) |
| contig_234 | 4169 | 3660 | - | Thymidylate kinase (EC 2.7.4.9) |
| contig_234 | 5166 | 4171 | - | Arginine decarboxylase (EC 4.1.1.19) / Lysine decarboxylase (EC 4.1.1.18) |
| contig_234 | 5591 | 5199 | - | Arginine decarboxylase (EC 4.1.1.19) / Lysine decarboxylase (EC 4.1.1.18) |
| contig_234 | 5839 | 5660 | - | CsfB protein |
| contig_234 | 6149 | 6034 | - | 5S RNA |
| contig_2341 | 143 | 415 | + | hypothetical protein |
| contig_2342 | 26 | 154 | + | hypothetical protein |
| contig_2344 | 190 | 59 | - | acetyltransferase, GNAT family family |
| contig_2346 | 276 | 58 | - | UDP-N-acetylmuramoylalanyl-D-glutamate--2,6-diaminopimelate ligase (EC 6.3.2.13) |
| contig_2346 | 452 | 276 | - | UDP-N-acetylmuramoylalanyl-D-glutamate--2,6-diaminopimelate ligase (EC 6.3.2.13) |
| contig_2346 | 595 | 434 | - | UDP-N-acetylmuramoylalanyl-D-glutamate--2,6-diaminopimelate ligase (EC 6.3.2.13) |
| contig_2347 | 111 | 224 | + | hypothetical protein |
| contig_2348 | 200 | 75 | - | hypothetical protein |
| contig_2349 | 146 | 271 | + | hypothetical protein |
| contig_2349 | 276 | 737 | + | Dihydrolipoamide dehydrogenase of acetoin dehydrogenase (EC 1.8.1.4) |
| contig_235 | 243 | 419 | + | Short chain fatty acids transporter |
| contig_235 | 1051 | 1488 | + | Short chain fatty acids transporter |
| contig_235 | 1784 | 1671 | - | FIG01226976: hypothetical protein |
| contig_235 | 2388 | 1858 | - | FIG01226976: hypothetical protein |
| contig_235 | 2539 | 2366 | - | putative oxidoreductase |
| contig_235 | 2802 | 2563 | - | FIG01225533: hypothetical protein |
| contig_235 | 2957 | 2799 | - | FIG01225533: hypothetical protein |
| contig_235 | 3416 | 3207 | - | FIG01229430: hypothetical protein |
| contig_2357 | 24 | 644 | + | FIG01225135: hypothetical protein |
| contig_2358 | 100 | 480 | + | Mg(2+) transport ATPase protein C |
| contig_2359 | 97 | 390 | + | FIG01227072: hypothetical protein |
| contig_2359 | 511 | 684 | + | hypothetical protein |
| contig_236 | 348 | 43 | - | FIG01225112: hypothetical protein |
| contig_236 | 1502 | 342 | - | FIG01225112: hypothetical protein |
| contig_236 | 1968 | 1561 | - | FIG01225112: hypothetical protein |
| contig_236 | 2180 | 2986 | + | Transcriptional regulator, LysR family |
| contig_236 | 4199 | 3114 | - | FIG01226007: hypothetical protein |
| contig_2360 | 209 | 658 | + | FIG01227114: hypothetical protein |
| contig_2361 | 176 | 319 | + | acetyltransferase, GNAT family |
| contig_2361 | 459 | 686 | + | D-glycero-D-manno-heptose 1,7-bisphosphate phosphatase (EC 3.1.1.-); possible Histidinol-phosphatase (EC 3.1.3.15) |
| contig_2361 | 751 | 987 | + | D-glycero-D-manno-heptose 1,7-bisphosphate phosphatase (EC 3.1.1.-); possible Histidinol-phosphatase (EC 3.1.3.15) |
| contig_2362 | 182 | 310 | + | Thiol:disulfide oxidoreductase related to ResA |
| contig_2362 | 330 | 479 | + | Thiol:disulfide oxidoreductase related to ResA |
| contig_2362 | 498 | 695 | + | prolipoprotein diacylglyceryl transferase family protein |
| contig_2363 | 533 | 309 | - | Permease of the drug/metabolite transporter (DMT) superfamily |
| contig_2363 | 637 | 497 | - | Permease of the drug/metabolite transporter (DMT) superfamily |
| contig_2363 | 920 | 1327 | + | Transcriptional regulator, GntR family domain / Aspartate aminotransferase (EC 2.6.1.1) |
| contig_2363 | 1320 | 1976 | + | Transcriptional regulator, GntR family domain / Aspartate aminotransferase (EC 2.6.1.1) |
| contig_2366 | 84 | 524 | + | Glutamate synthase [NADPH] large chain (EC 1.4.1.13) |
| contig_2366 | 586 | 717 | + | Glutamate synthase [NADPH] large chain (EC 1.4.1.13) |
| contig_2366 | 1058 | 909 | - | HigA protein (antitoxin to HigB) |
| contig_2366 | 1229 | 1092 | - | FIG01225310: hypothetical protein |
| contig_2367 | 560 | 423 | - | Alpha-1,3-N-acetylgalactosamine transferase PglA (EC 2.4.1.-) |
| contig_2368 | 569 | 267 | - | FIG01225455: hypothetical protein |
| contig_2369 | 324 | 163 | - | Oligopeptide ABC transporter, periplasmic oligopeptide-binding protein OppA (TC 3.A.1.5.1) |
| contig_2369 | 770 | 363 | - | Oligopeptide ABC transporter, periplasmic oligopeptide-binding protein OppA (TC 3.A.1.5.1) |
| contig_2369 | 1098 | 826 | - | Oligopeptide ABC transporter, periplasmic oligopeptide-binding protein OppA (TC 3.A.1.5.1) |
| contig_237 | 469 | 266 | - | Glutamine transport ATP-binding protein GlnQ (TC 3.A.1.3.2) |
| contig_237 | 745 | 602 | - | ABC transporter membrane-spanning permease - glutamine transport |
| contig_237 | 1262 | 708 | - | ABC transporter membrane-spanning permease - glutamine transport |
| contig_237 | 1472 | 1299 | - | Amino acid ABC transporter, amino acid-binding protein |
| contig_237 | 2076 | 1735 | - | Amino acid ABC transporter, amino acid-binding protein |
| contig_2370 | 774 | 649 | - | Nitrate/nitrite sensor protein (EC 2.7.3.-) |
| contig_2370 | 877 | 740 | - | Nitrate/nitrite sensor protein (EC 2.7.3.-) |
| contig_2371 | 486 | 34 | - | FIG01231976: hypothetical protein |
| contig_2372 | 214 | 32 | - | HAMP domain protein |
| contig_2373 | 747 | 220 | - | conserved domain protein |
| contig_2376 | 721 | 68 | - | tRNA (guanosine(18)-2'-O)-methyltransferase (EC 2.1.1.34) |
| contig_2378 | 667 | 278 | - | FIG01225847: hypothetical protein |
| contig_238 | 230 | 352 | + | hypothetical protein |
| contig_238 | 1474 | 416 | - | hypothetical protein |
| contig_238 | 1695 | 1468 | - | hypothetical protein |
| contig_238 | 2287 | 1781 | - | hypothetical protein |
| contig_238 | 2767 | 2549 | - | hypothetical protein |
| contig_2382 | 381 | 217 | - | LysR family regulatory protein CidR |
| contig_2383 | 1406 | 504 | - | 5-methylthioribose kinase (EC 2.7.1.100) |
| contig_2384 | 203 | 12 | - | methyl-accepting chemotaxis protein |
| contig_2385 | 401 | 111 | - | ABC transporter, ATP-binding protein |
| contig_2385 | 550 | 404 | - | ABC transporter, ATP-binding protein |
| contig_2387 | 1098 | 142 | - | Cell division protein FtsZ (EC 3.4.24.-) |
| contig_2388 | 342 | 175 | - | Methylthioribose-1-phosphate isomerase (EC 5.3.1.23) |
| contig_2389 | 310 | 161 | - | hypothetical protein |
| contig_2389 | 487 | 729 | + | Exosporium protein F |
| contig_239 | 652 | 404 | - | Catabolite repression HPr-like protein Crh |
| contig_239 | 1626 | 676 | - | FIG001886: Cytoplasmic hypothetical protein |
| contig_239 | 1838 | 1716 | - | FIG002813: LPPG:FO 2-phospho-L-lactate transferase like, CofD-like |
| contig_239 | 1963 | 1835 | - | FIG002813: LPPG:FO 2-phospho-L-lactate transferase like, CofD-like |
| contig_239 | 2672 | 1968 | - | FIG002813: LPPG:FO 2-phospho-L-lactate transferase like, CofD-like |
| contig_239 | 3557 | 2676 | - | Hypothetical ATP-binding protein UPF0042, contains P-loop |
| contig_239 | 3736 | 3578 | - | Mutator mutT protein (7,8-dihydro-8-oxoguanine-triphosphatase) (EC 3.6.1.-) |
| contig_239 | 4038 | 3823 | - | Mutator mutT protein (7,8-dihydro-8-oxoguanine-triphosphatase) (EC 3.6.1.-) |
| contig_239 | 5075 | 4269 | - | Integral membrane protein |
| contig_2390 | 300 | 434 | + | cysteine-rich protein |
| contig_2391 | 239 | 15 | - | FIG01249387: hypothetical protein |
| contig_2393 | 745 | 128 | - | Multidrug resistance ABC transporter ATP-binding and permease protein |
| contig_2394 | 631 | 203 | - | acetyltransferase, GNAT family |
| contig_2394 | 756 | 628 | - | FIG01228499: hypothetical protein |
| contig_2394 | 889 | 728 | - | FIG01228499: hypothetical protein |
| contig_2395 | 478 | 287 | - | sensor histidine kinase |
| contig_2395 | 847 | 635 | - | Purine nucleoside phosphorylase (EC 2.4.2.1) |
| contig_2396 | 370 | 483 | + | hypothetical protein |
| contig_2396 | 691 | 464 | - | Transcriptional regulator, MerR family |
| contig_2397 | 156 | 311 | + | FIG01229470: hypothetical protein |
| contig_2398 | 566 | 276 | - | FIG01226668: hypothetical protein |
| contig_2399 | 20 | 280 | + | membrane protein, putative |
| contig_24 | 502 | 26 | - | Fibronectin/fibrinogen-binding protein |
| contig_24 | 484 | 609 | + | hypothetical protein |
| contig_24 | 1374 | 853 | - | Fibronectin/fibrinogen-binding protein |
| contig_24 | 1747 | 1454 | - | Fibronectin/fibrinogen-binding protein |
| contig_24 | 2139 | 1909 | - | transposase, IS605 OrfB family |
| contig_24 | 2411 | 2175 | - | transposase, IS605 OrfB family |
| contig_24 | 2607 | 2380 | - | transposase, IS605 OrfB family |
| contig_24 | 2896 | 2564 | - | transposase, IS605 OrfB family |
| contig_240 | 572 | 120 | - | Probable membrane protein YetF |
| contig_240 | 2407 | 914 | - | Cardiolipin synthetase (EC 2.7.8.-) |
| contig_240 | 3097 | 2789 | - | Conserved protein |
| contig_2400 | 209 | 18 | - | Hypothetical protein perhaps functionally coupled to transcription elongation factor GreA |
| contig_2401 | 825 | 706 | - | response regulator, putative |
| contig_2404 | 205 | 32 | - | ECF-type sigma factor negative effector |
| contig_2405 | 470 | 258 | - | Membrane-attached cytochrome c550 |
| contig_2405 | 637 | 512 | - | hypothetical protein |
| contig_2405 | 998 | 837 | - | FIG01244734: hypothetical protein |
| contig_2408 | 816 | 58 | - | Triacylglycerol lipase (EC 3.1.1.3) |
| contig_2408 | 1105 | 830 | - | Triacylglycerol lipase (EC 3.1.1.3) |
| contig_2409 | 1096 | 155 | - | Glucokinase (EC 2.7.1.2) |
| contig_2409 | 1448 | 1116 | - | Tagatose-6-phosphate kinase (EC 2.7.1.144) / 1-phosphofructokinase (EC 2.7.1.56) |
| contig_241 | 131 | 349 | + | AA3-600 quinol oxidase subunit II |
| contig_241 | 318 | 1007 | + | AA3-600 quinol oxidase subunit II |
| contig_241 | 1041 | 2975 | + | AA3-600 quinol oxidase subunit I |
| contig_241 | 2989 | 3174 | + | AA3-600 quinol oxidase subunit IIII |
| contig_241 | 3164 | 3595 | + | AA3-600 quinol oxidase subunit IIII |
| contig_241 | 3760 | 3909 | + | AA3-600 quinol oxidase subunit IV |
| contig_2410 | 437 | 228 | - | FIG01226010: hypothetical protein |
| contig_2412 | 145 | 372 | + | FIG01225504: hypothetical protein |
| contig_2412 | 818 | 414 | - | Putative methyltransferase YodH |
| contig_2413 | 315 | 109 | - | FIG01227048: hypothetical protein |
| contig_2413 | 438 | 560 | + | Small, acid-soluble spore protein P |
| contig_2413 | 685 | 954 | + | Spore coat protein M |
| contig_2413 | 926 | 1066 | + | Spore coat protein M |
| contig_2415 | 231 | 344 | + | 2-amino-3-carboxymuconate-6-semialdehyde decarboxylase (EC 4.1.1.45) |
| contig_2415 | 337 | 465 | + | 2-amino-3-carboxymuconate-6-semialdehyde decarboxylase (EC 4.1.1.45) |
| contig_2415 | 443 | 682 | + | Bona fide RidA/YjgF/TdcF/RutC subgroup |
| contig_2415 | 697 | 900 | + | Bona fide RidA/YjgF/TdcF/RutC subgroup |
| contig_2415 | 953 | 1066 | + | hypothetical protein |
| contig_2416 | 363 | 19 | - | Allophanate hydrolase 2 subunit 2 (EC 3.5.1.54) |
| contig_2416 | 595 | 428 | - | Allophanate hydrolase 2 subunit 2 (EC 3.5.1.54) |
| contig_2416 | 894 | 586 | - | Allophanate hydrolase 2 subunit 1 (EC 3.5.1.54) |
| contig_2416 | 1533 | 1315 | - | Transcriptional regulator, IclR family |
| contig_242 | 234 | 58 | - | trifolitoxin immunity domain protein |
| contig_242 | 683 | 216 | - | trifolitoxin immunity domain protein |
| contig_242 | 855 | 649 | - | trifolitoxin immunity domain protein |
| contig_242 | 1120 | 875 | - | FIG01225146: hypothetical protein |
| contig_242 | 1370 | 1146 | - | FIG01225146: hypothetical protein |
| contig_2421 | 468 | 202 | - | FIG01226310: hypothetical protein |
| contig_2422 | 474 | 310 | - | 8-amino-7-oxononanoate synthase (EC 2.3.1.47) |
| contig_2424 | 125 | 640 | + | membrane protein, putative |
| contig_2424 | 871 | 680 | - | COG1649 predicted glycoside hydrolase |
| contig_2424 | 1418 | 858 | - | COG1649 predicted glycoside hydrolase |
| contig_2424 | 1602 | 1324 | - | COG1649 predicted glycoside hydrolase |
| contig_2425 | 1079 | 735 | - | DNA-binding response regulator |
| contig_2427 | 168 | 52 | - | hypothetical protein |
| contig_2427 | 305 | 814 | + | sensor histidine kinase |
| contig_2428 | 395 | 156 | - | dTDP-4-dehydrorhamnose reductase (EC 1.1.1.133) |
| contig_2428 | 688 | 407 | - | dTDP-glucose 4,6-dehydratase (EC 4.2.1.46) |
| contig_2428 | 1320 | 742 | - | dTDP-glucose 4,6-dehydratase (EC 4.2.1.46) |
| contig_2429 | 528 | 22 | - | NPQTN specific sortase B |
| contig_243 | 366 | 1127 | + | ComF operon protein A, DNA transporter ATPase |
| contig_243 | 1084 | 1353 | + | ComF operon protein A, DNA transporter ATPase |
| contig_243 | 1418 | 1717 | + | ComF operon protein A, DNA transporter ATPase |
| contig_243 | 1717 | 2421 | + | Competence protein F homolog, phosphoribosyltransferase domain; protein YhgH required for utilization of DNA as sole source of carbon and energy |
| contig_243 | 2542 | 2745 | + | Cold shock protein CspA |
| contig_2430 | 220 | 765 | + | Glycosyl transferase ,group 2 family, anthrose biosynthesis |
| contig_2430 | 762 | 881 | + | Glycosyl transferase ,group 2 family, anthrose biosynthesis |
| contig_2431 | 283 | 35 | - | Amidohydrolase AmhX |
| contig_2432 | 58 | 204 | + | Oligopeptide transport system permease protein OppB (TC 3.A.1.5.1) |
| contig_2432 | 259 | 510 | + | Oligopeptide transport system permease protein OppB (TC 3.A.1.5.1) |
| contig_2432 | 633 | 899 | + | Oligopeptide transport system permease protein OppC (TC 3.A.1.5.1) |
| contig_2432 | 890 | 1213 | + | Oligopeptide transport system permease protein OppC (TC 3.A.1.5.1) |
| contig_2432 | 1215 | 1514 | + | Oligopeptide transport system permease protein OppC (TC 3.A.1.5.1) |
| contig_2432 | 1797 | 1558 | - | Pyruvate oxidase [ubiquinone, cytochrome] (EC 1.2.2.2) |
| contig_2433 | 323 | 63 | - | FIG01225731: hypothetical protein |
| contig_2435 | 256 | 131 | - | Foldase protein PrsA precursor (EC 5.2.1.8) |
| contig_2436 | 555 | 627 | + | tRNA-Val-GAC |
| contig_2437 | 36 | 182 | + | Predicted transcriptional regulator of pyridoxine metabolism |
| contig_2437 | 176 | 301 | + | Predicted transcriptional regulator of pyridoxine metabolism |
| contig_2437 | 298 | 1158 | + | Predicted transcriptional regulator of pyridoxine metabolism |
| contig_2437 | 2188 | 1343 | - | membrane protein, putative |
| contig_2438 | 247 | 56 | - | Urease accessory protein UreG |
| contig_2438 | 513 | 262 | - | Urease accessory protein UreF |
| contig_2439 | 11 | 199 | + | D-alanyl-D-alanine carboxypeptidase (EC 3.4.16.4) |
| contig_244 | 23 | 163 | + | N-acetyl-gamma-glutamyl-phosphate reductase (EC 1.2.1.38) |
| contig_244 | 268 | 915 | + | N-acetyl-gamma-glutamyl-phosphate reductase (EC 1.2.1.38) |
| contig_2440 | 431 | 769 | + | membrane protein, putative |
| contig_2443 | 140 | 6 | - | Mobile element protein |
| contig_2445 | 193 | 5 | - | Phosphoribosylformylglycinamidine synthase, PurS subunit (EC 6.3.5.3) |
| contig_2447 | 88 | 213 | + | Na+ driven multidrug efflux pump |
| contig_2447 | 185 | 373 | + | Na+ driven multidrug efflux pump |
| contig_2447 | 1132 | 434 | - | Peptidase E (EC 3.4.11.2) |
| contig_2448 | 276 | 467 | + | Sodium/glycine symporter GlyP |
| contig_2449 | 184 | 411 | + | transcriptional regulator, putative |
| contig_2449 | 724 | 536 | - | FIG01225282: hypothetical protein |
| contig_245 | 276 | 998 | + | Non-heme chloroperoxidase (EC 1.11.1.10) |
| contig_2451 | 419 | 261 | - | Xylose-responsive transcription regulator, ROK family |
| contig_2451 | 609 | 412 | - | Xylose-responsive transcription regulator, ROK family |
| contig_2451 | 1060 | 1416 | + | Xylose isomerase (EC 5.3.1.5) |
| contig_2451 | 1413 | 1772 | + | Xylose isomerase (EC 5.3.1.5) |
| contig_2452 | 59 | 445 | + | membrane protein, putative |
| contig_2453 | 140 | 493 | + | [hypothetical fig\|282458.1.peg.581 homolog](http://rast.nmpdr.org/seedviewer.cgi?page=Annotation&feature=hypothetical%20fig\|282458.1.peg.581%20homolog) |
| contig_2456 | 170 | 42 | - | wall-associated protein, putative |
| contig_2456 | 465 | 142 | - | wall-associated protein, putative |
| contig_2457 | 498 | 67 | - | Ser/Thr protein phosphatase family protein |
| contig_2457 | 717 | 586 | - | hypothetical protein |
| contig_2457 | 938 | 810 | - | hypothetical protein |
| contig_2458 | 236 | 469 | + | membrane protein, putative |
| contig_2459 | 45 | 248 | + | Sporulation kinase B homolog 1 |
| contig_2459 | 352 | 471 | + | Sporulation kinase B homolog 1 |
| contig_246 | 586 | 374 | - | SSU ribosomal protein S1p |
| contig_246 | 825 | 682 | - | SSU ribosomal protein S1p |
| contig_2460 | 120 | 278 | + | diglucosyldiacylglycerol synthase (LTA membrane anchor synthesis) |
| contig_2460 | 275 | 613 | + | diglucosyldiacylglycerol synthase (LTA membrane anchor synthesis) |
| contig_2463 | 203 | 502 | + | Manganese-dependent inorganic pyrophosphatase (EC 3.6.1.1) |
| contig_2464 | 276 | 452 | + | Branched-chain amino acid transport system carrier protein |
| contig_2467 | 648 | 337 | - | Transcriptional regulator, TetR family |
| contig_2467 | 850 | 665 | - | Transcriptional regulator, TetR family |
| contig_2468 | 525 | 145 | - | 4-hydroxy-2-oxovalerate aldolase (EC 4.1.3.39) |
| contig_247 | 283 | 68 | - | FIG013761: LmbE family protein |
| contig_247 | 464 | 261 | - | FIG013761: LmbE family protein |
| contig_247 | 582 | 451 | - | FIG013761: LmbE family protein |
| contig_247 | 738 | 601 | - | FIG013761: LmbE family protein |
| contig_247 | 1104 | 754 | - | FIG011895: hypothetical protein |
| contig_2471 | 491 | 135 | - | D-serine dehydratase (EC 4.3.1.18) |
| contig_2472 | 21 | 233 | + | Tryptophan 2,3-dioxygenase (EC 1.13.11.11) |
| contig_2473 | 103 | 216 | + | hypothetical protein |
| contig_2474 | 491 | 63 | - | Phosphoglycerate mutase (EC 5.4.2.1) |
| contig_2475 | 454 | 149 | - | FIG01226135: hypothetical protein |
| contig_2478 | 20 | 280 | + | FIG01228469: hypothetical protein |
| contig_2478 | 1342 | 635 | - | NAD-dependent protein deacetylase of SIR2 family |
| contig_2479 | 406 | 278 | - | hypothetical protein |
| contig_2479 | 603 | 409 | - | Ribonuclease E inhibitor RraA |
| contig_248 | 1332 | 631 | - | Quinolinate synthetase (EC 2.5.1.72) |
| contig_248 | 1738 | 1292 | - | Quinolinate synthetase (EC 2.5.1.72) |
| contig_248 | 2269 | 1769 | - | Quinolinate phosphoribosyltransferase [decarboxylating] (EC 2.4.2.19) |
| contig_248 | 2603 | 2238 | - | Quinolinate phosphoribosyltransferase [decarboxylating] (EC 2.4.2.19) |
| contig_248 | 3162 | 2623 | - | L-aspartate oxidase (EC 1.4.3.16) |
| contig_248 | 3637 | 3137 | - | L-aspartate oxidase (EC 1.4.3.16) |
| contig_248 | 4130 | 3648 | - | L-aspartate oxidase (EC 1.4.3.16) |
| contig_2482 | 404 | 216 | - | Transcriptional regulator, XRE family |
| contig_2482 | 738 | 544 | - | Transcriptional regulator, XRE family |
| contig_2483 | 161 | 364 | + | 6-aminohexanoate-dimer hydrolase (EC 3.5.1.46) |
| contig_2483 | 620 | 1120 | + | 6-aminohexanoate-dimer hydrolase (EC 3.5.1.46) |
| contig_2484 | 145 | 312 | + | Urea channel UreI |
| contig_2485 | 708 | 82 | - | Glycerophosphoryl diester phosphodiesterase, periplasmic (EC 3.1.4.46) |
| contig_2486 | 146 | 286 | + | Lon-like protease with PDZ domain |
| contig_2489 | 179 | 15 | - | Glyoxalase family protein |
| contig_2489 | 456 | 205 | - | Glyoxalase family protein |
| contig_2489 | 794 | 531 | - | L-Cystine ABC transporter, periplasmic cystine-binding protein TcyA |
| contig_2489 | 1230 | 787 | - | L-Cystine ABC transporter, periplasmic cystine-binding protein TcyA |
| contig_2489 | 1684 | 1337 | - | amino acid ABC transporter, ATP-binding protein |
| contig_249 | 1594 | 158 | - | Protein-export membrane protein SecD (TC 3.A.5.1.1) / Protein-export membrane protein SecF (TC 3.A.5.1.1) |
| contig_249 | 1924 | 1646 | - | Protein-export membrane protein SecD (TC 3.A.5.1.1) / Protein-export membrane protein SecF (TC 3.A.5.1.1) |
| contig_249 | 2425 | 2234 | - | Protein-export membrane protein SecD (TC 3.A.5.1.1) / Protein-export membrane protein SecF (TC 3.A.5.1.1) |
| contig_249 | 2902 | 2561 | - | FIG01227478: hypothetical protein |
| contig_249 | 3156 | 3890 | + | Stage V sporulation protein B |
| contig_249 | 3931 | 4722 | + | Stage V sporulation protein B |
| contig_249 | 5400 | 4753 | - | Ortholog yrbG, yetE, ykjA, ydfS, ydfR B.subtilis |
| contig_249 | 5503 | 5886 | + | FIG01228422: hypothetical protein |
| contig_249 | 6078 | 5923 | - | Preprotein translocase subunit YajC (TC 3.A.5.1.1) |
| contig_249 | 6184 | 6068 | - | Preprotein translocase subunit YajC (TC 3.A.5.1.1) |
| contig_249 | 7351 | 6212 | - | tRNA-guanine transglycosylase (EC 2.4.2.29) |
| contig_249 | 7726 | 7364 | - | S-adenosylmethionine:tRNA ribosyltransferase-isomerase (EC 5.-.-.-) |
| contig_249 | 8417 | 7824 | - | S-adenosylmethionine:tRNA ribosyltransferase-isomerase (EC 5.-.-.-) |
| contig_2490 | 93 | 308 | + | Lipid A export ATP-binding/permease protein MsbA |
| contig_2492 | 338 | 117 | - | UPF0154 membrane protein YoxG |
| contig_2493 | 487 | 179 | - | IG hypothetical 18022 |
| contig_2494 | 299 | 475 | + | N-hydroxyarylamine O-acetyltransferase (EC 2.3.1.118) |
| contig_2494 | 444 | 671 | + | N-hydroxyarylamine O-acetyltransferase (EC 2.3.1.118) |
| contig_2495 | 617 | 99 | - | negative regulation of sporulation, septation and degradative enzyme genes (aprE, nprE, phoA, sacB) |
| contig_2496 | 85 | 255 | + | FIG01229422: hypothetical protein |
| contig_2496 | 398 | 571 | + | FIG01227321: hypothetical protein |
| contig_2497 | 471 | 611 | + | CAAX amino terminal protease family protein |
| contig_2498 | 532 | 14 | - | Tyrosine recombinase XerD |
| contig_2498 | 866 | 522 | - | Tyrosine recombinase XerD |
| contig_25 | 230 | 730 | + | Transcription-repair coupling factor |
| contig_25 | 797 | 1390 | + | Transcription-repair coupling factor |
| contig_25 | 1461 | 2624 | + | Transcription-repair coupling factor |
| contig_25 | 2761 | 3069 | + | Stage V sporulation protein T, AbrB family transcriptional regulator (SpoVT) |
| contig_25 | 3077 | 3298 | + | Stage V sporulation protein T, AbrB family transcriptional regulator (SpoVT) |
| contig_25 | 3529 | 4077 | + | Stage V sporulation protein B |
| contig_25 | 4104 | 4829 | + | Stage V sporulation protein B |
| contig_25 | 4879 | 5187 | + | Stage V sporulation protein B |
| contig_25 | 5147 | 6268 | + | possible tetrapyrrole methyltransferase domain / Nucleoside triphosphate pyrophosphohydrolase MazG (EC 3.6.1.8) |
| contig_25 | 6243 | 6608 | + | possible tetrapyrrole methyltransferase domain / Nucleoside triphosphate pyrophosphohydrolase MazG (EC 3.6.1.8) |
| contig_25 | 6623 | 6898 | + | Ribosome-associated heat shock protein implicated in the recycling of the 50S subunit (S4 paralog) |
| contig_25 | 6957 | 7265 | + | FIG007421: forespore shell protein |
| contig_25 | 7315 | 7797 | + | Spore cortex biosynthesis protein |
| contig_25 | 7915 | 8274 | + | Cell division protein DivIC (FtsB), stabilizes FtsL against RasP cleavage |
| contig_25 | 8430 | 8867 | + | RNA binding protein, contains ribosomal protein S1 domain |
| contig_25 | 9008 | 9081 | + | tRNA-Met-CAT |
| contig_25 | 9095 | 9166 | + | tRNA-Glu-TTC |
| contig_25 | 9451 | 9933 | + | Stage II sporulation serine phosphatase for sigma-F activation (SpoIIE) |
| contig_25 | 10067 | 9930 | - | hypothetical protein |
| contig_25 | 10114 | 11559 | + | Stage II sporulation serine phosphatase for sigma-F activation (SpoIIE) |
| contig_25 | 11540 | 11902 | + | Stage II sporulation serine phosphatase for sigma-F activation (SpoIIE) |
| contig_250 | 108 | 755 | + | ABC transporter, ATP-binding protein |
| contig_250 | 867 | 1211 | + | hypothetical protein |
| contig_250 | 1292 | 1432 | + | ABC transporter, ATP-binding protein |
| contig_250 | 1410 | 1553 | + | FIG01231247: hypothetical protein |
| contig_250 | 1911 | 2072 | + | FIG01231247: hypothetical protein |
| contig_250 | 2128 | 2445 | + | FIG01231247: hypothetical protein |
| contig_2503 | 246 | 58 | - | hypothetical protein |
| contig_2503 | 455 | 327 | - | hypothetical protein |
| contig_2504 | 252 | 545 | + | N-acetyltransferase family protein |
| contig_2507 | 350 | 87 | - | FIG01227733: hypothetical protein |
| contig_2508 | 43 | 255 | + | Sodium/glycine symporter GlyP |
| contig_2509 | 417 | 253 | - | FIG01236504: hypothetical protein |
| contig_251 | 508 | 41 | - | ABC transporter, permease |
| contig_251 | 627 | 514 | - | ABC transporter, permease |
| contig_251 | 1219 | 593 | - | ABC transporter, permease |
| contig_251 | 1896 | 1222 | - | ABC transporter ATP-binding protein YvcR |
| contig_251 | 2066 | 1893 | - | periplasmic component of efflux system |
| contig_251 | 2632 | 2063 | - | periplasmic component of efflux system |
| contig_251 | 2805 | 2632 | - | periplasmic component of efflux system |
| contig_251 | 3002 | 2862 | - | FIG01249387: hypothetical protein |
| contig_251 | 3144 | 2995 | - | FIG01233457: hypothetical protein |
| contig_251 | 3631 | 3125 | - | FIG01233457: hypothetical protein |
| contig_251 | 5219 | 3831 | - | FIG01233457: hypothetical protein |
| contig_2511 | 501 | 388 | - | hypothetical protein |
| contig_2511 | 805 | 530 | - | ThiJ/PfpI family protein |
| contig_2518 | 343 | 167 | - | FIG01228306: hypothetical protein |
| contig_252 | 146 | 274 | + | hypothetical protein |
| contig_252 | 699 | 355 | - | Glucokinase (EC 2.7.1.2) |
| contig_252 | 1339 | 722 | - | Glucokinase (EC 2.7.1.2) |
| contig_252 | 1559 | 1359 | - | FIG01225596: hypothetical protein |
| contig_252 | 2139 | 1663 | - | 5-formyltetrahydrofolate cyclo-ligase (EC 6.3.3.2) |
| contig_252 | 2242 | 2114 | - | 5-formyltetrahydrofolate cyclo-ligase (EC 6.3.3.2) |
| contig_252 | 2499 | 2341 | - | hypothetical protein |
| contig_252 | 3252 | 2569 | - | Mannose-1-phosphate guanylyltransferase (EC 2.7.7.13 ) / Phosphomannomutase (EC 5.4.2.8) |
| contig_252 | 3571 | 3266 | - | Mannose-1-phosphate guanylyltransferase (EC 2.7.7.13 ) / Phosphomannomutase (EC 5.4.2.8) |
| contig_252 | 4925 | 3630 | - | Mannose-1-phosphate guanylyltransferase (EC 2.7.7.13 ) / Phosphomannomutase (EC 5.4.2.8) |
| contig_2520 | 289 | 158 | - | hypothetical protein |
| contig_2521 | 120 | 788 | + | Enoyl-[acyl-carrier-protein] reductase [FMN] (EC 1.3.1.9) |
| contig_2521 | 1014 | 847 | - | Poly(glycerophosphate chain) D-alanine transfer protein DltD |
| contig_2523 | 415 | 161 | - | acetyltransferase, GNAT family |
| contig_2523 | 699 | 544 | - | acetyltransferase, GNAT family |
| contig_2523 | 1084 | 947 | - | hypothetical protein |
| contig_2524 | 163 | 47 | - | hypothetical protein |
| contig_2526 | 149 | 12 | - | hypothetical protein |
| contig_2528 | 72 | 248 | + | Transcriptional regulator, AraC family |
| contig_2529 | 232 | 71 | - | pXO1-08 |
| contig_253 | 797 | 1156 | + | Glutamate 5-kinase (EC 2.7.2.11) / RNA-binding C-terminal domain PUA |
| contig_253 | 1166 | 1750 | + | Glutamate 5-kinase (EC 2.7.2.11) / RNA-binding C-terminal domain PUA |
| contig_253 | 1778 | 1897 | + | Gamma-glutamyl phosphate reductase (EC 1.2.1.41) |
| contig_253 | 2037 | 2243 | + | Gamma-glutamyl phosphate reductase (EC 1.2.1.41) |
| contig_253 | 2358 | 2963 | + | Gamma-glutamyl phosphate reductase (EC 1.2.1.41) |
| contig_2530 | 850 | 485 | - | Penicillin-binding protein |
| contig_2530 | 960 | 805 | - | FIG01225684: hypothetical protein |
| contig_2531 | 334 | 74 | - | FIG01225689: hypothetical protein |
| contig_2531 | 438 | 304 | - | FIG01225689: hypothetical protein |
| contig_2532 | 272 | 108 | - | hypothetical protein |
| contig_2538 | 605 | 231 | - | DNA-binding response regulator |
| contig_2539 | 6 | 125 | + | hypothetical protein |
| contig_254 | 851 | 474 | - | Phage tail fiber protein |
| contig_254 | 1377 | 811 | - | Phage tail fiber protein |
| contig_254 | 1817 | 1494 | - | FIG01226865: hypothetical protein |
| contig_254 | 2062 | 1841 | - | FIG01228631: hypothetical protein |
| contig_254 | 2309 | 2139 | - | Rhodanese-like domain protein |
| contig_254 | 2646 | 2828 | + | FIG01225758: hypothetical protein |
| contig_254 | 3488 | 2877 | - | Homoserine O-acetyltransferase (EC 2.3.1.31) |
| contig_254 | 4000 | 3494 | - | Homoserine O-acetyltransferase (EC 2.3.1.31) |
| contig_254 | 4362 | 4502 | + | Spore germination protein GerHA/GerIA |
| contig_2540 | 268 | 131 | - | FIG01226085: hypothetical protein |
| contig_2540 | 645 | 460 | - | FIG01226085: hypothetical protein |
| contig_2540 | 871 | 758 | - | ABC transporter, substrate-binding protein |
| contig_2540 | 1631 | 849 | - | ABC transporter, substrate-binding protein |
| contig_2540 | 1737 | 1624 | - | hypothetical protein |
| contig_2540 | 2251 | 1697 | - | possible permease |
| contig_2540 | 2518 | 2381 | - | possible permease |
| contig_2540 | 2645 | 2523 | - | possible permease |
| contig_2543 | 254 | 102 | - | Transcriptional regulator, TrmB family |
| contig_2545 | 185 | 427 | + | Arginine/ornithine antiporter ArcD |
| contig_2547 | 78 | 194 | + | Lipid A export ATP-binding/permease protein MsbA |
| contig_255 | 252 | 115 | - | Phage protein |
| contig_255 | 368 | 252 | - | Phage protein |
| contig_255 | 1145 | 369 | - | Phage antirepressor protein |
| contig_255 | 1413 | 1213 | - | FIG01246605: hypothetical protein |
| contig_255 | 1753 | 1989 | + | transcriptional regulator |
| contig_255 | 2144 | 1995 | - | hypothetical protein |
| contig_255 | 2445 | 2296 | - | hypothetical protein |
| contig_255 | 3108 | 2452 | - | Phage DNA-binding protein @ Phage helix-turn-helix protein |
| contig_2551 | 1141 | 950 | - | Transcriptional regulator, AraC family |
| contig_2551 | 1415 | 1239 | - | Transcriptional regulator, AraC family |
| contig_2552 | 11 | 130 | + | FIG01226546: hypothetical protein |
| contig_2553 | 303 | 166 | - | hypothetical protein |
| contig_2554 | 260 | 39 | - | Putative membrane protein YfcA |
| contig_2554 | 811 | 257 | - | Putative membrane protein YfcA |
| contig_2555 | 475 | 242 | - | Flagellar hook-associated protein FlgL |
| contig_2558 | 234 | 115 | - | FIG01225447: hypothetical protein |
| contig_2558 | 427 | 275 | - | FIG01225447: hypothetical protein |
| contig_2559 | 224 | 99 | - | hypothetical protein |
| contig_2559 | 567 | 205 | - | FIG01227407: hypothetical protein |
| contig_256 | 17 | 280 | + | Collagen adhesin |
| contig_256 | 818 | 579 | - | FIG01225761: hypothetical protein |
| contig_256 | 1023 | 844 | - | FIG01225761: hypothetical protein |
| contig_256 | 1211 | 1516 | + | Sulfate permease |
| contig_256 | 1566 | 2573 | + | Sulfate permease |
| contig_2560 | 399 | 265 | - | FIG01229135: hypothetical protein |
| contig_2562 | 36 | 281 | + | Uncharacterized N-acetyltransferase BT9727_3663 (EC 2.3.1.-) |
| contig_2562 | 241 | 417 | + | Uncharacterized N-acetyltransferase BT9727_3663 (EC 2.3.1.-) |
| contig_2562 | 958 | 452 | - | Prespore specific transcriptional activator RsfA |
| contig_2563 | 5 | 451 | + | hypothetical protein |
| contig_2563 | 460 | 573 | + | hypothetical protein |
| contig_2564 | 240 | 124 | - | hypothetical protein |
| contig_2565 | 1109 | 1225 | + | hypothetical protein |
| contig_2567 | 177 | 43 | - | ATP-dependent RNA helicase BA2475 |
| contig_2567 | 399 | 196 | - | ATP-dependent RNA helicase BA2475 |
| contig_2568 | 11 | 145 | + | Chromosome-anchoring protein racA |
| contig_2568 | 529 | 236 | - | FIG01225585: hypothetical protein |
| contig_2568 | 915 | 787 | - | hypothetical protein |
| contig_2569 | 143 | 15 | - | FIG01227185: hypothetical protein |
| contig_2569 | 385 | 272 | - | FIG01227185: hypothetical protein |
| contig_2569 | 515 | 390 | - | FIG01227185: hypothetical protein |
| contig_257 | 223 | 1164 | + | Sporulation sigma-E factor processing peptidase (SpoIIGA) |
| contig_257 | 1161 | 1394 | + | RNA polymerase sporulation specific sigma factor SigE |
| contig_257 | 1431 | 1847 | + | RNA polymerase sporulation specific sigma factor SigE |
| contig_257 | 2039 | 2488 | + | RNA polymerase sporulation specific sigma factor SigG |
| contig_257 | 2466 | 2792 | + | RNA polymerase sporulation specific sigma factor SigG |
| contig_257 | 2994 | 3272 | + | FIG011856: hypothetical protein |
| contig_2570 | 150 | 335 | + | Oligopeptide ABC transporter, periplasmic oligopeptide-binding protein OppA (TC 3.A.1.5.1) |
| contig_2571 | 362 | 27 | - | FIG01225681: hypothetical protein |
| contig_2573 | 61 | 246 | + | membrane protein, putative |
| contig_2573 | 219 | 377 | + | membrane protein, putative |
| contig_2574 | 193 | 47 | - | phosphotransferase enzyme family protein, putative |
| contig_2574 | 803 | 633 | - | phosphotransferase enzyme family protein, putative |
| contig_2575 | 278 | 99 | - | FIG01225448: hypothetical protein |
| contig_2575 | 372 | 247 | - | FIG01225448: hypothetical protein |
| contig_2577 | 80 | 268 | + | Na+/H+ antiporter |
| contig_258 | 430 | 158 | - | periplasmic component of efflux system |
| contig_258 | 642 | 1376 | + | ABC transporter, permease protein, putative |
| contig_258 | 1493 | 1747 | + | Oligopeptide transport system permease protein OppB (TC 3.A.1.5.1) |
| contig_258 | 1820 | 1939 | + | Oligopeptide transport system permease protein OppB (TC 3.A.1.5.1) |
| contig_258 | 1953 | 2078 | + | Oligopeptide transport system permease protein OppB (TC 3.A.1.5.1) |
| contig_258 | 2337 | 3104 | + | Oligopeptide transport system permease protein OppC (TC 3.A.1.5.1) |
| contig_2580 | 273 | 73 | - | Chromosome initiation inhibitor |
| contig_2582 | 480 | 46 | - | 3-hydroxyacyl-[acyl-carrier-protein] dehydratase, FabZ form (EC 4.2.1.59) |
| contig_2584 | 261 | 121 | - | Ketosteroid isomerase-related protein |
| contig_2585 | 369 | 124 | - | Ketosteroid isomerase-related protein |
| contig_2586 | 204 | 16 | - | FIG01225947: hypothetical protein |
| contig_2586 | 382 | 179 | - | FIG01225947: hypothetical protein |
| contig_2586 | 778 | 524 | - | FIG01225947: hypothetical protein |
| contig_2586 | 906 | 775 | - | MoxR-like ATPases |
| contig_2586 | 1494 | 1045 | - | MoxR-like ATPases |
| contig_2586 | 1711 | 1538 | - | MoxR-like ATPases |
| contig_2586 | 1919 | 2113 | + | Transcriptional regulator, PadR family |
| contig_2586 | 2115 | 2237 | + | Transcriptional regulator, PadR family |
| contig_2586 | 2279 | 2629 | + | FIG01225222: hypothetical protein |
| contig_2587 | 168 | 767 | + | 5-carboxymethyl-2-hydroxymuconate semialdehyde dehydrogenase (EC 1.2.1.60) |
| contig_2587 | 737 | 1210 | + | 5-carboxymethyl-2-hydroxymuconate semialdehyde dehydrogenase (EC 1.2.1.60) |
| contig_2588 | 482 | 168 | - | Oligopeptide ABC transporter, periplasmic oligopeptide-binding protein OppA (TC 3.A.1.5.1) |
| contig_2589 | 41 | 274 | + | Acetaldehyde dehydrogenase, acetylating, (EC 1.2.1.10) in gene cluster for degradation of phenols, cresols, catechol |
| contig_2589 | 354 | 827 | + | Acetaldehyde dehydrogenase, acetylating, (EC 1.2.1.10) in gene cluster for degradation of phenols, cresols, catechol |
| contig_259 | 191 | 346 | + | FIG01229673: hypothetical protein |
| contig_259 | 530 | 1021 | + | FIG01231607: hypothetical protein |
| contig_259 | 1218 | 1057 | - | FIG01227510: hypothetical protein |
| contig_259 | 1598 | 1840 | + | FIG01225195: hypothetical protein |
| contig_259 | 3635 | 2241 | - | L-cystine uptake protein TcyP |
| contig_2591 | 292 | 38 | - | ABC transporter permease protein YvcS |
| contig_2591 | 525 | 265 | - | ABC transporter permease protein YvcS |
| contig_2593 | 260 | 373 | + | Ribosomal-protein-alanine acetyltransferase (EC 2.3.1.128) |
| contig_2594 | 604 | 413 | - | Hypothetical protein in cluster with SinR and SinI |
| contig_2595 | 612 | 196 | - | 5'-methylthioadenosine/S-adenosylhomocysteine nucleosidase related protein BCZK2595 / hydrolase, haloacid dehalogenase-like family protein BCZK2594 |
| contig_2595 | 1017 | 781 | - | 5'-methylthioadenosine/S-adenosylhomocysteine nucleosidase related protein BCZK2595 / hydrolase, haloacid dehalogenase-like family protein BCZK2594 |
| contig_2597 | 492 | 752 | + | Phosphoglycerate mutase (EC 5.4.2.1) |
| contig_2597 | 712 | 1230 | + | Phosphoglycerate mutase (EC 5.4.2.1) |
| contig_2598 | 61 | 210 | + | FIG01239590: hypothetical protein |
| contig_2599 | 212 | 718 | + | Tn7-like transposition protein A |
| contig_26 | 366 | 641 | + | DNA topoisomerase III (EC 5.99.1.2) |
| contig_26 | 1377 | 682 | - | phage infection protein |
| contig_26 | 1615 | 1418 | - | phage infection protein |
| contig_26 | 1892 | 1566 | - | phage infection protein |
| contig_26 | 3548 | 2244 | - | phage infection protein |
| contig_26 | 4647 | 3856 | - | Exodeoxyribonuclease III (EC 3.1.11.2) |
| contig_26 | 5308 | 4652 | - | PTS system, maltose and glucose-specific IIC component (EC 2.7.1.69) / PTS system, maltose and glucose-specific IIB component (EC 2.7.1.69) |
| contig_26 | 6290 | 5277 | - | PTS system, maltose and glucose-specific IIC component (EC 2.7.1.69) / PTS system, maltose and glucose-specific IIB component (EC 2.7.1.69) |
| contig_260 | 1462 | 1019 | - | tmRNA-binding protein SmpB |
| contig_260 | 1556 | 1434 | - | tmRNA-binding protein SmpB |
| contig_260 | 1542 | 1706 | + | hypothetical protein |
| contig_260 | 3884 | 1806 | - | 3'-to-5' exoribonuclease RNase R |
| contig_260 | 4340 | 4179 | - | Carboxylesterase (EC 3.1.1.1) |
| contig_2600 | 469 | 113 | - | D-xylose proton-symporter XylE |
| contig_2602 | 513 | 199 | - | Deoxyribonucleoside regulator DeoR (transcriptional repressor) |
| contig_2602 | 1147 | 470 | - | Deoxyribonucleoside regulator DeoR (transcriptional repressor) |
| contig_2603 | 581 | 135 | - | Chorismate synthase (EC 4.2.3.5) |
| contig_2604 | 287 | 144 | - | Aspartate racemase (EC 5.1.1.13) |
| contig_2605 | 575 | 72 | - | Oligopeptide transport ATP-binding protein OppF (TC 3.A.1.5.1) |
| contig_2606 | 498 | 268 | - | Glycerate kinase (EC 2.7.1.31) |
| contig_2606 | 757 | 479 | - | Glycerate kinase (EC 2.7.1.31) |
| contig_2606 | 1127 | 744 | - | Glycerate kinase (EC 2.7.1.31) |
| contig_2606 | 1342 | 1214 | - | Glycerate kinase (EC 2.7.1.31) |
| contig_2609 | 283 | 510 | + | FIG01225476: hypothetical protein |
| contig_261 | 1233 | 442 | - | Cell division protein FtsI [Peptidoglycan synthetase] (EC 2.4.1.129) |
| contig_261 | 1924 | 1217 | - | Cell division protein FtsI [Peptidoglycan synthetase] (EC 2.4.1.129) |
| contig_2610 | 482 | 195 | - | Glutathione peroxidase family protein |
| contig_2611 | 275 | 496 | + | ABC transporter ATP-binding protein |
| contig_2611 | 562 | 1002 | + | membrane protein, putative |
| contig_2611 | 1039 | 1173 | + | membrane protein, putative |
| contig_2612 | 292 | 122 | - | LPXTG-motif cell wall anchor domain protein |
| contig_2612 | 612 | 436 | - | LPXTG-motif cell wall anchor domain protein |
| contig_2612 | 1216 | 698 | - | LPXTG-motif cell wall anchor domain protein |
| contig_2614 | 93 | 569 | + | putative membrane protein |
| contig_2615 | 77 | 481 | + | S-layer homology domain protein |
| contig_2616 | 120 | 7 | - | Two-component response regulator, malate (EC 2.7.3.-) |
| contig_2616 | 263 | 120 | - | Two-component response regulator, malate (EC 2.7.3.-) |
| contig_2616 | 712 | 419 | - | Two-component response regulator, malate (EC 2.7.3.-) |
| contig_2616 | 1284 | 709 | - | Two-component sensor histidine kinase, malate (EC 2.7.3.-) |
| contig_2618 | 254 | 12 | - | GTP-binding protein HflX |
| contig_2619 | 131 | 12 | - | GTP-binding protein HflX |
| contig_262 | 1656 | 712 | - | 3'->5' exoribonuclease Bsu YhaM |
| contig_262 | 2261 | 1698 | - | DNA double-strand break repair Rad50 ATPase |
| contig_262 | 2436 | 2233 | - | DNA double-strand break repair Rad50 ATPase |
| contig_262 | 2884 | 2453 | - | DNA double-strand break repair Rad50 ATPase |
| contig_262 | 3380 | 2859 | - | DNA double-strand break repair Rad50 ATPase |
| contig_262 | 4626 | 3451 | - | DNA double-strand break repair Rad50 ATPase |
| contig_262 | 5165 | 4623 | - | DNA double-strand break repair protein Mre11 |
| contig_262 | 5865 | 5212 | - | DNA double-strand break repair protein Mre11 |
| contig_262 | 6026 | 6187 | + | FIG012187: hypothetical protein |
| contig_262 | 6408 | 6214 | - | FIG011566: hypothetical protein |
| contig_2621 | 265 | 576 | + | Teicoplanin resistance protein vanZ |
| contig_2622 | 574 | 317 | - | FIG01227739: hypothetical protein |
| contig_2622 | 790 | 584 | - | FIG01227739: hypothetical protein |
| contig_2625 | 650 | 18 | - | Glucose 1-dehydrogenase (EC 1.1.1.47) |
| contig_2626 | 173 | 54 | - | amidase family protein |
| contig_2629 | 286 | 429 | + | hypothetical protein |
| contig_263 | 116 | 1658 | + | Small Subunit Ribosomal RNA; ssuRNA; SSU rRNA |
| contig_263 | 1803 | 4583 | + | Large Subunit Ribosomal RNA; lsuRNA; LSU rRNA |
| contig_2630 | 361 | 149 | - | FIG006438: hypothetical protein |
| contig_2631 | 173 | 36 | - | Similar to ribosomal large subunit pseudouridine synthase F, group RluF1 |
| contig_2632 | 199 | 56 | - | FIG01228881: hypothetical protein |
| contig_2632 | 501 | 244 | - | FIG01228881: hypothetical protein |
| contig_2632 | 658 | 485 | - | FIG01228881: hypothetical protein |
| contig_2635 | 409 | 44 | - | transposase (08) |
| contig_2636 | 988 | 203 | - | D-alanine--D-alanine ligase (EC 6.3.2.4) |
| contig_2636 | 1111 | 992 | - | hypothetical protein |
| contig_2636 | 1361 | 1158 | - | Transcriptional regulator, GntR family domain / Aspartate aminotransferase (EC 2.6.1.1) |
| contig_2637 | 228 | 88 | - | hypothetical protein |
| contig_2637 | 338 | 225 | - | Glycerate kinase (EC 2.7.1.31) |
| contig_2637 | 495 | 289 | - | Glycerate kinase (EC 2.7.1.31) |
| contig_2637 | 644 | 510 | - | Glycerate kinase (EC 2.7.1.31) |
| contig_2637 | 929 | 795 | - | N-hydroxyarylamine O-acetyltransferase (EC 2.3.1.118) |
| contig_2639 | 47 | 292 | + | ABC transporter, permease protein, putative |
| contig_264 | 460 | 347 | - | hypothetical protein |
| contig_264 | 761 | 639 | - | acetyltransferase, GNAT family |
| contig_264 | 1146 | 739 | - | acetyltransferase, GNAT family |
| contig_264 | 1762 | 1439 | - | Osmosensitive K+ channel histidine kinase KdpD (EC 2.7.3.-) |
| contig_264 | 2054 | 1851 | - | Osmosensitive K+ channel histidine kinase KdpD (EC 2.7.3.-) |
| contig_264 | 2385 | 2101 | - | Osmosensitive K+ channel histidine kinase KdpD (EC 2.7.3.-) |
| contig_2640 | 148 | 393 | + | Putative membrane protein YfcA |
| contig_2640 | 454 | 633 | + | Putative membrane protein YfcA |
| contig_2641 | 189 | 971 | + | hydrolase, alpha/beta fold family |
| contig_2641 | 1361 | 990 | - | FIG01225201: hypothetical protein |
| contig_2641 | 1761 | 1450 | - | Phosphoglycerate mutase family 2 |
| contig_2643 | 25 | 657 | + | RNA polymerase sigma-70 factor, ECF subfamily |
| contig_2643 | 682 | 936 | + | ATP-dependent Clp protease proteolytic subunit (EC 3.4.21.92) |
| contig_2644 | 366 | 121 | - | Oligopeptide ABC transporter, periplasmic oligopeptide-binding protein OppA (TC 3.A.1.5.1) |
| contig_2645 | 100 | 330 | + | Hypothetical protein SAV1846 |
| contig_2647 | 96 | 650 | + | membrane protein, putative |
| contig_2648 | 26 | 229 | + | FIG012639: hypothetical Membrane Spanning Protein |
| contig_2649 | 355 | 230 | - | oxidoreductase, aldo/keto reductase family |
| contig_2649 | 734 | 333 | - | oxidoreductase, aldo/keto reductase family |
| contig_2649 | 1056 | 829 | - | oxidoreductase, aldo/keto reductase family |
| contig_265 | 162 | 383 | + | FIG01225565: hypothetical protein |
| contig_2652 | 203 | 81 | - | Bacillolysin (EC 3.4.24.28) |
| contig_2652 | 386 | 228 | - | Bacillolysin (EC 3.4.24.28) |
| contig_2653 | 683 | 147 | - | oxetanocin A resistance protein |
| contig_2653 | 864 | 676 | - | oxetanocin A resistance protein |
| contig_2654 | 23 | 724 | + | Spore germination protein GerLC |
| contig_2655 | 41 | 181 | + | Na+ dependent nucleoside transporter NupC |
| contig_2656 | 308 | 144 | - | Regulatory sensor-transducer, BlaR1/MecR1 family |
| contig_2657 | 382 | 251 | - | Predicted N-ribosylNicotinamide CRP-like regulator |
| contig_2657 | 558 | 716 | + | FIG00517589: hypothetical protein |
| contig_2657 | 742 | 933 | + | FIG00517589: hypothetical protein |
| contig_2658 | 1000 | 887 | - | hypothetical protein |
| contig_266 | 634 | 125 | - | Ribosomal-protein-alanine acetyltransferase (EC 2.3.1.128) |
| contig_2660 | 601 | 239 | - | Protein export cytoplasm protein SecA ATPase RNA helicase (TC 3.A.5.1.1) |
| contig_2662 | 149 | 6 | - | Mobile element protein |
| contig_2663 | 396 | 247 | - | Transcriptional regulator, XRE family |
| contig_2663 | 886 | 380 | - | Transcriptional regulator, XRE family |
| contig_2664 | 73 | 393 | + | Protein export cytoplasm protein SecA ATPase RNA helicase (TC 3.A.5.1.1) |
| contig_2664 | 420 | 548 | + | Protein export cytoplasm protein SecA ATPase RNA helicase (TC 3.A.5.1.1) |
| contig_2664 | 775 | 587 | - | Sorbitol-6-phosphate 2-dehydrogenase (EC 1.1.1.140) |
| contig_2664 | 938 | 801 | - | Sorbitol-6-phosphate 2-dehydrogenase (EC 1.1.1.140) |
| contig_2664 | 1399 | 1250 | - | Sorbitol-6-phosphate 2-dehydrogenase (EC 1.1.1.140) |
| contig_2667 | 230 | 51 | - | hypothetical protein |
| contig_2668 | 426 | 262 | - | monooxygenase, putative |
| contig_2668 | 694 | 515 | - | serine/threonine protein phosphatase family |
| contig_2668 | 1245 | 805 | - | serine/threonine protein phosphatase family |
| contig_2669 | 318 | 527 | + | phage transcriptional regulator, ArpU family subfamily |
| contig_2669 | 508 | 639 | + | phage transcriptional regulator, ArpU family subfamily |
| contig_267 | 279 | 590 | + | membrane protein, putative |
| contig_267 | 646 | 1107 | + | membrane protein, putative |
| contig_267 | 1483 | 1172 | - | FIG01232668: hypothetical protein |
| contig_267 | 1651 | 1764 | + | hypothetical protein |
| contig_267 | 1896 | 2570 | + | Tryptophanyl-tRNA synthetase (EC 6.1.1.2) |
| contig_267 | 2660 | 2803 | + | Tryptophanyl-tRNA synthetase (EC 6.1.1.2) |
| contig_267 | 2781 | 2897 | + | Tryptophanyl-tRNA synthetase (EC 6.1.1.2) |
| contig_267 | 3188 | 3637 | + | Maltose O-acetyltransferase (EC 2.3.1.79) |
| contig_2672 | 227 | 111 | - | hypothetical protein |
| contig_2673 | 345 | 151 | - | hypothetical protein |
| contig_2679 | 276 | 88 | - | S-layer protein, putative |
| contig_268 | 327 | 442 | + | 5S RNA |
| contig_2680 | 185 | 72 | - | hydrolase, alpha/beta fold family |
| contig_2680 | 504 | 286 | - | hydrolase, alpha/beta fold family |
| contig_2680 | 905 | 621 | - | hydrolase, alpha/beta fold family |
| contig_2681 | 246 | 374 | + | Histidine kinase of the competence regulon ComD |
| contig_2681 | 652 | 765 | + | Histidine kinase of the competence regulon ComD |
| contig_2682 | 190 | 8 | - | hypothetical protein |
| contig_2683 | 749 | 198 | - | FIG01225212: hypothetical protein |
| contig_2683 | 898 | 773 | - | hypothetical protein |
| contig_2684 | 25 | 177 | + | Late competence protein ComGE, FIG015513 |
| contig_269 | 48 | 163 | + | 5S RNA |
| contig_269 | 173 | 247 | + | tRNA-Asn-GTT |
| contig_269 | 250 | 341 | + | tRNA-Ser-GGA |
| contig_269 | 359 | 433 | + | tRNA-Glu-TTC |
| contig_269 | 438 | 513 | + | tRNA-Val-TAC |
| contig_269 | 538 | 614 | + | tRNA-Met-CAT |
| contig_269 | 618 | 693 | + | tRNA-Asp-GTC |
| contig_269 | 703 | 778 | + | tRNA-Phe-GAA |
| contig_269 | 797 | 872 | + | tRNA-Thr-TGT |
| contig_269 | 883 | 966 | + | tRNA-Tyr-GTA |
| contig_269 | 974 | 1047 | + | tRNA-Trp-CCA |
| contig_269 | 1067 | 1142 | + | tRNA-His-GTG |
| contig_269 | 1206 | 1280 | + | tRNA-Gln-TTG |
| contig_269 | 1286 | 1360 | + | tRNA-Gly-GCC |
| contig_269 | 1375 | 1445 | + | tRNA-Cys-GCA |
| contig_269 | 1456 | 1540 | + | tRNA-Leu-CAA |
| contig_269 | 1677 | 1973 | + | Phage-related integrase/recombinase |
| contig_269 | 2044 | 2427 | + | integrase/recombinase |
| contig_269 | 2441 | 2650 | + | integrase/recombinase |
| contig_269 | 3474 | 3611 | + | hypothetical protein |
| contig_269 | 3840 | 4007 | + | hypothetical protein |
| contig_269 | 4059 | 4592 | + | hypothetical protein |
| contig_269 | 5264 | 4908 | - | hypothetical protein |
| contig_269 | 5583 | 5269 | - | hypothetical protein |
| contig_269 | 6004 | 5762 | - | hypothetical protein |
| contig_2690 | 719 | 138 | - | Methyltransferase (EC 2.1.1.-) |
| contig_2692 | 43 | 630 | + | FIG01228744: hypothetical protein |
| contig_2695 | 314 | 3 | - | Integral membrane protein |
| contig_2698 | 421 | 197 | - | FIG01226009: hypothetical protein |
| contig_2699 | 168 | 52 | - | Chitin binding protein |
| contig_2699 | 865 | 152 | - | Chitin binding protein |
| contig_2699 | 1164 | 1030 | - | Chitin binding protein |
| contig_27 | 166 | 32 | - | Chitin binding protein |
| contig_270 | 51 | 269 | + | Glutamate N-acetyltransferase (EC 2.3.1.35) / N-acetylglutamate synthase (EC 2.3.1.1) |
| contig_270 | 259 | 408 | + | Glutamate N-acetyltransferase (EC 2.3.1.35) / N-acetylglutamate synthase (EC 2.3.1.1) |
| contig_270 | 420 | 971 | + | Acetylglutamate kinase (EC 2.7.2.8) |
| contig_270 | 1009 | 1188 | + | Acetylglutamate kinase (EC 2.7.2.8) |
| contig_270 | 1185 | 2345 | + | Acetylornithine aminotransferase (EC 2.6.1.11) |
| contig_270 | 2358 | 3272 | + | Ornithine carbamoyltransferase (EC 2.1.3.3) |
| contig_270 | 3482 | 3826 | + | FIG01226037: hypothetical protein |
| contig_270 | 3897 | 4043 | + | FIG01226037: hypothetical protein |
| contig_270 | 4102 | 4299 | + | FIG01227927: hypothetical protein |
| contig_270 | 4461 | 4324 | - | Transcriptional regulator, MarR family |
| contig_270 | 4774 | 4628 | - | Transcriptional regulator, MarR family |
| contig_270 | 5233 | 4847 | - | FIG006988: Lipase/Acylhydrolase with GDSL-like motif |
| contig_2700 | 347 | 75 | - | FIG01227287: hypothetical protein |
| contig_2700 | 683 | 504 | - | FIG01229340: hypothetical protein |
| contig_2700 | 1008 | 745 | - | microcin immunity protein MccF |
| contig_2701 | 297 | 40 | - | ABC transporter, ATP-binding protein |
| contig_2702 | 441 | 88 | - | Catalyzes the cleavage of p-aminobenzoyl-glutamate to p-aminobenzoate and glutamate, subunit A |
| contig_2704 | 233 | 111 | - | hypothetical protein |
| contig_2704 | 447 | 238 | - | Putative transcriptional antiterminator, BglG family / PTS system, mannitol/fructose-specific IIA component (EC 2.7.1.69) |
| contig_2705 | 80 | 265 | + | potassium uptake protein, TrkH family |
| contig_2708 | 433 | 44 | - | Duplicated ATPase component CbrU of energizing module of predicted cobalamin ECF transporter |
| contig_271 | 488 | 222 | - | hypothetical protein |
| contig_2710 | 227 | 33 | - | FIG01234939: hypothetical protein |
| contig_2714 | 403 | 230 | - | Agmatine deiminase (EC 3.5.3.12) |
| contig_2717 | 345 | 16 | - | RND multidrug efflux transporter; Acriflavin resistance protein |
| contig_2717 | 975 | 439 | - | RND multidrug efflux transporter; Acriflavin resistance protein |
| contig_2718 | 482 | 222 | - | Chloramphenicol acetyltransferase (EC 2.3.1.28) |
| contig_2718 | 680 | 501 | - | Chloramphenicol acetyltransferase (EC 2.3.1.28) |
| contig_2719 | 73 | 828 | + | L-Proline/Glycine betaine transporter ProP |
| contig_2721 | 55 | 225 | + | FIG01234196: hypothetical protein |
| contig_2721 | 542 | 375 | - | carboxymuconolactone decarboxylase |
| contig_2723 | 583 | 191 | - | Oligosaccharide repeat unit polymerase Wzy |
| contig_2727 | 41 | 475 | + | FIG01239327: hypothetical protein |
| contig_2728 | 115 | 273 | + | Purine nucleoside phosphorylase (EC 2.4.2.1) |
| contig_2729 | 372 | 124 | - | FIG01228233: hypothetical protein |
| contig_2730 | 484 | 329 | - | Lactoylglutathione lyase and related lyases |
| contig_2732 | 103 | 420 | + | oxidoreductase, Gfo/Idh/MocA family |
| contig_2733 | 199 | 41 | - | Agmatine deiminase (EC 3.5.3.12) |
| contig_2734 | 333 | 193 | - | FIG01227784: hypothetical protein |
| contig_2735 | 25 | 717 | + | drug resistance transporter, Bcr/CflA family |
| contig_2736 | 408 | 166 | - | Cell division protein FtsI [Peptidoglycan synthetase] (EC 2.4.1.129) / Stage V sporulation protein D (Sporulation-specific penicillin-binding protein) |
| contig_2737 | 790 | 161 | - | Chromosome initiation inhibitor |
| contig_2737 | 1001 | 825 | - | Chromosome initiation inhibitor |
| contig_2737 | 1124 | 1762 | + | quinone oxidoreductase |
| contig_274 | 27 | 335 | + | Long-chain-fatty-acid--CoA ligase (EC 6.2.1.3) |
| contig_274 | 385 | 1419 | + | 3-ketoacyl-CoA thiolase (EC 2.3.1.16) @ Acetyl-CoA acetyltransferase (EC 2.3.1.9) |
| contig_274 | 2032 | 1427 | - | FIG01227087: hypothetical protein |
| contig_274 | 2112 | 2555 | + | Nudix hydrolase family protein |
| contig_274 | 2977 | 2600 | - | Alanyl-tRNA synthetase family protein |
| contig_274 | 3329 | 2970 | - | Alanyl-tRNA synthetase family protein |
| contig_274 | 3516 | 3331 | - | Alanyl-tRNA synthetase family protein |
| contig_274 | 3838 | 3470 | - | Alanyl-tRNA synthetase family protein |
| contig_274 | 4008 | 4325 | + | FIG01227058: hypothetical protein |
| contig_274 | 4449 | 5006 | + | Multidrug resistance protein B |
| contig_274 | 5017 | 5280 | + | Multidrug resistance protein B |
| contig_274 | 5414 | 5689 | + | hypothetical protein |
| contig_2740 | 271 | 143 | - | Acetoacetyl-CoA reductase (EC 1.1.1.36) |
| contig_2740 | 506 | 336 | - | Acetoacetyl-CoA reductase (EC 1.1.1.36) |
| contig_2742 | 291 | 46 | - | Chemotaxis protein CheV (EC 2.7.3.-) |
| contig_2742 | 475 | 269 | - | Chemotaxis protein CheV (EC 2.7.3.-) |
| contig_2742 | 922 | 605 | - | FIG01226809: hypothetical protein |
| contig_2742 | 1089 | 919 | - | FIG01226809: hypothetical protein |
| contig_2744 | 43 | 282 | + | Single-stranded DNA-binding protein |
| contig_2745 | 317 | 90 | - | APH, Aminoglycoside phosphotransferase |
| contig_2745 | 680 | 492 | - | APH, Aminoglycoside phosphotransferase |
| contig_2746 | 661 | 194 | - | Alpha/beta hydrolase fold (EC 3.8.1.5) |
| contig_2747 | 318 | 109 | - | Ribonucleotide reductase of class II (coenzyme B12-dependent) (EC 1.17.4.1) |
| contig_2749 | 200 | 72 | - | hypothetical protein |
| contig_2749 | 328 | 197 | - | Thioredoxin |
| contig_275 | 212 | 57 | - | Chaperone protein DnaK |
| contig_275 | 1683 | 181 | - | Chaperone protein DnaK |
| contig_275 | 1894 | 1649 | - | Chaperone protein DnaK |
| contig_275 | 2358 | 1921 | - | Heat shock protein GrpE |
| contig_275 | 2501 | 2385 | - | Heat shock protein GrpE |
| contig_275 | 2778 | 2617 | - | Heat-inducible transcription repressor HrcA |
| contig_275 | 3640 | 2798 | - | Heat-inducible transcription repressor HrcA |
| contig_275 | 4838 | 3774 | - | Hypothetical radical SAM family enzyme in heat shock gene cluster, similarity with CPO of BS HemN-type |
| contig_2750 | 219 | 43 | - | Catalyzes the cleavage of p-aminobenzoyl-glutamate to p-aminobenzoate and glutamate, subunit A |
| contig_2751 | 53 | 277 | + | FIG01227536: hypothetical protein |
| contig_2751 | 550 | 344 | - | hypothetical protein |
| contig_2753 | 100 | 324 | + | Inosine-uridine preferring nucleoside hydrolase (EC 3.2.2.1) |
| contig_2754 | 549 | 764 | + | D-alanyl-D-alanine carboxypeptidase (EC 3.4.16.4) |
| contig_2755 | 29 | 232 | + | Oligopeptide ABC transporter, periplasmic oligopeptide-binding protein OppA (TC 3.A.1.5.1) |
| contig_2756 | 206 | 15 | - | 5'-methylthioadenosine/S-adenosylhomocysteine nucleosidase related protein BCZK2595 / hydrolase, haloacid dehalogenase-like family protein BCZK2594 |
| contig_2757 | 599 | 24 | - | Acetyl-CoA:acetoacetyl-CoA transferase, beta subunit (EC 2.8.3.8) |
| contig_2758 | 592 | 434 | - | mutT/nudix family protein |
| contig_2759 | 291 | 154 | - | 1-pyrroline-4-hydroxy-2-carboxylate deaminase (EC 3.5.4.22) |
| contig_2759 | 474 | 343 | - | 1-pyrroline-4-hydroxy-2-carboxylate deaminase (EC 3.5.4.22) |
| contig_276 | 496 | 173 | - | general stress protein 26 |
| contig_276 | 770 | 1825 | + | Calcium/proton antiporter |
| contig_276 | 1970 | 2482 | + | FIG01226204: hypothetical protein |
| contig_276 | 2487 | 2705 | + | FIG01226204: hypothetical protein |
| contig_276 | 3210 | 2680 | - | Thioredoxin-like oxidoreductases |
| contig_276 | 3358 | 3221 | - | Thioredoxin-like oxidoreductases |
| contig_2761 | 452 | 255 | - | FIG01230588: hypothetical protein |
| contig_2766 | 383 | 12 | - | Replicative DNA helicase (EC 3.6.1.-) |
| contig_2766 | 934 | 380 | - | DnaD domain protein |
| contig_2767 | 182 | 45 | - | sensory box/GGDEF family protein, putative |
| contig_2767 | 614 | 498 | - | sensory box/GGDEF family protein, putative |
| contig_2768 | 131 | 469 | + | conserved hypothetical protein, truncation |
| contig_2768 | 894 | 724 | - | hypothetical protein |
| contig_277 | 102 | 1868 | + | Transcription accessory protein (S1 RNA-binding domain) |
| contig_277 | 1968 | 1849 | - | hypothetical protein |
| contig_277 | 2236 | 2481 | + | Putative metallopeptidase (Zinc) SprT family |
| contig_277 | 2546 | 2695 | + | Putative metallopeptidase (Zinc) SprT family |
| contig_277 | 2810 | 2884 | + | tRNA-Asn-GTT |
| contig_277 | 2888 | 2978 | + | tRNA-Ser-GCT |
| contig_277 | 2987 | 3061 | + | tRNA-Glu-TTC |
| contig_277 | 3066 | 3141 | + | tRNA-Val-TAC |
| contig_277 | 3188 | 3263 | + | tRNA-Asp-GTC |
| contig_277 | 3351 | 3425 | + | tRNA-Gln-TTG |
| contig_2770 | 193 | 23 | - | Mg(2+) transport ATPase protein C |
| contig_2770 | 514 | 284 | - | Mg(2+) transport ATPase protein C |
| contig_2771 | 259 | 140 | - | Phosphohydrolase (MutT/nudix family protein) |
| contig_2772 | 72 | 404 | + | Permease of the drug/metabolite transporter (DMT) superfamily |
| contig_2773 | 225 | 359 | + | Cell division protein FtsX |
| contig_2776 | 41 | 280 | + | 8-amino-7-oxononanoate synthase (EC 2.3.1.47) |
| contig_2779 | 226 | 98 | - | FIG01227596: hypothetical protein |
| contig_278 | 21 | 96 | + | tRNA-Lys-TTT |
| contig_278 | 111 | 191 | + | tRNA-Leu-TAG |
| contig_278 | 221 | 295 | + | tRNA-Gly-GCC |
| contig_278 | 312 | 400 | + | tRNA-Leu-TAA |
| contig_278 | 404 | 477 | + | tRNA-Arg-ACG |
| contig_278 | 488 | 561 | + | tRNA-Pro-TGG |
| contig_278 | 577 | 649 | + | tRNA-Ala-TGC |
| contig_278 | 670 | 746 | + | tRNA-Met-CAT |
| contig_278 | 751 | 827 | + | tRNA-Met-CAT |
| contig_278 | 845 | 937 | + | tRNA-Ser-TGA |
| contig_2780 | 370 | 206 | - | Transcriptional regulator, PadR family |
| contig_2784 | 198 | 338 | + | hypothetical protein |
| contig_2786 | 808 | 392 | - | acetyltransferase, GNAT family |
| contig_2788 | 173 | 60 | - | UDP-N-acetylmuramoylalanyl-D-glutamyl-2,6-diaminopimelate--D-alanyl-D-alanine ligase (EC 6.3.2.10) |
| contig_2788 | 576 | 139 | - | UDP-N-acetylmuramoylalanyl-D-glutamyl-2,6-diaminopimelate--D-alanyl-D-alanine ligase (EC 6.3.2.10) |
| contig_2788 | 734 | 585 | - | UDP-N-acetylmuramoylalanyl-D-glutamyl-2,6-diaminopimelate--D-alanyl-D-alanine ligase (EC 6.3.2.10) |
| contig_2789 | 244 | 372 | + | hypothetical protein |
| contig_2789 | 696 | 550 | - | FIG01230164: hypothetical protein |
| contig_279 | 22 | 98 | + | tRNA-Met-CAT |
| contig_279 | 100 | 175 | + | tRNA-Asp-GTC |
| contig_279 | 188 | 263 | + | tRNA-Phe-GAA |
| contig_279 | 278 | 353 | + | tRNA-Thr-TGT |
| contig_279 | 364 | 439 | + | tRNA-Lys-TTT |
| contig_279 | 453 | 523 | + | tRNA-Gly-TCC |
| contig_279 | 534 | 610 | + | tRNA-Ile-GAT |
| contig_279 | 618 | 692 | + | tRNA-Asn-GTT |
| contig_279 | 700 | 790 | + | tRNA-Ser-GCT |
| contig_279 | 797 | 868 | + | tRNA-Glu-TTC |
| contig_2790 | 770 | 654 | - | Chitosanase |
| contig_2791 | 522 | 343 | - | Potassium efflux system KefA protein / Small-conductance mechanosensitive channel |
| contig_2792 | 716 | 303 | - | Aspartate-semialdehyde dehydrogenase (EC 1.2.1.11) |
| contig_2792 | 1065 | 775 | - | Aspartate-semialdehyde dehydrogenase (EC 1.2.1.11) |
| contig_2792 | 1323 | 1186 | - | Dipicolinate synthase subunit B |
| contig_2794 | 517 | 651 | + | hypothetical protein |
| contig_2795 | 293 | 174 | - | hypothetical protein |
| contig_2796 | 99 | 512 | + | YcgQ-like protein |
| contig_2796 | 784 | 1290 | + | cytosolic long-chain acyl-CoA thioester hydrolase family protein |
| contig_28 | 452 | 48 | - | Chitin binding protein |
| contig_28 | 1610 | 2029 | + | FIG01235405: hypothetical protein |
| contig_28 | 2315 | 2109 | - | Small acid-soluble spore protein |
| contig_28 | 2580 | 2870 | + | Aspartyl-tRNA(Asn) amidotransferase subunit C (EC 6.3.5.6) @ Glutamyl-tRNA(Gln) amidotransferase subunit C (EC 6.3.5.7) |
| contig_28 | 2886 | 3080 | + | Aspartyl-tRNA(Asn) amidotransferase subunit A (EC 6.3.5.6) @ Glutamyl-tRNA(Gln) amidotransferase subunit A (EC 6.3.5.7) |
| contig_280 | 29 | 142 | + | CBS domain protein, lmo1865 homolog |
| contig_280 | 193 | 534 | + | ATP/GTP-binding protein, SA1392 homolog |
| contig_280 | 572 | 841 | + | ATP/GTP-binding protein, SA1392 homolog |
| contig_280 | 819 | 1028 | + | ATP/GTP-binding protein, SA1392 homolog |
| contig_2800 | 93 | 632 | + | CAAX amino terminal protease family protein (Ste24 endopeptidase) (EC 3.4.24.84) |
| contig_2801 | 336 | 43 | - | FIG01225320: hypothetical protein |
| contig_2802 | 372 | 566 | + | Membrane protein involved in the export of O-antigen, teichoic acid lipoteichoic acids |
| contig_2805 | 215 | 469 | + | FIG01247645: hypothetical protein |
| contig_2807 | 305 | 138 | - | FIG01225932: hypothetical protein |
| contig_2808 | 129 | 7 | - | Methyltransferase |
| contig_2808 | 346 | 191 | - | Methyltransferase |
| contig_2808 | 493 | 347 | - | Methyltransferase |
| contig_2809 | 265 | 38 | - | membrane protein, putative |
| contig_2809 | 1025 | 861 | - | ATP synthase protein I |
| contig_281 | 400 | 209 | - | Transcriptional regulator, GntR family domain / Aspartate aminotransferase (EC 2.6.1.1) |
| contig_281 | 800 | 354 | - | Transcriptional regulator, GntR family domain / Aspartate aminotransferase (EC 2.6.1.1) |
| contig_2810 | 237 | 106 | - | Multimodular transpeptidase-transglycosylase (EC 2.4.1.129) (EC 3.4.-.-) |
| contig_2810 | 451 | 215 | - | Multimodular transpeptidase-transglycosylase (EC 2.4.1.129) (EC 3.4.-.-) |
| contig_2814 | 166 | 32 | - | hypothetical protein |
| contig_2814 | 183 | 668 | + | oxalate:formate antiporter, putative |
| contig_2815 | 540 | 238 | - | putative cytochrome P450 hydroxylase |
| contig_2815 | 751 | 617 | - | putative cytochrome P450 hydroxylase |
| contig_2817 | 365 | 207 | - | Aminoglycoside phosphotransferase |
| contig_2817 | 872 | 738 | - | hydrolase, haloacid dehalogenase-like family |
| contig_2817 | 1153 | 869 | - | hydrolase, haloacid dehalogenase-like family |
| contig_2818 | 362 | 249 | - | pXO1-11 |
| contig_282 | 1113 | 37 | - | Mg(2+) transport ATPase, P-type (EC 3.6.3.2) |
| contig_282 | 1990 | 1157 | - | Mg(2+) transport ATPase, P-type (EC 3.6.3.2) |
| contig_282 | 2286 | 1972 | - | Mg(2+) transport ATPase, P-type (EC 3.6.3.2) |
| contig_282 | 2649 | 2425 | - | Mg(2+) transport ATPase, P-type (EC 3.6.3.2) |
| contig_282 | 3596 | 3429 | - | FIG01225716: hypothetical protein |
| contig_2821 | 337 | 474 | + | FIG01225640: hypothetical protein |
| contig_2824 | 235 | 375 | + | Oligopeptide ABC transporter, periplasmic oligopeptide-binding protein OppA (TC 3.A.1.5.1) |
| contig_2825 | 403 | 182 | - | ABC transporter ATP-binding protein YvcR |
| contig_2825 | 686 | 375 | - | ABC transporter ATP-binding protein YvcR |
| contig_2825 | 855 | 724 | - | ABC transporter ATP-binding protein YvcR |
| contig_2829 | 587 | 162 | - | UDP-N-acetylmuramoylalanyl-D-glutamate--2,6-diaminopimelate ligase (EC 6.3.2.13) |
| contig_283 | 841 | 26 | - | FIG01227880: hypothetical protein |
| contig_283 | 1066 | 1194 | + | hypothetical protein |
| contig_283 | 1323 | 1502 | + | lysophospholipase-like family protein |
| contig_283 | 1714 | 2124 | + | lysophospholipase-like family protein |
| contig_283 | 2127 | 2276 | + | lysophospholipase-like family protein |
| contig_2831 | 157 | 291 | + | FIG01230237: hypothetical protein |
| contig_2835 | 152 | 15 | - | Transcriptional regulator, TrmB family |
| contig_2836 | 313 | 182 | - | sensor histidine kinase SrrB, putative |
| contig_2838 | 128 | 466 | + | FIG01227037: hypothetical protein |
| contig_2839 | 480 | 142 | - | FIG01228367: hypothetical protein |
| contig_284 | 20 | 463 | + | Membrane protein involved in the export of O-antigen, teichoic acid lipoteichoic acids |
| contig_284 | 450 | 1154 | + | Membrane protein involved in the export of O-antigen, teichoic acid lipoteichoic acids |
| contig_284 | 1799 | 1458 | - | Glycosyl transferase, group 2 family protein |
| contig_284 | 2238 | 1909 | - | Glycosyl transferase, group 2 family protein |
| contig_284 | 2414 | 2298 | - | hypothetical protein |
| contig_284 | 2644 | 2501 | - | hypothetical protein |
| contig_284 | 2705 | 3493 | + | ABC transporter ATP-binding protein uup |
| contig_284 | 3600 | 3782 | + | ABC transporter ATP-binding protein uup |
| contig_284 | 3745 | 4260 | + | ABC transporter ATP-binding protein uup |
| contig_284 | 4376 | 4885 | + | Probable poly(beta-D-mannuronate) O-acetylase (EC 2.3.1.-) |
| contig_284 | 5124 | 5753 | + | Probable poly(beta-D-mannuronate) O-acetylase (EC 2.3.1.-) |
| contig_284 | 5967 | 6440 | + | FIG01231430: hypothetical protein |
| contig_284 | 6635 | 6952 | + | FIG01231430: hypothetical protein |
| contig_284 | 7854 | 6982 | - | TetR family transcriptional regulator probably coupled to RND multidrug efflux transporter |
| contig_284 | 7997 | 8857 | + | RND multidrug efflux transporter; Acriflavin resistance protein |
| contig_284 | 8814 | 9356 | + | RND multidrug efflux transporter; Acriflavin resistance protein |
| contig_284 | 9370 | 9822 | + | RND multidrug efflux transporter; Acriflavin resistance protein |
| contig_284 | 9996 | 11123 | + | RND multidrug efflux transporter; Acriflavin resistance protein |
| contig_284 | 11355 | 11152 | - | Peptide methionine sulfoxide reductase MsrA (EC 1.8.4.11) / Peptide methionine sulfoxide reductase MsrB (EC 1.8.4.12) |
| contig_284 | 12118 | 11339 | - | Peptide methionine sulfoxide reductase MsrA (EC 1.8.4.11) / Peptide methionine sulfoxide reductase MsrB (EC 1.8.4.12) |
| contig_284 | 13162 | 12218 | - | membrane protein, putative |
| contig_284 | 14271 | 13579 | - | LrgA-associated membrane protein LrgB |
| contig_284 | 14737 | 14306 | - | Antiholin-like protein LrgA |
| contig_2840 | 621 | 487 | - | hypothetical protein |
| contig_2842 | 215 | 18 | - | FIG01227107: hypothetical protein |
| contig_2842 | 509 | 252 | - | FIG01227107: hypothetical protein |
| contig_2842 | 1087 | 620 | - | Nudix dNTPase DR1776 (EC 3.6.1.-) |
| contig_2843 | 347 | 520 | + | hypothetical protein |
| contig_2844 | 131 | 496 | + | Monofunctional biosynthetic peptidoglycan transglycosylase (EC 2.4.2.-) |
| contig_2846 | 704 | 267 | - | Lactoylglutathione lyase (EC 4.4.1.5) |
| contig_2846 | 1000 | 851 | - | Arsenical resistance operon repressor |
| contig_285 | 22 | 97 | + | tRNA-Val-TAC |
| contig_285 | 106 | 189 | + | tRNA-Tyr-GTA |
| contig_285 | 201 | 275 | + | tRNA-Gln-TTG |
| contig_2850 | 379 | 86 | - | FIG01227640: hypothetical protein |
| contig_2855 | 291 | 88 | - | multidrug resistance protein, putative |
| contig_2857 | 243 | 70 | - | acetyltransferase, GNAT family |
| contig_2857 | 364 | 236 | - | acetyltransferase, GNAT family |
| contig_2858 | 380 | 42 | - | 4-oxalocrotonate decarboxylase (EC 4.1.1.77) |
| contig_2858 | 609 | 403 | - | 4-oxalocrotonate decarboxylase (EC 4.1.1.77) |
| contig_2859 | 248 | 99 | - | hypothetical protein |
| contig_286 | 244 | 669 | + | Glutathione biosynthesis bifunctional protein gshF (EC 6.3.2.2)(EC 6.3.2.3) |
| contig_286 | 662 | 2515 | + | Glutathione biosynthesis bifunctional protein gshF (EC 6.3.2.2)(EC 6.3.2.3) |
| contig_286 | 3368 | 3781 | + | Beta-galactosidase (EC 3.2.1.23) |
| contig_286 | 4083 | 4778 | + | Spore germination protein GerKA |
| contig_286 | 4775 | 5785 | + | Spore germination protein xc. bacillus |
| contig_286 | 5739 | 5855 | + | Spore germination protein xc. bacillus |
| contig_2860 | 156 | 34 | - | hypothetical protein |
| contig_2860 | 260 | 132 | - | Sodium-dependent transporter |
| contig_2863 | 692 | 126 | - | pyridoxal phosphate-dependent deaminase, putative |
| contig_2863 | 915 | 751 | - | pyridoxal phosphate-dependent deaminase, putative |
| contig_2867 | 165 | 25 | - | Possible caffeoyl-CoA O-methyltransferase (EC 2.1.1.104) |
| contig_2867 | 472 | 585 | + | FIG01226713: hypothetical protein |
| contig_2870 | 444 | 235 | - | FMN-dependent NADH-azoreductase |
| contig_2870 | 664 | 494 | - | FMN-dependent NADH-azoreductase |
| contig_2870 | 927 | 655 | - | FMN-dependent NADH-azoreductase |
| contig_2872 | 125 | 307 | + | 5'-nucleotidase YjjG (EC 3.1.3.5) |
| contig_2874 | 286 | 131 | - | FIG01227864: hypothetical protein |
| contig_2876 | 36 | 404 | + | Branched-chain amino acid transport system carrier protein |
| contig_2878 | 29 | 271 | + | Aerobic C4-dicarboxylate transporter for fumarate, L-malate, D-malate, succunate, aspartate |
| contig_288 | 295 | 38 | - | Microbial collagenase (EC 3.4.24.3) |
| contig_288 | 803 | 645 | - | Microbial collagenase (EC 3.4.24.3) |
| contig_288 | 1833 | 883 | - | Microbial collagenase (EC 3.4.24.3) |
| contig_288 | 2080 | 3228 | + | Glycine betaine transporter OpuD |
| contig_288 | 3201 | 3632 | + | Glycine betaine transporter OpuD |
| contig_288 | 3636 | 3776 | + | hypothetical protein |
| contig_288 | 3823 | 4488 | + | ABC transporter membrane-spanning permease - glutamine transport |
| contig_288 | 4469 | 4651 | + | Amino acid ABC transporter, permease protein |
| contig_288 | 4675 | 5124 | + | Amino acid ABC transporter, permease protein |
| contig_288 | 5155 | 5922 | + | amino acid ABC transporter, ATP-binding protein |
| contig_288 | 5919 | 6782 | + | Lysine-arginine-ornithine-binding periplasmic protein precursor (TC 3.A.1.3.1) |
| contig_2880 | 240 | 121 | - | hypothetical protein |
| contig_2881 | 68 | 316 | + | FIG011739: membrane protein, putative |
| contig_2881 | 696 | 355 | - | FIG01227782: hypothetical protein |
| contig_2881 | 880 | 665 | - | FIG01227782: hypothetical protein |
| contig_2881 | 1101 | 964 | - | Aminopeptidase YpdF (MP-, MA-, MS-, AP-, NP- specific) |
| contig_2885 | 216 | 34 | - | hypothetical protein |
| contig_2886 | 185 | 69 | - | hypothetical protein |
| contig_289 | 105 | 30 | - | tRNA-Thr-TGT |
| contig_289 | 185 | 110 | - | tRNA-Val-TAC |
| contig_289 | 306 | 191 | - | 5S RNA |
| contig_2894 | 476 | 240 | - | acetyltransferase, GNAT family |
| contig_2894 | 619 | 506 | - | acetyltransferase, GNAT family |
| contig_2894 | 711 | 595 | - | COG1720: Uncharacterized conserved protein |
| contig_2895 | 23 | 214 | + | Substrate-specific component MtsA of methionine-regulated ECF transporter |
| contig_2896 | 6 | 221 | + | FIG002540: Haloacid dehalogenase-like hydrolase |
| contig_2896 | 184 | 321 | + | FIG002540: Haloacid dehalogenase-like hydrolase |
| contig_2897 | 135 | 425 | + | ABC transporter, ATP-binding protein |
| contig_29 | 170 | 1219 | + | FIG01239592: hypothetical protein |
| contig_29 | 1360 | 1737 | + | Phage protein |
| contig_29 | 1757 | 2245 | + | FIG01229157: hypothetical protein |
| contig_29 | 2326 | 2916 | + | possible DNA-binding protein |
| contig_29 | 3984 | 3019 | - | FIG01245139: hypothetical protein |
| contig_29 | 4462 | 4061 | - | FIG01232771: hypothetical protein |
| contig_29 | 5307 | 4477 | - | Chromosome (plasmid) partitioning protein ParA |
| contig_290 | 145 | 285 | + | FIG01239453: hypothetical protein |
| contig_290 | 531 | 1079 | + | CAAX amino terminal protease family protein |
| contig_2903 | 436 | 74 | - | hypothetical protein |
| contig_2905 | 498 | 292 | - | FIG01225662: hypothetical protein |
| contig_2909 | 283 | 113 | - | DUF1093 domain-containing protein |
| contig_291 | 230 | 33 | - | Mobile element protein |
| contig_291 | 376 | 1242 | + | Permease of the drug/metabolite transporter (DMT) superfamily |
| contig_2910 | 229 | 44 | - | FIG01228581: hypothetical protein |
| contig_2911 | 12 | 218 | + | Branched-chain amino acid transport system carrier protein |
| contig_2912 | 653 | 216 | - | acetyltransferase, GNAT family |
| contig_2913 | 503 | 249 | - | acetyltransferase, GNAT family |
| contig_2914 | 217 | 23 | - | hydrolase, alpha/beta fold family |
| contig_2915 | 285 | 124 | - | Oligopeptide ABC transporter, periplasmic oligopeptide-binding protein OppA (TC 3.A.1.5.1) |
| contig_2916 | 378 | 61 | - | 1-pyrroline-4-hydroxy-2-carboxylate deaminase (EC 3.5.4.22) |
| contig_2919 | 136 | 2 | - | Fumarate hydratase class I, aerobic (EC 4.2.1.2) |
| contig_292 | 454 | 59 | - | diguanylate cyclase/phosphodiesterase (GGDEF & EAL domains) with PAS/PAC sensor(s) |
| contig_2921 | 28 | 147 | + | Glycine betaine ABC transport system, ATP-binding protein OpuAA (EC 3.6.3.32) |
| contig_2921 | 128 | 385 | + | Glycine betaine ABC transport system, ATP-binding protein OpuAA (EC 3.6.3.32) |
| contig_2922 | 11 | 541 | + | putative cytochrome P450 hydroxylase |
| contig_2924 | 140 | 712 | + | possible permease |
| contig_2924 | 809 | 961 | + | possible permease |
| contig_293 | 1152 | 100 | - | Phosphopantetheine adenylyltransferase (EC 2.7.7.3) |
| contig_2933 | 834 | 106 | - | Transcriptional activator of acetoin dehydrogenase operon AcoR |
| contig_2933 | 1271 | 936 | - | Transcriptional activator of acetoin dehydrogenase operon AcoR |
| contig_2933 | 1605 | 1249 | - | Transcriptional activator of acetoin dehydrogenase operon AcoR |
| contig_2934 | 449 | 39 | - | Oligopeptide ABC transporter, periplasmic oligopeptide-binding protein OppA (TC 3.A.1.5.1) |
| contig_2935 | 183 | 64 | - | putative cytochrome P450 hydroxylase |
| contig_2935 | 633 | 328 | - | putative cytochrome P450 hydroxylase |
| contig_2936 | 689 | 570 | - | Cell division protein DivIC (FtsB), stabilizes FtsL against RasP cleavage |
| contig_2936 | 796 | 677 | - | Cell division protein DivIC (FtsB), stabilizes FtsL against RasP cleavage |
| contig_2937 | 150 | 10 | - | FIG01226135: hypothetical protein |
| contig_2938 | 222 | 10 | - | Cell envelope-associated transcriptional attenuator LytR-CpsA-Psr, subfamily F2 (as in PMID19099556) |
| contig_2939 | 81 | 527 | + | Outer membrane protein romA |
| contig_294 | 247 | 107 | - | FIG01226109: hypothetical protein |
| contig_294 | 446 | 282 | - | Dolichol-phosphate mannosyltransferase (EC 2.4.1.83) in lipid-linked oligosaccharide synthesis cluster |
| contig_294 | 1126 | 455 | - | Dolichol-phosphate mannosyltransferase (EC 2.4.1.83) in lipid-linked oligosaccharide synthesis cluster |
| contig_294 | 1424 | 1173 | - | Dolichol-phosphate mannosyltransferase (EC 2.4.1.83) in lipid-linked oligosaccharide synthesis cluster |
| contig_2942 | 444 | 265 | - | FIG01228880: hypothetical protein |
| contig_2943 | 381 | 40 | - | lipoprotein, putative |
| contig_2943 | 851 | 543 | - | lipoprotein, putative |
| contig_2948 | 282 | 133 | - | Aminotransferase, anthrose biosynthesis |
| contig_2948 | 497 | 381 | - | Aminotransferase, anthrose biosynthesis |
| contig_2949 | 179 | 51 | - | FIG01230354: hypothetical protein |
| contig_295 | 710 | 102 | - | Ferric iron ABC transporter, permease protein |
| contig_295 | 1092 | 736 | - | Ferric iron ABC transporter, permease protein |
| contig_295 | 1382 | 1215 | - | Ferric iron ABC transporter, permease protein |
| contig_295 | 2406 | 1456 | - | Ferric iron ABC transporter, ATP-binding protein |
| contig_295 | 2599 | 2486 | - | Ferric iron ABC transporter, iron-binding protein |
| contig_295 | 3271 | 2807 | - | Ferric iron ABC transporter, iron-binding protein |
| contig_295 | 3488 | 3231 | - | Ferric iron ABC transporter, iron-binding protein |
| contig_295 | 4010 | 4198 | + | FIG01229031: hypothetical protein |
| contig_295 | 4210 | 4365 | + | FIG01229031: hypothetical protein |
| contig_295 | 4873 | 4382 | - | Potassium uptake protein KtrB |
| contig_295 | 5159 | 4932 | - | sodium transporter family protein |
| contig_295 | 5646 | 5191 | - | acetyltransferase, GNAT family |
| contig_295 | 6063 | 5719 | - | FIG01115259: hypothetical protein |
| contig_295 | 6497 | 6066 | - | Zwittermicin A resistance protein ZmaR |
| contig_2957 | 882 | 181 | - | sensor histidine kinase SrrB, putative |
| contig_2959 | 28 | 300 | + | ABC transporter permease protein YvcS |
| contig_296 | 592 | 254 | - | Two-component sensor kinase SA14-24 |
| contig_296 | 1685 | 585 | - | Two-component sensor kinase SA14-24 |
| contig_296 | 2098 | 1682 | - | Two-component sensor kinase SA14-24 |
| contig_296 | 2810 | 2181 | - | Two-component response regulator SA14-24 |
| contig_2960 | 211 | 546 | + | membrane protein, putative |
| contig_2960 | 605 | 895 | + | membrane protein, putative |
| contig_2961 | 424 | 227 | - | ABC transporter ATP-binding protein YvcR |
| contig_2962 | 31 | 345 | + | bacitracin transport permease protein bcrc |
| contig_2967 | 325 | 212 | - | crotonyl-CoA reductase |
| contig_2967 | 460 | 347 | - | crotonyl-CoA reductase |
| contig_2967 | 759 | 646 | - | crotonyl-CoA reductase |
| contig_2968 | 77 | 238 | + | Endopeptidase spore protease Gpr (EC 3.4.24.78) |
| contig_2968 | 195 | 431 | + | Endopeptidase spore protease Gpr (EC 3.4.24.78) |
| contig_2969 | 154 | 471 | + | FIG01226300: hypothetical protein |
| contig_297 | 477 | 16 | - | COG1272: Predicted membrane protein hemolysin III homolog |
| contig_297 | 693 | 806 | + | hypothetical protein |
| contig_297 | 1643 | 1419 | - | Chromosome (plasmid) partitioning protein ParB |
| contig_297 | 2148 | 1705 | - | Chromosome (plasmid) partitioning protein ParB |
| contig_297 | 2350 | 2141 | - | Chromosome (plasmid) partitioning protein ParA |
| contig_297 | 2903 | 2307 | - | Chromosome (plasmid) partitioning protein ParA |
| contig_297 | 3964 | 3092 | - | Chromosome (plasmid) partitioning protein ParB |
| contig_297 | 4788 | 4069 | - | rRNA small subunit 7-methylguanosine (m7G) methyltransferase GidB |
| contig_297 | 6699 | 4810 | - | tRNA uridine 5-carboxymethylaminomethyl modification enzyme GidA |
| contig_297 | 7570 | 6746 | - | GTPase and tRNA-U34 5-formylation enzyme TrmE |
| contig_297 | 8123 | 7653 | - | GTPase and tRNA-U34 5-formylation enzyme TrmE |
| contig_2970 | 603 | 436 | - | FIG01226124: hypothetical protein |
| contig_2972 | 492 | 178 | - | oxalate:formate antiporter, putative |
| contig_2975 | 919 | 605 | - | FIG01225986: hypothetical protein |
| contig_2977 | 188 | 9 | - | Microbial collagenase (EC 3.4.24.3) |
| contig_2978 | 232 | 489 | + | transporter, putative |
| contig_2978 | 459 | 713 | + | transporter, putative |
| contig_2978 | 683 | 859 | + | transporter, putative |
| contig_2979 | 431 | 75 | - | Glycerate kinase (EC 2.7.1.31) |
| contig_298 | 97 | 25 | - | tRNA-Ala-TGC |
| contig_298 | 179 | 108 | - | tRNA-Gly-GCC |
| contig_2980 | 473 | 57 | - | ATP synthase protein I |
| contig_2980 | 769 | 527 | - | Magnesium and cobalt efflux protein CorC |
| contig_2983 | 464 | 207 | - | Alpha/beta hydrolase |
| contig_2983 | 546 | 415 | - | Alpha/beta hydrolase |
| contig_2984 | 434 | 201 | - | hypothetical protein |
| contig_2986 | 66 | 272 | + | PTS system, glucose-specific IIC component (EC 2.7.1.69) / PTS system, glucose-specific IIB component (EC 2.7.1.69) / PTS system, glucose-specific IIA component (EC 2.7.1.69) |
| contig_2989 | 297 | 569 | + | SpoVS-related protein, type 1 |
| contig_299 | 98 | 23 | - | tRNA-Ala-TGC |
| contig_299 | 181 | 108 | - | tRNA-Pro-TGG |
| contig_299 | 263 | 190 | - | tRNA-Arg-ACG |
| contig_299 | 355 | 267 | - | tRNA-Leu-TAA |
| contig_299 | 446 | 372 | - | tRNA-Gly-GCC |
| contig_2990 | 583 | 443 | - | peptidase, M23/M37 family |
| contig_2991 | 325 | 95 | - | Antibiotic biosynthesis monooxygenase domain-containing protein |
| contig_2991 | 602 | 414 | - | Macrolide efflux protein |
| contig_2992 | 159 | 7 | - | acetyltransferase, GNAT family family |
| contig_2995 | 292 | 137 | - | Transcriptional regulator, DeoR family |
| contig_2995 | 599 | 315 | - | Transcriptional regulator, DeoR family |
| contig_2997 | 490 | 179 | - | DltD protein |
| contig_2999 | 103 | 231 | + | CAAX amino terminal protease family family |
| contig_3 | 552 | 986 | + | 5'-nucleotidase family protein in cluster with NagD-like phosphatase |
| contig_3 | 979 | 1833 | + | 5'-nucleotidase family protein in cluster with NagD-like phosphatase |
| contig_3 | 1899 | 2201 | + | DUF1805 domain-containing protein |
| contig_3 | 2325 | 2987 | + | FIG01225377: hypothetical protein |
| contig_3 | 3986 | 3024 | - | Cell wall endopeptidase, family M23/M37 |
| contig_3 | 4198 | 5094 | + | Lipoate synthase |
| contig_3 | 5594 | 5115 | - | FIG042921: similarity to aminoacyl-tRNA editing enzymes YbaK, ProX |
| contig_3 | 6154 | 5669 | - | Phosphoglycerate mutase family 2 |
| contig_3 | 6357 | 6800 | + | FIG01228712: hypothetical protein |
| contig_3 | 7598 | 6825 | - | membrane protein, putative |
| contig_3 | 7741 | 8007 | + | transcriptional activator tipA |
| contig_3 | 7997 | 8332 | + | transcriptional activator tipA |
| contig_3 | 8295 | 8474 | + | transcriptional activator tipA |
| contig_3 | 9091 | 8513 | - | Phosphonate ABC transporter phosphate-binding periplasmic component (TC 3.A.1.9.1) |
| contig_3 | 9137 | 9250 | + | hypothetical protein |
| contig_3 | 9210 | 9437 | + | Hypothetical DUF1027 domain protein |
| contig_3 | 9397 | 9513 | + | Hypothetical DUF1027 domain protein |
| contig_3 | 10047 | 9550 | - | FIG01227044: hypothetical protein |
| contig_3 | 10688 | 10422 | - | hypothetical protein BH3432 |
| contig_3 | 11679 | 10756 | - | Fructose-1,6-bisphosphatase, GlpX type (EC 3.1.3.11) |
| contig_3 | 11827 | 12267 | + | BH3430 unknown conserved protein |
| contig_3 | 12339 | 12971 | + | Transcriptional regulator, DeoR family |
| contig_3 | 13082 | 13846 | + | Hypothetical NagD-like phosphatase |
| contig_30 | 1261 | 290 | - | Formiminoglutamase (EC 3.5.3.8) |
| contig_30 | 1842 | 1240 | - | Imidazolonepropionase (EC 3.5.2.7) |
| contig_30 | 2512 | 1829 | - | Imidazolonepropionase (EC 3.5.2.7) |
| contig_30 | 2672 | 2526 | - | Urocanate hydratase (EC 4.2.1.49) |
| contig_30 | 3261 | 2641 | - | Urocanate hydratase (EC 4.2.1.49) |
| contig_30 | 3669 | 3475 | - | Urocanate hydratase (EC 4.2.1.49) |
| contig_30 | 4127 | 3705 | - | Urocanate hydratase (EC 4.2.1.49) |
| contig_30 | 5542 | 4208 | - | Histidine ammonia-lyase (EC 4.3.1.3) |
| contig_30 | 5733 | 5536 | - | Histidine ammonia-lyase (EC 4.3.1.3) |
| contig_30 | 6152 | 5838 | - | Hut operon positive regulatory protein |
| contig_30 | 6279 | 6109 | - | Hut operon positive regulatory protein |
| contig_300 | 446 | 249 | - | Phosphate ABC transporter, periplasmic phosphate-binding protein PstS (TC 3.A.1.7.1) |
| contig_300 | 873 | 436 | - | Phosphate ABC transporter, periplasmic phosphate-binding protein PstS (TC 3.A.1.7.1) |
| contig_300 | 1109 | 903 | - | Phosphate ABC transporter, periplasmic phosphate-binding protein PstS (TC 3.A.1.7.1) |
| contig_300 | 1669 | 1926 | + | Ferrous iron transport protein A |
| contig_300 | 1923 | 2573 | + | Ferrous iron transport protein B |
| contig_300 | 2625 | 4025 | + | Ferrous iron transport protein B |
| contig_300 | 4225 | 4088 | - | hypothetical protein |
| contig_300 | 4597 | 4286 | - | Spore germination protein GerLC |
| contig_300 | 4874 | 4650 | - | Spore germination protein GerLC |
| contig_300 | 5046 | 4867 | - | Spore germination protein GerLC |
| contig_300 | 5407 | 5117 | - | Spore germination protein GerLC |
| contig_300 | 5774 | 5391 | - | Spore germination protein GerLB |
| contig_300 | 5886 | 5740 | - | Spore germination protein GerLB |
| contig_300 | 6085 | 5879 | - | Spore germination protein GerLB |
| contig_300 | 6521 | 6162 | - | Spore germination protein GerLB |
| contig_300 | 7484 | 6522 | - | Spore germination protein GerLA |
| contig_300 | 7647 | 7522 | - | Spore germination protein GerLA |
| contig_300 | 7840 | 7640 | - | Spore germination protein GerLA |
| contig_300 | 9016 | 8105 | - | Acetate permease ActP (cation/acetate symporter) |
| contig_300 | 9466 | 9278 | - | Acetate permease ActP (cation/acetate symporter) |
| contig_3000 | 411 | 208 | - | methyl-accepting chemotaxis protein |
| contig_3002 | 208 | 59 | - | Thioredoxin |
| contig_3003 | 322 | 86 | - | FIG01225212: hypothetical protein |
| contig_3005 | 194 | 403 | + | FIG01226859: hypothetical protein |
| contig_3007 | 133 | 579 | + | Undecaprenyl-diphosphatase (EC 3.6.1.27) |
| contig_3008 | 63 | 239 | + | drug transport protein |
| contig_301 | 663 | 1031 | + | Gluconolactonase (EC 3.1.1.17) |
| contig_301 | 1045 | 1566 | + | Gluconolactonase (EC 3.1.1.17) |
| contig_3010 | 259 | 98 | - | Stage 0 sporulation regulatory protein |
| contig_3010 | 326 | 213 | - | Stage 0 sporulation regulatory protein |
| contig_3013 | 196 | 324 | + | NAD-specific glutamate dehydrogenase (EC 1.4.1.2) |
| contig_3016 | 335 | 90 | - | Potassium efflux system KefA protein / Small-conductance mechanosensitive channel |
| contig_3017 | 384 | 199 | - | Manganese-dependent inorganic pyrophosphatase (EC 3.6.1.1) |
| contig_3018 | 303 | 91 | - | Transcription state regulatory protein abrB |
| contig_3019 | 427 | 68 | - | Ferric iron ABC transporter, ATP-binding protein |
| contig_3020 | 141 | 28 | - | Uncharacterized protein conserved in bacteria |
| contig_3022 | 504 | 385 | - | hypothetical protein |
| contig_3023 | 512 | 357 | - | Exosporium protein E |
| contig_3025 | 332 | 63 | - | hypothetical protein |
| contig_3027 | 42 | 380 | + | Ubiquinone/menaquinone biosynthesis methyltransferase UbiE (EC 2.1.1.-) |
| contig_3029 | 106 | 315 | + | Magnesium and cobalt transport protein CorA |
| contig_303 | 48 | 167 | + | membrane protein, putative |
| contig_303 | 375 | 223 | - | L-lactate dehydrogenase (EC 1.1.1.27) |
| contig_303 | 1104 | 571 | - | L-lactate dehydrogenase (EC 1.1.1.27) |
| contig_3032 | 113 | 229 | + | transporter, putative |
| contig_3032 | 687 | 565 | - | hypothetical protein |
| contig_3032 | 611 | 727 | + | FIG01227646: hypothetical protein |
| contig_3033 | 122 | 514 | + | Glutamate-aspartate carrier protein |
| contig_304 | 55 | 255 | + | Subtilase family domain protein |
| contig_304 | 408 | 797 | + | Subtilase family domain protein |
| contig_304 | 766 | 1017 | + | Subtilase family domain protein |
| contig_304 | 2232 | 1357 | - | L-lactate dehydrogenase (EC 1.1.1.27) |
| contig_3040 | 210 | 61 | - | UvrC-like protein |
| contig_3041 | 224 | 108 | - | Threonine dehydrogenase and related Zn-dependent dehydrogenases |
| contig_3044 | 494 | 165 | - | Ferredoxin--sulfite reductase, bacillial type (EC 1.8.7.1) |
| contig_3045 | 68 | 223 | + | Zinc uptake regulation protein ZUR |
| contig_3047 | 181 | 2 | - | Branched-chain amino acid transport system carrier protein |
| contig_3048 | 239 | 24 | - | methylase |
| contig_305 | 730 | 326 | - | TraG/TraD family protein |
| contig_305 | 2400 | 748 | - | TraG/TraD family protein |
| contig_305 | 3625 | 2384 | - | TraG/TraD family protein |
| contig_305 | 4830 | 4219 | - | conserved hypothetical Bacillus plasmid protein |
| contig_305 | 5506 | 4838 | - | conserved hypothetical Bacillus plasmid protein |
| contig_305 | 7258 | 6158 | - | tubulin/FtsZ family, GTPase domain protein |
| contig_305 | 7634 | 7401 | - | FIG01226898: hypothetical protein |
| contig_305 | 8208 | 8348 | + | FIG01234934: hypothetical protein |
| contig_3051 | 103 | 231 | + | Sodium/glycine symporter GlyP |
| contig_3052 | 68 | 352 | + | membrane protein, putative |
| contig_3055 | 72 | 209 | + | Tellurium resistance protein TerD |
| contig_3056 | 221 | 93 | - | oxidoreductase, Gfo/Idh/MocA family |
| contig_3057 | 28 | 441 | + | F0F1 ATP synthase subunit alpha |
| contig_3057 | 452 | 619 | + | F0F1 ATP synthase subunit alpha |
| contig_3058 | 1 | 378 | + | Intramembrane protease RasP/YluC, implicated in cell division based on FtsL cleavage |
| contig_306 | 22 | 183 | + | hypothetical protein |
| contig_306 | 562 | 1095 | + | hypothetical protein |
| contig_306 | 1061 | 2464 | + | hypothetical protein |
| contig_306 | 2468 | 3160 | + | protein export membrane protein |
| contig_306 | 4157 | 3249 | - | Ubiquinone biosynthesis monooxygenase UbiB |
| contig_306 | 4924 | 4139 | - | Ubiquinone biosynthesis monooxygenase UbiB |
| contig_306 | 5253 | 4921 | - | hypothetical protein |
| contig_306 | 5578 | 5285 | - | UPF0028 protein YchK |
| contig_306 | 6063 | 5674 | - | UPF0028 protein YchK |
| contig_306 | 6427 | 6555 | + | hypothetical protein |
| contig_306 | 6512 | 7060 | + | Sulfite reductase [NADPH] flavoprotein alpha-component (EC 1.8.1.2) |
| contig_3061 | 11 | 631 | + | hypothetical protein |
| contig_3062 | 165 | 407 | + | Probable low-affinity inorganic phosphate transporter |
| contig_3063 | 382 | 176 | - | Copper(I) chaperone CopZ |
| contig_3063 | 701 | 585 | - | Repressor CsoR of the copZA operon |
| contig_3064 | 619 | 395 | - | ABC transporter, permease protein, putative |
| contig_3068 | 97 | 375 | + | ABC transporter permease protein YvcS |
| contig_307 | 2082 | 451 | - | prophage LambdaBa01, minor structural protein |
| contig_307 | 3560 | 2082 | - | Phage tail protein |
| contig_307 | 4099 | 3602 | - | Tail length tape measure protein |
| contig_307 | 4113 | 4538 | + | hypothetical protein |
| contig_307 | 5326 | 4556 | - | Phage tail length tape-measure protein |
| contig_307 | 6281 | 5442 | - | Phage tail length tape-measure protein |
| contig_307 | 6738 | 6301 | - | Phage tail length tape-measure protein |
| contig_307 | 6925 | 6809 | - | hypothetical protein |
| contig_307 | 7432 | 6998 | - | Phage tail length tape-measure protein |
| contig_307 | 8044 | 7700 | - | Phage protein |
| contig_307 | 8649 | 8086 | - | Phage major tail protein |
| contig_307 | 8967 | 8650 | - | Phage protein |
| contig_307 | 9325 | 8972 | - | Phage protein |
| contig_307 | 9599 | 9318 | - | Phage protein |
| contig_307 | 9780 | 9649 | - | hypothetical protein |
| contig_307 | 10087 | 9953 | - | hypothetical protein |
| contig_307 | 11132 | 10179 | - | Phage capsid protein |
| contig_307 | 11682 | 11125 | - | Phage head maturation protease |
| contig_307 | 12146 | 11691 | - | Phage portal protein |
| contig_307 | 12862 | 12152 | - | Phage portal protein |
| contig_307 | 14509 | 12884 | - | Phage terminase, large subunit |
| contig_307 | 14601 | 14485 | - | Phage terminase, large subunit |
| contig_307 | 14985 | 14635 | - | Phage terminase, small subunit |
| contig_307 | 15336 | 15100 | - | Phage protein |
| contig_307 | 15994 | 15776 | - | hypothetical protein |
| contig_307 | 16411 | 16124 | - | conserved phage protein |
| contig_307 | 16532 | 16413 | - | conserved phage protein |
| contig_307 | 16857 | 16669 | - | hypothetical protein |
| contig_307 | 16976 | 16854 | - | hypothetical protein |
| contig_3072 | 607 | 212 | - | Flavohemoprotein (Hemoglobin-like protein) (Flavohemoglobin) (Nitric oxide dioxygenase) (EC 1.14.12.17) |
| contig_3073 | 216 | 1 | - | Chloramphenicol acetyltransferase (EC 2.3.1.28) |
| contig_3076 | 7 | 123 | + | 6-aminohexanoate-dimer hydrolase (EC 3.5.1.46) |
| contig_3076 | 476 | 601 | + | membrane protein, putative |
| contig_308 | 677 | 105 | - | Dihydroxyacetone kinase family protein |
| contig_308 | 879 | 703 | - | Dihydroxyacetone kinase family protein |
| contig_308 | 1625 | 1065 | - | Dihydroxyacetone kinase family protein |
| contig_308 | 2012 | 1749 | - | FIG001802: Putative alkaline-shock protein |
| contig_308 | 2391 | 2579 | + | LSU ribosomal protein L28p |
| contig_3080 | 366 | 109 | - | Glyoxylase family protein |
| contig_3081 | 302 | 162 | - | Arginine/ornithine antiporter ArcD |
| contig_3082 | 257 | 30 | - | Pyrrolidone-carboxylate peptidase (EC 3.4.19.3) |
| contig_3084 | 546 | 199 | - | FIG01227230: hypothetical protein |
| contig_3084 | 739 | 578 | - | FIG01225567: hypothetical protein |
| contig_3085 | 66 | 461 | + | Rrf2 family transcriptional regulator |
| contig_3087 | 271 | 59 | - | hydrolase, alpha/beta fold family |
| contig_3088 | 178 | 47 | - | FIG01227030: hypothetical protein |
| contig_3089 | 435 | 298 | - | hypothetical protein |
| contig_309 | 384 | 312 | - | tRNA-Phe-GAA |
| contig_309 | 502 | 427 | - | tRNA-Asp-GTC |
| contig_309 | 767 | 1156 | + | Vancomycin B-type resistance protein VanW |
| contig_309 | 1203 | 1913 | + | Vancomycin B-type resistance protein VanW |
| contig_309 | 2355 | 1999 | - | FIG01227571: hypothetical protein |
| contig_309 | 2889 | 2371 | - | Phosphonate ABC transporter phosphate-binding periplasmic component (TC 3.A.1.9.1) |
| contig_309 | 3268 | 3846 | + | membrane protein, putative |
| contig_309 | 4903 | 3878 | - | neutral protease |
| contig_309 | 5318 | 4950 | - | neutral protease |
| contig_309 | 5580 | 5359 | - | neutral protease |
| contig_309 | 5879 | 5751 | - | hypothetical protein |
| contig_309 | 6136 | 5876 | - | transcriptional regulator/TPR domain protein |
| contig_309 | 7095 | 6133 | - | transcriptional regulator/TPR domain protein |
| contig_309 | 7356 | 8408 | + | Nicotinate phosphoribosyltransferase (EC 2.4.2.11) |
| contig_3090 | 313 | 71 | - | FIG011741: hypothetical protein |
| contig_3093 | 280 | 420 | + | Aspartokinase (EC 2.7.2.4) |
| contig_3095 | 524 | 246 | - | FIG01225916: hypothetical protein |
| contig_3099 | 411 | 187 | - | ABC transporter, permease protein |
| contig_31 | 51 | 347 | + | sodium-dependent transporter, putative |
| contig_31 | 331 | 1023 | + | sodium-dependent transporter, putative |
| contig_31 | 1016 | 1483 | + | sodium-dependent transporter, putative |
| contig_31 | 1684 | 1887 | + | ABC transporter, permease protein, putative |
| contig_31 | 1892 | 2233 | + | ABC transporter, permease protein, putative |
| contig_310 | 584 | 69 | - | FIG01228550: hypothetical protein |
| contig_310 | 1116 | 682 | - | FIG01228550: hypothetical protein |
| contig_3104 | 76 | 426 | + | ABC transporter permease protein |
| contig_3104 | 383 | 541 | + | ABC transporter permease protein |
| contig_3106 | 472 | 71 | - | drug transport protein |
| contig_3106 | 696 | 469 | - | drug transport protein |
| contig_3107 | 3 | 152 | + | membrane protein, putative |
| contig_3107 | 163 | 327 | + | membrane protein, putative |
| contig_3108 | 619 | 744 | + | hypothetical protein |
| contig_311 | 245 | 90 | - | L-alanyl-gamma-D-glutamyl-L-diamino acid endopeptidase |
| contig_311 | 621 | 217 | - | L-alanyl-gamma-D-glutamyl-L-diamino acid endopeptidase |
| contig_311 | 832 | 590 | - | L-alanyl-gamma-D-glutamyl-L-diamino acid endopeptidase |
| contig_311 | 1141 | 872 | - | L-alanine-DL-glutamate epimerase |
| contig_311 | 1310 | 1197 | - | L-alanine-DL-glutamate epimerase |
| contig_3113 | 146 | 451 | + | Transmembrane component MtsC of energizing module of methionine-regulated ECF transporter |
| contig_3119 | 423 | 136 | - | Transcriptional regulator, MarR family |
| contig_312 | 742 | 56 | - | D-alanyl-D-alanine carboxypeptidase (EC 3.4.16.4) |
| contig_3121 | 570 | 457 | - | hypothetical protein |
| contig_3128 | 348 | 82 | - | Integral membrane protein |
| contig_3128 | 573 | 391 | - | Integral membrane protein |
| contig_313 | 382 | 254 | - | hypothetical protein |
| contig_313 | 420 | 794 | + | Arginine/ornithine antiporter ArcD |
| contig_313 | 807 | 1307 | + | Arginine/ornithine antiporter ArcD |
| contig_313 | 1489 | 1373 | - | FIG01226075: hypothetical protein |
| contig_313 | 1676 | 1533 | - | hypothetical protein |
| contig_313 | 2591 | 1782 | - | FIG002540: Haloacid dehalogenase-like hydrolase |
| contig_313 | 3069 | 3266 | + | FIG01227868: hypothetical protein |
| contig_313 | 3478 | 3684 | + | ClpB protein |
| contig_313 | 3665 | 4927 | + | ClpB protein |
| contig_313 | 5045 | 5332 | + | ClpB protein |
| contig_313 | 5385 | 5588 | + | ClpB protein |
| contig_313 | 5578 | 6084 | + | ClpB protein |
| contig_313 | 6304 | 6122 | - | Putative uncharacterized protein GBAA1178 |
| contig_313 | 6726 | 6932 | + | HMP-PP hydrolase (pyridoxal phosphatase) Cof, detected in genetic screen for thiamin metabolic genes (PMID:15292217) |
| contig_3130 | 408 | 244 | - | Ferric uptake regulation protein FUR |
| contig_3132 | 293 | 180 | - | hypothetical protein |
| contig_3133 | 217 | 2 | - | FIG01226109: hypothetical protein |
| contig_3133 | 471 | 334 | - | FIG01226109: hypothetical protein |
| contig_3134 | 507 | 28 | - | ABC transporter, permease protein, putative |
| contig_3137 | 491 | 375 | - | Lipase (EC 3.1.1.3) |
| contig_3137 | 691 | 488 | - | Lipase (EC 3.1.1.3) |
| contig_314 | 1020 | 574 | - | LSU ribosomal protein L9p |
| contig_314 | 1285 | 1097 | - | Phosphoesterase, DHH family protein |
| contig_314 | 2306 | 1308 | - | Phosphoesterase, DHH family protein |
| contig_314 | 2746 | 2336 | - | Phosphoesterase, DHH family protein |
| contig_314 | 3766 | 3209 | - | FIG003573: hypothetical protein |
| contig_314 | 3974 | 3738 | - | FIG003573: hypothetical protein |
| contig_314 | 4327 | 4094 | - | SSU ribosomal protein S18p @ SSU ribosomal protein S18p, zinc-independent |
| contig_314 | 4882 | 4373 | - | Single-stranded DNA-binding protein |
| contig_314 | 5202 | 4912 | - | SSU ribosomal protein S6p |
| contig_314 | 5941 | 5468 | - | GTP-binding and nucleic acid-binding protein YchF |
| contig_314 | 6503 | 6297 | - | GTP-binding and nucleic acid-binding protein YchF |
| contig_314 | 6815 | 6618 | - | FIG001891: protein involved in chromosome partitioning |
| contig_314 | 7288 | 6836 | - | Potassium efflux system KefA protein / Small-conductance mechanosensitive channel |
| contig_314 | 7719 | 7300 | - | Potassium efflux system KefA protein / Small-conductance mechanosensitive channel |
| contig_314 | 8032 | 8577 | + | Spore protease GPR related protein |
| contig_3140 | 120 | 266 | + | transporter, EamA family |
| contig_3141 | 592 | 272 | - | Negative regulator of genetic competence MecA |
| contig_3142 | 182 | 385 | + | Transcriptional regulator, ArsR family |
| contig_3143 | 142 | 2 | - | Glycosyltransferase (EC 2.4.1.-) |
| contig_3146 | 37 | 261 | + | Ribose 5-phosphate isomerase A (EC 5.3.1.6) |
| contig_3147 | 168 | 49 | - | FIG01229291: hypothetical protein |
| contig_3147 | 675 | 821 | + | hypothetical protein |
| contig_315 | 750 | 337 | - | RNA polymerase sporulation specific sigma factor SigF |
| contig_315 | 1203 | 763 | - | Anti-sigma F factor (EC 2.7.11.1) |
| contig_315 | 1554 | 1204 | - | Anti-sigma F factor antagonist (spoIIAA-2); Anti-sigma B factor antagonist RsbV |
| contig_315 | 2915 | 1725 | - | D-alanyl-D-alanine carboxypeptidase (EC 3.4.16.4) |
| contig_315 | 2881 | 2997 | + | hypothetical protein |
| contig_315 | 3181 | 3504 | + | Transcriptional regulator, GntR family |
| contig_315 | 3470 | 3670 | + | ABC transporter, ATP-binding protein |
| contig_315 | 3862 | 4077 | + | ABC transporter, ATP-binding protein |
| contig_315 | 4246 | 4362 | + | ABC transporter, ATP-binding protein |
| contig_315 | 4352 | 5440 | + | acetoin transport permease protein |
| contig_3151 | 268 | 152 | - | FIG01225574: hypothetical protein |
| contig_3152 | 120 | 245 | + | hypothetical protein |
| contig_3153 | 391 | 188 | - | HAMP domain protein |
| contig_3156 | 209 | 54 | - | 3-oxoacyl-[acyl-carrier protein] reductase (EC 1.1.1.100) |
| contig_3157 | 332 | 141 | - | hypothetical protein |
| contig_3158 | 147 | 28 | - | hypothetical protein |
| contig_3158 | 331 | 134 | - | hypothetical protein |
| contig_316 | 144 | 965 | + | ABC transporter, permease protein |
| contig_3162 | 308 | 36 | - | Acetyl-CoA:acetoacetyl-CoA transferase, alpha subunit (EC 2.8.3.8) |
| contig_3164 | 226 | 35 | - | FIG01229538: hypothetical protein |
| contig_3165 | 114 | 242 | + | FIG01226779: hypothetical protein |
| contig_3166 | 353 | 234 | - | COG1720: Uncharacterized conserved protein |
| contig_3169 | 152 | 283 | + | DNA polymerase III beta subunit (EC 2.7.7.7) |
| contig_317 | 257 | 87 | - | Isochorismatase (EC 3.3.2.1) |
| contig_317 | 654 | 244 | - | Isochorismatase (EC 3.3.2.1) |
| contig_317 | 1292 | 1672 | + | FIG00628819: hypothetical protein |
| contig_3171 | 233 | 42 | - | Lipoteichoic acid synthase LtaS Type Ia |
| contig_3172 | 165 | 344 | + | FIG01225951: hypothetical protein |
| contig_3173 | 1 | 159 | + | TraG/TraD family protein |
| contig_3177 | 127 | 312 | + | FIG01226999: hypothetical protein |
| contig_3181 | 201 | 55 | - | Signal transduction histidine kinase |
| contig_3182 | 370 | 546 | + | Stage IV sporulation protein |
| contig_3184 | 266 | 9 | - | Protein LiaI |
| contig_3187 | 63 | 314 | + | Malate Na(+) symporter |
| contig_3189 | 285 | 142 | - | Ribosomal protein L5 domain protein |
| contig_3189 | 410 | 282 | - | Ribosomal protein L5 domain protein |
| contig_319 | 650 | 132 | - | Hydroxyacylglutathione hydrolase (EC 3.1.2.6) |
| contig_319 | 817 | 1656 | + | Pyrroline-5-carboxylate reductase (EC 1.5.1.2) |
| contig_319 | 2192 | 1707 | - | L-serine dehydratase, alpha subunit (EC 4.3.1.17) |
| contig_319 | 2586 | 2176 | - | L-serine dehydratase, alpha subunit (EC 4.3.1.17) |
| contig_319 | 3264 | 2605 | - | L-serine dehydratase, beta subunit (EC 4.3.1.17) |
| contig_3193 | 322 | 38 | - | FIG01237696: hypothetical protein |
| contig_3194 | 121 | 282 | + | FIG01226494: hypothetical protein |
| contig_3194 | 483 | 770 | + | Alkaline phosphatase like protein |
| contig_3194 | 771 | 956 | + | DedA family protein |
| contig_3195 | 271 | 86 | - | hypothetical protein |
| contig_3195 | 271 | 408 | + | FIG01229056: hypothetical protein |
| contig_3195 | 488 | 622 | + | FIG01229056: hypothetical protein |
| contig_3195 | 852 | 682 | - | Possible serine/threonine specific protein phosphatase (EC 3.1.3.16) |
| contig_3196 | 320 | 168 | - | internalin, putative |
| contig_3199 | 144 | 272 | + | hypothetical protein |
| contig_32 | 130 | 483 | + | polysaccharide deacetylase, putative |
| contig_32 | 518 | 790 | + | Polysaccharide deacetylase (EC 3.5.1.41) |
| contig_32 | 791 | 949 | + | Polysaccharide deacetylase (EC 3.5.1.41) |
| contig_320 | 469 | 23 | - | FIG004556: membrane metalloprotease |
| contig_320 | 619 | 473 | - | FIG01238343: hypothetical protein |
| contig_320 | 582 | 767 | + | 4-oxalocrotonate tautomerase (EC 5.3.2.-); Xylose transport system permease protein xylH |
| contig_320 | 1587 | 796 | - | dNTP triphosphohydrolase, putative |
| contig_320 | 1972 | 1598 | - | dNTP triphosphohydrolase, putative |
| contig_320 | 2111 | 1932 | - | dNTP triphosphohydrolase, putative |
| contig_320 | 2380 | 2258 | - | iron compound ABC transporter, iron compound-binding protein |
| contig_320 | 3207 | 2497 | - | Iron(III) dicitrate transport system, periplasmic iron-binding protein FecB (TC 3.A.1.14.1) |
| contig_320 | 4215 | 3403 | - | Ferrichrome transport ATP-binding protein FhuC (TC 3.A.1.14.3) |
| contig_320 | 4322 | 4203 | - | ABC-type Fe3+-siderophore transport system, permease 2 component |
| contig_320 | 4729 | 4334 | - | ABC-type Fe3+-siderophore transport system, permease 2 component |
| contig_320 | 5258 | 4743 | - | ABC-type Fe3+-siderophore transport system, permease 2 component |
| contig_320 | 6187 | 5255 | - | ABC-type Fe3+-siderophore transport system, permease component |
| contig_320 | 6311 | 6156 | - | ABC-type Fe3+-siderophore transport system, permease component |
| contig_3200 | 198 | 67 | - | Autoinducer 2 (AI-2) kinase LsrK (EC 2.7.1.-) |
| contig_3202 | 129 | 7 | - | Glyoxalase family protein |
| contig_3203 | 305 | 141 | - | Tripeptide aminopeptidase (EC 3.4.11.4) |
| contig_3206 | 520 | 338 | - | HigA protein (antitoxin to HigB) |
| contig_3207 | 373 | 567 | + | ABC transporter, permease protein, putative |
| contig_3208 | 280 | 101 | - | Flagellar biosynthesis protein FlhF |
| contig_3208 | 380 | 249 | - | hypothetical protein |
| contig_321 | 245 | 1141 | + | 4-hydroxy-3-methylbut-2-enyl diphosphate reductase (EC 1.17.1.2) |
| contig_321 | 1874 | 1182 | - | FIG146262: hypothetical protein |
| contig_321 | 2305 | 1835 | - | FIG146262: hypothetical protein |
| contig_321 | 2481 | 2302 | - | Putative tRNA-m1A22 methylase |
| contig_321 | 3012 | 2686 | - | Putative tRNA-m1A22 methylase |
| contig_321 | 3540 | 3283 | - | Membrane-attached cytochrome c550 |
| contig_321 | 4380 | 4108 | - | RNA polymerase sigma factor RpoD |
| contig_321 | 4936 | 4343 | - | RNA polymerase sigma factor RpoD |
| contig_321 | 5073 | 4933 | - | RNA polymerase sigma factor RpoD |
| contig_321 | 6096 | 5338 | - | DNA primase (EC 2.7.7.-) |
| contig_321 | 6480 | 6115 | - | DNA primase (EC 2.7.7.-) |
| contig_321 | 6718 | 6473 | - | DNA primase (EC 2.7.7.-) |
| contig_321 | 6942 | 6718 | - | DNA primase (EC 2.7.7.-) |
| contig_3210 | 231 | 103 | - | FIG01229170: hypothetical protein |
| contig_3213 | 151 | 35 | - | Transcriptional regulator, MerR family |
| contig_3213 | 239 | 111 | - | Transcriptional regulator, MerR family |
| contig_3213 | 535 | 311 | - | Transcriptional regulator, MerR family |
| contig_3219 | 186 | 455 | + | DegV family protein |
| contig_322 | 46 | 1080 | + | Fatty acid desaturase (EC 1.14.99.-) |
| contig_322 | 1459 | 1133 | - | / Glutamine transport system permease protein GlnP (TC 3.A.1.3.2) |
| contig_322 | 1824 | 1462 | - | / Glutamine transport system permease protein GlnP (TC 3.A.1.3.2) |
| contig_322 | 2710 | 1916 | - | Cystine-binding periplasmic protein precursor |
| contig_322 | 3555 | 2866 | - | Glutamate transport ATP-binding protein |
| contig_3228 | 257 | 102 | - | FIG01226187: hypothetical protein |
| contig_3229 | 136 | 17 | - | Pullulanase (EC 3.2.1.41) |
| contig_3229 | 489 | 232 | - | Pullulanase (EC 3.2.1.41) |
| contig_323 | 86 | 706 | + | Stage V sporulation protein AA (SpoVAA) |
| contig_323 | 763 | 984 | + | Stage V sporulation protein AB (SpoVAB) |
| contig_323 | 1246 | 1563 | + | Stage V sporulation protein AC (SpoVAC) |
| contig_323 | 1608 | 2150 | + | Stage V sporulation protein AD (SpoVAD) |
| contig_323 | 2176 | 2625 | + | Stage V sporulation protein AD (SpoVAD) |
| contig_323 | 2629 | 2979 | + | Stage V sporulation protein AE (SpoVAE) |
| contig_323 | 3003 | 3545 | + | Stage V sporulation protein AE (SpoVAE) |
| contig_323 | 3542 | 4648 | + | Stage V sporulation protein AF (SpoVAF) |
| contig_3231 | 505 | 332 | - | hypothetical protein |
| contig_3234 | 144 | 19 | - | FIG01226009: hypothetical protein |
| contig_3237 | 494 | 381 | - | Urease accessory protein UreE |
| contig_324 | 1848 | 22 | - | Butyryl-CoA dehydrogenase (EC 1.3.99.2) |
| contig_324 | 2065 | 1949 | - | FIG01242077: hypothetical protein |
| contig_324 | 3244 | 2072 | - | 3-ketoacyl-CoA thiolase (EC 2.3.1.16) @ Acetyl-CoA acetyltransferase (EC 2.3.1.9) |
| contig_324 | 4285 | 3266 | - | Enoyl-CoA hydratase [isoleucine degradation] (EC 4.2.1.17) / 3-hydroxyacyl-CoA dehydrogenase (EC 1.1.1.35) |
| contig_324 | 4784 | 4254 | - | Enoyl-CoA hydratase [isoleucine degradation] (EC 4.2.1.17) / 3-hydroxyacyl-CoA dehydrogenase (EC 1.1.1.35) |
| contig_324 | 5649 | 4960 | - | Enoyl-CoA hydratase [isoleucine degradation] (EC 4.2.1.17) / 3-hydroxyacyl-CoA dehydrogenase (EC 1.1.1.35) |
| contig_324 | 5885 | 6031 | + | hypothetical protein |
| contig_3240 | 18 | 200 | + | Transcriptional regulator, TetR family |
| contig_3249 | 352 | 200 | - | Exosporium protein G |
| contig_325 | 28 | 1434 | + | Acetoacetyl-CoA synthetase [leucine] (EC 6.2.1.16) |
| contig_325 | 1431 | 1547 | + | hypothetical protein |
| contig_325 | 1719 | 1904 | + | Acetoacetyl-CoA synthetase [leucine] (EC 6.2.1.16) |
| contig_325 | 2249 | 2395 | + | Exosporium protein K |
| contig_3250 | 273 | 133 | - | Transcriptional regulator, GntR family domain / Aspartate aminotransferase (EC 2.6.1.1) |
| contig_3251 | 340 | 191 | - | Magnesium and cobalt transport protein CorA |
| contig_3253 | 250 | 59 | - | FIG01241628: hypothetical protein |
| contig_3257 | 27 | 218 | + | Hypothetical protein perhaps functionally coupled to transcription elongation factor GreA |
| contig_326 | 324 | 797 | + | Branched-chain amino acid transport system carrier protein |
| contig_326 | 784 | 1527 | + | Branched-chain amino acid transport system carrier protein |
| contig_326 | 1920 | 1744 | - | FIG01228193: hypothetical protein |
| contig_326 | 2093 | 1914 | - | FIG01228193: hypothetical protein |
| contig_326 | 2304 | 2140 | - | Response regulator LiaR |
| contig_326 | 2771 | 2295 | - | Response regulator LiaR |
| contig_3261 | 174 | 37 | - | Similar to ribosomal large subunit pseudouridine synthase F, group RluF1 |
| contig_3263 | 206 | 72 | - | hypothetical protein |
| contig_3266 | 288 | 103 | - | Aldehyde dehydrogenase (EC 1.2.1.3) in 4-hydroxyproline catabolic gene cluster |
| contig_327 | 1386 | 1048 | - | Oligopeptide transport ATP-binding protein OppF (TC 3.A.1.5.1) |
| contig_327 | 1753 | 1505 | - | ABC transporter, ATP-binding protein |
| contig_327 | 2762 | 1761 | - | Oligopeptide transport ATP-binding protein OppD (TC 3.A.1.5.1) |
| contig_327 | 2958 | 2719 | - | Oligopeptide transport system permease protein OppC (TC 3.A.1.5.1) |
| contig_3274 | 236 | 24 | - | UDP-galactose-lipid carrier transferase (EC 2.-.-.-) |
| contig_328 | 441 | 193 | - | Spore germination protein GerYA |
| contig_328 | 600 | 451 | - | Spore germination protein GerYA |
| contig_328 | 1292 | 849 | - | Glycerate kinase (EC 2.7.1.31) |
| contig_328 | 1560 | 2120 | + | Putative nitroreductase family protein SACOL0874 |
| contig_328 | 2235 | 2453 | + | Stage V sporulation protein involved in spore cortex synthesis (SpoVR) |
| contig_328 | 2425 | 3654 | + | Stage V sporulation protein involved in spore cortex synthesis (SpoVR) |
| contig_328 | 3608 | 3745 | + | hypothetical protein |
| contig_329 | 83 | 223 | + | hypothetical protein |
| contig_329 | 639 | 343 | - | Phage shock protein A |
| contig_329 | 965 | 681 | - | Phage shock protein A |
| contig_329 | 1164 | 1015 | - | conserved hypothetical protein |
| contig_329 | 1564 | 1136 | - | conserved hypothetical protein |
| contig_329 | 1719 | 2441 | + | Permease of the drug/metabolite transporter (DMT) superfamily |
| contig_329 | 2506 | 2724 | + | Permease of the drug/metabolite transporter (DMT) superfamily |
| contig_33 | 334 | 59 | - | Nitroreductase family protein |
| contig_33 | 886 | 737 | - | 2-hydroxychromene-2-carboxylate isomerase family protein |
| contig_33 | 1115 | 975 | - | 2-hydroxychromene-2-carboxylate isomerase family protein |
| contig_33 | 1393 | 1082 | - | 2-hydroxychromene-2-carboxylate isomerase family protein |
| contig_33 | 1656 | 1465 | - | Rrf2 family transcriptional regulator, group III |
| contig_33 | 1897 | 1685 | - | Rrf2 family transcriptional regulator, group III |
[truncated: 274,913 more chars]
