# Supplementary material for: Data on genome sequencing, analysis and annotation of a pathogenic Bacillus cereus 062011msu
Source: Data Brief. 2018 Jan 3;17:15–23. doi: 10.1016/j.dib.2017.12.054 (PMC5988026; doi:10.1016/j.dib.2017.12.054)
Supplement: Supplementary file 4 — Supplementary material [file mmc6.docx]

Table S3: List of antibiotic resistance genes identified in *Bacillus cereus* 062011msu

| **Contig_ID** | **Gene** | **Resistance Type** | **Antibiotic Resistance** |
| --- | --- | --- | --- |
| contig_1013 | Beta-lactamase class A | bl2a_iii | penicillin |
| contig_1222, contig_3007, contig_613 | Undecaprenyl-diphosphatase | baca | bacitracin |
| contig_2168 | multidrug ABC transporter, ATP-binding protein | bcra | bacitracin |
| contig_1881 | ABC transporter, ATP-binding protein, putative | vgaa | streptogramin_a |
| contig_2199, contig_2718 | Chloramphenicol acetyltransferase | cata6 | chloramphenicol |
| contig_2010 | Aminoglycoside 6-adenylyltransferase | ant6ia | streptomycin |
| contig_636 | Fosfomycin resistance protein FosB | fosb | fosfomycin |
| contig_343 | beta-lactamase II | bl2a_1 | penicillin |
| contig_93 | Multidrug resistance protein B | blt | chloramphenicol, doxorubicin, fluoroquinolone, puromycin |
| contig_688 | Penicillin-binding protein 3 | meca | beta_lactam |
| contig_686 | Penicillin-binding protein 2B | pbp2x | penicillin |
| contig_2962 | bacitracin transport permease protein bcrc | bcrc | bacitracin |
| contig_803 | drug resistance transporter, EmrB/QacA subfamily | lmrb | lincomycin |
| contig_561 | fosmidomycin resistance protein | rosa | fosmidomycin |
