# Supplementary material for: Data on genome sequencing, analysis and annotation of a pathogenic Bacillus cereus 062011msu
Source: Data Brief. 2018 Jan 3;17:15–23. doi: 10.1016/j.dib.2017.12.054 (PMC5988026; doi:10.1016/j.dib.2017.12.054)
Supplement: Supplementary file 5 — Supplementary material [file mmc7.docx]

Table S4: Top 20 closest neighboring strains of *Bacillus cereus* 062011msu based on RAST annotation

| **Genome ID** | **Score** | **Genome Name** |
| --- | --- | --- |
| 1053166.3 | 544 | *Bacillus cereus* AND1407 |
| 1053222.3 | 406 | *Bacillus cereus* MSX-D12 |
| 1053182.3 | 387 | *Bacillus cereus* BAG3O-2 |
| 1053184.3 | 374 | *Bacillus cereus* BAG3X2-2 |
| 1053228.3 | 371 | *Bacillus cereus* VD102 |
| 1053221.3 | 367 | *Bacillus cereus* MSX-A12 |
| 1053185.3 | 365 | *Bacillus cereus* BAG4O-1 |
| 1053186.3 | 356 | *Bacillus cereus* BAG4X12-1 |
| 1053216.3 | 317 | *Bacillus cereus* ISP3191 |
| 1053200.3 | 298 | *Bacillus cereus* HD73 |
| 222523.1 | 275 | *Bacillus cereus* ATCC 10987 |
| 222523.6 | 266 | *Bacillus cereus* ATCC 10987 |
| 1163722.3 | 248 | *Bacillus cereus* LCT-BC244 |
| 1053168.3 | 239 | *Bacillus cereus* BAG1O-2 |
| 1053180.3 | 229 | *Bacillus cereus* BAG2X1-2 |
| 1217984.3 | 223 | *Bacillus cereus* FRI-35 |
| 1053225.3 | 219 | *Bacillus cereus* VD045 |
| 198094.1 | 211 | *Bacillus anthracis* str. Ames |
| 1234146.3 | 197 | *Bacillus sp.* WBUNB001 |
| 269801.1 | 195 | *Bacillus cereus* G9241 |
