# Supplementary material for: Data on genome sequencing, analysis and annotation of a pathogenic Bacillus cereus 062011msu
Source: Data Brief. 2018 Jan 3;17:15–23. doi: 10.1016/j.dib.2017.12.054 (PMC5988026; doi:10.1016/j.dib.2017.12.054)
Supplement: Supplementary file 6 — Supplementary material [file mmc8.docx]

Table S5: Top 10 BLAST hit species resulted from genome alignment of *Bacillus cereus* 062011msu with other Bacillus species

| **Accession** | **Description** | **Max_score** | **Total_score** | **Query_cover** | **Identity** |
| --- | --- | --- | --- | --- | --- |
| AE017194.1 | *Bacillus cereus* ATCC 10987, complete genome | 40331 | 3.377e+06 | 89% | 99% |
| CP016316.1 | *Bacillus cereus* strain M3, complete sequence | 40234 | 3.081e+06 | 84% | 99% |
| CP003747.1 | *Bacillus cereus* FRI-35, complete genome | 40084 | 3.100e+06 | 85% | 99% |
| CP002508.1 | *Bacillus thuringiensis* serovar finitimus YBT-020, complete genome | 39873 | 2.874e+06 | 83% | 99% |
| CP023179.1 | *Bacillus cereus* strain CC-1 chromosome, complete genome | 39857 | 2.870e+06 | 83% | 99% |
| CP017016.1 | Bacillus sp. ABP14, complete genome | 39857 | 2.833e+06 | 82% | 99% |
| AP007209.1 | Bacillus cereus NC7401 genomic DNA, complete genome | 39829 | 2.895e+06 | 83% | 99% |
| CP001177.1 | Bacillus cereus AH187, complete genome | 39829 | 2.907e+06 | 83% | 99% |
| CP000227.1 | Bacillus cereus Q1, complete genome | 39796 | 2.850e+06 | 83% | 99% |
| CP023726.1 | Bacillus cereus strain BHU2  chromosome | 39390 | 2.587e+06 | 81% | 99% |
